# Mathematical model of COVID-19 spread in Turkey and South Africa: theory, methods, and applications

**DOI:** 10.1186/s13662-020-03095-w

**Published:** 2020-11-25

**Authors:** Abdon Atangana, Seda İğret Araz

**Affiliations:** 1 Institute for Groundwater Studies, Faculty of Natural and Agricultural Sciences, University of the Free State, Bloemfontein, South Africa; 2 Department of Medical Research, China Medical University Hospital, China Medical University, Taichung, Taiwan; 3 Department of Mathematic Education, Faculty of Education, Siirt University, Siirt, 56100 Turkey

**Keywords:** Statistical analysis, Bell curve, Prediction, New COVID-19 model, Nonlocal operators, Optimal control, Turkey vs South Africa

## Abstract

A comprehensive study about the spread of COVID-19 cases in Turkey and South Africa has been presented in this paper. An exhaustive statistical analysis was performed using data collected from Turkey and South Africa within the period of 11 March 2020 to 3 May 2020 and 05 March and 3 of May, respectively. It was observed that in the case of Turkey, a negative Spearman correlation for the number of infected class and a positive Spearman correlation for both the number of deaths and recoveries were obtained. This implied that the daily infections could decrease, while the daily deaths and number of recovered people could increase under current conditions. In the case of South Africa, a negative Spearman correlation for both daily deaths and daily infected people were obtained, indicating that these numbers may decrease if the current conditions are maintained. The utilization of a statistical technique predicted the daily number of infected, recovered, and dead people for each country; and three results were obtained for Turkey, namely an upper boundary, a prediction from current situation and lower boundary. The histograms of the daily number of newly infected, recovered and death showed a sign of lognormal and normal distribution, which is presented using the Bell curving method parameters estimation. A new mathematical model COVID-19 comprised of nine classes was suggested; of which a formula of the reproductive number, well-poseness of the solutions and the stability analysis were presented in detail. The suggested model was further extended to the scope of nonlocal operators for each case; whereby a numerical method was used to provide numerical solutions, and simulations were performed for different non-integer numbers. Additionally, sections devoted to control optimal and others dedicated to compare cases between Turkey and South Africa with the aim to comprehend why there are less numbers of deaths and infected people in South Africa than Turkey were presented in detail.

## Introduction

It was a Thursday morning, March 5, 2020, in South Africa, everybody was busy with his daily routine, when the National Institute for Communicable Diseases confirmed the first positive case of Covid-19. A situation that was known for other countries now has become true and real in South Africa. How did we get here? The outbreak of Covid-19 started in China, Wuhan City, around December 2019, but within a short period the spread crossed over to some countries in Europe like the United Kingdom, Italy, Spain, and France. The first confirmed patient was a 38-year old male who visited Italy and arrived back in South Africa on March 1, 2020. The patient, after noticing symptoms of fever, malaise, a sore throat, cough, and headache consulted a private general practitioner on March 3. From 5 March 2020 to 15 March 2020 the number of infected people increased significantly, as a result on 15 March 2020 a national state of disaster was declared by the President of South Africa to mitigate the spread of Covid-19. This announcement was followed by measures including immediate travel restrictions and closure of schools from 18 March 2020. On 23 March 2020, the South African government announced a country-wide lockdown taking effect on 26 March 2020. By the end of April, South Africa officially had 5647 confirmed cases. To date, South Africa has officially confirmed to be an African country with most confirmed cases, with 3471 active confirmed cases, 2073 recovered, and 103 deaths due to Covid-19. In the case of Turkey, the first case of Covid-19 was confirmed and recorded on 11 March 2020. Four days later, Turkey registered its first death caused by Covid-19 which spread like wildfire in Turkey; by 21 April 2020 the country had confirmed approximately 95591 cases of infected people, with 14918 recovered people, and 2259 deaths recorded. The rapid spreading of Covid-19 has raised the total number of confirmed cases to 120200, of which 48900 have recovered and 3200 have died by the end of April 2020. In comparison to other countries such as Iran, it is recorded that the total number of confirmed cases in Turkey surpassed it exceedingly, resulting in Turkey being categorized as the most affected country in terms of confirmed cases within the the Middle East. Furthermore, Turkey’s total number of confirmed cases by 20 April 2020 was also recorded to exceed that of China, even though there were some concerns that the total confirmed cases in China could have been underestimated. The consideration of these statistics prompted researchers from Turkey and South Africa to undertake research in different fields of science, technology, and engineering in the last 3 months, since their future is left uncertain. As the virologists are focusing their attention on developing a vaccine that could be used to prevent the spread of the deadly virus, mathematicians rely on modeling techniques to produce multi-scenario models that could be utilized to foresee the future [1–9]. Therefore, as mathematicians our role is to use and apply mathematical tools, particularly mathematical models, on suggested scenarios that could be helpful in predicting the future. In this paper, we present a detailed analysis of the spread in both countries and structured the paper as follows: Sect. 2 presents a detailed statistical analysis of Covid-19 spread in Turkey. Then we present a detailed statistical analysis of Covid-19 spread in South Africa. Also after using the inverse problem and bell curve approaches, we present the parameter estimation, followed by a comparative analysis between Turkey and South Africa. In Sect. 3, we suggest a new mathematical model of Covid-19 that takes into account nine classes, including susceptible, infected with 5 subclasses, recovered, dead, and vaccinated. Then we present the positivity of solutions of the model, as well as the reproduction number, and also deal with local and global asymptotic stability of disease-free equilibrium and endemic points. In Sect. 4 we present an analysis of the suggested model with nonlocal operators [10, 11]. In Sect. 5, we present numerical computations of the suggested mathematical model for Covid-19 using a numerical scheme for fractional and fractal-fractional operators. In Sect. 6, we present the optimal control of the disease. Finally, we present a discussion, recommendations, and conclusions, respectively.

## Statistical analysis of Covid-19 spread in Turkey and South Africa

To understand the impact of Covid-19, the numbers of daily new infected, recovered, and dead are collected all over the globe, and such a process follows a discrete approach. Thus, to understand and predict the impact of the Covid-19 on humans, statistics is associated with such collection, analysis, interpretation, organization, and presentation. We shall recall that this mathematical branch is widely applicable in numerous academic fields, for example, natural and social sciences, business, and government. Some important and useful statistical formulas are various means, variance, skewness, correlation, linear regression, Pearson’s correlation coefficient, Spearman’s rank correlation coefficient, and many orders. In this section, we present some formulas that will be used in this work for interpretation and prediction purposes. We define a dataset whose values can be chosen as $x_{1},x_{2},\dots,x_{n}$. We start with the arithmetic mean, *x̅*, which provides the mean of $x_{1},x_{2},\dots,x_{n}$. The formula of the arithmetic mean

2.1 $$ \frac{1}{n}\sum_{i=1}^{n}x_{i}. $$

The formula of the geometric mean is

2.2 $$ \Biggl( \prod_{i=1}^{n}x_{i} \Biggr) ^{1/n}. $$

The formula of the harmonic mean is

2.3 $$ \biggl( \frac{\sum_{i=1}^{n}\frac{1}{x_{i}}}{n} \biggr) ^{-1}. $$

The formula of the standard deviation is

2.4 $$ \Biggl( \frac{1}{n-1}\sum _{i=1}^{n} ( x_{i}-\overline{x} ) ^{2} \Biggr) ^{1/2}. $$

The formula of the skewness is

2.5 $$ \frac{\frac{1}{n}\sum_{i=1}^{n} ( x_{i}-\overline{x} ) ^{3}}{ ( \frac{1}{n-1}\sum_{i=1}^{n} ( x_{i}-\overline{x} ) ^{2} ) ^{3/2}}. $$

The formula of the variance is

2.6 $$ \frac{1}{n}\sum_{i=1}^{n} ( x_{i}-\overline{x} ) ^{2}\text{.} $$

The formula of the covariance is

2.7 $$ \sum_{i=1}^{n} ( x_{i}-\overline{x} ) ( y_{i}-\overline{y} ). $$

The formula of the Pearson correlation is

2.8 $$ \frac{\sum_{i=1}^{n} ( x_{i}-\overline{x} ) ( y_{i}-\overline{y} ) }{\overline{x}\overline{y}}. $$

The formula of the Spearman correlation is

2.9 $$ 1- \frac{6\sum_{i=1}^{n} ( \mathrm{rank} x_{i}-\mathrm{rank} y_{i} ) ^{2}}{n ( n^{2}-1 ) } ,$$

where rank enables to compare a numeric value with other values in the same list.

### Statistical analysis for Turkey

**Figure 1:**
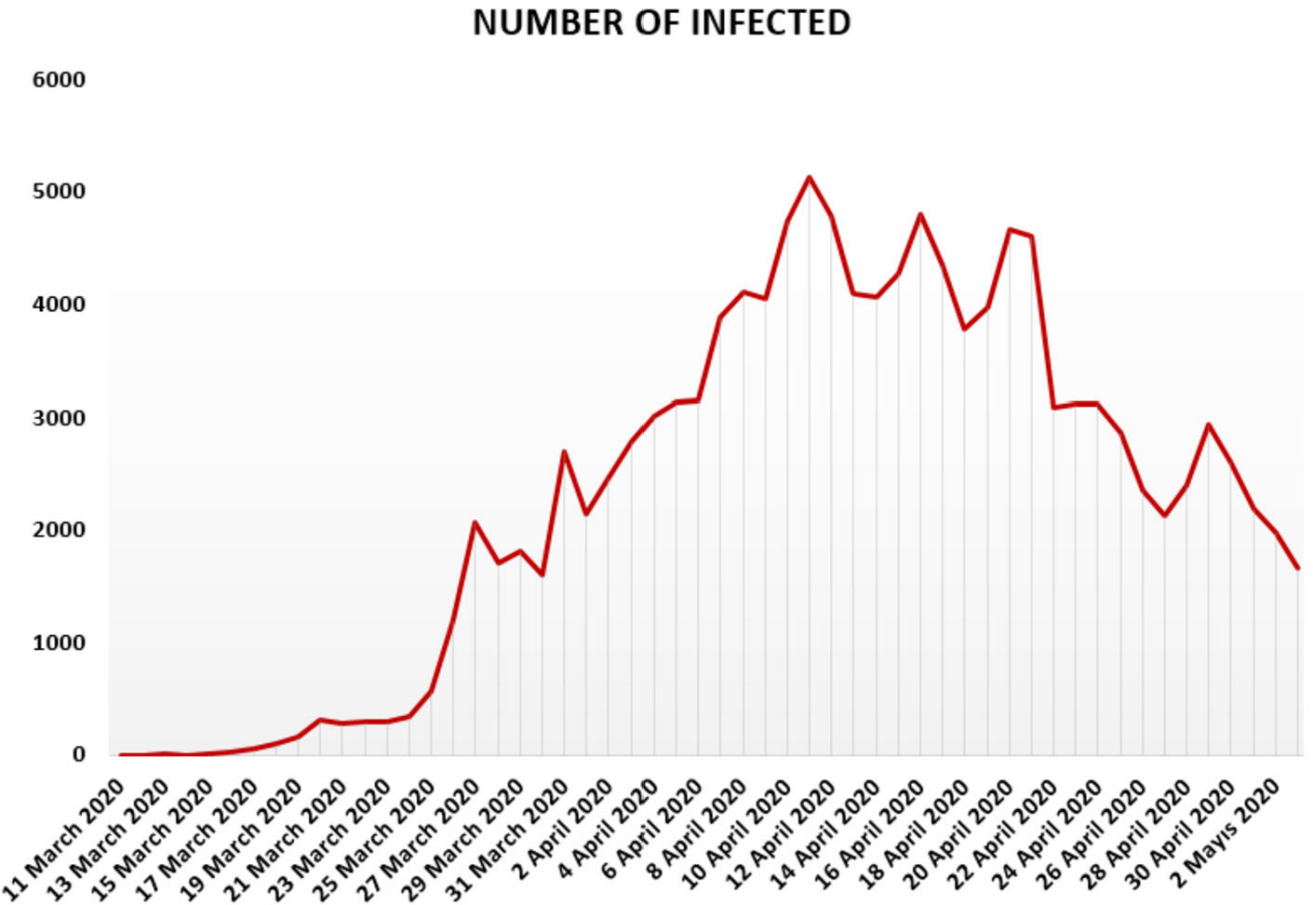
Number of infected people in Turkey from 11 March 2020 to 3 May 2020

**Figure 2:**
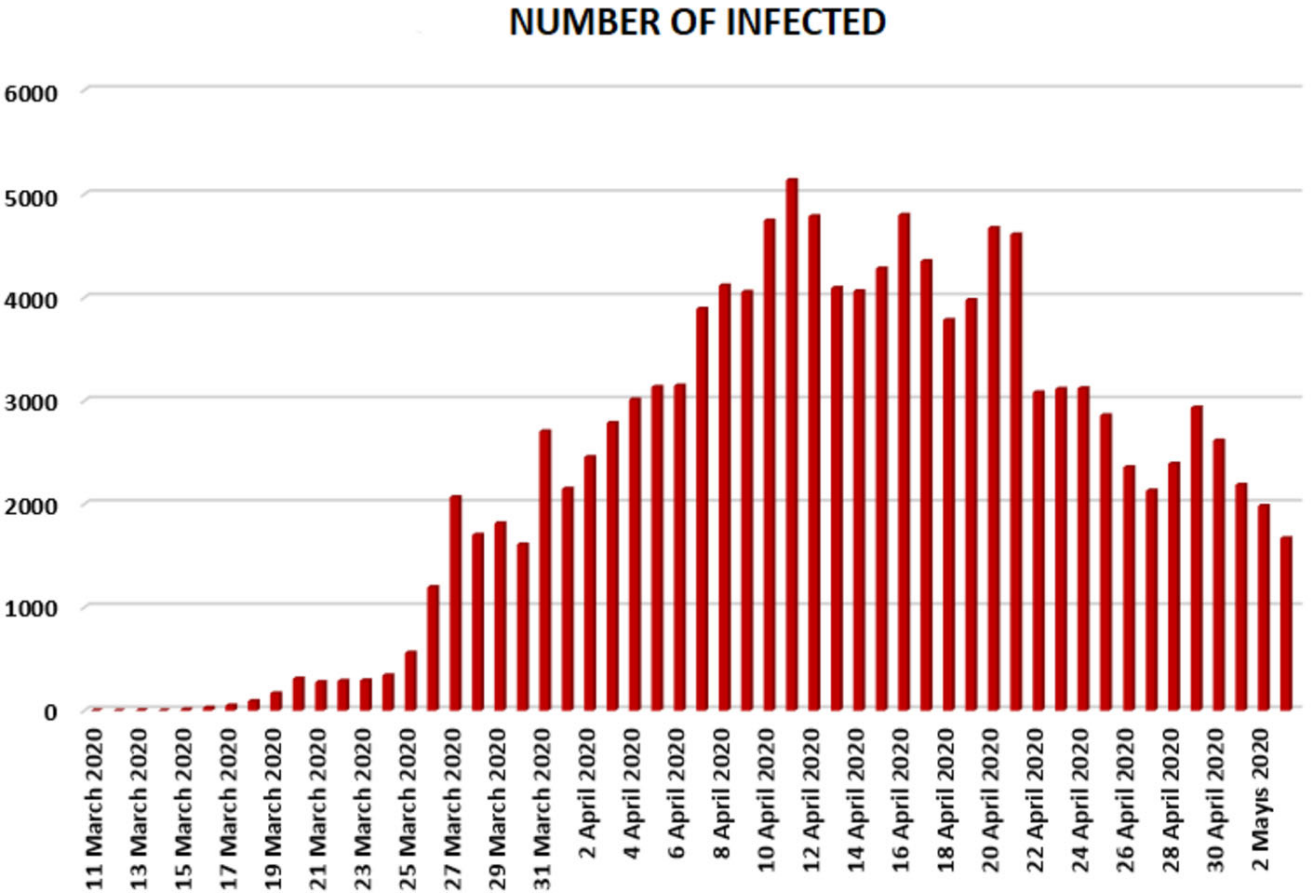
Number of infected people in Turkey from 11 March 2020 to 3 May 2020

**Figure 3:**
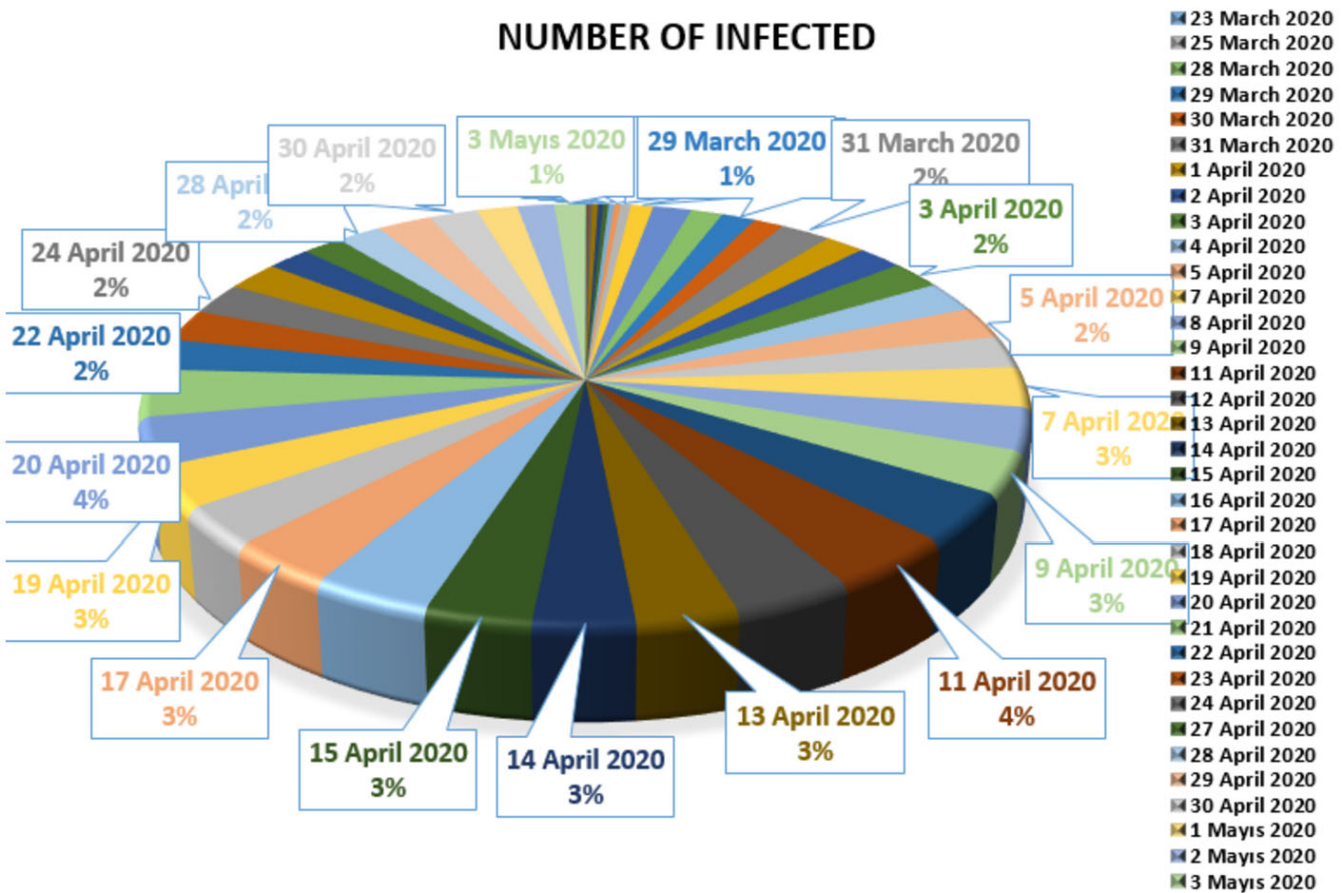
Number of infected people in Turkey from 11 March 2020 to 3 May 2020

**Figure 4:**
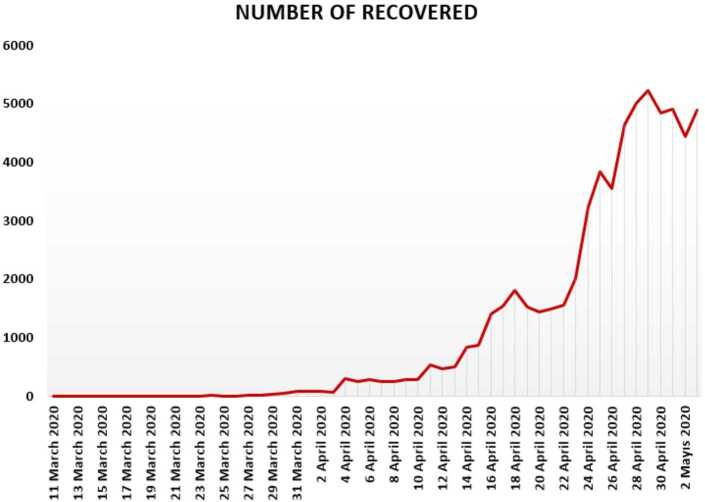
Number of recovered people in Turkey from 11 March 2020 to 3 May 2020

**Figure 5:**
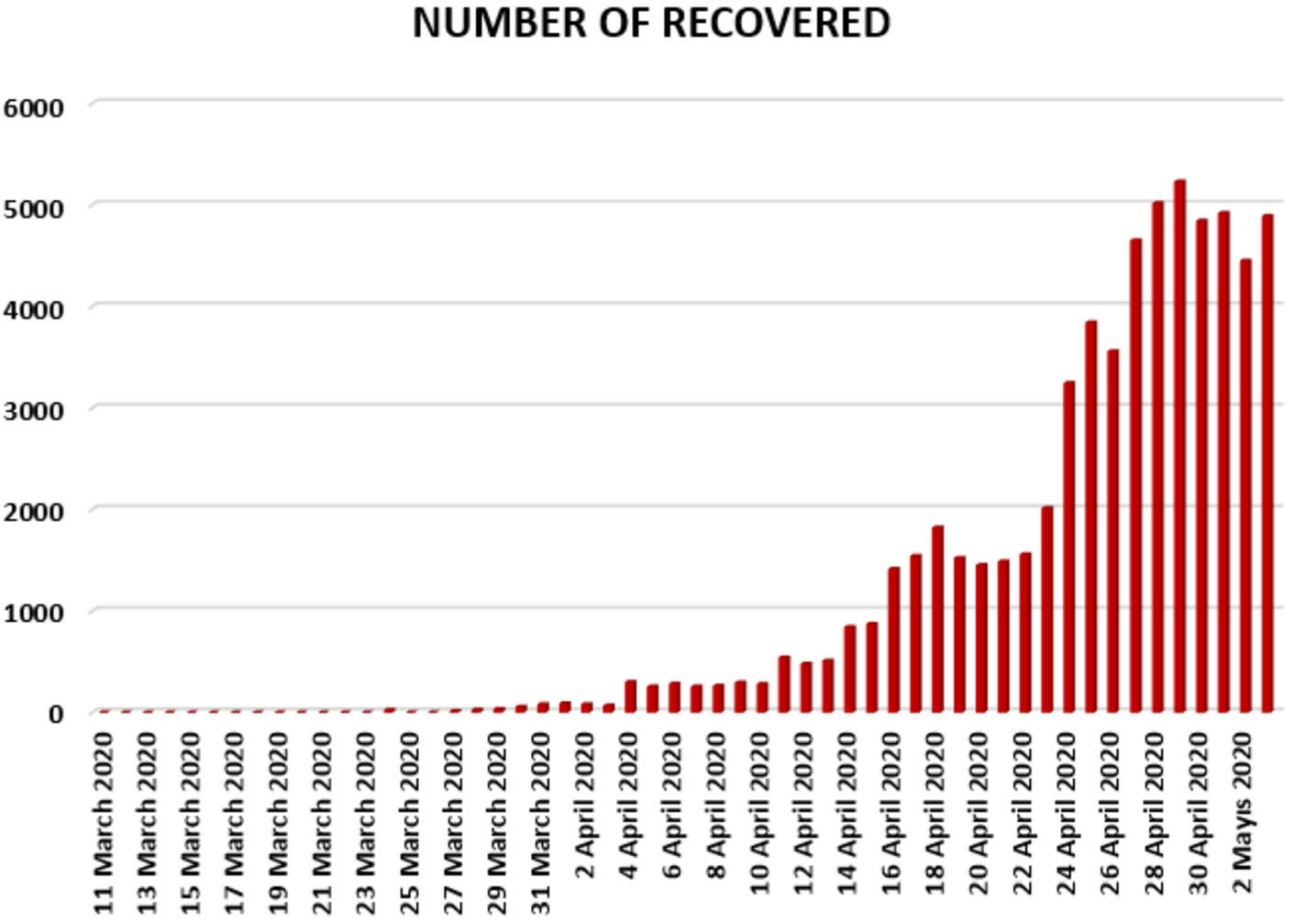
Number of recovered people in Turkey from 11 March 2020 to 3 May 2020

**Figure 6:**
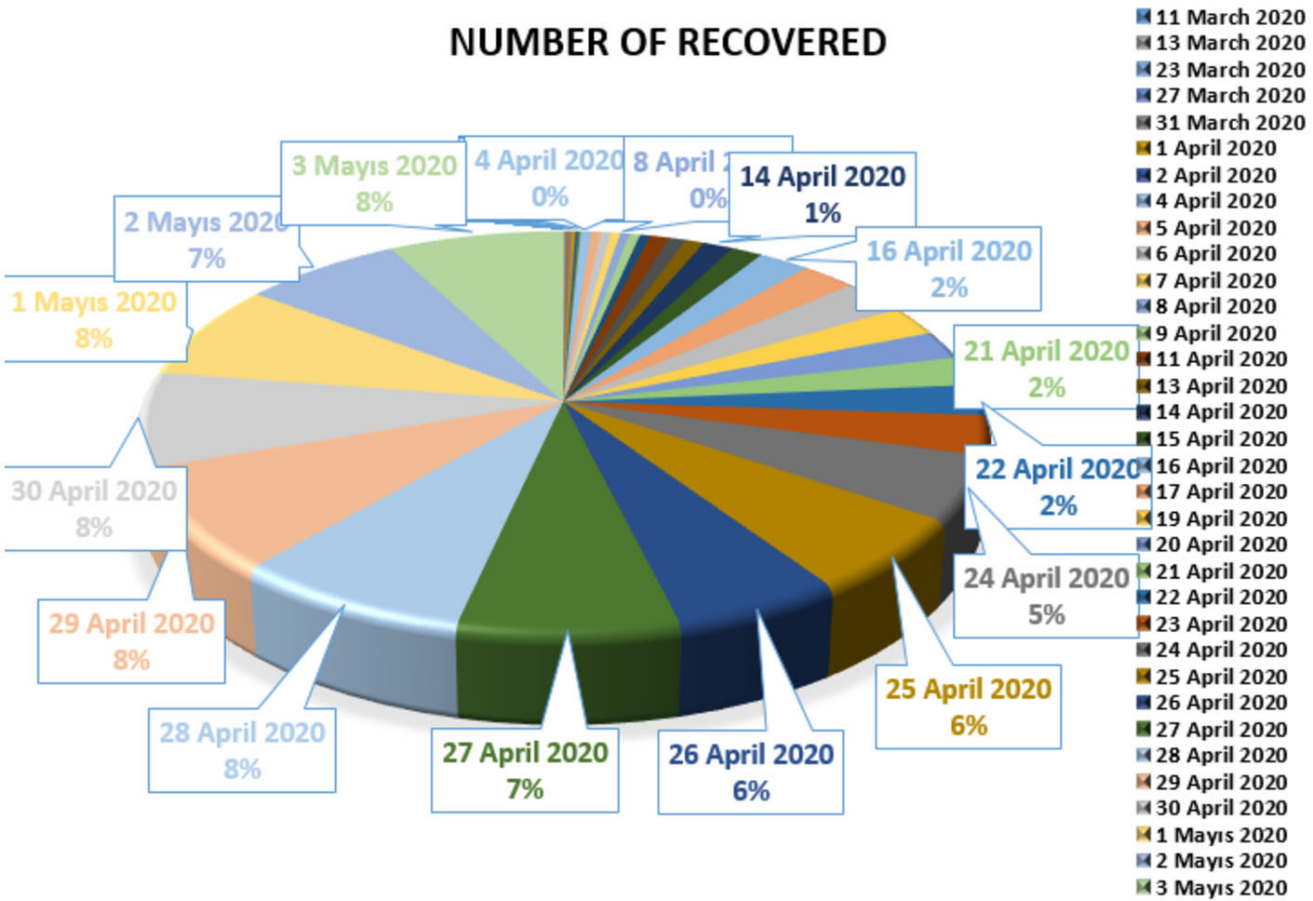
Number of recovered people in Turkey from 11 March 2020 to 3 May 2020

**Figure 7:**
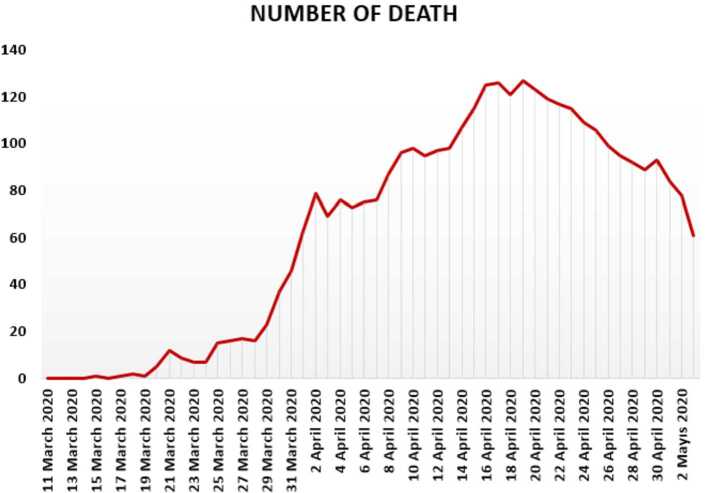
Number of dead people due to Covid-19 in Turkey from 11 March 2020 to 3 May 2020

**Figure 8:**
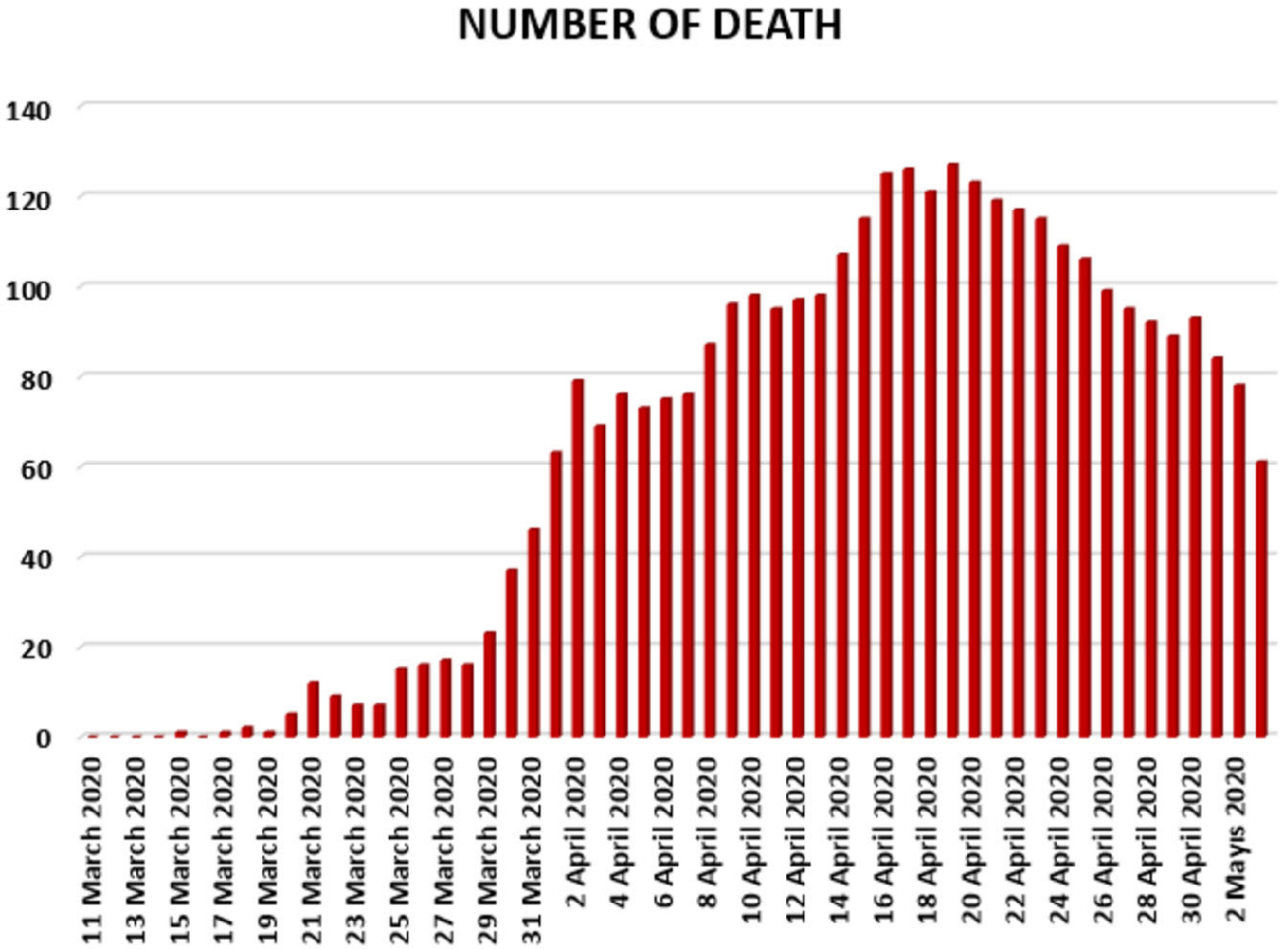
Number of dead people due to Covid-19 in Turkey from 11 March 2020 to 3 May 2020

**Figure 9:**
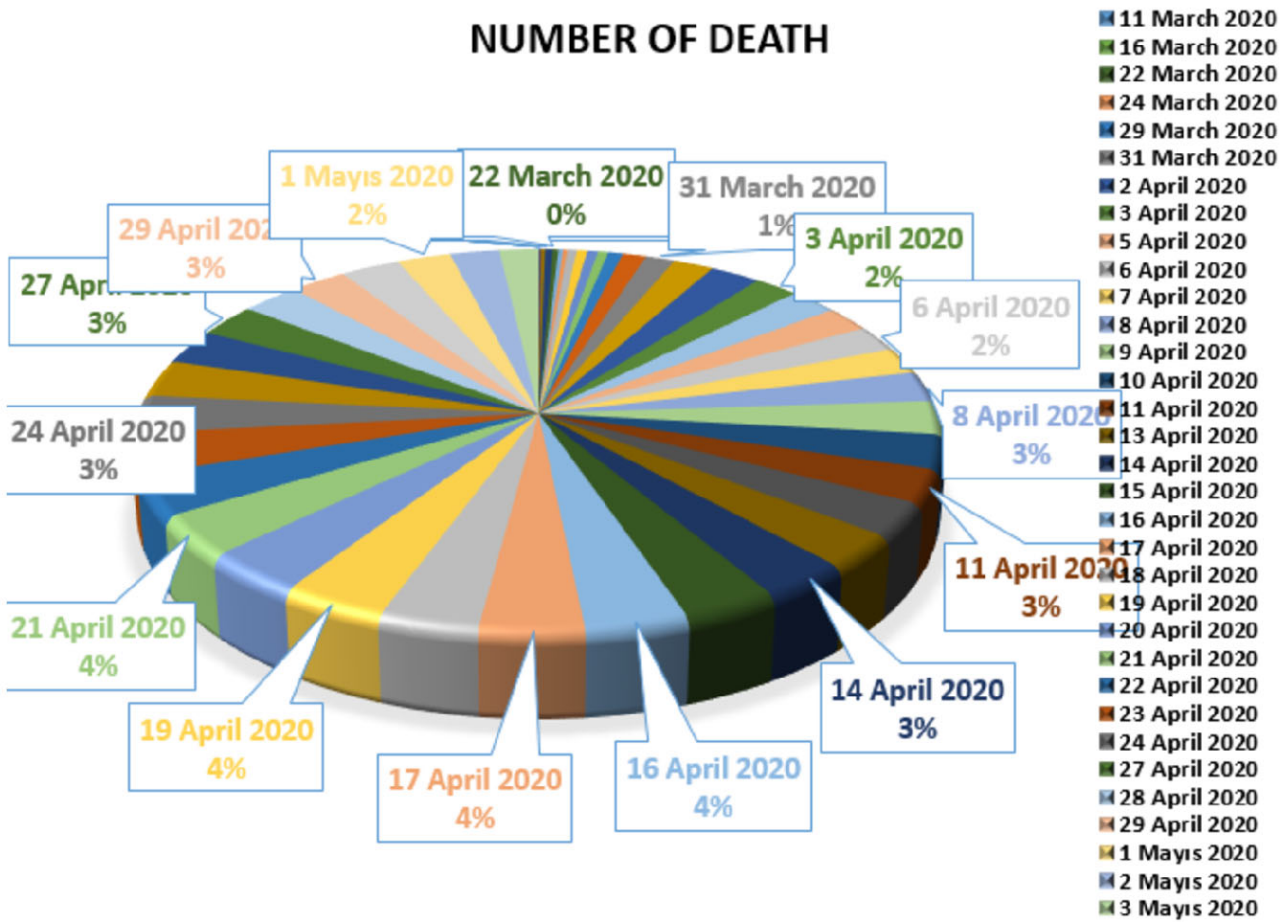
Number of dead people due to Covid-19 in Turkey from 11 March 2020 to 3 May 2020

**Figure 10:**
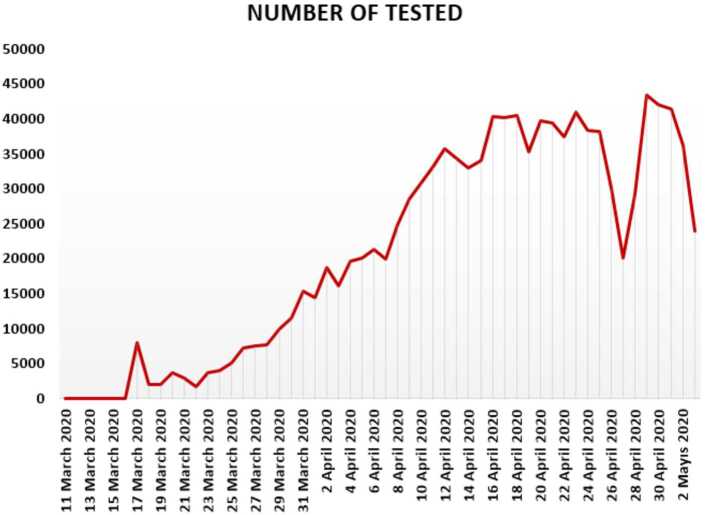
Number of tested people in Turkey from 11 March 2020 to 3 May 2020

**Figure 11:**
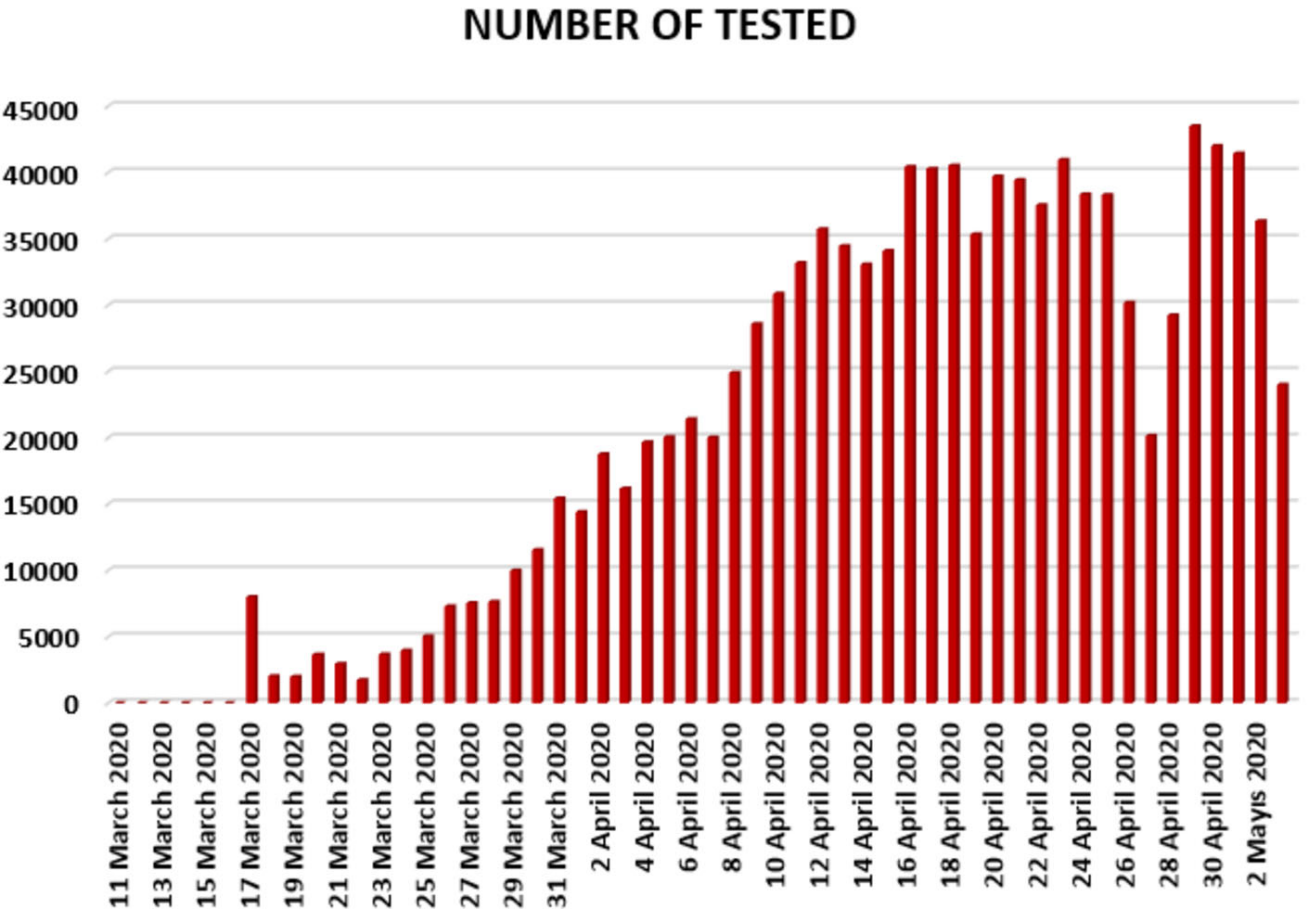
Number of tested people in Turkey from 11 March 2020 to 3 May 2020

**Figure 12:**
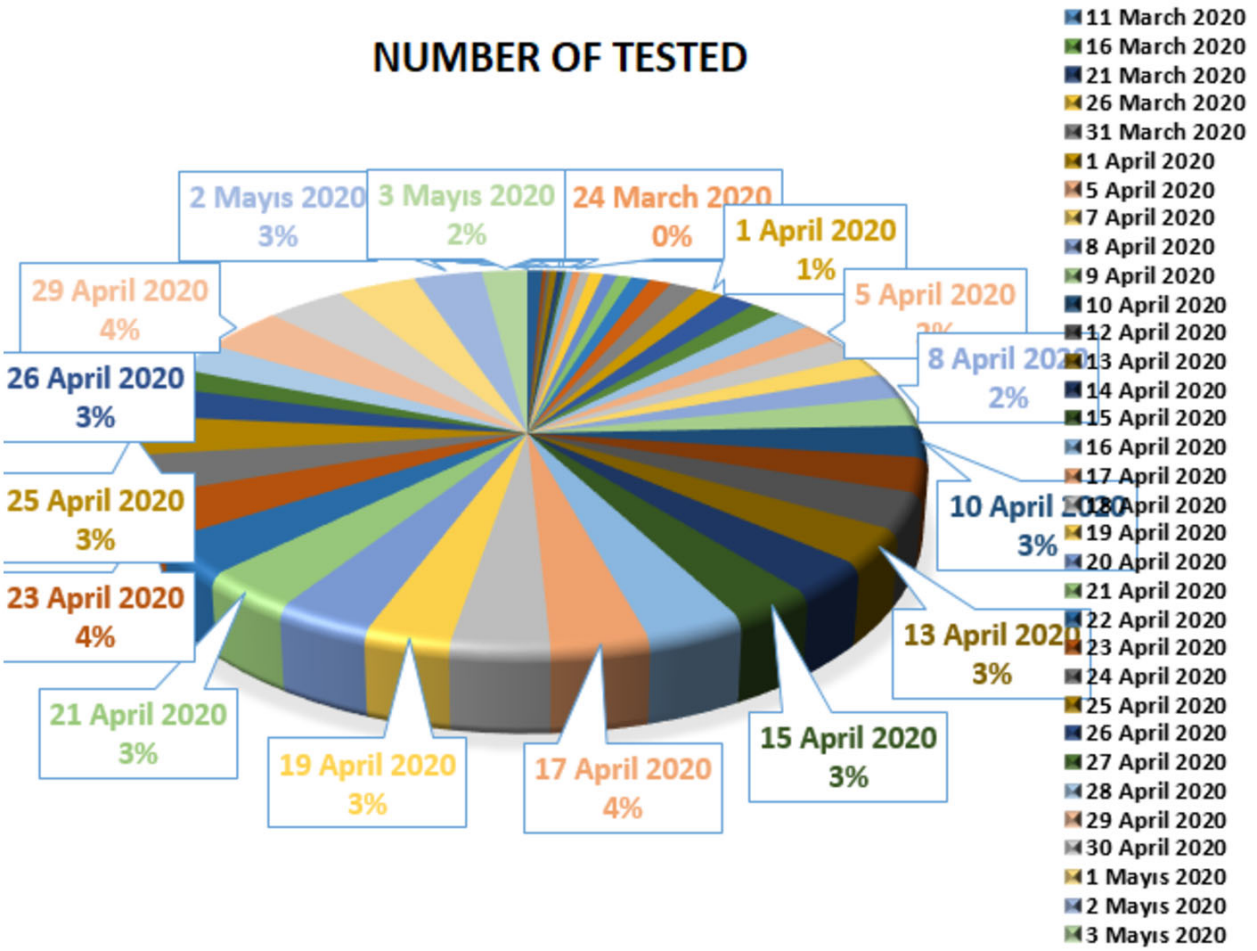
Number of tested people in Turkey from 11 March 2020 to 3 May 2020

**Figure 13:**
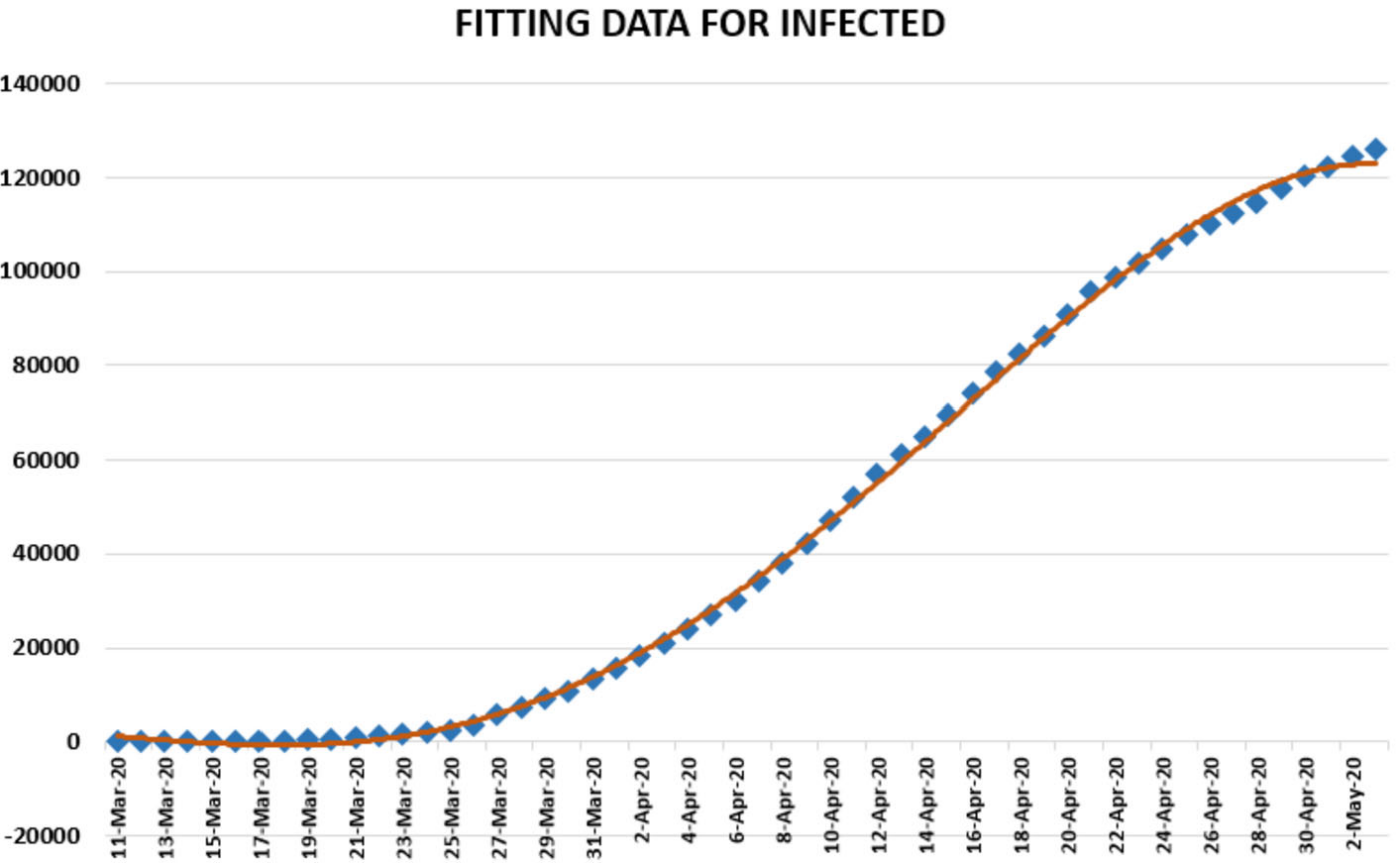
Polynomial fitting for infected people in Turkey from 11 March 2020 to 3 May 2020

**Figure 14:**
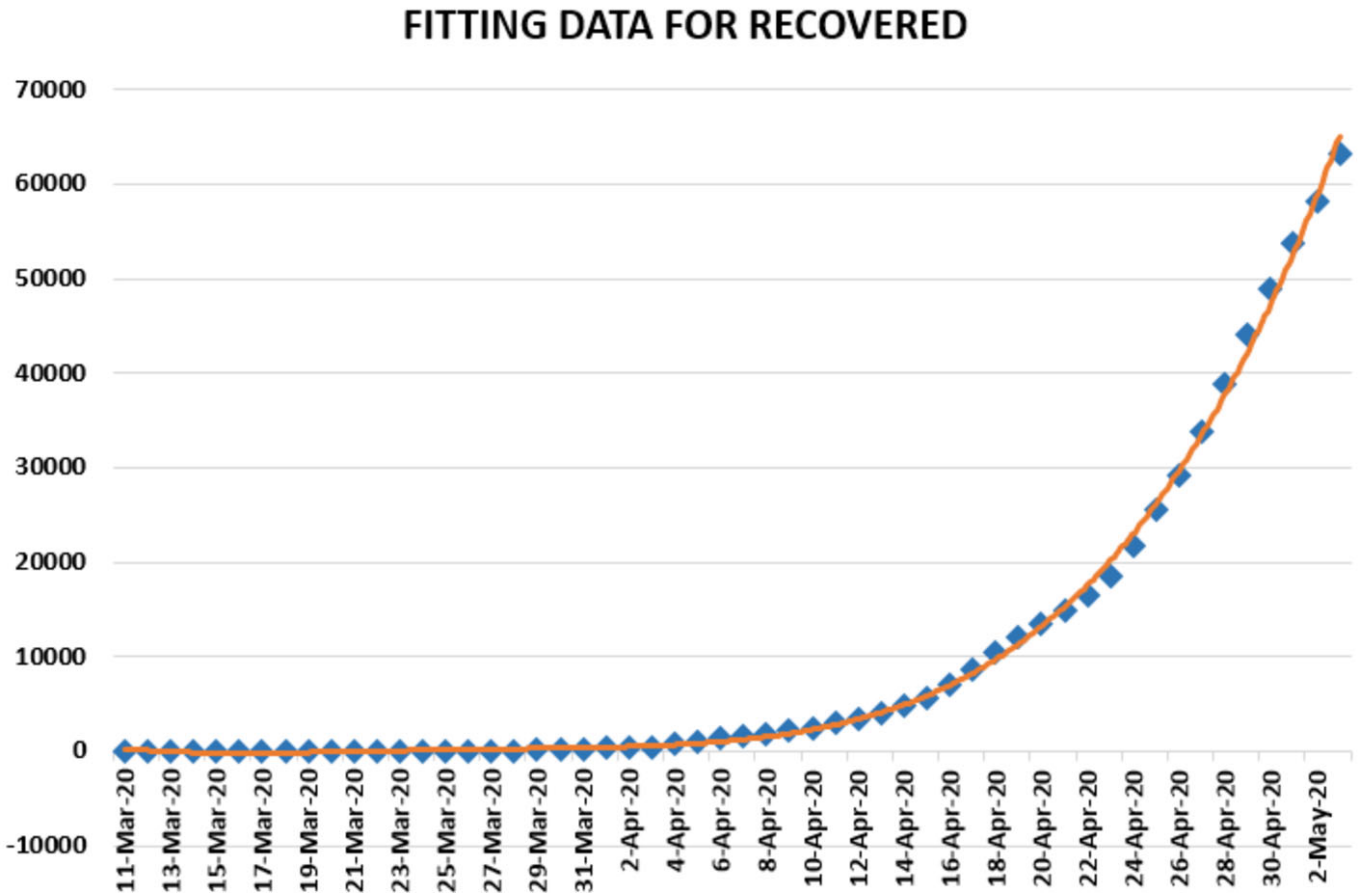
Polynomial fitting for recovered people in Turkey from 11 March 2020 to 3 May 2020

**Figure 15:**
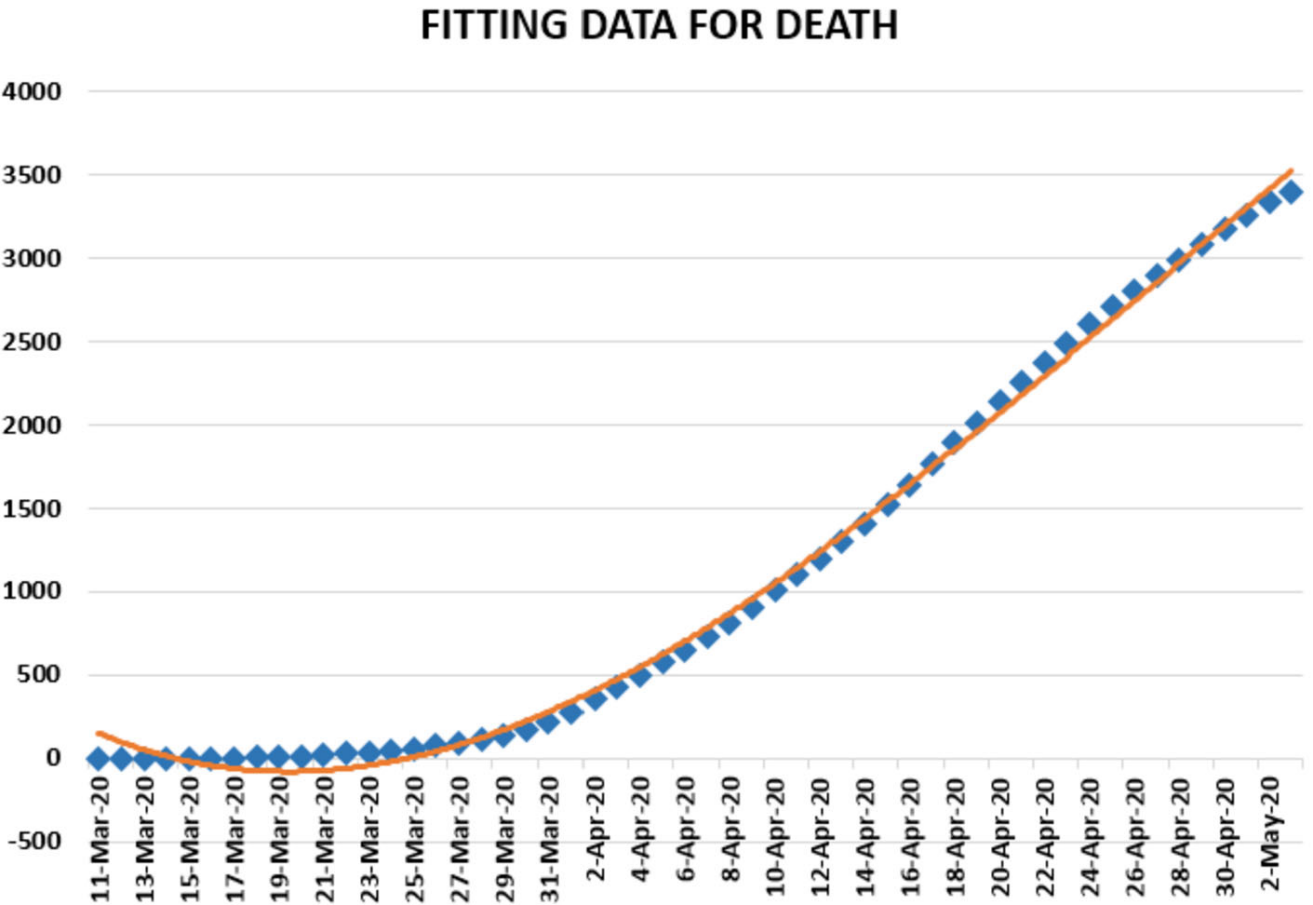
Polynomial fitting for dead people in Turkey from 11 March 2020 to 3 May 2020

**Figure 16:**
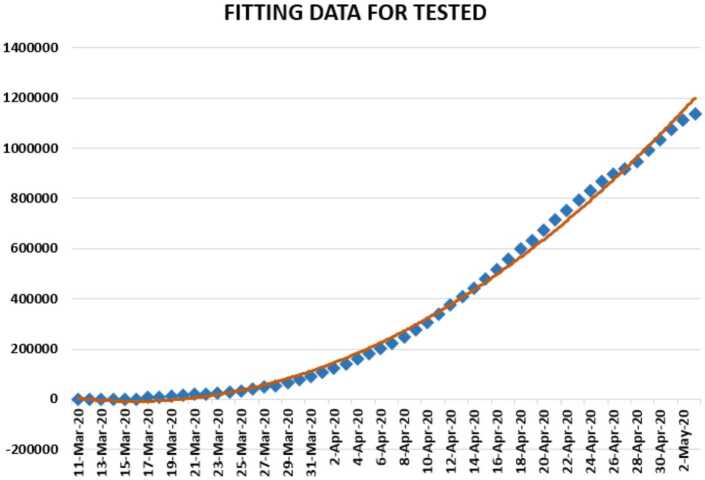
Polynomial fitting for tested people in Turkey from 11 March 2020 to 3 May 2020

In this section, we aim to provide a detailed statistical analysis of the collected data from Turkey. These data include the daily numbers of new infected, dead, recovered, and finally, tested individuals. The collected data are from 11 March 2020 to 3 May 2020. The main aim of this section is to predict what could possibly happen in the near future using the reliability level method, additionally, to find which distribution each class follows. With the collected data, we will first present a histogram, pie chart, and nonlinear graphs for each class. The histograms will help identify the density of probability associated to each set of collected data. Additionally, we provide a polynomial fitting against collected. The results are presented in Figs. 1 to 16. For each case, we present arithmetic, geometric, and harmonic means, as well as skewness, variance, covariance, Pearson correlation and Spearman correlation, and these are presented in Table 1.

**Table 1:** Some data about coronavirus cases in Turkey

	Infected	Recovered	Dead
Arithmetic mean	2334.166667	1169.462963	56.63636364
Geometric mean	1029.876948	592.2822821	41.57620243
Harmonic mean	21.75203938	142.4039454	8.182426471
Standard deviation	1641.461139	1701.397957	48.05766445
Skewness	−0.07519891	1.416616957	0.100241163
Variance	2644498.472	2841148.434	2045.624143
Covariance	17324.10185	21927.12037	607.7037037
Pearson Correlation	0.683518717	0.834653045	0.862085432
Spearman Correlation	−17.9276729	0.188679245	−8.39622641

In Figs. 1, 2, and 3, we present some statistical simulation of the number of infected people due to Covid-19 in Turkey from 11 March 2020 to 3 May 2020.

In Figs. 4, 5, and 6, we present some statistical simulation of the number of recovered people due to Covid-19 in Turkey from 11 March 2020 to 3 May 2020.

In Figs. 7, 8, and 9, we present some statistical simulation of the number of dead people due to Covid-19 in Turkey from 11 March 2020 to 3 May 2020.

In Figs. 10, 11, and 12, we present some statistical simulation about number of tested people due to Covid-19 in Turkey from 11 March 2020 to 3 May 2020.

#### Regression analysis

Regression analysis which is also used in epidemiologic research enables us to examine relationships among a set of variables. Here the aim is to estimate outcomes benefitting from this set of variables. To do this, we find a prediction model in which we obtain a model that best fits the considered data and explains the response variable. We can utilize all possible independent variables, interactions, and transformations of these models. To evaluate goodness of fit for the obtained model, we can utilize $R^{2}$ measure which is one of the different techniques used in regression diagnostics.

Linear regression models are given by

2.10 $$ y=\beta _{0}+\beta _{i}x_{i}+e_{i}, $$

where $\beta _{0},\beta _{i}$ are the unknown constants, $x_{i}$ are the independent variables, *y* is the dependent variable, and $e_{i}$ are the error terms in given data. If the value of $R^{2}$ is close to zero, this means that the significance of fit for the model is unsuitable to predict outcomes. In other words, the obtained model is not suitable for the given data and it should be discarded in favor of another model that should be found.

If the value of $R^{2}$ is close to one, this means that the significance of fit for the model is suitable to predict outcomes. In this case, it can be passed to the following step of control analysis.

We first present a predictive analysis for infected people. According to the results obtained, we obtain a linear regression which is calculated as

2.11 $$ y=-29772.4+2786.833x. $$

The *F*-test statistic was calculated as $1.94\times 10^{-32}$, while $R^{2}$ was calculated as 0.93445. We can conclude from these values that the significance of fit for the obtained model is suitable for the considered data. Also we present a polynomial regression which is calculated as

2.12 $$ y=-0.0551x^{4}+9682.6x^{3}-0.6\times 10^{-8}x^{2}+0.2 \times 10^{-13}x-0.2 \times 10^{-17}. $$

For this polynomial, $R^{2}$ was calculated to be 0.9993. We present polynomial fitting data for infected people from 11 March 2020 to 3 May 2020.

We present a predictive analysis for recovered people. According to the results obtained, we get a linear regression, which can be calculated as

2.13 $$ y=-13029.8+845.9233x. $$

The *F*-test statistic was calculated to be $1.39\times 10^{-12}$, while $R^{2}$ was calculated as 0.622381. We can say that the significance of fit for this model is not good enough for the considered data. To overcome this issue, we suggest another regression model

2.14 $$ y=0.028x^{4}-4922x^{3}+0.3\times 10^{-8}x^{2}-0.9 \times 10^{-12}x+0.1 \times 10^{-17}, $$

which is polynomial. For this polynomial, $R^{2}$ was calculated to be 0.9987. We present a simulation using polynomial fitting for the data of recovered people from 11 March 2020 to 3 May 2020.

We present a predictive analysis for dead people. According to the results obtained, we get a linear regression which can be calculated as

2.15 $$ y=-822.246+70.72746x. $$

The *F*-test statistic was calculated to be $1.75\times 10^{-28}$, while $R^{2}$ was calculated as 0.907007. We can say that the significance of fit for model is suitable for the considered data. Also, we can present the following regression model:

2.16 $$ y=-0.0266x^{3}+3512.1x^{2}-0.2\times 10^{-8}x+0.2 \times 10^{-12}, $$

which is polynomial of third order. For this polynomial, $R^{2}$ was calculated to be 0.9971. We present a simulation of the polynomial fitting for dead people from 11 March 2020 to 3 May 2020.

We now present a predictive analysis for tested people. According to the results obtained, we get a linear regression which can be calculated as

2.17 $$ y=-257388+22572.98x. $$

The *F*-test statistic was calculated to be $5.17\times 10^{-28}$, while $R^{2}$ was calculated as 0.903051. We can say that the significance of fit for model is suitable for the considered data. Also, we give a polynomial regression model

2.18 $$ y=520.26x^{2}-0.5\times 10^{-7}x+0.1\times 10^{-12} ,$$

which is polynomial of second order. For this polynomial, $R^{2}$ was calculated as 0.9962. We present a simulation of polynomial fitting for tested people from 11 March 2020 to 3 May 2020.

We present some statistical data about coronavirus cases in Turkey in Table 1.

Table 2 presents the covariance, Pearson and Spearman correlation coefficients between daily cases–recovered, recovered–dead, and infected–dead related to Covid-19 in Turkey.

**Table 2:** Some data for coronavirus cases in Turkey

	Infected–Recovered	Recovered–Dead	Infected–Dead
Covariance	622993.071	38856,60837	66660.3642
Pearson Correlation	0.22728176	0.509688703	0.90632320
Spearman Correlation	0.64139508	0.817762531	0.88601105

**Figure 17:**
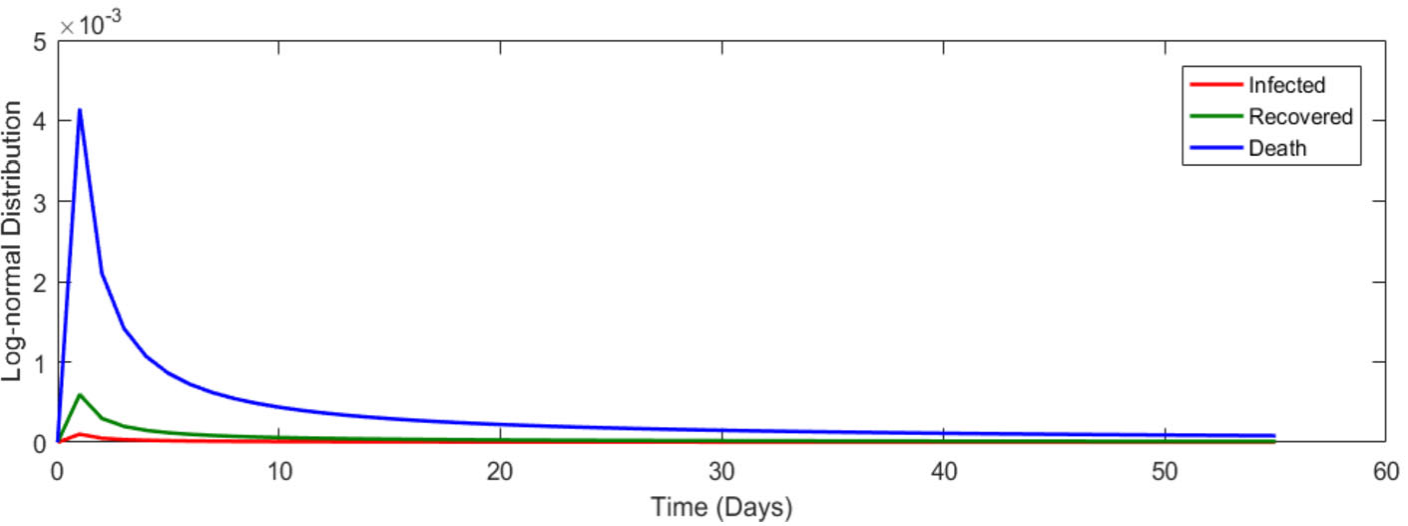
Lognormal distribution for all cases in Turkey from 11 March 2020 to 3 May 2020

We now fit a lognormal distribution for all cases in Turkey from 11 March 2020 to 03 May 2020 in Fig. 17.

#### Prediction for coronavirus data in Turkey

**Figure 18:**
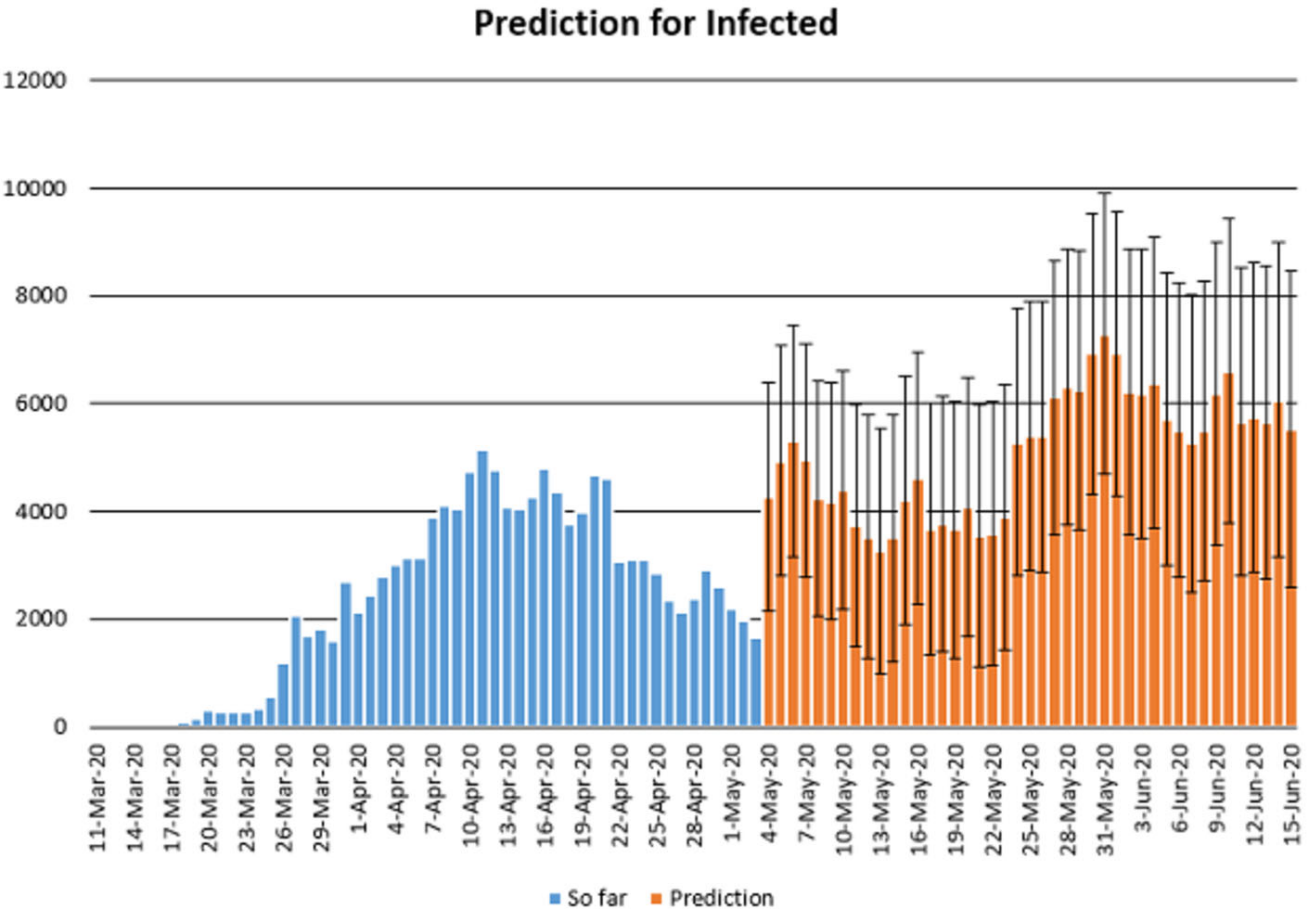
Prediction of daily number of infected in Turkey using forecast sheet with reliability level of 97%

**Figure 19:**
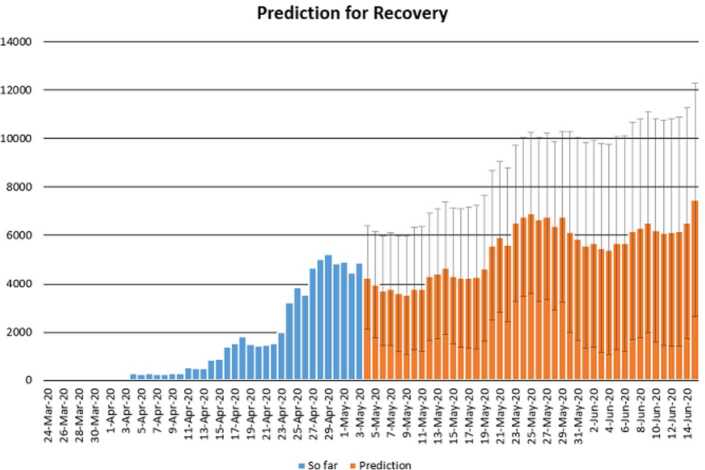
Prediction of daily number of recovered in Turkey using forecast sheet with reliability level of 97%

**Figure 20:**
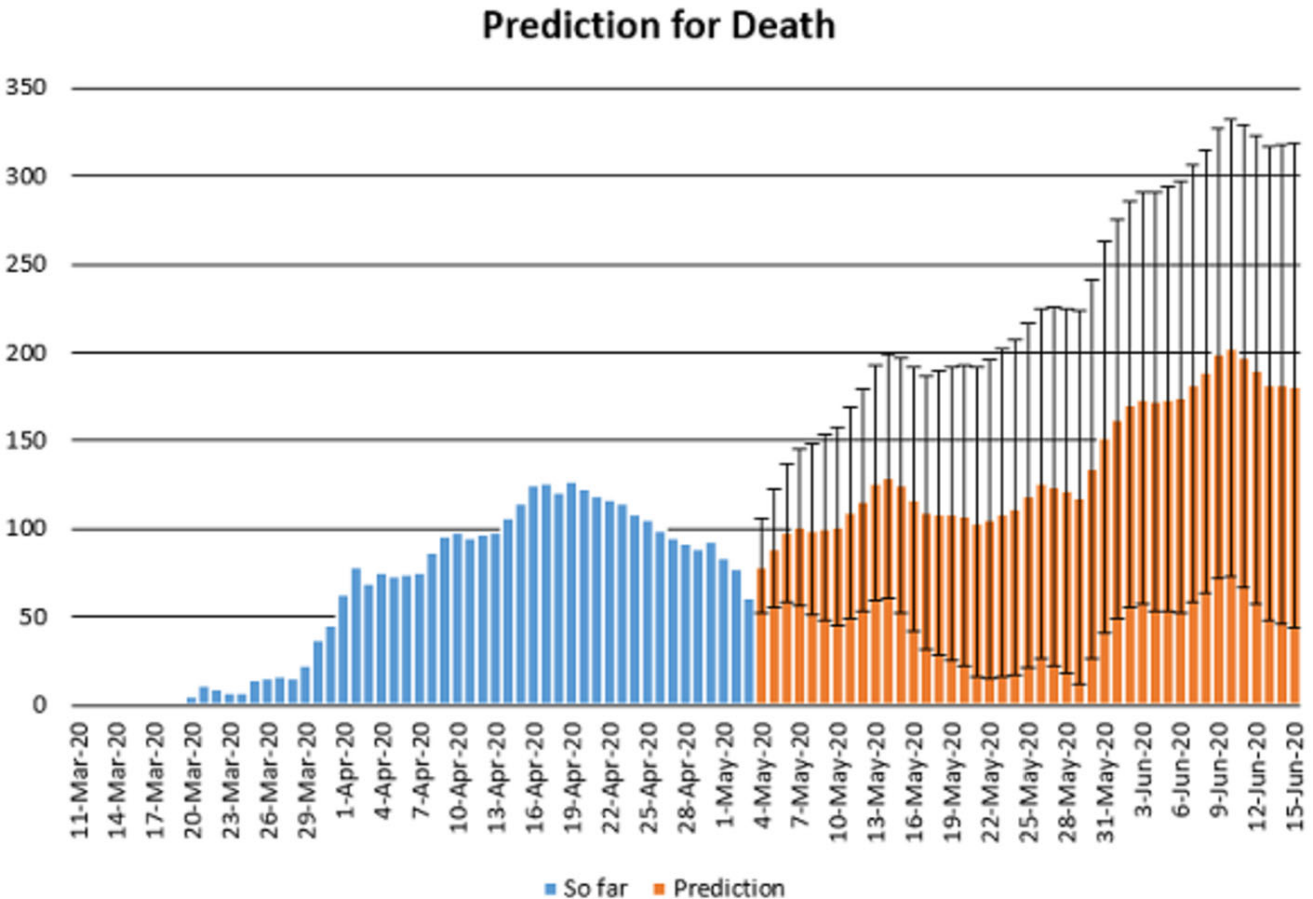
Prediction of daily number of dead in Turkey using Forecast Sheet with reliability level of 97%

In this section, we aim at performing prediction using existing data and reliability level method. The collected data will be considered from 11 March 2020 to 3 May 2020 [12]. The future prediction will start from 3 May 2020 to 15 June 2020. This will help us give a prediction on the daily numbers of new infected, recovered, and dead in Turkey within this period. The prediction will consist of three different graphs comprising upper boundaries, middle lines, and low boundaries. The upper boundaries represent the worse case scenario, of course, a scenario that is not needed for the classes of dead and infected, but an ideal one for the recovered class, and the lower boundaries represent the perfect scenario (a scenario that is needed) for Turkey to get rid of the infection. These results of prediction for future daily new infected, recovered, and dead are represented graphically in Figs. 18, 19, and 20, respectively.

### Detailed statistical analysis for South Africa

**Figure 21:**
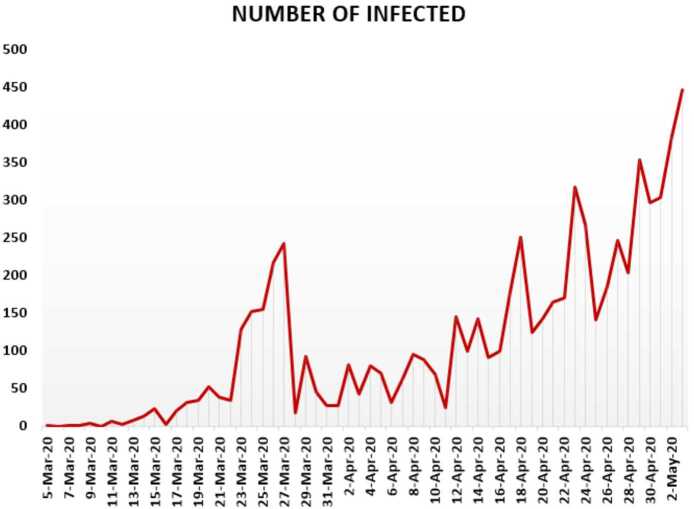
Number of infected people in South Africa from 5 March 2020 to 3 May 2020

**Figure 22:**
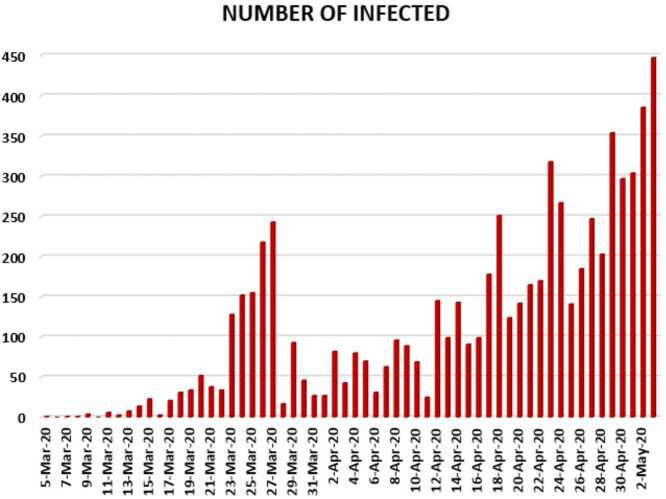
Number of infected people in South Africa from 5 March 2020 to 3 May 2020

**Figure 23:**
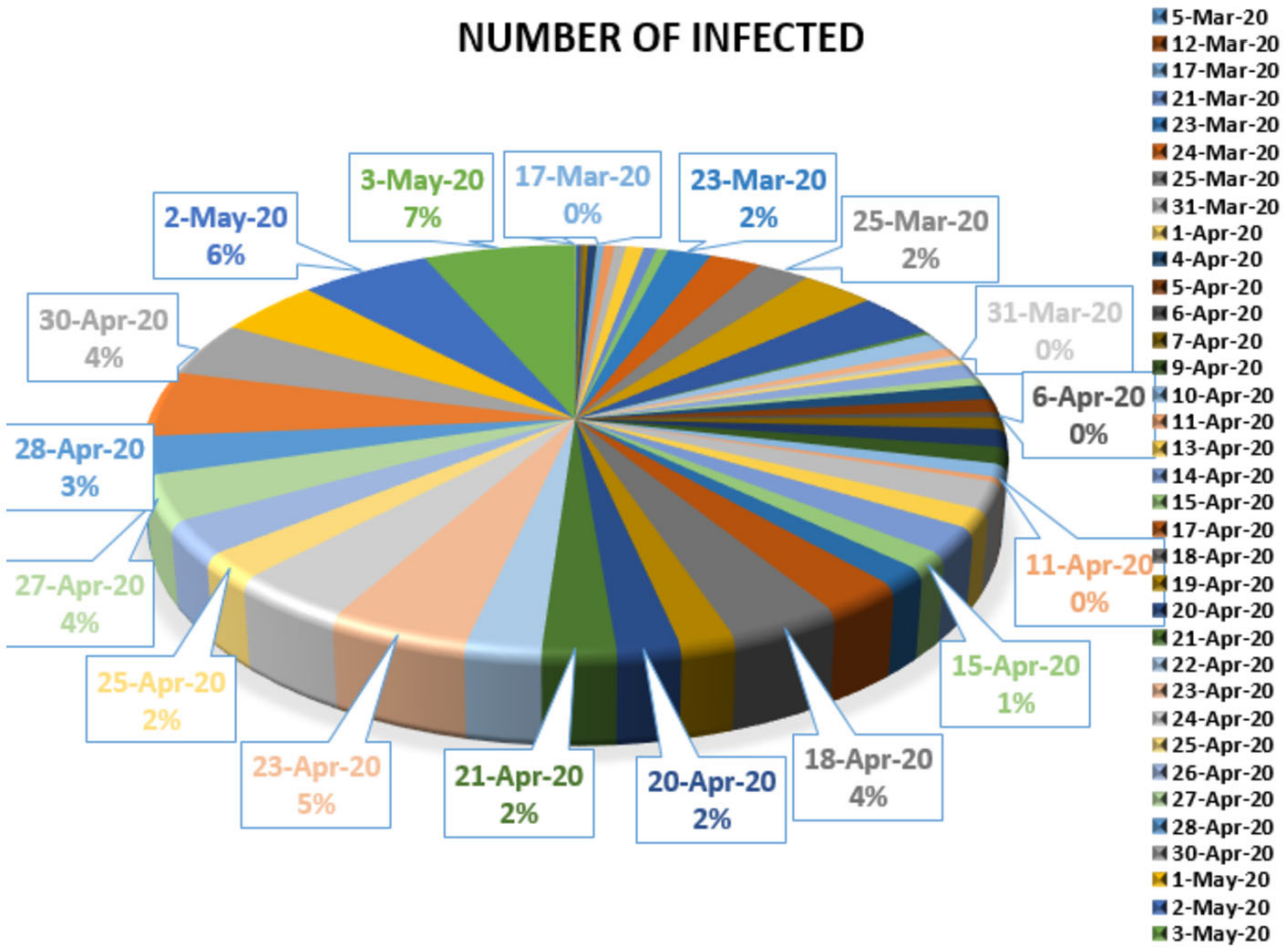
Number of infected people in South Africa from 5 March 2020 to 3 May 2020

**Figure 24:**
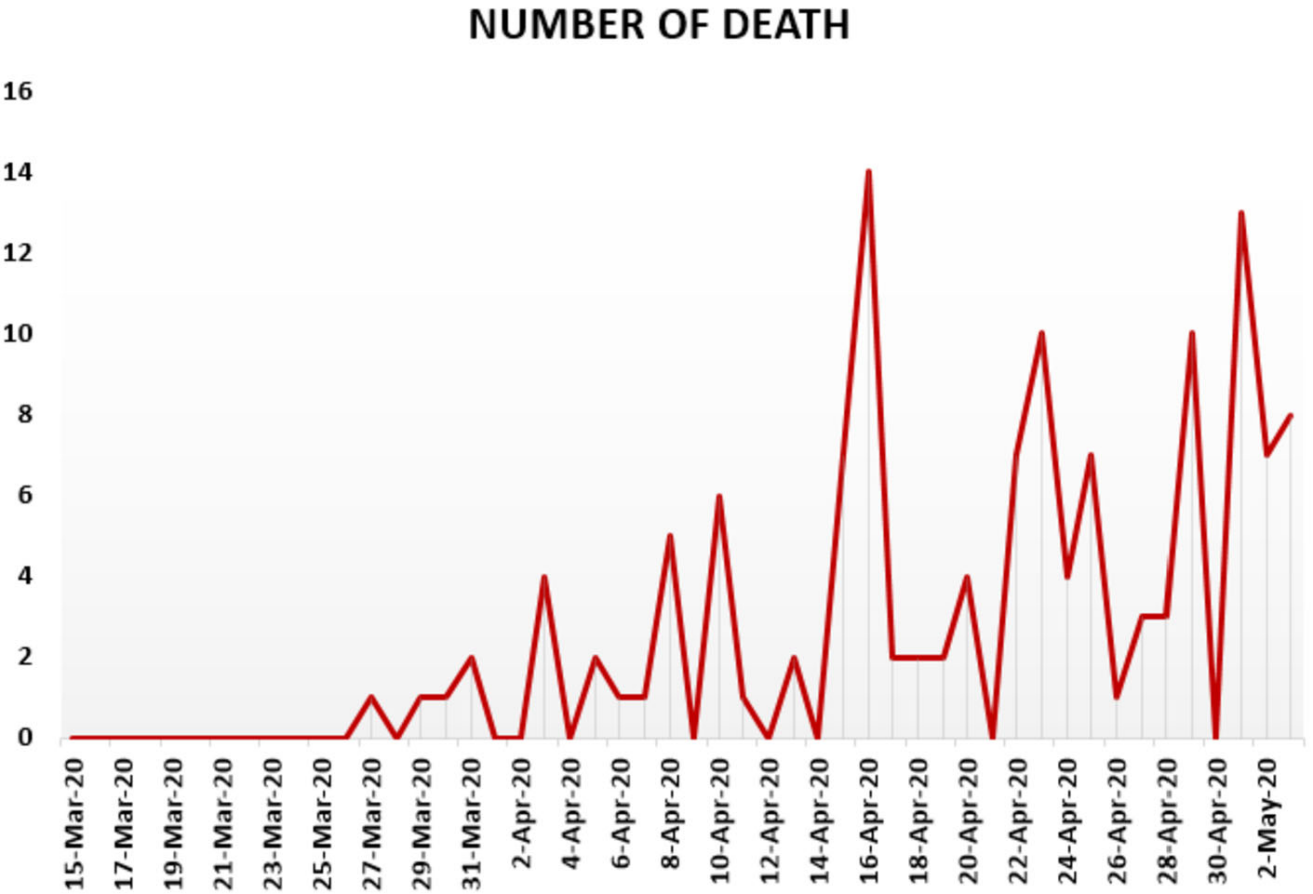
Number of dead people due to Covid-19 in South Africa from 15 March 2020 to 3 May 2020

**Figure 25:**
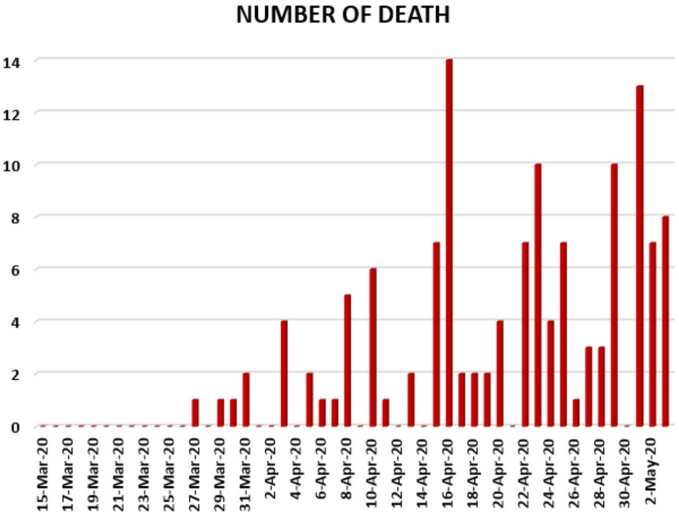
Number of dead people due to Covid-19 in South Africa from 15 March 2020 to 3 May 2020

**Figure 26:**
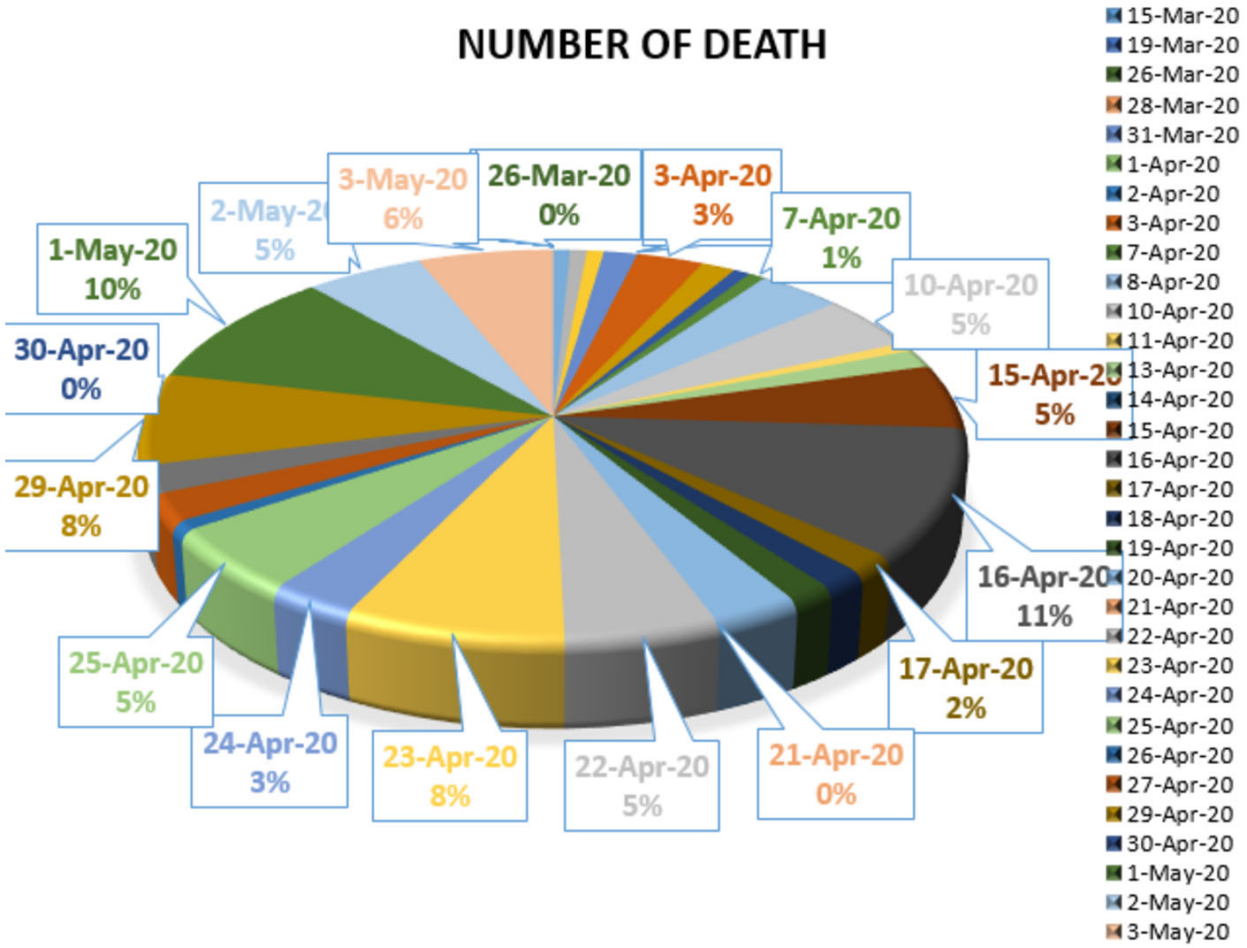
Number of dead people due to Covid-19 in South Africa from 15 March 2020 to 3 May 2020

**Figure 27:**
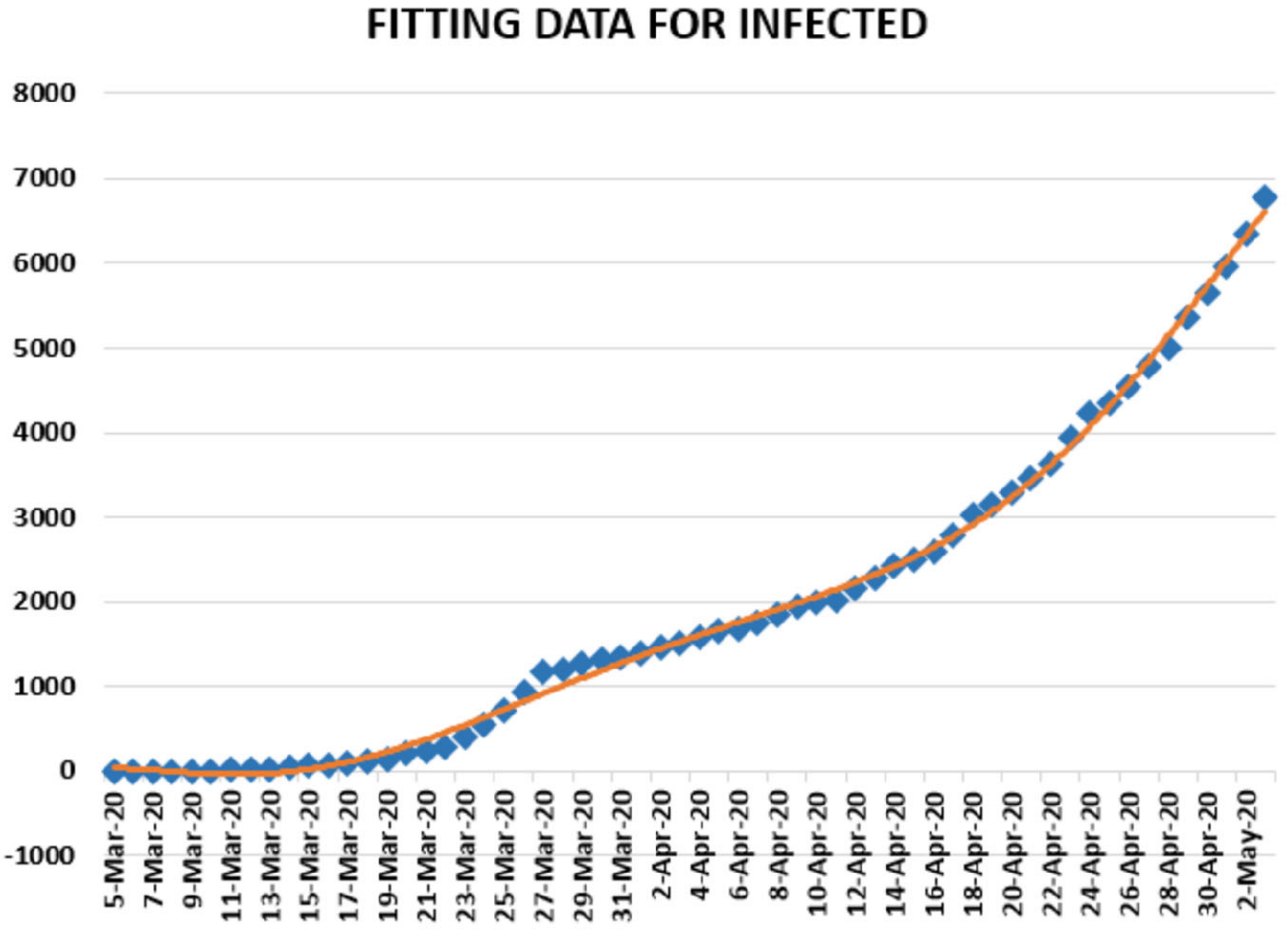
Polynomial fitting for infected in South Africa from 5 March 2020 to 3 May 2020

**Figure 28:**
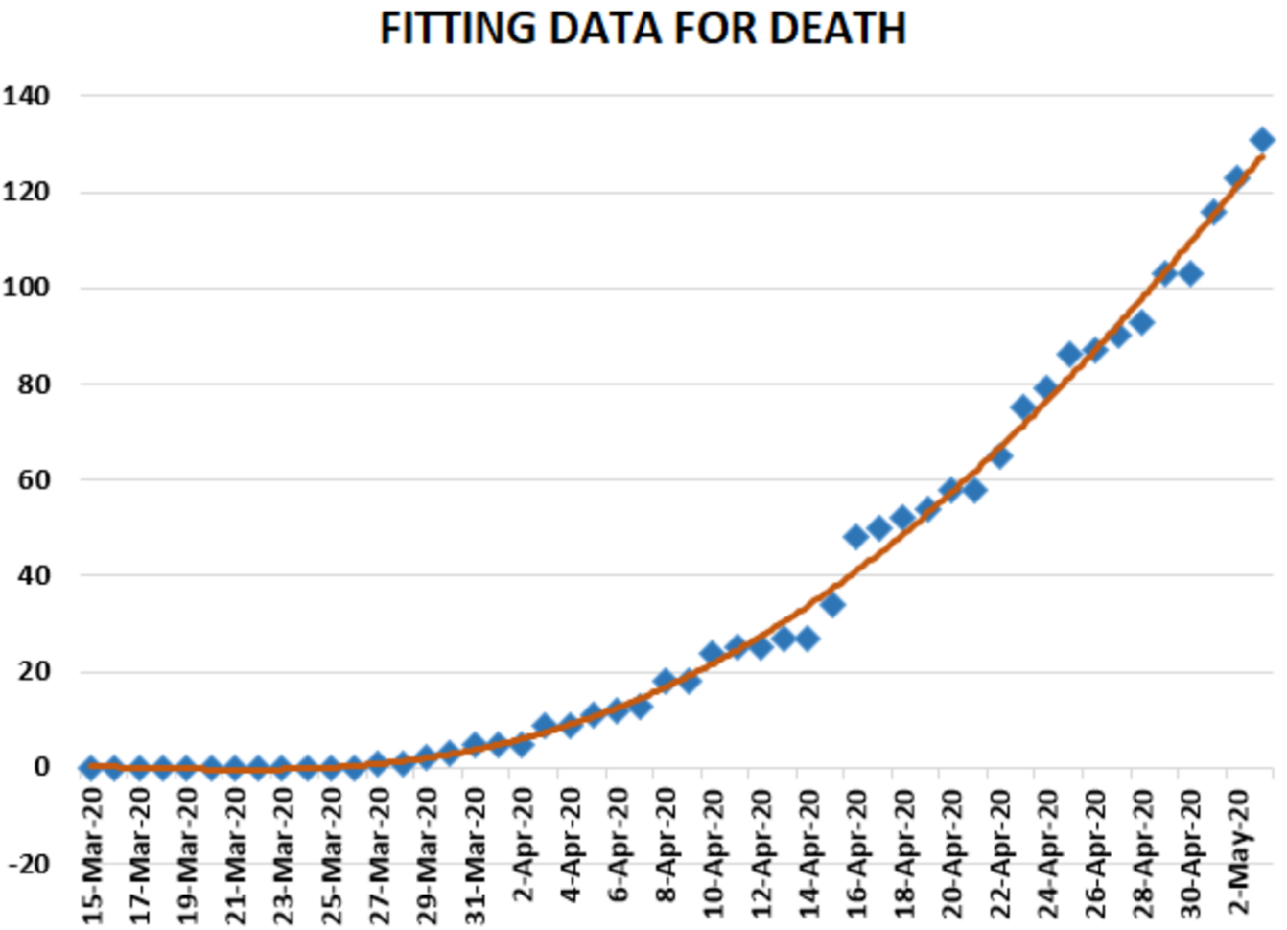
Polynomial fitting data dead people from Covid-19 in South Africa from 15 March 2020 to 3 May 2020

**Figure 29:**
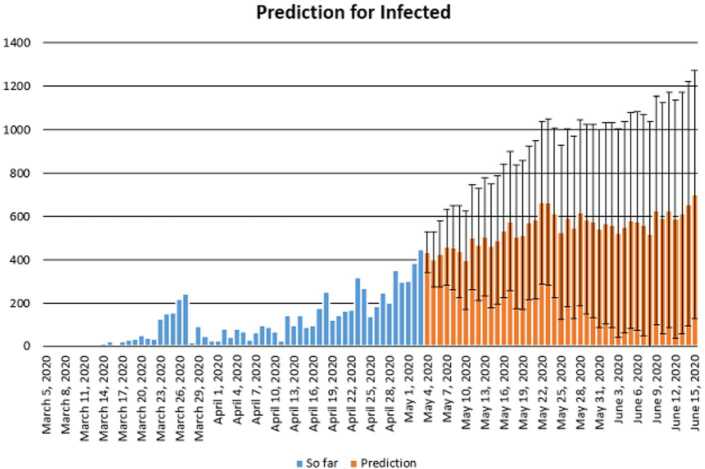
Prediction of daily number of infected in South Africa using forecast sheet with reliability level of 90%

**Figure 30:**
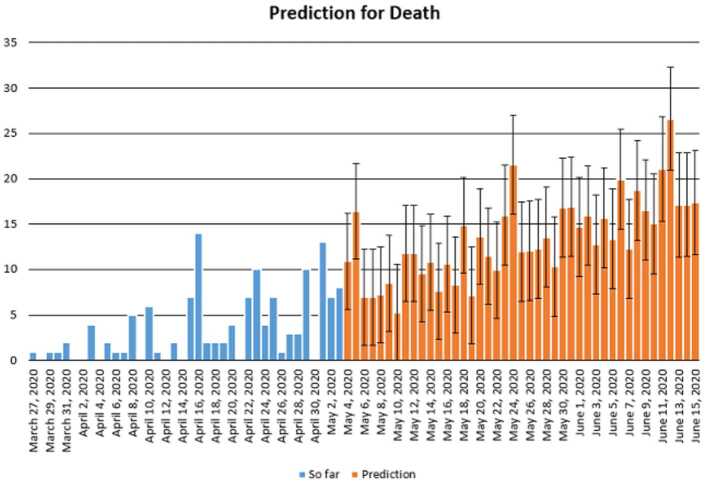
Prediction of daily number of dead in South Africa using forecast sheet with reliability level of 90%

In this section, we aim to provide a detailed statistical analysis of the collected data representing the evolution of Covid-19 spread within the Republic of South Africa. These data include the daily numbers of new infected and dead. The collected data are from 5 March 2020 to 3 May 2020 [13]. The main aim of this section is to predict what could possibly happen in the near future using the reliability level method, additionally, to find which distribution each class follows. With the collected data, we will first present a histogram, pie chart, and nonlinear graphs for each class. The histograms will help identify the density of probability associated to each set of collected data. Additionally, we provide a polynomial fitting against collected data. The results are presented in Figs. 21 to 30. For each case, we present arithmetic, geometric, and harmonic means, respectively, as well as skewness, variance, covariance, Pearson correlation and Spearman correlation, and these results are presented in Table 2.

In Figs. 21, 22, and 23, we present some statistical simulation of the number of infected people due to Covid-19 in South Africa from 5 March 2020 to 3 May 2020.

In Figs. 24, 25, and 26, we present some statistical simulation of the number of dead people due to Covid-19 in South Africa from 15 March 2020 to 3 May 2020.

Now we present regression analysis of Covid-19 data in South Africa from 5 March 2020 to 3 May 2020. We first present a predictive analysis for infected people. According to the results obtained, we get a linear regression which can be calculated as

2.19 $$ y=-4488415+102.2293x. $$

The *F*-test statistics was calculated to be $4.84\times 10^{-31}$, while $R^{2}$ was calculated as 0.902781. We can say that the significance of fit for model is suitable for the considered data. We can give another regression model

2.20 $$\begin{aligned} y={}&{-}0.4\times 10^{-6}x^{6}+0.9253x^{5}-101611x^{4}+0.6 \times 10^{-9}x^{3}-0.2 \times 10^{-14}x^{2} \\ &{}+0.3 \times 10^{-18}x-0.3\times 10^{-22}, \end{aligned}$$

which is polynomial of sixth order. For this polynomial, $R^{2}$ was calculated as 0.9978. We present a simulation of the polynomial fitting for infected people from 5 March 2020 to 3 May 2020.

Now we present a predictive analysis for dead people. According to the results obtained, we get a linear regression which can be calculated as

2.21 $$ y=-29.2547+2.51587x. $$

The *F*-test statistic was calculated to be $5.36\times 10^{-22}$, while $R^{2}$ was calculated as 0.858225. We can say that the significance of fit for this model is high enough for the considered data. We can suggest another regression model

2.22 $$ y=-0.2\times 10^{-5}x^{4}+3.7609x^{3}-247847x^{2}+0.7 \times 10^{-9}x-0.8 \times 10^{-13}, $$

which is polynomial of fourth order. For this polynomial, $R^{2}$ was calculated as 0.9958. We present a simulation of polynomial fitting for dead people from 15 March 2020 to 3 May 2020.

#### Prediction for coronavirus data in South Africa

In this section, we aim at performing prediction using existing collected data representing daily numbers of new infected, dead, and reliability level method. The collected data will be considered from 5 March 2020, corresponding to the first day of confirmed case of Covid-19 in South Africa, to 3 May 2020. The future prediction will start from 3 May 2020 to 15 June 2020. This will help us give a prediction on the numbers of new daily infected, recovered, and dead in South Africa within this period. The prediction will consist of three different graphs comprising upper boundaries, middle lines, and low boundaries. The upper boundaries represent the worse case scenario, of course, a scenario that is not needed for the classes of dead and infected, but an ideal one for the recovered class, and the lower boundaries representing the perfect scenario (a scenario that is needed) for South Africa to get rid of the infection. These results of prediction for future daily new infected, recovered and dead are represented graphically in Figs. 29 and 30, respectively. The prediction of daily new infected in the case of South Africa seems to follow the upper boundaries and low boundary for daily number of dead.

We now give some statistics for the coronavirus data in South Africa in Table 3.

**Table 3:** Some data about coronavirus cases in South Africa

	Infected	Dead
Arithmetic mean	113.05000	2.62000
Geometric mean	57.35444	3.184267
Harmonic mean	11.569120	2.260659
Standard deviation	109.71834	3.613410
Skewness	1.1222456	1.594677
Variance	11837.4808	12.79560

**Figure 31:**
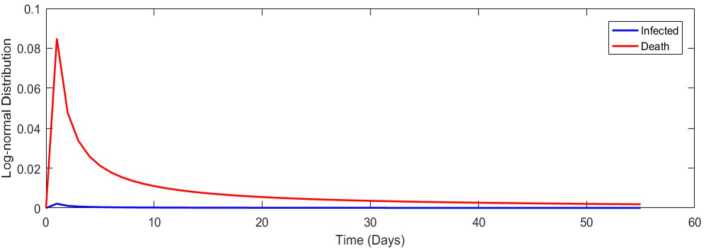
Lognormal distribution for all cases in South Africa

Table 4 presents the covariance, Pearson and Spearman correlation coefficients between daily cases–recovered, recovered–dead, and infected–dead for Covid-19 data in South Africa. We present a lognormal distribution for infected and dead for Covid-19 data in Fig. 31.

**Table 4:** Some data for coronavirus cases in South Africa

	Infected–Dead
Covariance	209.4908333
Pearson Correlation	0.564936726
Spearman Correlation	0.607349264

### Parameter estimation using the bell curve approach

In the previous section, we presented the graph of a day-to-day evolution of Covid-19 spread including infected, recovered, and dead for South Africa and Turkey. To be honest, one cannot for sure tell if those curves follow the normal or lognormal distribution. Therefore in this section, two cases are considered. In the first case, we assume a lognormal curve and then we assume the normal distribution curve.

*Case* I. We consider the lognormal density of probability

2.23 $$ L_{p} ( x ) =\frac{1}{x}\frac{1}{\sigma \sqrt{2\pi }}\exp \biggl[ -\frac{1}{2}\frac{ ( \ln x-\mu ) ^{2}}{\sigma ^{2}} \biggr] . $$

We now define a function *β* that captures daily occurrences

2.24 $$ \beta =O_{0}\exp \biggl[ -\frac{1}{2} \frac{ ( \ln x-\mu ) ^{2}}{\sigma ^{2}} \biggr]. $$

We aim to estimate $O_{0},\sigma $, and *μ*. To achieve this, we consider the first four different days

2.25 $$ d_{1}< d_{2}< d_{3}< d_{4}\quad\text{where }d_{i}=\ln x_{i}. $$

We first start by estimating *μ*, by assuming a proportion

2.26 $$ \frac{\beta ( d_{2} ) }{\beta ( d_{1} ) }= \exp \biggl[ -\frac{1}{2\sigma ^{2}} \bigl\{ ( d_{2}-\mu ) ^{2}- ( d_{1}-\mu ) ^{2} \bigr\} \biggr] $$

and

2.27 $$ \frac{\beta ( d_{4} ) }{\beta ( d_{3} ) }= \exp \biggl[ -\frac{1}{2\sigma ^{2}} \bigl\{ ( d_{4}-\mu ) ^{2}- ( d_{3}-\mu ) ^{2} \bigr\} \biggr]. $$

To proceed, we apply on both sides the ln function

2.28 $$\begin{aligned} \begin{aligned} &\ln \biggl[ \frac{\beta ( d_{2} ) }{\beta ( d_{1} ) } \biggr] = -\frac{1}{2\sigma ^{2}} \bigl\{ ( d_{2}-\mu ) ^{2}- ( d_{1}-\mu ) ^{2} \bigr\} \\ &\quad\Rightarrow\quad \sigma ^{2}=- \frac{1}{2\ln [ \frac{\beta ( d_{2} ) }{\beta ( d_{1} ) } ] } \bigl\{ ( d_{2}-\mu ) ^{2}- ( d_{1}-\mu ) ^{2} \bigr\} , \\ &\ln \biggl[ \frac{\beta ( d_{4} ) }{\beta ( d_{3} ) } \biggr] = -\frac{1}{2\sigma ^{2}} \bigl\{ ( d_{4}-\mu ) ^{2}- ( d_{3}-\mu ) ^{2} \bigr\} \\ &\quad \Rightarrow \quad\sigma ^{2}=- \frac{1}{2\ln [ \frac{\beta ( d_{4} ) }{\beta ( d_{3} ) } ] } \bigl\{ ( d_{4}-\mu ) ^{2}- ( d_{3}-\mu ) ^{2} \bigr\} . \end{aligned} \end{aligned}$$

Due to the equality, we can now have

2.29 $$ \frac{1}{\ln [ \frac{\beta ( d_{2} ) }{\beta ( d_{1} ) } ] } \bigl\{ ( d_{2}-\mu ) ^{2}- ( d_{1}-\mu ) ^{2} \bigr\} = \frac{1}{\ln [ \frac{\beta ( d_{4} ) }{\beta ( d_{3} ) } ] } \bigl\{ ( d_{4}-\mu ) ^{2}- ( d_{3}-\mu ) ^{2} \bigr\} . $$

Thus

2.30 $$ \frac{\ln [ \frac{\beta ( d_{4} ) }{\beta ( d_{3} ) } ] }{\ln [ \frac{\beta ( d_{2} ) }{\beta ( d_{1} ) } ] }= \frac{ ( d_{4}+d_{3}-2\mu ) - ( d_{4}-d_{3} ) }{ ( d_{2}+d_{1}-2\mu ) - ( d_{2}-d_{1} ) }. $$

The solution for the above is

2.31 $$ \mu = \frac{\frac{1}{2} ( d_{4}+d_{3} ) ( d_{4}-d_{3} ) -\frac{\ln [ \frac{\beta ( d_{4} ) }{\beta ( d_{3} ) } ] }{\ln [ \frac{\beta ( d_{2} ) }{\beta ( d_{1} ) } ] } ( d_{2}+d_{1} ) ( d_{2}-d_{1} ) }{\frac{1}{2} ( d_{4}-d_{3} ) -\frac{\ln [ \frac{\beta ( d_{4} ) }{\beta ( d_{3} ) } ] }{\ln [ \frac{\beta ( d_{2} ) }{\beta ( d_{1} ) } ] } ( d_{2}-d_{1} ) }. $$

Having *μ*, we can determine

2.32 $$ \sigma =\sqrt{ \frac{1}{2\ln [ \frac{\beta ( d_{2} ) }{\beta ( d_{1} ) } ] } \bigl\{ - ( d_{2}-\mu ) ^{2}+ ( d_{1}-\mu ) ^{2} \bigr\} }. $$

Alternatively, we consider 8 days to capture more facts $x_{i}$, $i=1,2,3,4,5,6,7,8$, where we put $d_{i}=\ln x_{i}$. We assume a proportionality *λ* of $\{ d_{1},d_{2},d_{3},d_{4} \} $ and $\{ d_{5},d_{6},d_{7},d_{8} \} $. Therefore

2.33 $$ P_{1}= \frac{ ( d_{4}+d_{3}-2\mu ) - ( d_{4}-d_{3} ) }{ ( d_{2}+d_{1}-2\mu ) - ( d_{2}-d_{1} ) } $$

and

2.34 $$ P_{2}= \frac{ ( d_{8}+d_{7}-2\mu ) - ( d_{8}-d_{7} ) }{ ( d_{6}+d_{5}-2\mu ) - ( d_{6}-d_{5} ) }. $$

We now assume that $P_{1}$ is proportional to $P_{2}$, thus

2.35 $$ \frac{ ( d_{4}+d_{3}-2\mu ) - ( d_{4}-d_{3} ) }{ ( d_{2}+d_{1}-2\mu ) - ( d_{2}-d_{1} ) }=\lambda \frac{ ( d_{8}+d_{7}-2\mu ) - ( d_{8}-d_{7} ) }{ ( d_{6}+d_{5}-2\mu ) - ( d_{6}-d_{5} ) }. $$

For simplicity, we put $d_{i}+d_{j}=A_{ij}=A_{ji}$ and then

2.36 $$ d_{14}=\frac{d_{4}-d_{3}}{d_{2}-d_{1}},\qquad d_{58}= \frac{d_{8}-d_{7}}{d_{6}-d_{5}}. $$

Therefore, the above can be reformulated as

2.37 $$ \frac{A_{43}-2\mu }{A_{21}-2\mu }d_{14}=\lambda \frac{A_{87}-2\mu }{A_{65}-2\mu }d_{58}. $$

Also we write

2.38 $$ \bigl( A_{43}A_{65}-2\mu ( A_{43}+A_{65} ) +4\mu ^{2} \bigr) \frac{d_{14}}{\lambda d_{58}}= \bigl( A_{21}A_{87}-2 \mu ( A_{21}+A_{87} ) +4\mu ^{2} \bigr) $$

and

2.39 $$ 4\mu ^{2} \biggl\{ \frac{d_{14}}{\lambda d_{58}}-1 \biggr\} -2\mu \biggl\{ \frac{d_{14}}{\lambda d_{58}} ( A_{43}+A_{65} ) + ( A_{21}+A_{87} ) \biggr\} +A_{43}A_{65} \frac{d_{14}}{\lambda d_{58}}-A_{21}A_{87}=0. $$

Thus we have

2.40 $$ \mu _{1,2}= \frac{ ( \frac{d_{14}}{\lambda d_{58}} ( A_{43}+A_{65} ) + ( A_{21}+A_{87} ) ) \pm \sqrt{\textstyle\begin{array}{c} ( \frac{d_{14}}{\lambda d_{58}} ( A_{43}+A_{65} ) + ( A_{21}+A_{87} ) ) ^{2} \\ -4 \{ \frac{d_{14}}{\lambda d_{58}}-1 \} ( A_{43}A_{65}\frac{d_{14}}{\lambda d_{58}}-A_{21}A_{87} )\end{array}\displaystyle }}{4 \{ \frac{d_{14}}{\lambda d_{58}}-1 \} }. $$

Thus for Case I, we get

2.41 $$ O_{0}= \frac{\sum_{j=1}^{4}\beta ( \ln x_{i} ) }{\sum_{j=1}^{4}\frac{1}{x_{j}}\exp [ -\frac{1}{2}\frac{ ( \ln x_{j}-\mu ) ^{2}}{\sigma ^{2}} ] }. $$

In the second case, we get

2.42 $$\begin{aligned} \begin{aligned} &O_{0} = \frac{\sum_{j=1}^{8}\beta ( \ln x_{j} ) }{\sum_{j=1}^{8}\frac{1}{x_{j}}\exp [ -\frac{1}{2}\frac{ ( \ln x_{j}-\mu ) ^{2}}{\sigma ^{2}} ] } , \\ &\lambda = \frac{\ln [ \frac{\beta ( d_{4} ) }{\beta ( d_{3} ) } ] }{\ln [ \frac{\beta ( d_{2} ) }{\beta ( d_{1} ) } ] } \times \frac{\ln [ \frac{\beta ( d_{6} ) }{\beta ( d_{5} ) } ] }{\ln [ \frac{\beta ( d_{8} ) }{\beta ( d_{7} ) } ] }. \end{aligned} \end{aligned}$$

For each case, the cumulative distribution function can be calculated by

2.43 $$ \Phi ( x ) =\frac{1}{2} \biggl[ 1+\operatorname{erf} \biggl( \frac{ ( \ln x_{j}-\mu ) }{\sigma \sqrt{2}} \biggr) \biggr]. $$

*Case* II. We assume that the curve follows the normal distribution, thus

2.44 $$ \Phi ( x ) =\frac{1}{\sigma \sqrt{2\pi }}\exp \biggl[ - \frac{1}{2} \biggl( \frac{x-\mu }{\sigma } \biggr) ^{2} \biggr]. $$

However, we consider the following function:

2.45 $$ \lambda ( x ) =\lambda _{0}\exp \biggl[ -\frac{1}{2} \biggl( \frac{x-\mu }{\sigma } \biggr) ^{2} \biggr]. $$

We aim to determine $\lambda _{0},\sigma $, and *μ*. Here we choose three points $d_{1},d_{2},d_{3}$ such that $\lambda ( d_{2} ) $ corresponds to the maximum point. Following the procedure presented earlier, we have

2.46 $$ \frac{\ln [ \frac{\lambda ( d_{3} ) }{\lambda ( d_{2} ) } ] }{\ln [ \frac{\lambda ( d_{2} ) }{\lambda ( d_{1} ) } ] }= \frac{ ( d_{3}+d_{2}-2\mu ) - ( d_{3}-d_{2} ) }{ ( d_{2}+d_{1}-2\mu ) - ( d_{2}-d_{1} ) }. $$

Thus

2.47 $$ \mu = \frac{\frac{1}{2} ( d_{3}+d_{2} ) ( d_{3}-d_{2} ) -\frac{\ln [ \frac{\lambda ( d_{3} ) }{\lambda ( d_{2} ) } ] }{\ln [ \frac{\lambda ( d_{2} ) }{\lambda ( d_{1} ) } ] } ( d_{2}+d_{1} ) ( d_{2}-d_{1} ) }{\frac{1}{2} ( d_{3}+d_{2} ) -\frac{\ln [ \frac{\lambda ( d_{3} ) }{\lambda ( d_{2} ) } ] }{\ln [ \frac{\lambda ( d_{2} ) }{\lambda ( d_{1} ) } ] } ( d_{2}-d_{1} ) }. $$

With *μ* in hand, we determine

2.48 $$ \sigma =\sqrt{ \frac{1}{2\ln [ \frac{\lambda ( d_{2} ) }{\lambda ( d_{1} ) } ] } \bigl\{ ( d_{1}-\mu ) ^{2}- ( d_{2}-\mu ) ^{2} \bigr\} } $$

and

2.49 $$ \lambda _{0}= \frac{\sum_{j=1}^{3}\lambda ( d_{j} ) }{\sum_{j=1}^{3}\exp [ -\frac{1}{2}\frac{ ( d_{j}-\mu ) ^{2}}{\sigma ^{2}} ] }. $$

In particular, if we consider the case where $d_{2}-d_{1}=d_{3}-d_{2}$, that is, $\lambda ( d_{1} ) =\lambda ( d_{3} ) $ due to symmetry of normal distribution, then we get

2.50 $$ \mu = \frac{\frac{1}{2} ( d_{3}+d_{2} ) ( d_{3}-d_{2} ) + ( d_{2}+d_{1} ) ( d_{2}-d_{1} ) }{\frac{1}{2} ( d_{3}+d_{2} ) + ( d_{2}-d_{1} ) }. $$

### Comparison: Turkey vs South Africa

In this subsection, we present a comparison between Turkey and South Africa regarding Covid-19 as edicated in Table 5.

**Table 5:**
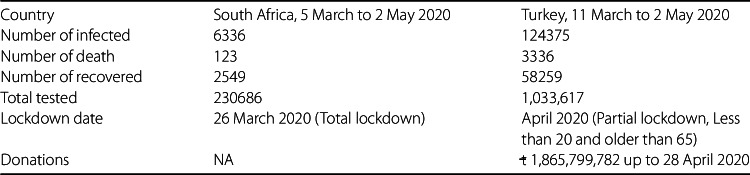
Comparison between Turkey and South Africa updated on Covid-19

The analysis presented in this section does not aim at praising or criticizing any country, it is just to assess the effect of lockdown and its regulations, and to perceive if this concept can help save humans before the vaccine. The fundamental question to answer here is why South Africa has fewer dead and infected people than Turkey, if it recorded its first confirmed case six days earlier before Turkey. The answer may rely on two fundamental facts which include the period lockdown was implemented and the type of lockdown put in place. The South African government publicized on 23 March 2020 a 21-day of national lockdown which started effectively from midnight 27 March 2020. This was announced 22 days after the first confirmed case was recorded in the country. The lockdown came with strict measures encompassing immediate deployment of South African National Defence force to ensure that all people living within the territory of South Africa obey the lockdown rules. Only workers considered necessary to operative response to the pandemic were exempted, namely health caregivers, security service providers, essential service providers that are fundamental to the rudimentary functioning of economy, as well as other workers in industries that cannot be economically shut down. This implies that the mentioned categories were permitted to go to their places of work during the lockdown. On the other hand, the number of people at gatherings apart from funerals was limited to 50 people; while restaurants, taverns, bottle stores, and shops that are not selling indispensable goods were forced to close. Thus, a large population was not allowed to leave their houses except for essential needs. Consequently, the movement between provinces, metropolitan, and districts was also restricted except for essential reasons that cannot be catered for within provincial boundaries. The South African government further closed all of its national borders and only allowed the transportation for indispensable reasons. Likewise, all international and domestics passenger flights were prohibited, except those assigned to evacuate citizens from foreign countries and certain repatriations due to Covid-19. However, the measures taken by Turkey were not implemented swiftly upon the confirmation of its first Covid-19 case. It is recorded that the Turkish government announced a partial lockdown on 11 April 2020, a month after the country registered it first confirmed case of Covid-19. Prior to the announcement of the partial lockdown, mosques, cafes, night clubs, and all universities within the country were already closed on 11 March 2020. The restriction measures applied only to people younger than 20 year old and older than 65 year old, who were not allowed to leave their homes except for indispensable reasons. In addition, the government ordered a ban on movement between 30 major cities with metropolitan status as well as Zonguldak; whereby the lockdown was applied every weekend from 11 April 2020 and also 21–23 April 2020. Punishment (money) was applied for people who went out. Very importantly the government has totally banned the sale of masks, but provided free masks to its people to be compulsorily utilized in public places. However, the newly placed order exempted health care assistance, funerals, military and passenger transport from the ban, provided that certain conditions were met. Although both countries have put severe measures to protect their citizens from the deadly disease, there are still records of the rising number of infected and dead people in both Turkey and South Africa. Does that mean that the lockdown regulations are worthless? Absolutely not! It is only that several citizens in respective countries were not adhering to the rules and regulations put in place by their respective government authorities. This results from the concept of social distancing being largely misunderstood, as it is not clearly defined to mean whether persons should stay one meter away from one another or only from any infected humans, contaminated air, and other objects because of the nature in which Covid-19 can be spread. Nevertheless, due to a long incubation period of Covid-19, approximately 14 days maximum, which renders ordinary citizens not to differentiate an infected person from others, then it is crucial that stringent measures be implemented, which will prohibit people from leaving their homes, and in case they go out, they should maintain the one meter distance away from each other and frequently wash their hands upon touching any object.

## Mathematical model of Covid-19 in South Africa and Turkey

Mathematical models of infected diseases are deemed not that useful by some people who feel that they cannot be utilized to develop a vaccine or cure any given disease. However, it is important to note that the principal aim of these mathematical models is to describe a system using mathematical tools, concepts, and language. Hence, throughout the history of human beings, researchers working within the field of mathematics have developed more accurate and efficient mathematical models. For instance, history has made reference to one of the well-known Newtonian laws which described very accurately many problems in our daily lives, although they are coupled with some limits. In instances where these laws failed, two other well-known concepts, namely the theory of relativity and quantum mechanics, using mathematical formulas can be utilized instead. Generally, these concepts are of great importance in all fields of science such as in natural sciences including chemistry, biology, physics, and earth science, in engineering such as computer science, and electrical engineering, as well as in social science where their applicability to economics, sociology, psychology, and political science can be relevant. In other words, mathematical models can help provide a clear explanation of a system and investigate the effect of several components, and later make accurate predictions based on the observed facts. In the current situation under study, due to the magnitude of fear imposed by Covid-19 on humans, it is therefore paramount for mathematicians to provide conceptual models, using mathematical tools called differential and integral operators, to suggest well-constructed mathematical models that will be used to understand and predict the spread of Covid-19.

In this section, a mathematical model that takes into account nine classes (susceptible, infected which has 5 subclasses, recovered, dead, and vaccinated classes), the dynamic is presented and explained with the subsequent diagrams, but the class of dead is omitted because it can produce a complex model. The created model incorporates the lockdown effect, represented by a coefficient that takes into account the social distancing and a contact coefficient:

3.1 $$\begin{aligned} &\overset{\cdot }{S} = \Lambda - \bigl( \alpha ( x ) +\gamma _{1}+\mu _{1} \bigr) S, \\ &\overset{\cdot }{I}= \alpha ( x ) S- ( \varepsilon +\xi +\lambda +\mu _{1} ) I, \\ &\overset{\cdot }{I_{A}}= \xi I- ( \theta +\mu +\chi + \mu _{1} ) I_{A}, \\ &\overset{\cdot }{I_{D}} = \varepsilon I- ( \eta + \varphi + \mu _{1} ) I_{D}, \\ &\overset{\cdot }{I_{R}} = \eta I_{D}+\theta I_{A}- ( v+ \xi +\mu _{1} ) I_{R}, \\ &\overset{\cdot }{I_{T}}= \mu I_{A}+vI_{R}- ( \sigma + \tau +\mu _{1} ) I_{T}, \\ &\overset{\cdot }{R}= \lambda I+\varphi I_{D}+\chi I_{A}+ \xi I_{R}+\sigma I_{T}- ( \Phi +\mu _{1} ) R, \\ &\overset{\cdot }{D}= \tau I_{T}, \\ &\overset{\cdot }{V}= \gamma _{1}S+\Phi R-\mu _{1}V, \end{aligned}$$

where

3.2 $$\begin{aligned} \begin{aligned} &\overset{\cdot }{N}= \Lambda -\mu _{1}N, \\ &\alpha ( x ) = \frac{k_{1}pe^{-x}}{N} \bigl( I+w ( \beta I_{D}+\gamma I_{A}+\delta _{1}I_{R} ) \bigr). \end{aligned} \end{aligned}$$

Here $S ( t ) $ is the class of individuals that are susceptible to contact Covid-19 at time *t*; $I ( t ) $ is the class of individuals that are susceptible to contacted Covid-19, but have no symptoms and have not been tested; $I_{A} ( t ) $ is the class of individuals that have some symptoms but were not tested yet; $I_{D} ( t ) $ is the class of individuals that have contacted Covid-19, have been tested positive, but show no symptoms; $I_{R} ( t ) $ is the class of individuals that have contacted Covid-19, have been tested positive, and have symptoms; $I_{T} ( t ) $ is the class of individuals that have contacted Covid-19 and are in critical condition; $R ( t ) $ is the class of recovered individuals at time *t*; $D ( t ) $ is the number of dead at time *t*; $V ( t ) $ is the class of individuals that have been vaccinated, Table 6.

**Table 6:** Parameters of the suggested Covid-19 model

Φ: The rate at which recovered and were vaccinated
$\mu _{1}$: Turkish natural mortality rate
*α*(*x*): The infection force
*β*: Transmission rate of $I_{D} ( t )$ class
*γ*: Transmission rate of $I_{A} ( t )$ class
$\delta _{1}$: Transmission rate of $I_{R} ( t )$ class
*ε*: Proportion rate of detection relative to asymptomatic class
*θ*: Proportion rate of detection relative to symptomatic class
*ξ*: Rate that infected are not aware of their status
*η*: Rate that infected are aware of their status
*μ*: Rate that nontested join class $I_{T} ( t )$
*v*: Rate that tested join class $I_{T} ( t )$
*τ*: The mortality rate due to Covid-19
*λ*: Recovery rate of class *I*(*t*)
*κ*: Recovery rate of class $I_{A} ( t )$
*ξ*: Recovery rate of class $I_{R} ( t )$
*φ*: Recovery rate of class $I_{D} ( t )$
*σ*: Recovery rate of class $I_{T} ( t )$
$k_{1}$: Contact rate
*p*: Proportion that a contact is sufficient enough to lead to transmission
*w*: Transmission coefficient for the infected classes
Λ: Recruitment rate into class *S*(*t*)
$\gamma _{1}$: Rate of vaccination

The initial conditions are given as:

3.3 $$\begin{aligned} \begin{aligned} &N ( 0 ) = N_{0},\qquad S ( 0 ) =S_{0},\qquad I ( 0 ) =I_{0},\qquad I_{A} ( 0 ) =I_{A}^{0},\qquad I_{D} ( 0 ) =I_{D}^{0}, \\ &I_{R} ( 0 ) = I_{R}^{0},\qquad I_{T} ( 0 ) =I_{T}^{0},\qquad R ( 0 ) =R_{0},\qquad D ( 0 ) =D_{0},\qquad V ( 0 ) =V_{0}. \end{aligned} \end{aligned}$$

**Figure 32:**
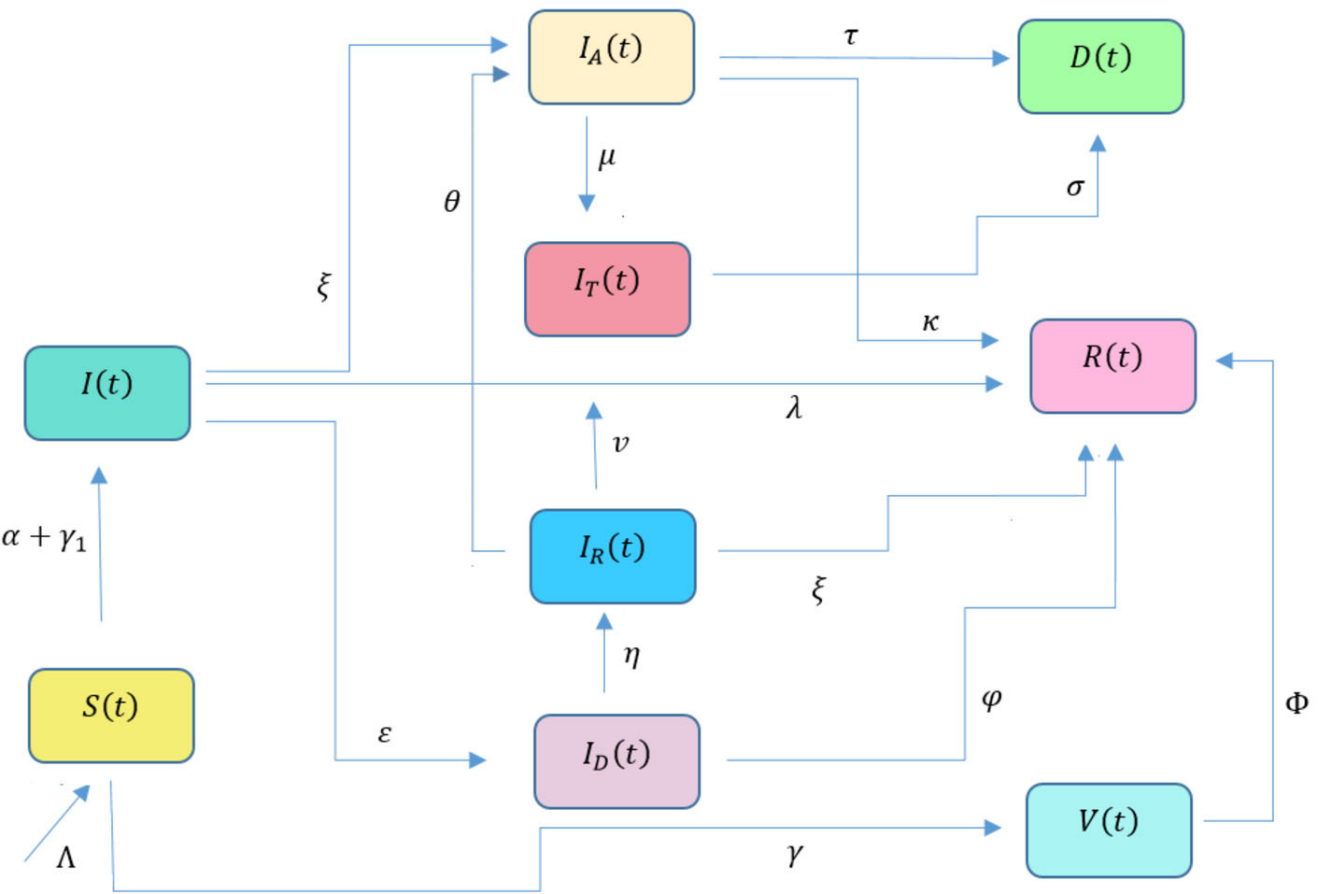
Diagram summarizing Covid-19 model given by the suggested system

We present a diagram which summarizes Covid-19 model which is described by the system (3.1) in Fig. 21. The diagram summarizing Covid-19 spread model is presented in Fig. 32.

### Boundedness and positivity of the solutions

In this section, we show that $\forall t\geq 0$, the system solution is positive, so that the model is well-posed and biologically feasible. We define the norm

3.4 $$ \Vert f \Vert =\sup_{t\in D_{f}} \bigl\vert f ( t ) \bigr\vert . $$

We assume that all the class

3.5 $$ S ( \alpha +\gamma _{1} ) >0, \quad\forall t\geq 0 $$

due to the model under this assumption. We write

3.6 $$\begin{aligned} \overset{\cdot }{I} ( t ) & = \alpha S- ( \varepsilon +\xi +\lambda +\mu _{1} ) I \\ &\geq - ( \varepsilon +\xi +\lambda +\mu _{1} ) I ( t ) \\ &\geq I^{0}e^{- ( \varepsilon +\xi +\lambda +\mu _{1} ) t}, \quad\forall t\geq 0. \end{aligned}$$

Since $I ( t ) \geq 0,\forall t\geq 0$, then

3.7 $$\begin{aligned} \overset{\cdot }{I_{A}} ( t ) & = \xi I ( t ) - ( \theta + \mu +\chi +\mu _{1} ) I_{A} ( t ) \\ &\geq - ( \theta +\mu +\chi +\mu _{1} ) I_{A} ( t ),\quad \forall t\geq 0, \end{aligned}$$

thus

3.8 $$ I_{A} ( t ) \geq I_{A}^{0}e^{- ( \theta +\mu + \chi +\mu _{1} ) t},\quad \forall t\geq 0. $$

The same holds for the $I_{D} ( t ) $ class:

3.9 $$ I_{D} ( t ) \geq I_{D}^{0}e^{- ( \eta + \varphi +\mu _{1} ) t},\quad \forall t\geq 0. $$

Also $I_{A} ( t ) $ and $I_{D} ( t ) $ are positive $\forall t\geq 0$ and $\eta,\theta \geq 0$ and then

3.10 $$\begin{aligned} \begin{aligned} &I_{R} ( t ) \geq I_{R}^{0}e^{- ( v+\xi + \mu _{1} ) t}, \\ &I_{T} ( t ) \geq I_{T}^{0}e^{- ( \sigma + \tau +\mu _{1} ) t}, \\ &R ( t ) \geq R_{0}e^{- ( \Phi +\mu _{1} ) t},\\ &D ( t ) \geq D_{0}, \quad \forall t\geq 0. \end{aligned} \end{aligned}$$

Also

3.11 $$\begin{aligned} \begin{aligned} &\overset{\cdot }{V} ( t ) \geq -\mu _{1}V ( t ), \\ &V ( t ) \geq V_{0}e^{-\mu _{1}t}, \quad \forall t \geq 0. \end{aligned} \end{aligned}$$

With $S ( t ) $, we have to assume that

3.12 $$ \Vert \alpha \Vert _{\infty }< \infty\quad \Rightarrow\quad \Vert I \Vert _{\infty }+w \Vert I_{D} \Vert _{ \infty }+\gamma \Vert I_{A} \Vert _{\infty }+\delta _{1} \Vert I_{R} \Vert _{\infty }< \infty $$

so that

3.13 $$\begin{aligned} \overset{\cdot }{S} ( t ) & = \Lambda - \bigl( \alpha ( x ) +\gamma _{1}+\mu _{1} \bigr) S \\ &\geq - \bigl( \alpha ( x ) +\gamma _{1}+\mu _{1} \bigr) S \\ &\geq - \bigl( \bigl\vert \alpha ( x ) \bigr\vert + \gamma _{1}+\mu _{1} \bigr) S \\ &\geq - \Bigl( \sup_{x\in D_{\alpha }} \bigl\vert \alpha ( x ) \bigr\vert +\gamma _{1}+\mu _{1} \Bigr) S \\ &\geq - \bigl( \Vert \alpha \Vert _{\infty }+\gamma _{1}+ \mu _{1} \bigr) S,\quad \forall t\geq 0. \end{aligned}$$

This implies that

3.14 $$ S ( t ) \geq S_{0}e^{- ( \Vert \alpha \Vert _{\infty }+\gamma _{1}+\mu _{1} ) t}, \quad\forall t \geq 0. $$

Now in the absence of the Covid-19, we have

3.15 $$ N ( t ) \leq \frac{\Lambda }{\mu _{1}}. $$

The above inequality is called the threshold population level. This is obtained because we assume that the total population size must be increased or be constant

3.16 $$ \frac{dN ( t ) }{dt}\geq 0\quad\Rightarrow\quad \Lambda -\mu _{1}N \geq 0, $$

therefore $N ( t ) \leq \frac{\Lambda }{\mu _{1}}$. It is therefore biologically feasible that

3.17 $$\begin{aligned} \Omega ={}& \biggl\{ ( S,I,I_{A},I_{D},I_{R},I_{T},R,D,V ) \in \mathbb{R}: \\ &0\leq S+I+I_{A}+I_{D}+I_{R}+I_{T}+R+D+V=N \leq \frac{\Lambda }{\mu _{1}} \biggr\} . \end{aligned}$$

The disease-free equilibrium point is

3.18 $$ \biggl( \frac{\Lambda }{\gamma _{1}+\mu _{1}},0,0,0,0,0,0,0, \frac{\Lambda \gamma _{1}}{\mu _{1} ( \gamma _{1}+\mu _{1} ) } \biggr). $$

We now derive the reproduction number using the next generation operator technique [14]. We have five infected classes $I ( t ),I_{A} ( t ),I_{D} ( t ),I_{R} ( t ) $, and $I_{T} ( t ) $. The matrices *F* and *V* will be obtained from

3.19 $$\begin{aligned} \begin{aligned} &\overset{\cdot }{I}= \alpha ( x ) S- ( \varepsilon +\xi +\lambda +\mu _{1} ) I, \\ &\overset{\cdot }{I_{A}}= \xi I- ( \theta +\mu +\chi + \mu _{1} ) I_{A}, \\ &\overset{\cdot }{I_{D}} = \varepsilon I- ( \eta + \varphi + \mu _{1} ) I_{D}, \\ &\overset{\cdot }{I_{R}} = \eta I_{D}+\theta I_{A}- ( v+ \xi +\mu _{1} ) I_{R}, \\ &\overset{\cdot }{I_{T}}= \mu I_{A}+vI_{R}- ( \sigma + \tau +\mu _{1} ) I_{T}. \end{aligned} \end{aligned}$$

We obtain the following matrices:

3.20 $$ F= \begin{bmatrix} \delta ( x ) & \gamma \delta ( x ) w & \beta \delta ( x ) w & \delta ( x ) w\delta _{1} & 0 \\ 0 & 0 & 0 & 0 & 0 \\ 0 & 0 & 0 & 0 & 0 \\ 0 & 0 & 0 & 0 & 0 \\ 0 & 0 & 0 & 0 & 0\end{bmatrix} $$

and

3.21 $$\begin{aligned} V= \begin{bmatrix} ( \varepsilon +\xi +\lambda +\mu _{1} ) & 0 & 0 & 0 & 0 \\ -\xi & ( \theta +\mu +\chi +\mu _{1} ) & 0 & 0 & 0 \\ -\varepsilon & 0 & ( \eta +\varphi +\mu _{1} ) & 0 & 0 \\ 0 & -\theta & -\eta & ( v+\xi +\mu _{1} ) & 0 \\ 0 & -\mu & 0 & -v & ( \sigma +\tau +\mu _{1} )\end{bmatrix}. \end{aligned}$$

For simplicity, write

3.22 $$ V= \begin{bmatrix} l_{1} & 0 & 0 & 0 & 0 \\ -\xi & l_{2} & 0 & 0 & 0 \\ -\varepsilon & 0 & l_{3} & 0 & 0 \\ 0 & -\theta & -\eta & l_{4} & 0 \\ 0 & -\mu & 0 & -v & l_{5}\end{bmatrix} $$

where

3.23 $$\begin{aligned} \begin{aligned} &l_{1} = \varepsilon +\xi +\lambda +\mu _{1}, \\ &l_{2} = \theta +\mu +\chi +\mu _{1}, \\ &l_{3} = \eta +\varphi +\mu _{1}, \\ &l_{4} = v+\xi +\mu _{1}, \\ &l_{5} = \sigma +\tau +\mu _{1}. \end{aligned} \end{aligned}$$

Then we have

3.24 $$ V^{-1}= \begin{bmatrix} \frac{1}{l_{1}} & 0 & 0 & 0 & 0 \\ \frac{\xi }{l_{1}l_{2}} & \frac{1}{l_{2}} & 0 & 0 & 0 \\ \frac{\varepsilon }{l_{1}l_{3}} & 0 & \frac{1}{l_{3}} & 0 & 0 \\ \frac{\eta l_{2}\varepsilon +\xi l_{3}\theta }{l_{1}l_{2}l_{3}l_{4}} & \frac{\theta }{l_{2}l_{4}} & \frac{\eta }{l_{3}l_{4}} & \frac{1}{l_{4}} & 0 \\ \frac{\eta l_{2}\varepsilon v+\xi l_{3}l_{4}\mu +\xi l_{3}v\theta }{l_{1}l_{2}l_{3}l_{4}l_{5}} & \frac{l_{4}\mu +v\theta }{l_{2}l_{4}l_{5}} & \frac{v\eta }{l_{3}l_{4}l_{5}} & \frac{v}{l_{4}l_{5}} & \frac{1}{l_{5}}\end{bmatrix}. $$

So we write the following:

3.25 $$\begin{aligned} {FV}^{-1} ={}& \begin{bmatrix} {\delta } ( x ) & {\gamma \delta } ( x ) { w} & { \beta \delta } ( x ) { w} & { \delta } ( x ) { w\delta }_{1} & { 0} \\ { 0} & { 0} & { 0} & { 0} & { 0} \\ { 0} & { 0} & { 0} & { 0} & { 0} \\ { 0} & { 0} & { 0} & { 0} & { 0} \\ { 0} & { 0} & { 0} & { 0} & { 0}\end{bmatrix} \\ &{}\times \begin{bmatrix} \frac{1}{l_{1}} & { 0} & { 0} & { 0} & { 0} \\ \frac{\xi }{l_{1}l_{2}} & \frac{1}{l_{2}} & { 0} & { 0} & { 0} \\ \frac{\varepsilon }{l_{1}l_{3}} & { 0} & \frac{1}{l_{3}} & { 0} & { 0} \\ \frac{\eta l_{2}\varepsilon +\xi l_{3}\theta }{l_{1}l_{2}l_{3}l_{4}} & \frac{\theta }{l_{2}l_{4}} & \frac{\eta }{l_{3}l_{4}} & \frac{1}{l_{4}} & { 0} \\ \frac{\eta l_{2}\varepsilon v+\xi l_{3}l_{4}\mu +\xi l_{3}v\theta }{l_{1}l_{2}l_{3}l_{4}l_{5}} & \frac{l_{4}\mu +v\theta }{l_{2}l_{4}l_{5}} & \frac{v\eta }{l_{3}l_{4}l_{5}} & \frac{v}{l_{4}l_{5}} & \frac{1}{l_{5}}\end{bmatrix}. \end{aligned}$$

Therefore, using $R_{0}=\rho ( { FV}^{-1} ) $, the basic reproductive number is given as

3.26 $$ R_{0}= \biggl\{ \frac{\delta ( x ) }{l_{1}l_{2}l_{3}l_{4}} \bigl( l_{2}l_{3}l_{4}+ \xi l_{3} ( \gamma wl_{4}+w\theta \delta _{1} ) +w\varepsilon l_{2} ( \beta l_{4}+\eta \delta _{1} ) \bigr) \biggr\} . $$

We now present disease equilibrium points. We achieve this by solving

3.27 $$\begin{aligned} &\Lambda - \bigl( \alpha ( x ) +\gamma _{1}+\mu _{1} \bigr) S = 0, \\ &\alpha ( x ) S- ( \varepsilon +\xi +\lambda +\mu _{1} ) I = 0, \\ &\xi I- ( \theta +\mu +\chi +\mu _{1} ) I_{A} = 0, \\ &\varepsilon I- ( \eta +\varphi +\mu _{1} ) I_{D} = 0, \\ &\eta I_{D}+\theta I_{A}- ( v+\xi +\mu _{1} ) I_{R} = 0, \\ &\mu I_{A}+vI_{R}- ( \sigma +\tau +\mu _{1} ) I_{T} = 0, \\ &\lambda I+\varphi I_{D}+\chi I_{A}+\xi I_{R}+\sigma I_{T}- ( \Phi +\mu _{1} ) R = 0, \\ &\tau I_{T} = 0, \\ &\gamma _{1}S+\Phi R-\mu _{1}V = 0. \end{aligned}$$

This implies that

3.28 $$\begin{aligned} \begin{aligned} &I_{A} = \frac{\xi }{\theta +\mu +\chi +\mu _{1}}I, \\ &I_{D} = \frac{\varepsilon }{\eta +\varphi +\mu _{1}}I, \\ &I_{R} = \biggl( \frac{\eta \varepsilon }{\eta +\varphi +\mu _{1}}+ \frac{\theta \xi }{\theta +\mu +\chi +\mu _{1}} \biggr) \frac{I}{v+\xi +\mu _{1}}, \\ &I_{T} = \biggl( \frac{\mu \xi }{\theta +\mu +\chi +\mu _{1}}+ \frac{\chi \eta \varepsilon }{\eta +\varphi +\mu _{1}}+ \frac{v\theta \xi }{\theta +\mu +\chi +\mu _{1}} \biggr) \frac{I}{\sigma +\tau +\mu _{1}}, \\ &V = \frac{\gamma _{1}}{\mu _{1}}S+\frac{\Phi }{\mu _{1}}R. \end{aligned} \end{aligned}$$

Thus

3.29 $$\begin{aligned} \begin{aligned} &S^{\ast }=\frac{\delta ( x ) }{N} \begin{pmatrix} 1+\frac{\beta \varepsilon w}{\eta +\varphi +\mu _{1}}+ \frac{\gamma \xi w}{\theta +\mu +\chi +\mu _{1}}+ \frac{\delta _{1}\eta \varepsilon w}{ ( \eta +\varphi +\mu _{1} ) ( v+\xi +\mu _{1} ) } \\ + \frac{\delta _{1}\theta \xi w}{ ( \theta +\mu +\chi +\mu _{1} ) ( v+\xi +\mu _{1} ) }\end{pmatrix}, \\ &\Lambda - \bigl( \delta ( x ) \bigl( I^{\ast }+w\beta I_{D}^{ \ast }+\gamma wI_{A}^{\ast }+w\delta _{1}I_{R}^{\ast } \bigr) + ( \gamma _{1}+\mu _{1} ) \bigr) S^{\ast }=0, \end{aligned} \end{aligned}$$

and

3.30 $$ \frac{\Lambda + ( \gamma _{1}+\mu _{1} ) S^{\ast }}{A}=I^{ \ast } ,$$

where

3.31 $$ A=\frac{\delta ( x ) }{N} \begin{pmatrix} 1+\frac{\beta \varepsilon w}{\eta +\varphi +\mu _{1}}+ \frac{\gamma \xi w}{\theta +\mu +\chi +\mu _{1}}+ \frac{\delta _{1}\eta \varepsilon w}{ ( \eta +\varphi +\mu _{1} ) ( v+\xi +\mu _{1} ) } \\ + \frac{\delta _{1}\theta \xi w}{ ( \theta +\mu +\chi +\mu _{1} ) ( v+\xi +\mu _{1} ) }\end{pmatrix}. $$

That is,

3.32 $$\begin{aligned} \begin{aligned} &I^{\ast } = \frac{\Lambda A+ ( \gamma _{1}+\mu _{1} ) ( \xi +\varepsilon +\lambda +\mu _{1} ) }{A^{2}}, \\ &I_{A}^{\ast } = \frac{\xi }{\theta +\mu +\chi +\mu _{1}} \biggl( \frac{\Lambda A+ ( \gamma _{1}+\mu _{1} ) ( \xi +\varepsilon +\lambda +\mu _{1} ) }{A^{2}} \biggr), \\ &I_{D}^{\ast } = \frac{\varepsilon }{\eta +\varphi +\mu _{1}} \biggl( \frac{\Lambda A+ ( \gamma _{1}+\mu _{1} ) ( \xi +\varepsilon +\lambda +\mu _{1} ) }{A^{2}} \biggr), \\ &I_{R}^{\ast } = \biggl( \frac{\eta \varepsilon }{\eta +\varphi +\mu _{1}}+\frac{\theta \xi }{\theta +\mu +\chi +\mu _{1}} \biggr) \biggl( \frac{\Lambda A+ ( \gamma _{1}+\mu _{1} ) ( \xi +\varepsilon +\lambda +\mu _{1} ) }{A^{2}} \biggr), \\ &I_{T}^{\ast } = \frac{\Lambda A+ ( \gamma _{1}+\mu _{1} ) ( \xi +\varepsilon +\lambda +\mu _{1} ) }{A^{2} ( \sigma +\tau +\mu _{1} ) }\\ &\phantom{I_{T}^{\ast } =}{}\times \biggl( \frac{\mu \xi }{\theta +\mu +\chi +\mu _{1}}+ \frac{\chi \eta \varepsilon }{\eta +\varphi +\mu _{1}}+ \frac{v\theta \xi }{\theta +\mu +\chi +\mu _{1}} \biggr). \end{aligned} \end{aligned}$$

Also we get

3.33 $$\begin{array}{rl} R^{*}= & \frac{\Lambda A+(\gamma_{1}+\mu_{1})(\xi +\varepsilon +\lambda +\mu_{1})}{A^{2}(\Phi +\mu_{1})}\\ & \times \left\{\begin{array}{c} \lambda +\frac{\eta \varepsilon }{\eta +\varphi +\mu_{1}}+\frac{\chi \xi }{\theta +\mu +\chi +\mu_{1}}\\ +\frac{\xi }{v+\xi +\mu_{1}}(\frac{\eta \varepsilon }{\eta +\varphi +\mu_{1}}+\frac{\theta \xi }{\theta +\mu +\chi +\mu_{1}})+\frac{\sigma \mu \xi }{\left(\theta +\mu +\chi +\mu_{1})(\sigma +\tau +\mu_{1}\right)}\\ +\frac{v\eta \varepsilon \sigma }{\left(\eta +\varphi +\mu_{1})(\sigma +\tau +\mu_{1}\right)}+\frac{\sigma v\theta \xi }{\left(\theta +\mu +\chi +\mu_{1})(\sigma +\tau +\mu_{1}\right)} \end{array}\right\} \end{array}$$

and

3.34 $$\begin{array}{rl} V^{*}= & \frac{\gamma_{1}}{\mu_{1}}\frac{\delta (x)}{N}\left(\begin{array}{c} 1+\frac{\beta \varepsilon w}{\eta +\varphi +\mu_{1}}+\frac{\gamma \xi w}{\theta +\mu +\chi +\mu_{1}}+\frac{\delta_{1}\eta \varepsilon w}{\left(\eta +\varphi +\mu_{1})(v+\xi +\mu_{1}\right)}\\ +\frac{\delta_{1}\theta \xi w}{\left(\theta +\mu +\chi +\mu_{1})(v+\xi +\mu_{1}\right)} \end{array}\right)\\ & +\frac{\Phi }{\mu_{1}}\frac{\Lambda A+(\gamma_{1}+\mu_{1})(\xi +\varepsilon +\lambda +\mu_{1})}{A^{2}(\Phi +\mu_{1})}\\ & \times \left\{\begin{array}{c} \lambda +\frac{\eta \varepsilon }{\eta +\varphi +\mu_{1}}+\frac{\chi \xi }{\theta +\mu +\chi +\mu_{1}}\\ +\frac{\xi }{v+\xi +\mu_{1}}(\frac{\eta \varepsilon }{\eta +\varphi +\mu_{1}}+\frac{\theta \xi }{\theta +\mu +\chi +\mu_{1}})+\frac{\sigma \mu \xi }{\left(\theta +\mu +\chi +\mu_{1})(\sigma +\tau +\mu_{1}\right)}\\ +\frac{v\eta \varepsilon \sigma }{\left(\eta +\varphi +\mu_{1})(\sigma +\tau +\mu_{1}\right)}+\frac{\sigma v\theta \xi }{\left(\theta +\mu +\chi +\mu_{1})(\sigma +\tau +\mu_{1}\right)} \end{array}\right\}. \end{array}$$

For the Covid-19 endemic with this model, we need to have

3.35 $$ \overset{\cdot }{I} ( t ) >0,\qquad \overset{\cdot }{I_{A}} ( t ) >0,\qquad \overset{\cdot }{I_{D}} ( t ) >0,\qquad \overset{\cdot }{I_{R}} ( t ) >0\quad\text{and}\quad \overset{\cdot }{I_{T}} ( t ) >0. $$

This implies

3.36 $$\begin{aligned} \begin{aligned} &\alpha ( x ) S- ( \varepsilon +\xi +\lambda +\mu _{1} ) I >0, \\ &\xi I- ( \theta +\mu +\chi +\mu _{1} ) I_{A} >0, \\ &\varepsilon I- ( \eta +\varphi +\mu _{1} ) I_{D} >0, \\ &\eta I_{D}+\theta I_{A}- ( v+\xi +\mu _{1} ) I_{R} >0, \\ &\mu I_{A}+vI_{R}- ( \sigma +\tau +\mu _{1} ) I_{T} >0, \quad\forall t\geq 0. \end{aligned} \end{aligned}$$

Thus

3.37 $$\begin{aligned} \begin{aligned} &I< \frac{\alpha ( x ) S}{\varepsilon +\xi +\lambda +\mu _{1}}, \\ &I_{A}< \frac{\xi }{\theta +\mu +\chi +\mu _{1}}I, \\ &I_{D}< \frac{\varepsilon }{\eta +\varphi +\mu _{1}}I, \\ &I_{R}< \frac{\eta }{ ( v+\xi +\mu _{1} ) }I_{D}+ \frac{\theta }{ ( v+\xi +\mu _{1} ) }I_{A}, \\ &I_{T}< \frac{\mu }{ ( \sigma +\tau +\mu _{1} ) }I_{A}+ \frac{v}{ ( \sigma +\tau +\mu _{1} ) }I_{R}. \end{aligned} \end{aligned}$$

We use the fact that $\frac{S}{N}<1 $, to get

3.38 $$ \frac{\delta ( x ) }{\varepsilon +\xi +\lambda +\mu _{1}} ( I+w\beta I_{D}+\gamma wI_{A}+w \delta _{1}I_{R} ) >I, $$

noting that

3.39 $$\begin{aligned} \begin{aligned} &I_{A} < \frac{\xi }{\theta +\mu +\chi +\mu _{1}}I, \\ &I_{D} < \frac{\varepsilon }{\eta +\varphi +\mu _{1}}I, \\ &I_{R} < \frac{\eta }{v+\xi +\mu _{1}}I_{D}+ \frac{\theta }{v+\xi +\mu _{1}}I_{A} \\ &\phantom{I_{R}}< \frac{\eta \varepsilon }{ ( v+\xi +\mu _{1} ) ( \eta +\varphi +\mu _{1} ) }I+ \frac{\xi \theta }{ ( \theta +\mu +\chi +\mu _{1} ) ( v+\xi +\mu _{1} ) }I. \end{aligned} \end{aligned}$$

Also

3.40 $$\begin{aligned} \begin{aligned} &w\beta I_{D} < \frac{w\beta \varepsilon I}{\eta +\varphi +\mu _{1}}, \\ &w\gamma _{1}I_{A} < \frac{w\gamma _{1}\xi I}{\theta +\mu +\chi +\mu _{1}}. \end{aligned} \end{aligned}$$

Therefore we have the following inequalities in terms of *I*:

3.41 $$ \frac{\delta ( x ) }{\varepsilon +\xi +\lambda +\mu _{1}} \begin{pmatrix} I+\frac{w\beta \varepsilon }{\eta +\varphi +\mu _{1}}I+ \frac{w\gamma _{1}\xi }{\theta +\mu +\chi +\mu _{1}}I \\ + \frac{w\eta \varepsilon \delta _{1}}{ ( v+\xi +\mu _{1} ) ( \eta +\varphi +\mu _{1} ) }I+ \frac{w\delta _{1}\xi \theta }{ ( \theta +\mu +\chi +\mu _{1} ) ( v+\xi +\mu _{1} ) }I\end{pmatrix} >I $$

and

3.42 $$ \frac{\delta ( x ) }{\varepsilon +\xi +\lambda +\mu _{1}} \begin{pmatrix} 1+\frac{w\beta \varepsilon }{\eta +\varphi +\mu _{1}}+ \frac{w\gamma _{1}\xi }{\theta +\mu +\chi +\mu _{1}} \\ + \frac{w\eta \varepsilon \delta _{1}}{ ( v+\xi +\mu _{1} ) ( \eta +\varphi +\mu _{1} ) }+ \frac{w\delta _{1}\xi \theta }{ ( \theta +\mu +\chi +\mu _{1} ) ( v+\xi +\mu _{1} ) }\end{pmatrix} >1. $$

Therefore

3.43 $$ R_{0}>1, $$

where

3.44 $$ R_{0}= \frac{\delta ( x ) }{\varepsilon +\xi +\lambda +\mu _{1}} \begin{pmatrix} 1+\frac{w\beta \varepsilon }{\eta +\varphi +\mu _{1}}+ \frac{w\gamma _{1}\xi }{\theta +\mu +\chi +\mu _{1}} \\ + \frac{w\eta \varepsilon \delta _{1}}{ ( v+\xi +\mu _{1} ) ( \eta +\varphi +\mu _{1} ) }+ \frac{w\delta _{1}\xi \theta }{ ( \theta +\mu +\chi +\mu _{1} ) ( v+\xi +\mu _{1} ) }\end{pmatrix}. $$

This shows that we have a unique endemic equilibrium when $R_{0}>1$.

### Local and global stability of the disease-free equilibrium

#### Lemma 3.1

*The disease*-*free equilibrium* $E_{0}$ *of the Covid*-19 *system is locally asymptotically stable when* $R_{0}<1$ *and unstable when* $R_{0}>1$. *The Jacobian matrix for Covid*-19 *system is given by*

$$ { \begin{bmatrix} - ( \gamma _{1}+\mu _{1} ) & 0 & 0 & 0 & 0 & 0 & 0 & 0 \\ 0 & - ( \varepsilon +\xi +\lambda +\mu _{1} ) & 0 & 0 & 0 & 0 & 0 & 0 \\ 0 & \xi & - ( \theta +\mu +\chi +\mu _{1} ) & 0 & 0 & 0 & 0 & 0 \\ 0 & \varepsilon & 0 & - ( \eta +\varphi +\mu _{1} ) & 0 & 0 & 0 & 0 \\ 0 & 0 & \theta & \eta & - ( v+\xi +\mu _{1} ) & 0 & 0 & 0 \\ 0 & 0 & \mu & 0 & v & - ( \sigma +\tau +\mu _{1} ) & 0 & 0 \\ 0 & \lambda & \chi & \varphi & \xi & \sigma & - ( \Phi +\mu _{1} ) & 0 \\ 0 & 0 & 0 & 0 & 0 & 0 & \Phi & -\mu _{1}\end{bmatrix} .} $$

*It is known that the disease*-*free equilibrium* $E_{0}$ *is asymptotically stable if and only if* $\mathrm{tr} ( J ( E_{0} ) ) <0$ *and* $\det ( J ( E_{0} ) ) >0$. *For the suggested Covid*-19, *the trace of* $J ( E_{0} ) $ *is*

3.45 $$ \mathrm{tr} \bigl( J ( E_{0} ) \bigr) =- ( \gamma _{1}+8 \mu _{1}+2\xi +\varepsilon +\lambda +\theta +\Phi +\tau +\mu + \varphi +\eta +v+\chi ) < 0. $$

*The determinant of* $J ( E_{0} ) $ *is*

3.46 $$\begin{aligned} \det \bigl( J ( E_{0} ) \bigr) ={}& ( \gamma _{1}+ \mu _{1} ) ( \varepsilon +\xi +\lambda +\mu _{1} ) ( \theta + \mu +\chi +\mu _{1} ) ( \eta +\varphi + \mu _{1} ) \\ &{}\times ( v+\xi +\mu _{1} ) ( \sigma +\tau +\mu _{1} ) ( \Phi +\mu _{1} ) \mu _{1}>0. \end{aligned}$$

*In this case*, *we can conclude that the disease*-*free equilibrium of the suggested model for Covid*-19 *under vaccination and treatment is locally asymptotically stable*.

#### Theorem 3.2

*The Covid*-19 *model disease*-*free equilibrium is globally asymptotically stable within the feasible interval if* $R_{0}<1$ *and unstable if* $R_{0}>1$.

#### Proof

We use the Lyapunov function defined by

3.47 $$ L=\frac{1}{l_{1}}I+\frac{1}{l_{2}}I_{A}+ \frac{1}{l_{3}}I_{D}+ \frac{1}{l_{4}}I_{R}+ \frac{1}{l_{5}}I_{T}. $$

Therefore its derivative along the solutions of the Covid-19 model is

3.48 $$ \frac{dL}{dt}=\frac{1}{l_{1}}\frac{dI}{dt}+\frac{1}{l_{2}} \frac{dI_{A}}{dt}+\frac{1}{l_{3}}\frac{dI_{D}}{dt}+ \frac{1}{l_{4}}\frac{dI_{R}}{dt}+ \frac{1}{l_{5}}\frac{dI_{T}}{dt}, $$

where

3.49 $$\begin{aligned} \begin{aligned} &l_{1} = \varepsilon +\xi +\lambda +\mu _{1}, \\ &l_{2} = \theta +\mu +\chi +\mu _{1}, \\ &l_{3} = \eta +\varphi +\mu _{1}, \\ &l_{4} = v+\xi +\mu _{1}, \\ &l_{5} = \sigma +\tau +\mu _{1}. \end{aligned} \end{aligned}$$

Then we write

3.50 $$\begin{aligned} \frac{dL}{dt} ={}& \frac{1}{l_{1}} \bigl( \alpha ( x ) S-l_{1}I \bigr) +\frac{1}{l_{2}} ( \xi I-l_{2}I_{A} ) + \frac{1}{l_{3}} ( \varepsilon I-l_{3}I_{D} ) \\ &{}+\frac{1}{l_{4}} ( \eta I_{D}+\theta I_{A}-l_{4}I_{R} ) + \frac{1}{l_{5}} ( \mu I_{A}+vI_{R}-l_{5}I_{T} ). \end{aligned}$$

We have, on the other hand, that

3.51 $$ \alpha ( x ) =\frac{\delta ( x ) }{N} \bigl( I+w ( \beta I_{D}+\gamma I_{A}+\delta _{1}I_{R} ) \bigr), $$

and we get

3.52 $$\begin{array}{rl} \frac{dL}{dt}= & \frac{1}{l_{1}}\left(\delta (x)(I+w\beta I_{D}+w\gamma I_{A}+w\delta_{1}I_{R})-l_{1}I\right)+\frac{1}{l_{2}}(\xi I-l_{2}I_{A})\\ & +\frac{1}{l_{3}}(\varepsilon I-l_{3}I_{D})+\frac{1}{l_{4}}(\eta I_{D}+\theta I_{A}-l_{4}I_{R})+\frac{1}{l_{5}}(\mu I_{A}+vI_{R}-l_{5}I_{T}),\\ \frac{dL}{dt}= & \left\{\begin{array}{c} \frac{\delta (x)}{l_{1}}(I+w\beta I_{D}+w\gamma I_{A}+w\delta_{1}I_{R})+\frac{1}{l_{2}}\xi I+\frac{1}{l_{3}}\varepsilon I\\ +\frac{1}{l_{4}}(\eta I_{D}+\theta I_{A})+\frac{1}{l_{5}}(\mu I_{A}+vI_{R}) \end{array}\right\}\\ & -(I+I_{D}+I_{A}+I_{R}+I_{T}),\\ \frac{dL}{dt}= & \left[\begin{array}{c} \left\{\begin{array}{c} \frac{\delta (x)}{l_{1}}(I+w\beta I_{D}+w\gamma I_{A}+w\delta_{1}I_{R})+\frac{1}{l_{2}}\xi I+\frac{1}{l_{3}}\varepsilon I\\ +\frac{1}{l_{4}}(\eta I_{D}+\theta I_{A})+\frac{1}{l_{5}}(\mu I_{A}+vI_{R}) \end{array}\right\}\\ \times \frac{1}{\left(I+I_{D}+I_{A}+I_{R}+I_{T}\right)}-1 \end{array}\right]\\ & \times (I+I_{D}+I_{A}+I_{R}+I_{T}). \end{array}$$

We now divide by *I* to obtain

$$\begin{array}{rl} \frac{dL}{dt}= & \left[\begin{array}{c} \left\{\begin{array}{c} \frac{\delta (x)}{l_{1}}(1+w\beta \frac{I_{D}}{I}+w\gamma \frac{I_{A}}{I}+w\delta_{1}\frac{I_{R}}{I})+\frac{\xi }{l_{2}}+\frac{\varepsilon }{l_{3}}\\ +\frac{1}{l_{4}}\frac{\left(\eta I_{D}+\theta I_{A}\right)}{I}+\frac{1}{l_{5}}\frac{\left(\mu I_{A}+vI_{R}\right)}{I} \end{array}\right\}\\ +\frac{1}{I(I+I_{D}+I_{A}+I_{R}+I_{T})}-1 \end{array}\right]\\ & \times (I+I_{D}+I_{A}+I_{R}+I_{T}). \end{array}$$

However, since *I* is greater than $I_{D},I_{A},I_{R},I_{T}$ classes, one gets

3.53 $$\begin{aligned} \frac{dL}{dt} &\leq \left \{ \frac{\delta ( x ) }{l_{1}} \begin{pmatrix} 1+\frac{w\beta \varepsilon }{\eta +\varphi +\mu _{1}}+ \frac{w\delta _{1}\xi }{\theta +\mu +\chi +\mu _{1}} \\ + \frac{w\delta _{1}\eta \varepsilon }{ ( \xi +\varphi +\mu _{1} ) ( v+\xi +\mu _{1} ) }+ \frac{w\delta _{1}\xi \theta }{ ( v+\xi +\mu _{1} ) ( \theta +\mu +\chi +\mu _{1} ) }\end{pmatrix} -1 \right \} ( I+I_{D}+I_{A}+I_{R}+I_{T} ) \\ &\leq ( I+I_{D}+I_{A}+I_{R}+I_{T} ) ( R_{0}-1 ) \leq 0\quad\text{if }R_{0}\leq 1, \end{aligned}$$

since $I+I_{D}+I_{A}+I_{R}+I_{T}>0,\forall t$. Therefore Covid-19 will be eliminated according to the suggested model if and only if $R_{0}<1$. In particular, since all parameters in the Covid-19 model are positive, then Lyapunov function decreases, i.e., $\frac{dL}{dt}<0$, if $R_{0}<1$ and increases if $R_{0}>1$, finally, $L=0$ if

3.54 $$ I=I_{D}=I_{A}=I_{R}=I_{T}=0. $$

Therefore *L* is a Lyapunov function within the feasible biological interval and the bigger compact invariant set in $\{ S,I,I_{D},I_{A},I_{R},I_{T},R,D,V\in \Omega:\frac{dL}{dt} \leq 0 \} $ is the point $E_{0}$. By the well-known Lasalle’s invariance principle [15], each solution of the Covid-19 model suggested in this work with initial condition in Ω leads to $E_{0}$ when $t\rightarrow \infty $ only if $R_{0}\leq 1$. In conclusion, the disease-free equilibrium $E_{0}$ of the Covid-19 model suggested here, which includes treatment and vaccination, is globally asymptotically stable. □

### Local and global stability of the endemic equilibrium

We compute first the Jacobian matrix of the Covid-19 model for the endemic equilibrium case:

3.55 $$ { JE_{\ast }= \begin{bmatrix} -\alpha ^{\ast } ( x ) - ( \gamma _{1}+\mu _{1} ) & -\delta ( x ) & - \gamma \delta ( x ) w & -\beta \delta ( x ) w & -\delta ( x ) w\delta _{1} & 0 & 0 & 0 \\ \alpha ^{\ast } ( x ) & \delta ( x ) -l_{1} & \gamma \delta ( x ) w & \beta\delta ( x ) w & \delta ( x ) w\delta _{1} & 0 & 0 & 0 \\ 0 & \xi & -l_{2} & 0 & 0 & 0 & 0 & 0 \\ 0 & \varepsilon & 0 & -l_{3} & 0 & 0 & 0 & 0 \\ 0 & 0 & \theta & \eta & -l_{4} & 0 & 0 & 0 \\ 0 & 0 & \mu & 0 & v & -l_{5} & 0 & 0 \\ 0 & \lambda & \chi & \varphi & \xi & \sigma & -l_{6} & 0 \\ \gamma _{1} & 0 & 0 & 0 & 0 & 0 & \Phi & -\mu _{1}\end{bmatrix} .} $$

We now construct a characteristic equation associated to this model

3.56 $$ P=\det \vert I_{M}\lambda -JE_{\ast } \vert =0, $$

where $I_{M}$ is the $8\times 8$ unit matrix. Then we have

3.57 $$ \det \begin{bmatrix} \lambda +l & -\delta ( x ) & - \gamma \delta ( x ) w & -\beta \delta ( x ) w & -\delta ( x ) w\delta _{1} & 0 & 0 & 0 \\ \alpha ^{\ast } & \lambda-\delta ( x ) +l_{1} & \gamma \delta ( x ) w & \beta\delta ( x ) w & \delta ( x ) w\delta _{1} & 0 & 0 & 0 \\ 0 & \xi & \lambda +l_{2} & 0 & 0 & 0 & 0 & 0 \\ 0 & \varepsilon & 0 & \lambda +l_{3} & 0 & 0 & 0 & 0 \\ 0 & 0 & \theta & \eta & \lambda +l_{4} & 0 & 0 & 0 \\ 0 & 0 & \mu & 0 & v & \lambda +l_{5} & 0 & 0 \\ 0 & \lambda & \chi & \varphi & \xi & \sigma & \lambda +l_{6} & 0 \\ \gamma _{1} & 0 & 0 & 0 & 0 & 0 & \Phi & \lambda +\mu _{1}\end{bmatrix} . $$

From the above, we obtain the following characteristic polynomial

3.58 $$ P ( \lambda ) =\lambda ^{8}+a_{1}\lambda ^{7}+a_{2} \lambda ^{6}+a_{3}\lambda ^{5}+a_{4}\lambda ^{4}+a_{5}\lambda ^{3}+a_{6} \lambda ^{2}+a_{7}\lambda +a_{8}. $$

The square Hurwitz matrix associated to the above polynomial $P ( \lambda ) $ is given as

3.59 $$ H= \begin{bmatrix} a_{1} & a_{3} & a_{5} & a_{7} & 0 & 0 & 0 & 0 \\ 1 & a_{2} & a_{4} & a_{6} & a_{8} & 0 & 0 & 0 \\ 0 & a_{1} & a_{3} & a_{5} & a_{7} & 0 & 0 & 0 \\ 0 & 1 & a_{2} & a_{4} & a_{6} & a_{8} & 0 & 0 \\ 0 & 0 & a_{1} & a_{3} & a_{5} & a_{7} & 0 & 0 \\ 0 & 0 & 1 & a_{2} & a_{4} & a_{6} & a_{8} & 0 \\ 0 & 0 & 0 & a_{1} & a_{3} & a_{5} & a_{7} & 0 \\ 0 & 0 & 0 & 1 & a_{2} & a_{4} & a_{6} & a_{8}\end{bmatrix} . $$

Then we have

3.60 $$\begin{aligned} H_{1}={}&a_{1}>0, \\ H_{2}={}&a_{1}a_{2}-a_{3}>0, \\ H_{3}={}&{-}a_{1}^{2}a_{4}+a_{1}a_{2}a_{3}+a_{1}a_{5}-a_{3}^{2}>0, \\ H_{4}={}&a_{1}^{2}a_{2}a_{6}-a_{1}^{2}a_{4}^{2}-a_{1}a_{2}^{2}a_{5}+a_{1}a_{2}a_{3}a_{4}-a_{1}a_{2}a_{7}-a_{1}a_{3}a_{6}+2a_{1}a_{4}a_{5} \\ &{}+a_{2}a_{3}a_{5}-a_{3}^{2}a_{4}+a_{3}a_{7}-a_{5}^{2}>0, \\ H_{5}={}&a_{1}^{3}a_{4}a_{8}-a_{1}^{3}a_{6}^{2}-a_{1}^{2}a_{2}a_{3}a_{8}-a_{1}^{2}a_{2}a_{4}a_{7}+2a_{1}^{2}a_{2}a_{5}a_{6}+a_{1}^{2}a_{3}a_{4}a_{6} \\ &{}+a_{1}a_{2}^{2}a_{3}a_{7}-a_{1}a_{2}^{2}a_{5}^{2}-a_{1}a_{2}a_{3}^{2}a_{6}+a_{1}a_{2}a_{3}a_{4}a_{5}-a_{1}^{2}a_{5}a_{8}+2a_{1}^{2}a_{6}a_{7} \\ &{}-a_{1}a_{2}a_{5}a_{7}-3a_{1}a_{3}a_{5}a_{6}-a_{1}a_{2}a +2a_{1}a_{4}a_{5}^{2}-a_{2}a_{3}^{2}a_{7}+a_{2}a_{3}a_{5}^{2} \\ &{}+a_{1}a_{3}^{2}a_{8}-a_{1}^{2}a_{4}^{2}a_{5}+a_{3}^{3}a_{6}-a_{3}^{2}a_{4}a_{5}-a_{1}a_{7}^{2}+2a_{3}a_{5}a_{7}-a_{5}^{3}>0, \\ H_{6}={}&{-}a_{1}^{3}a_{8}^{2}a_{2}+2a_{1}^{3}a_{4}a_{6}a_{8}-a_{1}^{3}a_{6}^{3}+2a_{1}^{2}a_{2}^{2}a_{7}a_{8}-a_{1}^{2}a_{2}a_{3}a_{6}a_{8}+a_{2}^{2}a_{3}a_{7}^{2} \\ &{}-3a_{1}^{2}a_{2}a_{6}a_{4}a_{7}+2a_{1}^{2}a_{2}a_{5}a_{6}^{2}-a_{1}^{2}a_{4}^{2}a_{3}a_{8}+a_{1}^{2}a_{6}^{2}a_{3}a_{4}+a_{1}^{2}a_{4}^{3}a_{7}-a_{1}^{2}a_{4}^{2}a_{5}a_{6} \\ &{}-a_{1}a_{2}^{3}a_{7}^{2}-a_{1}a_{2}^{2}a_{3}a_{8}a_{5}+2a_{1}a_{2}^{2}a_{3}a_{6}a_{7}+a_{1}a_{2}^{2}a_{4}a_{5}a_{7}-a_{1}a_{2}^{2}a_{5}^{2}a_{6}+a_{4}a_{5}^{2}a_{7} \\ &{}+a_{1}a_{2}a_{3}^{2}a_{4}a_{8}-a_{1}a_{2}a_{3}^{2}a_{6}^{2}-a_{1}a_{2}a_{3}a_{7}a_{4}^{2}+a_{1}a_{2}a_{3}a_{4}a_{5}a_{6}+a_{1}^{2}a_{3}a_{8}^{2} \\ &{}-2a_{1}^{2}a_{4}a_{7}a_{8}-2a_{1}^{2}a_{5}a_{6}a_{8}+3a_{1}^{2}a_{6}^{2}a_{7}-3a_{1}a_{2}a_{3}a_{7}a_{8}+a_{3}^{2}a_{4}^{2}a_{7}-3a_{1}a_{6}a_{7}^{2} \\ &{}-a_{5}^{3}a_{6}+3a_{1}a_{2}a_{4}a_{7}^{2}-a_{1}a_{2}a_{5}a_{7}a_{6}+a_{1}a_{3}^{2}a_{6}a_{8}+2a_{1}a_{4}a_{3}a_{5}a_{8}-a_{3}a_{8}a_{5}^{2} \\ &{}+a_{2}a_{3}a_{6}a_{5}^{2}+a_{1}a_{4}a_{3}a_{6}a_{7}-3a_{1}a_{3}a_{5}a_{6}^{2}-2a_{1}a_{4}^{2}a_{5}a_{7}+2a_{1}a_{4}a_{5}^{2}a_{6} \\ &{}+a_{2}a_{3}^{2}a_{5}a_{8}-2a_{2}a_{3}^{2}a_{6}a_{7}-a_{2}a_{3}a_{4}a_{5}a_{7}-a_{3}^{3}a_{4}a_{8}+a_{3}^{3}a_{6}^{2}+a_{7}^{3} \\ &{}-a_{3}^{2}a_{4}a_{5}a_{6}+2a_{1}a_{7}a_{5}a_{8}-a_{2}a_{7}^{2}a_{5}+a_{3}^{2}a_{7}a_{8}-2a_{4}a_{3}a_{7}^{2}+3a_{5}a_{3}a_{6}a_{7}>0, \\ H_{7} ={}& a_{1}^{4}a_{8}^{3} -3a_{1}^{3}a_{2}a_{7}a_{8}^{2}-a_{1}^{3}a_{3}a_{6}a_{8}^{2}-2a_{1}^{3}a_{8}^{2}a_{4}a_{5}+3a_{1}^{3}a_{4}a_{6}a_{8}a_{7}-a_{3}^{3}a_{5}a_{6}a_{8} \\ &{}+a_{1}^{3}a_{5}a_{6}^{2}a_{8}-a_{1}^{3}a_{6}^{3}a_{7}+3a_{1}^{2}a_{2}^{2}a_{7}^{2}a_{8}+3a_{1}^{2}a_{2}a_{3}a_{5}a_{8}^{2}-a_{1}^{2}a_{2}a_{3}a_{6}a_{7}a_{8}+a_{3}^{4}a_{8}^{2} \\ &{}+a_{1}^{2}a_{2}a_{5}a_{4}a_{7}a_{8}-3a_{1}^{2}a_{2}a_{4}a_{6}a_{7}^{2}-2a_{1}^{2}a_{2}a_{8}a_{6}a_{5}^{2}-a_{2}a_{3}a_{4}a_{5}a_{7}^{2}+a_{3}^{3}a_{6}^{2}a_{7} \\ &{}+2a_{1}^{2}a_{2}a_{5}a_{6}^{2}a_{7}+a_{1}^{2}a_{4}a_{3}^{2}a_{8}^{2}-2a_{1}^{2}a_{4}^{2}a_{3}a_{7}a_{8}-a_{1}^{2}a_{3}a_{4}a_{5}a_{6}a_{8}-2a_{3}^{3}a_{4}a_{7}a_{8} \\ &{}+a_{1}^{2}a_{6}^{2}a_{3}a_{4}a_{7}+a_{1}^{2}a_{4}^{3}a_{7}^{2}+a_{1}^{2}a_{4}^{2}a_{5}^{2}a_{8}-a_{1}^{2}a_{4}^{2}a_{5}a_{6}a_{7}-a_{1}a_{2}^{3}a_{7}^{3}+4a_{1}a_{5}a_{7}^{2}a_{8} \\ &{}-3a_{1}a_{2}^{2}a_{3}a_{5}a_{7}a_{8}+2a_{1}a_{2}^{2}a_{3}a_{6}a_{7}^{2}+a_{1}a_{2}^{2}a_{4}a_{5}a_{7}^{2}+a_{1}a_{2}^{2}a_{5}^{3}a_{8}+3a_{3}a_{5}a_{6}a_{7}^{2} \\ &{}-a_{1}a_{2}^{2}a_{5}^{2}a_{6}a_{7}-a_{1}a_{2}a_{3}^{3}a_{8}^{2}+2a_{1}a_{2}a_{3}^{2}a_{4}a_{7}a_{8}+a_{1}a_{2}a_{3}^{2}a_{5}a_{6}a_{8}-a_{1}a_{2}a_{3}^{2}a_{6}^{2}a_{7} \\ &{}-a_{1}a_{2}a_{3}a_{4}^{2}a_{7}^{2}-a_{1}a_{2}a_{3}a_{4}a_{5}^{2}a_{8}+a_{1}a_{2}a_{3}a_{4}a_{5}a_{6}a_{7}-a_{2}a_{3}a_{5}^{3}a_{8}+a_{2}a_{3}a_{5}^{2}a_{6}a_{7} \\ &{}+4a_{1}^{2}a_{3}a_{7}a_{8}^{2}-3a_{1}^{2}a_{4}a_{7}^{2}a_{8}+2a_{1}^{2}a_{5}^{2}a_{8}^{2}+a_{2}^{2}a_{3}a_{7}^{3}+3a_{2}a_{3}^{2}a_{5}a_{7}a_{8}+a_{3}^{2}a_{4}^{2}a_{7}^{2} \\ &{}-5a_{1}^{2}a_{5}a_{6}a_{7}a_{8}+3a_{1}^{2}a_{6}^{2}a_{7}^{2}-5a_{1}a_{2}a_{3}a_{7}^{2}a_{8}+3a_{1}a_{2}a_{4}a_{7}^{3}+a_{1}a_{2}a_{5}^{2}a_{7}a_{8} \\ &{}-a_{1}a_{2}a_{5}a_{6}a_{7}^{2}-4a_{1}a_{3}^{2}a_{5}a_{8}^{2}+a_{1}a_{3}^{2}a_{6}a_{7}a_{8}+4a_{1}a_{3}a_{4}a_{5}a_{7}a_{8}+a_{1}a_{3}a_{4}a_{6}a_{7}^{2} \\ &{}+3a_{1}a_{3}a_{5}^{2}a_{6}a_{8}-3a_{1}a_{3}a_{5}a_{6}^{2}a_{7}-2a_{1}a_{4}^{2}a_{5}a_{7}^{2}-2a_{1}a_{4}a_{5}^{3}a_{8}+2a_{1}a_{4}a_{5}^{2}a_{6}a_{7} \\ &{}-2a_{2}a_{3}^{2}a_{6}a_{7}^{2}+a_{3}^{2}a_{5}^{2}a_{4}a_{8}-a_{3}^{2}a_{4}a_{5}a_{6}a_{7}-3a_{1}a_{6}a_{7}^{3}-a_{2}a_{5}a_{7}^{3}+2a_{3}^{2}a_{7}^{2}a_{8} \\ &{}-2a_{3}a_{4}a_{7}^{3}-4a_{3}a_{5}^{2}a_{7}a_{8}+a_{4}a_{5}^{2}a_{7}^{2}+a_{5}^{4}a_{8}-a_{5}^{3}a_{6}a_{7}+a_{7}^{4}>0, \\ H_{8} ={}& a_{8}H_{7}>0. \end{aligned}$$

#### Theorem 3.3

*If* $R_{0}\geq 1$, *the endemic equilibrium point* $E_{\ast }$ *of the Covid*-19 *system is globally asymptotically stable*.

#### Proof

We prove this using the Lyapunov function

3.61 $$\begin{aligned} &L \bigl( S^{\ast },I^{\ast },I_{A}^{\ast },I_{D}^{\ast },I_{R}^{\ast },I_{T}^{ \ast },R^{\ast },D^{\ast },V^{\ast } \bigr) \\ &\quad = \biggl( S-S^{\ast }-S^{ \ast }\log \frac{S^{\ast }}{S} \biggr) + \biggl( I-I^{\ast }-I^{\ast } \log \frac{I^{\ast }}{I} \biggr) \\ &\qquad{}+ \biggl( I_{A}-I_{A}^{\ast }-I_{A}^{\ast } \log \frac{I_{A}^{\ast }}{I_{A}} \biggr) + \biggl( I_{D}-I_{D}^{\ast }-I_{D}^{\ast } \log \frac{I_{D}^{\ast }}{I_{D}} \biggr) \\ &\qquad{}+ \biggl( I_{R}-I_{R}^{\ast }-I_{R}^{\ast } \log \frac{I_{R}^{\ast }}{I_{R}} \biggr) + \biggl( I_{T}-I_{T}^{\ast }-I_{T}^{\ast } \log \frac{I_{T}^{\ast }}{I_{T}} \biggr) \\ &\qquad{}+ \biggl( R-R^{\ast }-R^{\ast }\log \frac{R^{\ast }}{R} \biggr) + \biggl( D-D^{\ast }-D^{\ast }\log \frac{D^{\ast }}{D} \biggr) \\ &\qquad{}+ \biggl( V-V^{\ast }-V^{\ast }\log \frac{V^{\ast }}{V} \biggr). \end{aligned}$$

Therefore taking the derivative with respect to *t* on both sides gives

3.62 $$\begin{aligned} \frac{dL}{dt} = \overset{\cdot }{L}={}& \biggl( \frac{S-S^{\ast }}{S} \biggr) \overset{\cdot }{S}+ \biggl( \frac{I-I^{\ast }}{I} \biggr) \overset{\cdot }{I}+ \biggl( \frac{I_{A}-I_{A}^{\ast }}{I_{A}} \biggr) \overset{\cdot }{I_{A}}+ \biggl( \frac{I_{D}-I_{D}^{\ast }}{I_{D}} \biggr) \overset{ \cdot }{I_{D}} \\ &{}+ \biggl( \frac{I_{R}-I_{R}^{\ast }}{I_{R}} \biggr) \overset{\cdot }{I_{R}}+ \biggl( \frac{I_{T}-I_{T}^{\ast }}{I_{T}} \biggr) \overset{\cdot }{I_{T}}+ \biggl( \frac{R-R^{\ast }}{R} \biggr) \overset{\cdot }{R}+ \biggl( \frac{D-D^{\ast }}{D} \biggr) \overset{\cdot }{D} \\ &{}+ \biggl( \frac{V-V^{\ast }}{V} \biggr) \overset{\cdot }{V}, \end{aligned}$$

where replacing $\overset{\cdot }{S},\overset{\cdot }{I},\overset{\cdot }{I_{A}},\overset{\cdot }{I_{D}},\overset{\cdot }{I_{R}}, \overset{\cdot }{I_{T}},\overset{\cdot }{R},\overset{\cdot }{D,}$ and $\overset{\cdot }{V}$ by their values, we obtain

3.63 $$\begin{aligned} \frac{dL}{dt} ={}& \biggl( \frac{S-S^{\ast }}{S} \biggr) \bigl( \Lambda - \bigl( \alpha ( x ) +\gamma _{1}+\mu _{1} \bigr) S \bigr) + \biggl( \frac{I-I^{\ast }}{I} \biggr) \bigl( \alpha ( x ) S-l_{1}I \bigr) \\ &{}+ \biggl( \frac{I_{A}-I_{A}^{\ast }}{I_{A}} \biggr) ( \xi I-l_{2}I_{A} ) + \biggl( \frac{I_{D}-I_{D}^{\ast }}{I_{D}} \biggr) ( \varepsilon I-l_{3}I_{D} ) \\ &{}+ \biggl( \frac{I_{R}-I_{R}^{\ast }}{I_{R}} \biggr) ( \eta I_{D}+ \theta I_{A}-l_{4}I_{R} ) + \biggl( \frac{I_{T}-I_{T}^{\ast }}{I_{T}} \biggr) ( \mu I_{A}+vI_{R}-l_{5}I_{T} ) \\ &{}+ \biggl( \frac{R-R^{\ast }}{R} \biggr) ( \lambda I+\varphi I_{D}+ \chi I_{A}+\xi I_{R}+\sigma I_{T}-l_{6}R ) + \biggl( \frac{D-D^{\ast }}{D} \biggr) ( \tau I_{T} ) \\ &{}+ \biggl( \frac{V-V^{\ast }}{V} \biggr) ( \gamma _{1}S+\Phi R- \mu _{1}V ). \end{aligned}$$

Then we have

3.64 $$\begin{aligned} \frac{dL}{dt} ={}& \biggl( \frac{S-S^{\ast }}{S} \biggr) \bigl( \Lambda - \bigl( \alpha ( x ) \bigl( S-S^{\ast } \bigr) + \gamma _{1} \bigl( S-S^{\ast } \bigr) +\mu _{1} \bigl( S-S^{\ast } \bigr) \bigr) \bigr) \\ &{}+ \biggl( \frac{I-I^{\ast }}{I} \biggr) \bigl( \alpha ( x ) \bigl( S-S^{\ast } \bigr) -l_{1} \bigl( I-I^{\ast } \bigr) \bigr) \\ &{}+ \biggl( \frac{I_{A}-I_{A}^{\ast }}{I_{A}} \biggr) \bigl( \xi \bigl( I-I^{\ast } \bigr) -l_{2} \bigl( I_{A}-I_{A}^{\ast } \bigr) \bigr) + \biggl( \frac{I_{D}-I_{D}^{\ast }}{I_{D}} \biggr) \bigl( \varepsilon \bigl( I-I^{\ast } \bigr) -l_{3} \bigl( I_{D}-I_{D}^{ \ast } \bigr) \bigr) \\ &{}+ \biggl( \frac{I_{R}-I_{R}^{\ast }}{I_{R}} \biggr) \bigl( \eta \bigl( I_{D}-I_{D}^{\ast } \bigr) +\theta \bigl( I_{A}-I_{A}^{ \ast } \bigr) -l_{4} \bigl( I_{R}-I_{R}^{\ast } \bigr) \bigr) \\ &{}+ \biggl( \frac{I_{T}-I_{T}^{\ast }}{I_{T}} \biggr) \bigl( \mu \bigl( I_{A}-I_{A}^{\ast } \bigr) +v \bigl( I_{R}-I_{R}^{\ast } \bigr) -l_{5} \bigl( I_{T}-I_{T}^{\ast } \bigr) \bigr) \\ &{}+ \biggl( \frac{R-R^{\ast }}{R} \biggr) \begin{pmatrix} \lambda ( I-I^{\ast } ) +\varphi ( I_{D}-I_{D}^{ \ast } ) +\chi ( I_{A}-I_{A}^{\ast } ) +\xi ( I_{R}-I_{R}^{ \ast } ) \\ +\sigma ( I_{T}-I_{T}^{\ast } ) -l_{6} ( R-R^{\ast } )\end{pmatrix} \\ &{}+ \biggl( \frac{D-D^{\ast }}{D} \biggr) \bigl( \tau \bigl( I_{T}-I_{T}^{ \ast } \bigr) \bigr) \\ &{}+ \biggl( \frac{V-V^{\ast }}{V} \biggr) \bigl( \gamma _{1} \bigl( S-S^{ \ast } \bigr) +\Phi \bigl( R-R^{\ast } \bigr) -\mu _{1} \bigl( V-V^{ \ast } \bigr) \bigr). \end{aligned}$$

The latter expression can be separated in two parts as follows:

3.65 $$\begin{aligned} \frac{dL}{dt} ={}& \frac{ ( S-S^{\ast } ) ^{2}}{S} \bigl( - \alpha ( x ) -\gamma _{1}-\mu _{1} \bigr) +\Lambda - \frac{S^{\ast }}{S}\Lambda -l_{1}\frac{ ( I-I^{\ast } ) ^{2}}{I}+\alpha ( x ) S-\alpha ( x ) S^{\ast } \\ &{}-\alpha ( x ) \frac{I^{\ast }}{I}S+\alpha ( x ) \frac{I^{\ast }}{I}S^{\ast }-l_{2} \frac{ ( I_{A}-I_{A}^{\ast } ) ^{2}}{I_{A}}+\xi I-\xi I^{\ast }-\xi \frac{I_{A}^{\ast }}{I_{A}}I+\xi \frac{I_{A}^{\ast }}{I_{A}}I^{\ast } \\ &{}-l_{3}\frac{ ( I_{D}-I_{D}^{\ast } ) ^{2}}{I_{D}}+ \varepsilon I-\varepsilon I^{\ast }-\varepsilon \frac{I_{D}^{\ast }}{I_{D}}I+\varepsilon \frac{I_{D}^{\ast }}{I_{D}}I^{ \ast }-l_{4}\frac{ ( I_{R}-I_{R}^{\ast } ) ^{2}}{I_{R}}+ \eta I_{D}-\eta I_{D}^{\ast } \\ &{}-\eta I_{D}\frac{I_{R}^{\ast }}{I_{R}}+\eta \frac{I_{R}^{\ast }}{I_{R}}+ \theta I_{A}-\theta I_{A}^{\ast }-\theta I_{A} \frac{I_{R}^{\ast }}{I_{R}}+\theta I_{A}^{\ast } \frac{I_{R}^{\ast }}{I_{R}}-l_{5} \frac{ ( I_{T}-I_{T}^{\ast } ) ^{2}}{I_{T}}+\mu I_{A}-\mu I_{A}^{\ast } \\ &{}-\mu I_{A}\frac{I_{T}^{\ast }}{I_{T}}+\mu I_{A}^{\ast } \frac{I_{T}^{\ast }}{I_{T}}+vI_{R}-vI_{R}^{\ast }-vI_{R} \frac{I_{T}^{\ast }}{I_{T}}+vI_{R}^{\ast } \frac{I_{T}^{\ast }}{I_{T}}-l_{6} \frac{ ( R-R^{\ast } ) ^{2}}{R}+\lambda I-\lambda I^{\ast } \\ &{}-\lambda I\frac{R^{\ast }}{R}+\lambda I^{\ast }\frac{R^{\ast }}{R}+ \varphi I_{D}-\varphi I_{D}^{\ast }-\varphi I_{D}\frac{R^{\ast }}{R}+ \varphi I_{D}^{\ast } \frac{R^{\ast }}{R}+\chi I_{A}-\chi I_{A}^{\ast } \\ &{}-\chi I_{A}\frac{R^{\ast }}{R}+\chi I_{A}^{\ast } \frac{R^{\ast }}{R}+\xi I_{R}-\xi I_{R}^{\ast }- \xi I_{R} \frac{R^{\ast }}{R}+\xi I_{R}^{\ast } \frac{R^{\ast }}{R}+\sigma I_{T}-\sigma I_{T}^{\ast } \\ &{}-\sigma I_{T}\frac{R^{\ast }}{R}+\sigma I_{T}^{\ast } \frac{R^{\ast }}{R}+\tau I_{T}-\tau I_{T}^{\ast }-\tau I_{T} \frac{D^{\ast }}{D}+\tau I_{T}^{\ast } \frac{D^{\ast }}{D} \\ &{}-\mu _{1}\frac{ ( V-V^{\ast } ) ^{2}}{V}-\gamma _{1}S- \gamma _{1}S^{\ast }-\gamma _{1}S\frac{V^{\ast }}{V}+ \gamma _{1}S^{ \ast }\frac{V^{\ast }}{V}+\Phi R-\Phi R^{\ast } \\ &{}-\Phi R\frac{V^{\ast }}{V}+\Phi R^{\ast }\frac{V^{\ast }}{V}. \end{aligned}$$

This can be simplified as

3.66 $$ \frac{dL}{dt}=\Pi -\Gamma, $$

where

3.67 $$\begin{aligned} \Pi = {}&\Lambda +\alpha ( x ) S+\alpha ( x ) \frac{I^{\ast }}{I}S^{\ast }+ \xi I+\xi \frac{I_{A}^{\ast }}{I_{A}}I^{\ast }+ \varepsilon I+\varepsilon \frac{I_{D}^{\ast }}{I_{D}}I^{\ast }+\eta I_{D} \\ &{}+\eta \frac{I_{R}^{\ast }}{I_{R}}+\theta I_{A}+\theta I_{A}^{\ast } \frac{I_{R}^{\ast }}{I_{R}}+\mu I_{A}+\mu I_{A}^{\ast } \frac{I_{T}^{\ast }}{I_{T}}+vI_{R}+vI_{R}^{\ast } \frac{I_{T}^{\ast }}{I_{T}}+\lambda I \\ &{}+\lambda I^{\ast }\frac{R^{\ast }}{R}+\varphi I_{D}+\varphi I_{D}^{ \ast }\frac{R^{\ast }}{R}+\chi I_{A}+\chi I_{A}^{\ast }\frac{R^{\ast }}{R}+ \xi I_{R}+\xi I_{R}^{\ast }\frac{R^{\ast }}{R} \\ &{}+\sigma I_{T}+\sigma I_{T}^{\ast } \frac{R^{\ast }}{R}+\tau I_{T}+\tau I_{T}^{\ast }\frac{D^{\ast }}{D}+\gamma _{1}S+ \gamma _{1}S^{\ast }\frac{V^{\ast }}{V}+\Phi R^{\ast } \frac{V^{\ast }}{V} \\ &{}+\Phi R \end{aligned}$$

and

3.68 $$\begin{aligned} \Gamma ={}& \frac{ ( S-S^{\ast } ) ^{2}}{S} \bigl( \alpha ( x ) +\gamma _{1}+\mu _{1} \bigr) +\frac{S^{\ast }}{S} \Lambda +l_{1}\frac{ ( I-I^{\ast } ) ^{2}}{I}+\alpha ( x ) S^{ \ast } \\ &{}+\alpha ( x ) \frac{I^{\ast }}{I}S+l_{2} \frac{ ( I_{A}-I_{A}^{\ast } ) ^{2}}{I_{A}}+\xi I^{\ast }+\xi \frac{I_{A}^{\ast }}{I_{A}}I+l_{3} \frac{ ( I_{D}-I_{D}^{\ast } ) ^{2}}{I_{D}}+ \varepsilon I^{\ast } \\ &{}+\varepsilon \frac{I_{D}^{\ast }}{I_{D}}I+l_{4} \frac{ ( I_{R}-I_{R}^{\ast } ) ^{2}}{I_{R}}+\eta I_{D}^{\ast }+\eta I_{D} \frac{I_{R}^{\ast }}{I_{R}}+ \theta I_{A}^{\ast }+\theta I_{A} \frac{I_{R}^{\ast }}{I_{R}}+\mu I_{A}^{\ast } \\ &{}+\mu I_{A}\frac{I_{T}^{\ast }}{I_{T}}+vI_{R}^{\ast }+vI_{R} \frac{I_{T}^{\ast }}{I_{T}}+l_{6}\frac{ ( R-R^{\ast } ) ^{2}}{R}+ \lambda I^{\ast }+l_{5} \frac{ ( I_{T}-I_{T}^{\ast } ) ^{2}}{I_{T}} \\ &{}+\lambda I\frac{R^{\ast }}{R}+\varphi I_{D}^{\ast }+\varphi I_{D} \frac{R^{\ast }}{R}+\chi I_{A}^{\ast }+\chi I_{A}\frac{R^{\ast }}{R}+\xi I_{R}^{ \ast }+\xi I_{R}\frac{R^{\ast }}{R}+\sigma I_{T}^{\ast } \\ &{}+\sigma I_{T}\frac{R^{\ast }}{R}+\tau I_{T}^{\ast }+ \tau I_{T}\frac{D^{\ast }}{D}+\mu _{1} \frac{ ( V-V^{\ast } ) ^{2}}{V} \\ &{}+\gamma _{1}S^{\ast }+\gamma _{1}S \frac{V^{\ast }}{V}+\Phi R^{\ast }+ \Phi R\frac{V^{\ast }}{V}. \end{aligned}$$

Therefore, having $\Pi <\Gamma $, this implies $\frac{dL}{dt}<0$, however,

3.69 $$ 0=\Pi -\Gamma \quad\Rightarrow\quad \frac{dL}{dt}=0 $$

if

3.70 $$ \begin{aligned} &S=S^{\ast },\quad I=I^{\ast },\quad I_{A}=I_{A}^{\ast },\quad I_{D}=I_{D}^{\ast },\quad I_{R}=I_{R}^{ \ast },\quad I_{T}=I_{T}^{\ast },\\ & R=R^{\ast },\quad D=D^{\ast }\quad \text{and}\quad V=V^{\ast }. \end{aligned} $$

We can now conclude that the largest compact invariant set for Covid-19 model in

3.71 $$ \biggl\{ \bigl( S^{\ast },I^{\ast },I_{A}^{\ast },I_{D}^{\ast },I_{R}^{ \ast },I_{T}^{\ast },R^{\ast },D^{\ast },V^{\ast } \bigr) \in \Omega:\frac{dL}{dt}=0 \biggr\} $$

is the point $\{ E_{\ast } \} $, the endemic equilibrium of the Covid-19 model. Therefore, using the Lasalle’s invariance principle, we conclude that $E_{\ast }$ is globally asymptotically stable in Ω if $\Pi <\Gamma $. □

## Modeling with nonlocal operators

Due to complexities around the spread of Covid-19, it is really hard to produce predictions, especially when multi-scenarios are requested. Indeed, it has been reported that including local operators cannot provide nonlocal processes, for example, change in processes. In this section, we present an analysis of Covid-19 model with local operators including Caputo, Caputo–Fabrizio, Atangana–Baleanu, and the new introduced fractal-fractional operators. We first present the definition of each operator. We start with the definition of the Caputo fractional derivative

4.1 $$ _{0}^{C}D_{t}^{\alpha }f ( t ) = \frac{1}{\Gamma ( 1-\alpha ) } \int _{0}^{t} \frac{d}{d\tau }f ( \tau ) ( t- \tau ) ^{- \alpha }\,d\tau. $$

The Caputo–Fabrizio fractional derivative is

4.2 $$ _{0}^{CF}D_{t}^{\alpha }f ( t ) = \frac{M ( \alpha ) }{1-\alpha } \int _{0}^{t}\frac{d}{d\tau }f ( \tau ) \exp \biggl[ -\frac{\alpha }{1-\alpha } ( t-\tau ) \biggr] \,d\tau. $$

The Atangana–Baleanu fractional derivative is

4.3 $$ _{0}^{ABC}D_{t}^{\alpha }f ( t ) = \frac{AB ( \alpha ) }{1-\alpha } \int _{0}^{t}\frac{d}{d\tau }f ( \tau ) E_{ \alpha } \biggl[ -\frac{\alpha }{1-\alpha } ( t-\tau ) ^{ \alpha } \biggr] \,d\tau. $$

The fractal-fractional derivative with a power-law kernel is

4.4 $$ _{0}^{FFP}D_{t}^{\alpha,\beta }f ( t ) = \frac{1}{\Gamma ( 1-\alpha ) }\frac{^{AG}d}{dt^{\beta }} \int _{0}^{t}f ( \tau ) ( t-\tau ) ^{-\alpha } \,d\tau, $$

where

4.5 $$ \frac{df ( t ) }{dt^{\beta }}=\lim_{t\rightarrow t_{1}} \frac{f ( t ) -f ( t_{1} ) }{t^{2-\beta }-t_{1}^{2-\beta }} ( 2-\beta ). $$

The fractal-fractional derivative with an exponential decay kernel is

4.6 $$ _{0}^{FFE}D_{t}^{\alpha,\beta }f ( t ) = \frac{M ( \alpha ) }{1-\alpha }\frac{^{AG}d}{dt^{\beta }} \int _{0}^{t}f ( \tau ) \exp \biggl[ - \frac{\alpha }{1-\alpha } ( t-\tau ) \biggr] \,d\tau. $$

The fractal-fractional derivative with a Mittag-Leffler kernel is

4.7 $$ _{0}^{FFM}D_{t}^{\alpha,\beta }f ( t ) = \frac{AB ( \alpha ) }{1-\alpha } \frac{^{AG}d}{dt^{\beta }} \int _{0}^{t}f ( \tau ) E_{ \alpha } \biggl[ - \frac{\alpha }{1-\alpha } ( t-\tau ) ^{ \alpha } \biggr] \,d\tau. $$

The associated integral operators of the last three operators are given as

4.8 $$\begin{aligned} &{}_{0}^{FFP}J_{t}^{\alpha,\beta }f ( t ) = \frac{1}{\Gamma ( \alpha ) } \int _{0}^{t} ( t-\tau ) ^{\alpha -1} \tau ^{1-\beta }f ( \tau ) \,d\tau, \\ &{}_{0}^{FFE}J_{t}^{\alpha,\beta }f ( t ) = \frac{1-\alpha }{M ( \alpha ) }t^{1-\beta }f ( t ) + \frac{\alpha }{M ( \alpha ) } \int _{0}^{t}\tau ^{1-\beta }f ( \tau ) \,d \tau, \\ &{}_{0}^{FFM}J_{t}^{\alpha,\beta }f ( t ) = \frac{1-\alpha }{AB ( \alpha ) }t^{1-\beta }f ( t ) + \frac{\alpha }{AB ( \alpha ) \Gamma ( \alpha ) } \int _{0}^{t} ( t-\tau ) ^{\alpha -1}\tau ^{1-\beta }f ( \tau ) \,d\tau. \end{aligned}$$

### Positive solutions with nonlocal operators

In this subsection, we present a detailed analysis of positiveness of the solutions for Covid-19 model with nonlocal operators. We start with ABC derivative case:

4.9 $$\begin{aligned} &{}_{0}^{ABC}D_{t}^{\alpha }S =\Lambda - \bigl( \alpha ( x ) +\gamma _{1}+\mu _{1} \bigr) S, \\ &{}_{0}^{ABC}D_{t}^{\alpha }I=\alpha ( x ) S- ( \varepsilon +\xi +\lambda +\mu _{1} ) I, \\ &{}_{0}^{ABC}D_{t}^{\alpha }I_{A} =\xi I- ( \theta +\mu + \chi +\mu _{1} ) I_{A}, \\ &{}_{0}^{ABC}D_{t}^{\alpha }I_{D} =\varepsilon I- ( \eta + \varphi +\mu _{1} ) I_{D}, \\ &{}_{0}^{ABC}D_{t}^{\alpha }I_{R} =\eta I_{D}+\theta I_{A}- ( v+\xi +\mu _{1} ) I_{R}, \\ &{}_{0}^{ABC}D_{t}^{\alpha }I_{T} =\mu I_{A}+vI_{R}- ( \sigma +\tau +\mu _{1} ) I_{T}, \\ &{}_{0}^{ABC}D_{t}^{\alpha }R=\lambda I+\varphi I_{D}+ \chi I_{A}+\xi I_{R}+\sigma I_{T}- ( \Phi +\mu _{1} ) R, \\ &{}_{0}^{ABC}D_{t}^{\alpha }D=\tau I_{T}, \\ &{}_{0}^{ABC}D_{t}^{\alpha }V=\gamma _{1}S+\Phi R-\mu _{1}V. \end{aligned}$$

The norm and all hypotheses of the classical results are valid here also

4.10 $$\begin{aligned} _{0}^{ABC}D_{t}^{\alpha }I&= \alpha ( x ) S- ( \varepsilon +\xi +\lambda +\mu _{1} ) I \\ &\geq - ( \varepsilon +\xi +\lambda +\mu _{1} ) I. \end{aligned}$$

This produces

4.11 $$\begin{aligned} &I ( t ) \geq I ( 0 ) E_{\alpha } \biggl[ - \frac{\alpha ( \varepsilon +\xi +\lambda +\mu _{1} ) t^{\alpha }}{AB ( \alpha ) - ( 1-\alpha ) ( \varepsilon +\xi +\lambda +\mu _{1} ) } \biggr], \\ &S ( t ) \geq S ( 0 ) E_{\alpha } \biggl[ - \frac{\alpha ( \Vert \alpha ( x ) \Vert _{\infty }+\gamma _{1}+\mu _{1} ) t^{\alpha }}{AB ( \alpha ) - ( 1-\alpha ) ( \Vert \alpha ( x ) \Vert _{\infty }+\gamma _{1}+\mu _{1} ) } \biggr], \\ &I_{A} ( t ) \geq I_{A} ( 0 ) E_{\alpha } \biggl[ - \frac{\alpha ( \theta +\mu +\chi +\mu _{1} ) t^{\alpha }}{AB ( \alpha ) - ( 1-\alpha ) ( \theta +\mu +\chi +\mu _{1} ) } \biggr], \\ &I_{D} ( t ) \geq I_{D} ( 0 ) E_{\alpha } \biggl[ - \frac{\alpha ( \eta +\varphi +\mu _{1} ) t^{\alpha }}{AB ( \alpha ) - ( 1-\alpha ) ( \eta +\varphi +\mu _{1} ) } \biggr], \\ &I_{R} ( t ) \geq I_{R} ( 0 ) E_{\alpha } \biggl[ - \frac{\alpha ( v+\xi +\mu _{1} ) t^{\alpha }}{AB ( \alpha ) - ( 1-\alpha ) ( v+\xi +\mu _{1} ) } \biggr], \\ &I_{T} ( t ) \geq I_{T} ( 0 ) E_{\alpha } \biggl[ - \frac{\alpha ( \sigma +\tau +\mu _{1} ) t^{\alpha }}{AB ( \alpha ) - ( 1-\alpha ) ( \sigma +\tau +\mu _{1} ) } \biggr], \\ &R ( t ) \geq R ( 0 ) E_{\alpha } \biggl[ - \frac{\alpha ( \Phi +\mu _{1} ) t^{\alpha }}{AB ( \alpha ) - ( 1-\alpha ) ( \Phi +\mu _{1} ) } \biggr], \\ &D ( t ) \geq D ( 0 ) E_{\alpha } \biggl[ - \frac{\alpha \mu _{1}t^{\alpha }}{AB ( \alpha ) - ( 1-\alpha ) \mu _{1}} \biggr], \\ &V ( t ) \geq V ( 0 ) E_{\alpha } \biggl[ - \frac{\alpha \mu _{1}t^{\alpha }}{AB ( \alpha ) - ( 1-\alpha ) \mu _{1}} \biggr],\quad \forall t\geq 0. \end{aligned}$$

This shows that if all the initial conditions are positive then all solutions are positive when using the Atangana–Baleanu derivative. With Caputo–Fabrizio derivative, we have

4.12 $$\begin{aligned} &I ( t ) \geq I ( 0 ) \exp \biggl[ - \frac{\alpha ( \varepsilon +\xi +\lambda +\mu _{1} ) t}{M ( \alpha ) - ( 1-\alpha ) ( \varepsilon +\xi +\lambda +\mu _{1} ) } \biggr], \\ &S ( t ) \geq S ( 0 ) \exp \biggl[ - \frac{\alpha ( \Vert \alpha ( x ) \Vert _{\infty }+\gamma _{1}+\mu _{1} ) t}{M ( \alpha ) - ( 1-\alpha ) ( \Vert \alpha ( x ) \Vert _{\infty }+\gamma _{1}+\mu _{1} ) } \biggr], \\ &I_{A} ( t ) \geq I_{A} ( 0 ) \exp \biggl[ - \frac{\alpha ( \theta +\mu +\chi +\mu _{1} ) t}{M ( \alpha ) - ( 1-\alpha ) ( \theta +\mu +\chi +\mu _{1} ) } \biggr], \\ &I_{D} ( t ) \geq I_{D} ( 0 ) \exp \biggl[ - \frac{\alpha ( \eta +\varphi +\mu _{1} ) t}{M ( \alpha ) - ( 1-\alpha ) ( \eta +\varphi +\mu _{1} ) } \biggr], \\ &I_{R} ( t ) \geq I_{R} ( 0 ) \exp \biggl[ - \frac{\alpha ( v+\xi +\mu _{1} ) t}{M ( \alpha ) - ( 1-\alpha ) ( v+\xi +\mu _{1} ) } \biggr], \\ &I_{T} ( t ) \geq I_{T} ( 0 ) \exp \biggl[ - \frac{\alpha ( \sigma +\tau +\mu _{1} ) t}{M ( \alpha ) - ( 1-\alpha ) ( \sigma +\tau +\mu _{1} ) } \biggr], \\ &R ( t ) \geq R ( 0 ) \exp \biggl[ - \frac{\alpha ( \Phi +\mu _{1} ) t}{M ( \alpha ) - ( 1-\alpha ) ( \Phi +\mu _{1} ) } \biggr], \\ &D ( t ) \geq D ( 0 ) \exp \biggl[ - \frac{\alpha \mu _{1}t}{M ( \alpha ) - ( 1-\alpha ) \mu _{1}} \biggr], \\ &V ( t ) \geq V ( 0 ) \exp \biggl[ - \frac{\alpha \mu _{1}t}{M ( \alpha ) - ( 1-\alpha ) \mu _{1}} \biggr], \quad\forall t\geq 0. \end{aligned}$$

This shows that all solutions are positive if all the initial conditions are positive using the Caputo–Fabrizio derivative. With Caputo derivative, we have

4.13 $$\begin{aligned} &I ( t ) \geq I ( 0 ) E_{\alpha } \bigl[ - ( \varepsilon +\xi +\lambda + \mu _{1} ) t^{\alpha } \bigr], \\ &S ( t ) \geq S ( 0 ) E_{\alpha } \bigl[ - \bigl( \bigl\Vert \alpha ( x ) \bigr\Vert _{\infty }+ \gamma _{1}+\mu _{1} \bigr) t^{\alpha } \bigr], \\ &I_{A} ( t ) \geq I_{A} ( 0 ) E_{\alpha } \bigl[ - ( \theta +\mu +\chi +\mu _{1} ) t^{\alpha } \bigr], \\ &I_{D} ( t ) \geq I_{D} ( 0 ) E_{\alpha } \bigl[ - ( \eta +\varphi +\mu _{1} ) t^{\alpha } \bigr], \\ &I_{R} ( t ) \geq I_{R} ( 0 ) E_{\alpha } \bigl[ - ( v+\xi +\mu _{1} ) t^{\alpha } \bigr], \\ &I_{T} ( t ) \geq I_{T} ( 0 ) E_{\alpha } \bigl[ - ( \sigma +\tau +\mu _{1} ) t^{\alpha } \bigr], \\ &R ( t ) \geq R ( 0 ) E_{\alpha } \bigl[ - ( \Phi +\mu _{1} ) t^{\alpha } \bigr], \\ &D ( t ) \geq D ( 0 ) E_{\alpha } \bigl[ -\mu _{1}t^{ \alpha } \bigr], \\ &V ( t ) \geq V ( 0 ) E_{\alpha } \bigl[ -\mu _{1}t^{ \alpha } \bigr], \quad\forall t\geq 0. \end{aligned}$$

This shows that all solutions are positive if all the initial conditions are positive when using the Caputo derivative.

For fractal-fractional case, without loss of generality, we present the proof for the *I* class and the rest can be deduced similarly. We start with the power-law case:

4.14 $$\begin{aligned} _{0}^{FFP}D_{t}^{\alpha,\beta }I& = \alpha ( x ) S- ( \varepsilon +\xi +\lambda +\mu _{1} ) I \\ &\geq - ( \varepsilon +\xi +\lambda +\mu _{1} ) I, \quad\forall t\geq 0. \end{aligned}$$

and

4.15 $$\begin{aligned} _{0}^{RL}D_{t}^{\alpha,\beta }I&\geq -t^{1-\beta } ( \varepsilon +\xi +\lambda +\mu _{1} ) I \\ &\geq -b^{1-\beta } ( \varepsilon +\xi +\lambda +\mu _{1} ) I,\quad \forall t\geq 0. \end{aligned}$$

Thus, we have

4.16 $$\begin{aligned} &I ( t ) \geq I ( 0 ) E_{\alpha } \bigl[ -b^{1- \beta } ( \varepsilon + \xi +\lambda +\mu _{1} ) t^{\alpha } \bigr], \\ &S ( t ) \geq S ( 0 ) E_{\alpha } \bigl[ -b^{1- \beta } \bigl( \bigl\Vert \alpha ( x ) \bigr\Vert _{ \infty }+\gamma _{1}+\mu _{1} \bigr) t^{\alpha } \bigr], \\ &I_{A} ( t ) \geq I_{A} ( 0 ) E_{\alpha } \bigl[ -b^{1-\beta } ( \theta +\mu +\chi +\mu _{1} ) t^{ \alpha } \bigr], \\ &I_{D} ( t ) \geq I_{D} ( 0 ) E_{\alpha } \bigl[ -b^{1-\beta } ( \eta +\varphi +\mu _{1} ) t^{ \alpha } \bigr], \\ &I_{R} ( t ) \geq I_{R} ( 0 ) E_{\alpha } \bigl[ -b^{1-\beta } ( v+\xi +\mu _{1} ) t^{\alpha } \bigr], \\ &I_{T} ( t ) \geq I_{T} ( 0 ) E_{\alpha } \bigl[ -b^{1-\beta } ( \sigma +\tau +\mu _{1} ) t^{ \alpha } \bigr], \\ &R ( t ) \geq R ( 0 ) E_{\alpha } \bigl[ -b^{1- \beta } ( \Phi +\mu _{1} ) t^{\alpha } \bigr], \\ &D ( t ) \geq D ( 0 ) E_{\alpha } \bigl[ -b^{1- \beta }\mu _{1}t^{\alpha } \bigr], \\ &V ( t ) \geq V ( 0 ) E_{\alpha } \bigl[ -b^{1- \beta }\mu _{1}t^{\alpha } \bigr],\quad \forall t\geq 0. \end{aligned}$$

With the exponential kernel, we have

4.17 $$\begin{aligned} &I ( t ) \geq I ( 0 ) \exp \biggl[ - \frac{b^{1-\beta }\alpha ( \varepsilon +\xi +\lambda +\mu _{1} ) t}{M ( \alpha ) - ( 1-\alpha ) ( \varepsilon +\xi +\lambda +\mu _{1} ) } \biggr], \\ &S ( t ) \geq S ( 0 ) \exp \biggl[ - \frac{b^{1-\beta }\alpha ( \Vert \alpha ( x ) \Vert _{\infty }+\gamma _{1}+\mu _{1} ) t}{M ( \alpha ) - ( 1-\alpha ) ( \Vert \alpha ( x ) \Vert _{\infty }+\gamma _{1}+\mu _{1} ) } \biggr], \\ &I_{A} ( t ) \geq I_{A} ( 0 ) \exp \biggl[ - \frac{b^{1-\beta }\alpha ( \theta +\mu +\chi +\mu _{1} ) t}{M ( \alpha ) - ( 1-\alpha ) ( \theta +\mu +\chi +\mu _{1} ) } \biggr], \\ &I_{D} ( t ) \geq I_{D} ( 0 ) \exp \biggl[ - \frac{b^{1-\beta }\alpha ( \eta +\varphi +\mu _{1} ) t}{M ( \alpha ) - ( 1-\alpha ) ( \eta +\varphi +\mu _{1} ) } \biggr], \\ &I_{R} ( t ) \geq I_{R} ( 0 ) \exp \biggl[ - \frac{b^{1-\beta }\alpha ( v+\xi +\mu _{1} ) t}{M ( \alpha ) - ( 1-\alpha ) ( v+\xi +\mu _{1} ) } \biggr], \\ &I_{T} ( t ) \geq I_{T} ( 0 ) \exp \biggl[ - \frac{b^{1-\beta }\alpha ( \sigma +\tau +\mu _{1} ) t}{M ( \alpha ) - ( 1-\alpha ) ( \sigma +\tau +\mu _{1} ) } \biggr], \\ &R ( t ) \geq R ( 0 ) \exp \biggl[ - \frac{b^{1-\beta }\alpha ( \Phi +\mu _{1} ) t}{M ( \alpha ) - ( 1-\alpha ) ( \Phi +\mu _{1} ) } \biggr], \\ &D ( t ) \geq D ( 0 ) \exp \biggl[ - \frac{b^{1-\beta }\alpha \mu _{1}t}{M ( \alpha ) - ( 1-\alpha ) \mu _{1}} \biggr], \\ &V ( t ) \geq V ( 0 ) \exp \biggl[ - \frac{b^{1-\beta }\alpha \mu _{1}t}{M ( \alpha ) - ( 1-\alpha ) \mu _{1}} \biggr],\quad \forall t\geq 0. \end{aligned}$$

With the Mittag-Leffler kernel, we obtain

4.18 $$\begin{aligned} &I ( t ) \geq I ( 0 ) E_{\alpha } \biggl[ - \frac{b^{1-\beta }\alpha ( \varepsilon +\xi +\lambda +\mu _{1} ) t^{\alpha }}{AB ( \alpha ) - ( 1-\alpha ) ( \varepsilon +\xi +\lambda +\mu _{1} ) } \biggr], \\ &S ( t ) \geq S ( 0 ) E_{\alpha } \biggl[ - \frac{b^{1-\beta }\alpha ( \Vert \alpha ( x ) \Vert _{\infty }+\gamma _{1}+\mu _{1} ) t^{\alpha }}{AB ( \alpha ) - ( 1-\alpha ) ( \Vert \alpha ( x ) \Vert _{\infty }+\gamma _{1}+\mu _{1} ) } \biggr], \\ &I_{A} ( t ) \geq I_{A} ( 0 ) E_{\alpha } \biggl[ - \frac{b^{1-\beta }\alpha ( \theta +\mu +\chi +\mu _{1} ) t^{\alpha }}{AB ( \alpha ) - ( 1-\alpha ) ( \theta +\mu +\chi +\mu _{1} ) } \biggr], \\ &I_{D} ( t ) \geq I_{D} ( 0 ) E_{\alpha } \biggl[ - \frac{b^{1-\beta }\alpha ( \eta +\varphi +\mu _{1} ) t^{\alpha }}{AB ( \alpha ) - ( 1-\alpha ) ( \eta +\varphi +\mu _{1} ) } \biggr], \\ &I_{R} ( t ) \geq I_{R} ( 0 ) E_{\alpha } \biggl[ - \frac{b^{1-\beta }\alpha ( v+\xi +\mu _{1} ) t^{\alpha }}{AB ( \alpha ) - ( 1-\alpha ) ( v+\xi +\mu _{1} ) } \biggr], \\ &I_{T} ( t ) \geq I_{T} ( 0 ) E_{\alpha } \biggl[ - \frac{b^{1-\beta }\alpha ( \sigma +\tau +\mu _{1} ) t^{\alpha }}{AB ( \alpha ) - ( 1-\alpha ) ( \sigma +\tau +\mu _{1} ) } \biggr], \\ &R ( t ) \geq R ( 0 ) E_{\alpha } \biggl[ - \frac{b^{1-\beta }\alpha ( \Phi +\mu _{1} ) t^{\alpha }}{AB ( \alpha ) - ( 1-\alpha ) ( \Phi +\mu _{1} ) } \biggr], \\ &D ( t ) \geq D ( 0 ) E_{\alpha } \biggl[ - \frac{b^{1-\beta }\alpha \mu _{1}t^{\alpha }}{AB ( \alpha ) - ( 1-\alpha ) \mu _{1}} \biggr], \\ &V ( t ) \geq V ( 0 ) E_{\alpha } \biggl[ - \frac{b^{1-\beta }\alpha \mu _{1}t^{\alpha }}{AB ( \alpha ) - ( 1-\alpha ) \mu _{1}} \biggr],\quad \forall t\geq 0. \end{aligned}$$

## Numerical analysis of Covid-19 models from classical to nonlocal operators: application of Atangana–Seda numerical scheme

While analytical methods are adequate to provide the exact solution of a giving equation, or systems of equations, it is important to note that when dealing with nonlinear equations, analytical methods cannot be used. In particular, the model of Covid-19 suggested in this work either with classical or nonlocal operators contains nonlinear components and therefore analytical methods are ineffective. Very recently, Atangana and Seda [16] made use of Newton polynomial to introduce an alternative numerical scheme that can be used to solving nonlinear equations arising in many fields of science, technology, and engineering. The method has been recognized to be very efficient and accurate. In this section, we will make use of the Atangana–Seda scheme to solve the suggested mathematical model for Covid-19 for different differential operators.

We start with the classical case for numerical solution of Covid-19 model:

5.1 $$\begin{aligned} &\overset{\cdot }{S} = \Lambda - \bigl( \alpha ( x ) + \gamma _{1}+ \mu _{1} \bigr) S, \\ &\overset{\cdot }{I}= \alpha ( x ) S- ( \varepsilon +\xi +\lambda +\mu _{1} ) I, \\ &\overset{\cdot }{I_{A}}= \xi I- ( \theta +\mu +\chi + \mu _{1} ) I_{A}, \\ &\overset{\cdot }{I_{D}}= \varepsilon I- ( \eta + \varphi + \mu _{1} ) I_{D}, \\ &\overset{\cdot }{I_{R}}= \eta I_{D}+\theta I_{A}- ( v+ \xi +\mu _{1} ) I_{R}, \\ &\overset{\cdot }{I_{T}}= \mu I_{A}+vI_{R}- ( \sigma + \tau +\mu _{1} ) I_{T}, \\ &\overset{\cdot }{R}= \lambda I+\varphi I_{D}+\chi I_{A}+ \xi I_{R}+\sigma I_{T}- ( \Phi +\mu _{1} ) R, \\ &\overset{\cdot }{D}= \tau I_{T}, \\ &\overset{\cdot }{V}= \gamma _{1}S+\Phi R-\mu _{1}V. \end{aligned}$$

For simplicity, we write the above equations as follows:

5.2 $$\begin{aligned} &\overset{\cdot }{S} = \widetilde{S} ( t,S,I,I_{A},I_{D},I_{R},I_{T},R,D,V ), \\ &\overset{\cdot }{I}= \widetilde{I} ( t,S,I,I_{A},I_{D},I_{R},I_{T},R,D,V ), \\ &\overset{\cdot }{I_{A}} = \widetilde{I_{A}} ( t,S,I,I_{A},I_{D},I_{R},I_{T},R,D,V ), \\ &\overset{\cdot }{I_{D}}= \widetilde{I_{D}} ( t,S,I,I_{A},I_{D},I_{R},I_{T},R,D,V ), \\ &\overset{\cdot }{I_{R}}= \widetilde{I_{R}} ( t,S,I,I_{A},I_{D},I_{R},I_{T},R,D,V ), \\ &\overset{\cdot }{I_{T}}= \widetilde{I_{T}} ( t,S,I,I_{A},I_{D},I_{R},I_{T},R,D,V ), \\ &\overset{\cdot }{R}= \widetilde{R} ( t,S,I,I_{A},I_{D},I_{R},I_{T},R,D,V ), \\ &\overset{\cdot }{D}= \widetilde{D} ( t,S,I,I_{A},I_{D},I_{R},I_{T},R,D,V ), \\ &\overset{\cdot }{V}= \widetilde{V} ( t,S,I,I_{A},I_{D},I_{R},I_{T},R,D,V ), \end{aligned}$$

where

5.3 $$\begin{aligned} &\widetilde{S} ( t,S,I,I_{A},I_{D},I_{R},I_{T},R,D,V ) = \Lambda - \bigl( \alpha ( x ) +\gamma _{1}+\mu _{1} \bigr) S, \\ &\widetilde{I} ( t,S,I,I_{A},I_{D},I_{R},I_{T},R,D,V ) = \alpha ( x ) S- ( \varepsilon +\xi +\lambda +\mu _{1} ) I, \\ &\widetilde{I_{A}} ( t,S,I,I_{A},I_{D},I_{R},I_{T},R,D,V ) = \xi I- ( \theta +\mu +\chi +\mu _{1} ) I_{A}, \\ &\widetilde{I_{D}}( t,S,I,I_{A},I_{D},I_{R},I_{T},R,D,V ) = \varepsilon I- ( \eta +\varphi +\mu _{1} ) I_{D}, \\ &\widetilde{I_{R}} ( t,S,I,I_{A},I_{D},I_{R},I_{T},R,D,V ) = \eta I_{D}+\theta I_{A}- ( v+\xi +\mu _{1} ) I_{R}, \\ &\widetilde{I_{T}} ( t,S,I,I_{A},I_{D},I_{R},I_{T},R,D,V ) = \mu I_{A}+vI_{R}- ( \sigma +\tau +\mu _{1} ) I_{T} , \\ &\widetilde{R} ( t,S,I,I_{A},I_{D},I_{R},I_{T},R,D,V ) = \lambda I+\varphi I_{D}+\chi I_{A}+\xi I_{R}+\sigma I_{T}- ( \Phi +\mu _{1} ) R, \\ &\widetilde{D}( t,S,I,I_{A},I_{D},I_{R},I_{T},R,D,V ) = \tau I_{T}, \\ &\widetilde{V}( t,S,I,I_{A},I_{D},I_{R},I_{T},R,D,V ) = \gamma _{1}S+\Phi R-\mu _{1}V. \end{aligned}$$

After applying fractal-fractional integral with the exponential kernel, we have the following:

5.4 $$\begin{aligned} S ( t_{p+1} ) ={}& S ( t_{p} ) + \begin{bmatrix} \widetilde{S} ( t_{p},S^{p},I^{p},I_{A}^{p},I_{D}^{ p},I_{R}^{p},I_{T}^{p},R^{p},D^{p},V^{p} ) \\ -\widetilde{S} ( t_{p-1},S^{p-1},I^{p-1},I_{A}^{p-1},I_{D}^{p-1},I_{R}^{p-1},I_{T}^{p-1},R^{p-1},D^{p-1},V^{p-1} )\end{bmatrix} \\ &{}+ \int _{t_{p}}^{t_{p+1}}\widetilde{S} ( \tau,S,I,I_{A},I_{D},I_{R},I_{T},R,D,V ) \,d\tau, \\ I ( t_{p+1} ) ={}& I ( t_{p} ) + \begin{bmatrix} \widetilde{I} ( t_{p},S^{p},I^{p},I_{A}^{p},I_{D}^{ p},I_{R}^{p},I_{T}^{p},R^{p},D^{p},V^{p} ) \\ -\widetilde{I} ( t_{p-1},S^{p-1},I^{p-1},I_{A}^{p-1},I_{D}^{p-1},I_{R}^{p-1},I_{T}^{p-1},R^{p-1},D^{p-1},V^{p-1} )\end{bmatrix} \\ &{}+ \int _{t_{p}}^{t_{p+1}}\widetilde{I} ( \tau,S,I,I_{A},I_{D},I_{R},I_{T},R,D,V ) \,d\tau, \\ I_{A} ( t_{p+1} ) ={}& I_{A} ( t_{p} ) + \begin{bmatrix} \widetilde{I_{A}} ( t_{p},S^{p},I^{p},I_{A}^{p},I_{D}^{p},I_{R}^{p},I_{T}^{p},R^{p},D^{p},V^{p} ) \\ -\widetilde{I_{A}} ( t_{p-1},S^{p-1},I^{p-1},I_{A}^{ p-1},I_{D}^{p-1},I_{R}^{p-1},I_{T}^{p-1},R^{p-1},D^{p-1},V^{p-1} )\end{bmatrix} \\ &{}+ \int _{t_{p}}^{t_{p+1}}\widetilde{I_{A}} ( \tau,S,I,I_{A},I_{D},I_{R},I_{T},R,D,V ) \,d\tau, \\ I_{D} ( t_{p+1} ) = {}&I_{D} ( t_{p} ) + \begin{bmatrix} \widetilde{I_{D}} ( t_{p},S^{p},I^{p},I_{A}^{p},I_{D}^{ p},I_{R}^{p},I_{T}^{p},R^{p},D^{p},V^{p} ) \\ -\widetilde{I_{D}} ( t_{p-1},S^{p-1},I^{p-1},I_{A}^{p-1},I_{D}^{p-1},I_{R}^{p-1},I_{T}^{p-1},R^{p-1},D^{p-1},V^{p-1} )\end{bmatrix} \\ &{}+ \int _{t_{p}}^{t_{p+1}}\widetilde{I_{D}} ( \tau,S,I,I_{A},I_{D},I_{R},I_{T},R,D,V ) \,d\tau, \end{aligned}$$

$$\begin{aligned} I_{R} ( t_{p+1} ) = {}&I_{R} ( t_{p} ) + \begin{bmatrix} \widetilde{I_{R}} ( t_{p},S^{p},I^{p},I_{A}^{p},I_{D}^{ p},I_{R}^{p},I_{T}^{p},R^{p},D^{p},V^{p} ) \\ -\widetilde{I_{R}} ( t_{p-1},S^{p-1},I^{p-1},I_{A}^{p-1},I_{D}^{p-1},I_{R}^{p-1},I_{T}^{p-1},R^{p-1},D^{p-1},V^{p-1} )\end{bmatrix} \\ &{}+ \int _{t_{p}}^{t_{p+1}}\widetilde{I_{R}} ( \tau,S,I,I_{A},I_{D},I_{R},I_{T},R,D,V ) \,d\tau, \\ I_{T} ( t_{p+1} ) = {}&I_{T} ( t_{p} ) + \begin{bmatrix} \widetilde{I_{T}} ( t_{p},S^{p},I^{p},I_{A}^{p},I_{D}^{ p},I_{R}^{p},I_{T}^{p},R^{p},D^{p},V^{p} ) \\ -\widetilde{I_{T}} ( t_{p-1},S^{p-1},I^{p-1},I_{A}^{p-1},I_{D}^{p-1},I_{R}^{p-1},I_{T}^{p-1},R^{p-1},D^{p-1},V^{p-1} )\end{bmatrix} \\ &{}+ \int _{t_{p}}^{t_{p+1}}\widetilde{I_{T}} ( \tau,S,I,I_{A},I_{D},I_{R},I_{T},R,D,V ) \,d\tau, \\ R ( t_{p+1} ) = {}&R ( t_{p} ) + \begin{bmatrix} \widetilde{R} ( t_{p},S^{p},I^{p},I_{A}^{p},I_{D}^{ p},I_{R}^{p},I_{T}^{p},R^{p},D^{p},V^{p} ) \\ -\widetilde{R} ( t_{p-1},S^{p-1},I^{p-1},I_{A}^{p-1},I_{D}^{p-1},I_{R}^{p-1},I_{T}^{p-1},R^{p-1},D^{p-1},V^{p-1} )\end{bmatrix} \\ &{}+ \int _{t_{p}}^{t_{p+1}}\widetilde{R} ( \tau,S,I,I_{A},I_{D},I_{R},I_{T},R,D,V ) \,d\tau, \\ D ( t_{p+1} ) = {}&D ( t_{p} ) + \begin{bmatrix} \widetilde{D} ( t_{p},S^{p},I^{p},I_{A}^{p},I_{D}^{ p},I_{R}^{p},I_{T}^{p},R^{p},D^{p},V^{p} ) \\ -\widetilde{D} ( t_{p-1},S^{p-1},I^{p-1},I_{A}^{p-1},I_{D}^{p-1},I_{R}^{p-1},I_{T}^{p-1},R^{p-1},D^{p-1},V^{p-1} )\end{bmatrix} \\ &{}+ \int _{t_{p}}^{t_{p+1}}\widetilde{D} ( \tau,S,I,I_{A},I_{D},I_{R},I_{T},R,D,V ) \,d\tau, \\ V ( t_{p+1} ) ={}& V ( t_{p} ) + \begin{bmatrix} \widetilde{V} ( t_{p},S^{p},I^{p},I_{A}^{p},I_{D}^{ p},I_{R}^{p},I_{T}^{p},R^{p},D^{p},V^{p} ) \\ -\widetilde{V} ( t_{p-1},S^{p-1},I^{p-1},I_{A}^{p-1},I_{D}^{p-1},I_{R}^{p-1},I_{T}^{p-1},R^{p-1},D^{p-1},V^{p-1} )\end{bmatrix} \\ &{}+ \int _{t_{p}}^{t_{p+1}}\widetilde{V} ( \tau,S,I,I_{A},I_{D},I_{R},I_{T},R,D,V ) \,d\tau. \end{aligned}$$

We can have the following scheme for this model:

$$\begin{array}{rl} S^{p+1}= & S^{p}+\frac{1-\alpha }{M(\alpha )}\left[\begin{array}{c} \tilde{S}(t_{p},S^{p},I^{p},I_{A}^{p},I_{D}^{p},I_{R}^{p},I_{T}^{p},R^{p},D^{p},V^{p})\\ -\tilde{S}(t_{p-1},S^{p-1},I^{p-1},I_{A}^{p-1},I_{D}^{p-1},I_{R}^{p-1},I_{T}^{p-1},R^{p-1},D^{p-1},V^{p-1}) \end{array}\right]\\ & +\frac{\alpha }{M(\alpha )}\left\{\begin{array}{c} \frac{23}{12}\tilde{S}(t_{p},S^{p},I^{p},I_{A}^{p},I_{D}^{p},I_{R}^{p},I_{T}^{p},R^{p},D^{p},V^{p})\Delta t\\ -\frac{4}{3}\tilde{S}(t_{p-1},S^{p-1},I^{p-1},I_{A}^{p-1},I_{D}^{p-1},I_{R}^{p-1},I_{T}^{p-1},R^{p-1},D^{p-1},V^{p-1})\Delta t\\ +\frac{5}{12}\tilde{S}(t_{p-2},S^{p-2},I^{p-2},I_{A}^{p-2},I_{D}^{p-2},I_{R}^{p-2},I_{T}^{p-2},R^{p-2},D^{p-2},V^{p-2})\Delta t \end{array}\right\},\\ I^{p+1}= & I^{p}+\frac{1-\alpha }{M(\alpha )}\left[\begin{array}{c} \tilde{I}(t_{p},S^{p},I^{p},I_{A}^{p},I_{D}^{p},I_{R}^{p},I_{T}^{p},R^{p},D^{p},V^{p})\\ -\tilde{I}(t_{p-1},S^{p-1},I^{p-1},I_{A}^{p-1},I_{D}^{p-1},I_{R}^{p-1},I_{T}^{p-1},R^{p-1},D^{p-1},V^{p-1}) \end{array}\right]\\ & +\frac{\alpha }{M(\alpha )}\left\{\begin{array}{c} \frac{23}{12}\tilde{I}(t_{p},S^{p},I^{p},I_{A}^{p},I_{D}^{p},I_{R}^{p},I_{T}^{p},R^{p},D^{p},V^{p})\Delta t\\ -\frac{4}{3}\tilde{I}(t_{p-1},S^{p-1},I^{p-1},I_{A}^{p-1},I_{D}^{p-1},I_{R}^{p-1},I_{T}^{p-1},R^{p-1},D^{p-1},V^{p-1})\Delta t\\ +\frac{5}{12}\tilde{I}(t_{p-2},S^{p-2},I^{p-2},I_{A}^{p-2},I_{D}^{p-2},I_{R}^{p-2},I_{T}^{p-2},R^{p-2},D^{p-2},V^{p-2})\Delta t \end{array}\right\},\\ I_{A}^{p+1}= & I_{A}^{p}+\frac{1-\alpha }{M(\alpha )}\left[\begin{array}{c} \tilde{I_{A}}(t_{p},S^{p},I^{p},I_{A}^{p},I_{D}^{p},I_{R}^{p},I_{T}^{p},R^{p},D^{p},V^{p})\\ -\tilde{I_{A}}(t_{p-1},S^{p-1},I^{p-1},I_{A}^{p-1},I_{D}^{p-1},I_{R}^{p-1},I_{T}^{p-1},R^{p-1},D^{p-1},V^{p-1}) \end{array}\right]\\ & +\frac{\alpha }{M(\alpha )}\left\{\begin{array}{c} \frac{23}{12}\tilde{I_{A}}(t_{p},S^{p},I^{p},I_{A}^{p},I_{D}^{p},I_{R}^{p},I_{T}^{p},R^{p},D^{p},V^{p})\Delta t\\ -\frac{4}{3}\tilde{I_{A}}(t_{p-1},S^{p-1},I^{p-1},I_{A}^{p-1},I_{D}^{p-1},I_{R}^{p-1},I_{T}^{p-1},R^{p-1},D^{p-1},V^{p-1})\Delta t\\ +\frac{5}{12}\tilde{I_{A}}(t_{p-2},S^{p-2},I^{p-2},I_{A}^{p-2},I_{D}^{p-2},I_{R}^{p-2},I_{T}^{p-2},R^{p-2},D^{p-2},V^{p-2})\Delta t \end{array}\right\},\\ I_{D}^{p+1}= & I_{D}^{p}+\frac{1-\alpha }{M(\alpha )}\left[\begin{array}{c} \tilde{I_{D}}(t_{p},S^{p},I^{p},I_{A}^{p},I_{D}^{p},I_{R}^{p},I_{T}^{p},R^{p},D^{p},V^{p})\\ -\tilde{I_{D}}(t_{p-1},S^{p-1},I^{p-1},I_{A}^{p-1},I_{D}^{p-1},I_{R}^{p-1},I_{T}^{p-1},R^{p-1},D^{p-1},V^{p-1}) \end{array}\right]\\ & +\frac{\alpha }{M(\alpha )}\left\{\begin{array}{c} \frac{23}{12}\tilde{I_{D}}(t_{p},S^{p},I^{p},I_{A}^{p},I_{D}^{p},I_{R}^{p},I_{T}^{p},R^{p},D^{p},V^{p})\Delta t\\ -\frac{4}{3}\tilde{I_{D}}(t_{p-1},S^{p-1},I^{p-1},I_{A}^{p-1},I_{D}^{p-1},I_{R}^{p-1},I_{T}^{p-1},R^{p-1},D^{p-1},V^{p-1})\Delta t\\ +\frac{5}{12}\tilde{I_{D}}(t_{p-2},S^{p-2},I^{p-2},I_{A}^{p-2},I_{D}^{p-2},I_{R}^{p-2},I_{T}^{p-2},R^{p-2},D^{p-2},V^{p-2})\Delta t \end{array}\right\}, \end{array}$$

5.5 $$\begin{array}{rl} I_{R}^{p+1}= & I_{R}^{p}+\frac{1-\alpha }{M(\alpha )}\left[\begin{array}{c} \tilde{I_{R}}(t_{p},S^{p},I^{p},I_{A}^{p},I_{D}^{p},I_{R}^{p},I_{T}^{p},R^{p},D^{p},V^{p})\\ -\tilde{I_{R}}(t_{p-1},S^{p-1},I^{p-1},I_{A}^{p-1},I_{D}^{p-1},I_{R}^{p-1},I_{T}^{p-1},R^{p-1},D^{p-1},V^{p-1}) \end{array}\right]\\ & +\frac{\alpha }{M(\alpha )}\left\{\begin{array}{c} \frac{23}{12}\tilde{I_{R}}(t_{p},S^{p},I^{p},I_{A}^{p},I_{D}^{p},I_{R}^{p},I_{T}^{p},R^{p},D^{p},V^{p})\Delta t\\ -\frac{4}{3}\tilde{I_{R}}(t_{p-1},S^{p-1},I^{p-1},I_{A}^{p-1},I_{D}^{p-1},I_{R}^{p-1},I_{T}^{p-1},R^{p-1},D^{p-1},V^{p-1})\Delta t\\ +\frac{5}{12}\tilde{I_{R}}(t_{p-2},S^{p-2},I^{p-2},I_{A}^{p-2},I_{D}^{p-2},I_{R}^{p-2},I_{T}^{p-2},R^{p-2},D^{p-2},V^{p-2})\Delta t \end{array}\right\} \end{array}$$

$$\begin{array}{rl} I_{T}^{p+1}= & I_{T}^{p}+\frac{1-\alpha }{M(\alpha )}\left[\begin{array}{c} \tilde{I_{T}}(t_{p},S^{p},I^{p},I_{A}^{p},I_{D}^{p},I_{R}^{p},I_{T}^{p},R^{p},D^{p},V^{p})\\ -\tilde{I_{T}}(t_{p-1},S^{p-1},I^{p-1},I_{A}^{p-1},I_{D}^{p-1},I_{R}^{p-1},I_{T}^{p-1},R^{p-1},D^{p-1},V^{p-1}) \end{array}\right]\\ & +\frac{\alpha }{M(\alpha )}\left\{\begin{array}{c} \frac{23}{12}\tilde{I_{T}}(t_{p},S^{p},I^{p},I_{A}^{p},I_{D}^{p},I_{R}^{p},I_{T}^{p},R^{p},D^{p},V^{p})\Delta t\\ -\frac{4}{3}\tilde{I_{T}}(t_{p-1},S^{p-1},I^{p-1},I_{A}^{p-1},I_{D}^{p-1},I_{R}^{p-1},I_{T}^{p-1},R^{p-1},D^{p-1},V^{p-1})\Delta t\\ +\frac{5}{12}\tilde{I_{T}}(t_{p-2},S^{p-2},I^{p-2},I_{A}^{p-2},I_{D}^{p-2},I_{R}^{p-2},I_{T}^{p-2},R^{p-2},D^{p-2},V^{p-2})\Delta t \end{array}\right\},\\ R^{p+1}= & R^{p}+\frac{1-\alpha }{M(\alpha )}\left[\begin{array}{c} \tilde{R}(t_{p},S^{p},I^{p},I_{A}^{p},I_{D}^{p},I_{R}^{p},I_{T}^{p},R^{p},D^{p},V^{p})\\ -\tilde{R}(t_{p-1},S^{p-1},I^{p-1},I_{A}^{p-1},I_{D}^{p-1},I_{R}^{p-1},I_{T}^{p-1},R^{p-1},D^{p-1},V^{p-1}) \end{array}\right]\\ & +\frac{\alpha }{M(\alpha )}\left\{\begin{array}{c} \frac{23}{12}\tilde{R}(t_{p},S^{p},I^{p},I_{A}^{p},I_{D}^{p},I_{R}^{p},I_{T}^{p},R^{p},D^{p},V^{p})\Delta t\\ -\frac{4}{3}\tilde{R}(t_{p-1},S^{p-1},I^{p-1},I_{A}^{p-1},I_{D}^{p-1},I_{R}^{p-1},I_{T}^{p-1},R^{p-1},D^{p-1},V^{p-1})\Delta t\\ +\frac{5}{12}\tilde{R}(t_{p-2},S^{p-2},I^{p-2},I_{A}^{p-2},I_{D}^{p-2},I_{R}^{p-2},I_{T}^{p-2},R^{p-2},D^{p-2},V^{p-2})\Delta t \end{array}\right\},\\ D^{p+1}= & D^{p}+\frac{1-\alpha }{M(\alpha )}\left[\begin{array}{c} \tilde{D}(t_{p},S^{p},I^{p},I_{A}^{p},I_{D}^{p},I_{R}^{p},I_{T}^{p},R^{p},D^{p},V^{p})\\ -\tilde{D}(t_{p-1},S^{p-1},I^{p-1},I_{A}^{p-1},I_{D}^{p-1},I_{R}^{p-1},I_{T}^{p-1},R^{p-1},D^{p-1},V^{p-1}) \end{array}\right]\\ & +\frac{\alpha }{M(\alpha )}\left\{\begin{array}{c} \frac{23}{12}\tilde{D}(t_{p},S^{p},I^{p},I_{A}^{p},I_{D}^{p},I_{R}^{p},I_{T}^{p},R^{p},D^{p},V^{p})\Delta t\\ -\frac{4}{3}\tilde{D}(t_{p-1},S^{p-1},I^{p-1},I_{A}^{p-1},I_{D}^{p-1},I_{R}^{p-1},I_{T}^{p-1},R^{p-1},D^{p-1},V^{p-1})\Delta t\\ +\frac{5}{12}\tilde{D}(t_{p-2},S^{p-2},I^{p-2},I_{A}^{p-2},I_{D}^{p-2},I_{R}^{p-2},I_{T}^{p-2},R^{p-2},D^{p-2},V^{p-2})\Delta t \end{array}\right\},\\ V^{p+1}= & V^{p}+\frac{1-\alpha }{M(\alpha )}\left[\begin{array}{c} \tilde{V}(t_{p},S^{p},I^{p},I_{A}^{p},I_{D}^{p},I_{R}^{p},I_{T}^{p},R^{p},D^{p},V^{p})\\ -\tilde{V}(t_{p-1},S^{p-1},I^{p-1},I_{A}^{p-1},I_{D}^{p-1},I_{R}^{p-1},I_{T}^{p-1},R^{p-1},D^{p-1},V^{p-1}) \end{array}\right]\\ & +\frac{\alpha }{M(\alpha )}\left\{\begin{array}{c} \frac{23}{12}\tilde{V}(t_{p},S^{p},I^{p},I_{A}^{p},I_{D}^{p},I_{R}^{p},I_{T}^{p},R^{p},D^{p},V^{p})\Delta t\\ -\frac{4}{3}\tilde{V}(t_{p-1},S^{p-1},I^{p-1},I_{A}^{p-1},I_{D}^{p-1},I_{R}^{p-1},I_{T}^{p-1},R^{p-1},D^{p-1},V^{p-1})\Delta t\\ +\frac{5}{12}\tilde{V}(t_{p-2},S^{p-2},I^{p-2},I_{A}^{p-2},I_{D}^{p-2},I_{R}^{p-2},I_{T}^{p-2},R^{p-2},D^{p-2},V^{p-2})\Delta t \end{array}\right\}. \end{array}$$

Now, we handle the following model with classical derivative:

5.6 $$\begin{aligned} &\overset{\cdot }{S} = \Lambda - \bigl( \alpha ( x ) + \gamma _{1}+ \mu _{1} \bigr) S, \\ &\overset{\cdot }{I} = \alpha ( x ) S- ( \varepsilon +\xi +\lambda +\mu _{1} ) I, \\ &\overset{\cdot }{I_{A}} = \xi I- ( \theta +\mu +\chi +\mu _{1} ) I_{A}, \\ &\overset{\cdot }{I_{D}} = \varepsilon I- ( \eta +\varphi +\mu _{1} ) I_{D}, \\ &\overset{\cdot }{I_{R}} = \eta I_{D}+\theta I_{A}- ( v+\xi + \mu _{1} ) I_{R}, \\ &\overset{\cdot }{I_{T}} = \mu I_{A}+vI_{R}- ( \sigma +\tau + \mu _{1} ) I_{T}, \\ &\overset{\cdot }{R} = \lambda I+\varphi I_{D}+\chi I_{A}+\xi I_{R}+ \sigma I_{T}- ( \Phi +\mu _{1} ) R, \\ &\overset{\cdot }{D} = \tau I_{T}, \\ &\overset{\cdot }{V} = \gamma _{1}S+\Phi R-\mu _{1}V, \end{aligned}$$

where initial conditions are

5.7 $$\begin{aligned} &S ( 0 ) = 57780000,\qquad I ( 0 ) =1,\qquad I_{A} ( 0 ) =1,\qquad I_{D} ( 0 ) =1,\qquad I_{R} ( 0 ) =1, \\ &I_{T} ( 0 ) = 1,\qquad R ( 0 ) =0,\qquad D ( 0 ) =0,\qquad V ( 0 ) =0. \end{aligned}$$

Also the parameters are chosen as follows:

5.8 $$\begin{aligned} \begin{aligned} &\Lambda = 57000000,\qquad k=3,\qquad p=0.5,\qquad \eta =0.12,\qquad \chi =0.015,\\ & v=0.027,\qquad x=0.4, \qquad\theta = 0.301,\qquad \gamma =0.09,\qquad \beta =0.013,\\ & \lambda =0.0345,\qquad \varphi =0.0345, \qquad \delta _{1}=0.01,\qquad \gamma _{1} = 0.4,\qquad\mu _{1}=0.3,\\ &\varepsilon =0.161,\qquad\xi =0.015,\qquad\sigma =0.015,\qquad \tau =0.0199,\qquad\Phi =0.2. \end{aligned} \end{aligned}$$

**Figure 33:**
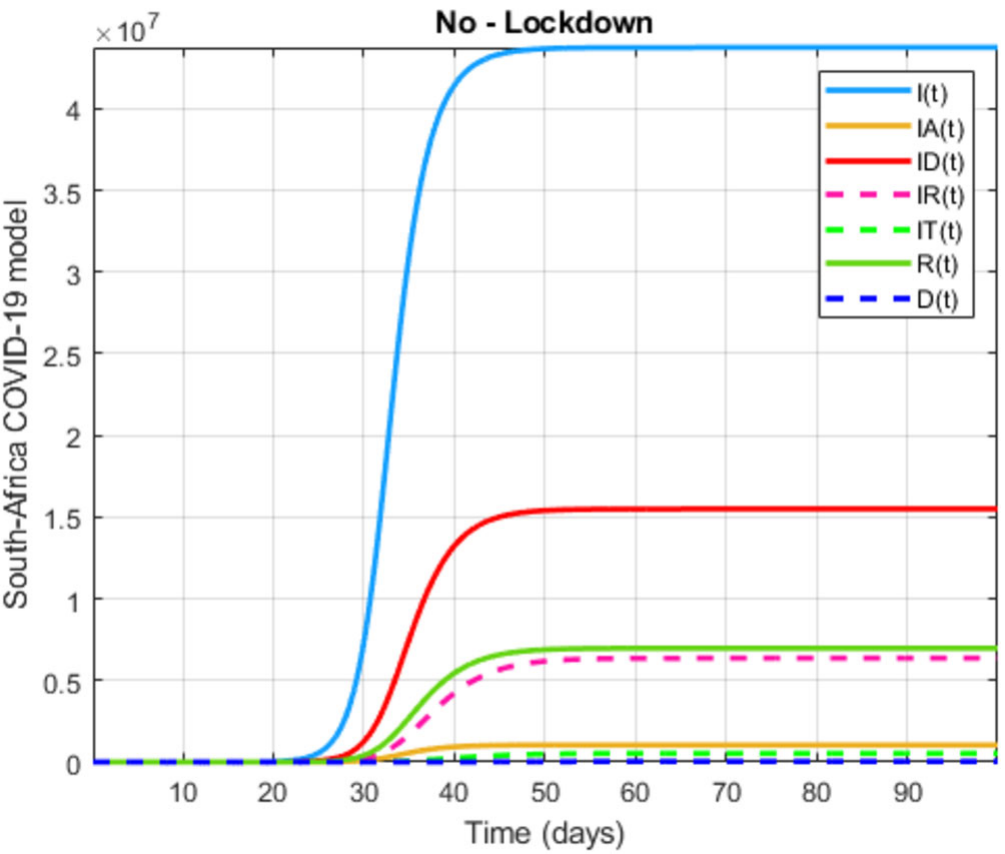
Numerical visualization for Covid-19 model in South Africa

**Figure 34:**
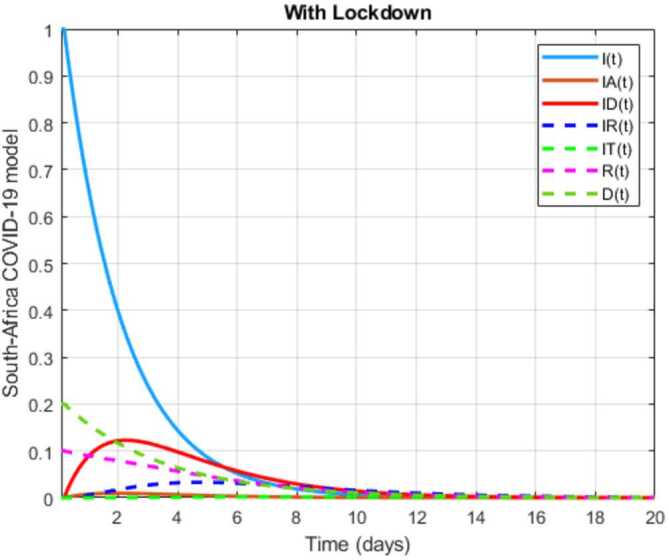
Numerical visualization for Covid-19 model in South Africa

We present a numerical simulation for Covid-19 model in Figs. 33 and 34.

**Figure 35:**
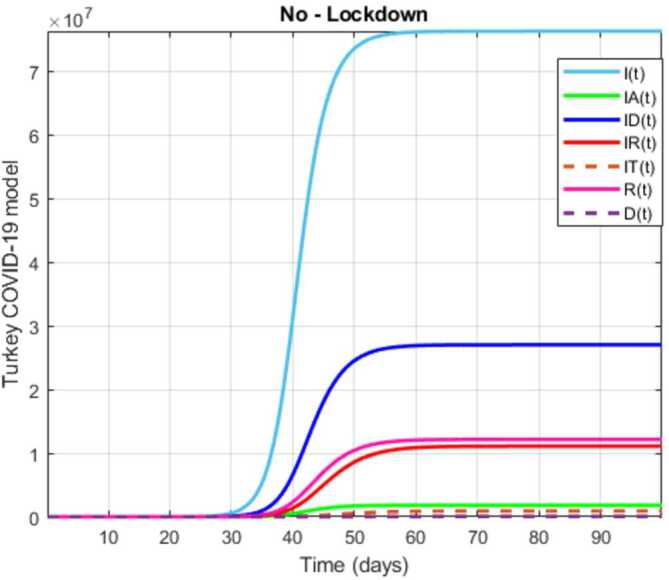
Numerical visualization for Covid-19 model in Turkey

**Figure 36:**
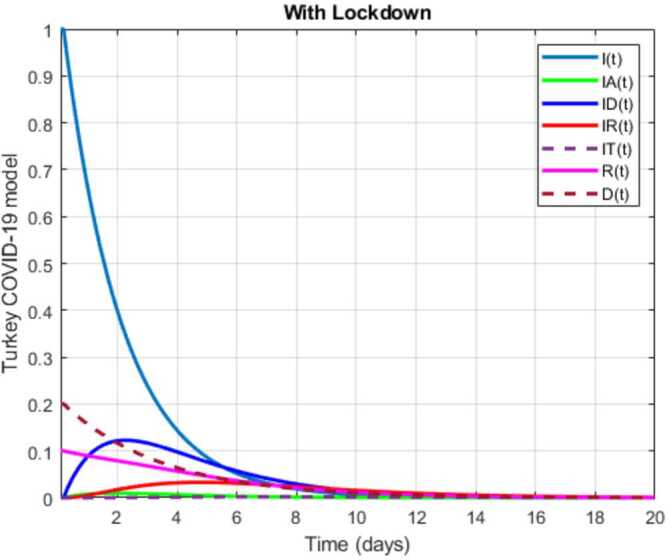
Numerical visualization for Covid-19 model in Turkey

In Figs. 35 and 36, the initial conditions are chosen as

5.9 $$\begin{aligned} &S ( 0 ) = 81000000,\qquad I ( 0 ) =1,\qquad I_{A} ( 0 ) =1,\qquad I_{D} ( 0 ) =1,\qquad I_{R} ( 0 ) =1, \\ &I_{T} ( 0 ) = 1,\qquad R ( 0 ) =0,\qquad D ( 0 ) =0,\qquad V ( 0 ) =0. \end{aligned}$$

Also the parameters are

5.10 $$\begin{aligned} \begin{aligned} &\Lambda = 80000000,\qquad k=2,\qquad p=0.5,\qquad \eta =0.12,\qquad \chi =0.015,\\ & v=0.027,\qquad x=0.4, \qquad\theta =0.301, \qquad \gamma = 0.09,\qquad \beta =0.013,\\ & \gamma _{1}=0.4,\qquad \mu _{1}=0.3,\qquad \varepsilon =0.161,\qquad \xi =0.015,\qquad \sigma =0.015,\\ & \tau = 0.0199,\qquad \Phi =0.2,\qquad \lambda =0.0345,\qquad \varphi =0.0345,\qquad \delta _{1}=0.01. \end{aligned} \end{aligned}$$

We present a numerical simulation for Covid-19 model in Figs. 35 and 36.

Now, we replace the classical differential operator by the operator with power-law, exponential decay, and Mittag-Leffler kernels. We start with the exponential decay kernel:

5.11 $$\begin{aligned} &{}_{0}^{CF}D_{t}^{\alpha }S = \Lambda - \bigl( \alpha ( x ) +\gamma _{1}+\mu _{1} \bigr) S, \\ &{}_{0}^{CF}D_{t}^{\alpha }I= \alpha ( x ) S- ( \varepsilon +\xi +\lambda +\mu _{1} ) I, \\ &{}_{0}^{CF}D_{t}^{\alpha }I_{A} = \xi I- ( \theta +\mu + \chi +\mu _{1} ) I_{A}, \\ &{}_{0}^{CF}D_{t}^{\alpha }I_{D} = \varepsilon I- ( \eta + \varphi +\mu _{1} ) I_{D}, \\ &{}_{0}^{CF}D_{t}^{\alpha }I_{R} = \eta I_{D}+\theta I_{A}- ( v+\xi +\mu _{1} ) I_{R}, \\ &{}_{0}^{CF}D_{t}^{\alpha }I_{T} = \mu I_{A}+vI_{R}- ( \sigma +\tau +\mu _{1} ) I_{T}, \\ &{}_{0}^{CF}D_{t}^{\alpha }R= \lambda I+\varphi I_{D}+ \chi I_{A}+\xi I_{R}+\sigma I_{T}- ( \Phi +\mu _{1} ) R, \\ &{}_{0}^{CF}D_{t}^{\alpha }D= \tau I_{T}, \\ &{}_{0}^{CF}D_{t}^{\alpha }V= \gamma _{1}S+\Phi R-\mu _{1}V. \end{aligned}$$

For simplicity, we write the above equations as follows:

5.12 $$\begin{aligned} &{}_{0}^{CF}D_{t}^{\alpha }S = \widetilde{S} ( t,S,I,I_{A},I_{D},I_{R},I_{T},R,D,V ), \\ &{}_{0}^{CF}D_{t}^{\alpha }I= \widetilde{I} ( t,S,I,I_{A},I_{D},I_{R},I_{T},R,D,V ), \\ &{}_{0}^{CF}D_{t}^{\alpha }I_{A} = \widetilde{I_{A}} ( t,S,I,I_{A},I_{D},I_{R},I_{T},R,D,V ), \\ &{}_{0}^{CF}D_{t}^{\alpha }I_{D} = \widetilde{I_{D}} ( t,S,I,I_{A},I_{D},I_{R},I_{T},R,D,V ), \\ &{}_{0}^{CF}D_{t}^{\alpha }I_{R} = \widetilde{I_{R}} ( t,S,I,I_{A},I_{D},I_{R},I_{T},R,D,V ), \\ &{}_{0}^{CF}D_{t}^{\alpha }I_{T} = \widetilde{I_{T}} ( t,S,I,I_{A},I_{D},I_{R},I_{T},R,D,V ), \\ &{}_{0}^{CF}D_{t}^{\alpha }R= \widetilde{R} ( t,S,I,I_{A},I_{D},I_{R},I_{T},R,D,V ), \\ &{}_{0}^{CF}D_{t}^{\alpha }D= \widetilde{D} ( t,S,I,I_{A},I_{D},I_{R},I_{T},R,D,V ), \\ &{}_{0}^{CF}D_{t}^{\alpha }V= \widetilde{V} ( t,S,I,I_{A},I_{D},I_{R},I_{T},R,D,V ). \end{aligned}$$

After applying fractal-fractional integral with the exponential kernel, we have the following:

5.13 $$\begin{aligned} S ( t_{p+1} ) ={}& S ( t_{p} ) + \frac{1-\alpha }{M ( \alpha ) } \begin{bmatrix} \widetilde{S} ( t_{p},S^{p},I^{p},I_{A}^{p},I_{D}^{ p},I_{R}^{p},I_{T}^{p},R^{p},D^{p},V^{p} ) \\ -\widetilde{S} ( t_{p-1},S^{p-1},I^{p-1},I_{A}^{p-1},I_{D}^{p-1},I_{R}^{p-1},I_{T}^{p-1},R^{p-1},D^{p-1},V^{p-1} )\end{bmatrix} \\ &{}+\frac{\alpha }{M ( \alpha ) } \int _{t_{p}}^{t_{p+1}} \widetilde{S} ( \tau,S,I,I_{A},I_{D},I_{R},I_{T},R,D,V ) \,d\tau, \\ I ( t_{p+1} ) = {}&I ( t_{p} ) + \frac{1-\alpha }{M ( \alpha ) } \begin{bmatrix} \widetilde{I} ( t_{p},S^{p},I^{p},I_{A}^{p},I_{D}^{ p},I_{R}^{p},I_{T}^{p},R^{p},D^{p},V^{p} ) \\ -\widetilde{I} ( t_{p-1},S^{p-1},I^{p-1},I_{A}^{p-1},I_{D}^{p-1},I_{R}^{p-1},I_{T}^{p-1},R^{p-1},D^{p-1},V^{p-1} )\end{bmatrix} \\ &{}+\frac{\alpha }{M ( \alpha ) } \int _{t_{p}}^{t_{p+1}} \widetilde{I} ( \tau,S,I,I_{A},I_{D},I_{R},I_{T},R,D,V ) \,d\tau, \\ I_{A} ( t_{p+1} ) = {}&I_{A} ( t_{p} ) + \frac{1-\alpha }{M ( \alpha ) } \begin{bmatrix} \widetilde{I_{A}} ( t_{p},S^{p},I^{p},I_{A}^{p},I_{D}^{p},I_{R}^{p},I_{T}^{p},R^{p},D^{p},V^{p} ) \\ -\widetilde{I_{A}} ( t_{p-1},S^{p-1},I^{p-1},I_{A}^{ p-1},I_{D}^{p-1},I_{R}^{p-1},I_{T}^{p-1},R^{p-1},D^{p-1},V^{p-1} )\end{bmatrix} \\ &{}+\frac{\alpha }{M ( \alpha ) } \int _{t_{p}}^{t_{p+1}} \widetilde{I_{A}} ( \tau,S,I,I_{A},I_{D},I_{R},I_{T},R,D,V ) \,d\tau, \\ I_{D} ( t_{p+1} ) = {}&I_{D} ( t_{p} ) + \frac{1-\alpha }{M ( \alpha ) } \begin{bmatrix} \widetilde{I_{D}} ( t_{p},S^{p},I^{p},I_{A}^{p},I_{D}^{ p},I_{R}^{p},I_{T}^{p},R^{p},D^{p},V^{p} ) \\ -\widetilde{I_{D}} ( t_{p-1},S^{p-1},I^{p-1},I_{A}^{p-1},I_{D}^{p-1},I_{R}^{p-1},I_{T}^{p-1},R^{p-1},D^{p-1},V^{p-1} )\end{bmatrix} \\ &{}+\frac{\alpha }{M ( \alpha ) } \int _{t_{p}}^{t_{p+1}} \widetilde{I_{D}} ( \tau,S,I,I_{A},I_{D},I_{R},I_{T},R,D,V ) \,d\tau, \\ I_{R} ( t_{p+1} ) ={}& I_{R} ( t_{p} ) + \frac{1-\alpha }{M ( \alpha ) } \begin{bmatrix} \widetilde{I_{R}} ( t_{p},S^{p},I^{p},I_{A}^{p},I_{D}^{ p},I_{R}^{p},I_{T}^{p},R^{p},D^{p},V^{p} ) \\ -\widetilde{I_{R}} ( t_{p-1},S^{p-1},I^{p-1},I_{A}^{p-1},I_{D}^{p-1},I_{R}^{p-1},I_{T}^{p-1},R^{p-1},D^{p-1},V^{p-1} )\end{bmatrix} \\ &{}+\frac{\alpha }{M ( \alpha ) } \int _{t_{p}}^{t_{p+1}} \widetilde{I_{R}} ( \tau,S,I,I_{A},I_{D},I_{R},I_{T},R,D,V ) \,d\tau, \\ I_{T} ( t_{p+1} ) ={}& I_{T} ( t_{p} ) + \frac{1-\alpha }{M ( \alpha ) } \begin{bmatrix} \widetilde{I_{T}} ( t_{p},S^{p},I^{p},I_{A}^{p},I_{D}^{ p},I_{R}^{p},I_{T}^{p},R^{p},D^{p},V^{p} ) \\ -\widetilde{I_{T}} ( t_{p-1},S^{p-1},I^{p-1},I_{A}^{p-1},I_{D}^{p-1},I_{R}^{p-1},I_{T}^{p-1},R^{p-1},D^{p-1},V^{p-1} )\end{bmatrix} \\ &{}+\frac{\alpha }{M ( \alpha ) } \int _{t_{p}}^{t_{p+1}} \widetilde{I_{T}} ( \tau,S,I,I_{A},I_{D},I_{R},I_{T},R,D,V ) \,d\tau, \\ R ( t_{p+1} ) = {}&R ( t_{p} ) + \frac{1-\alpha }{M ( \alpha ) } \begin{bmatrix} \widetilde{R} ( t_{p},S^{p},I^{p},I_{A}^{p},I_{D}^{ p},I_{R}^{p},I_{T}^{p},R^{p},D^{p},V^{p} ) \\ -\widetilde{R} ( t_{p-1},S^{p-1},I^{p-1},I_{A}^{p-1},I_{D}^{p-1},I_{R}^{p-1},I_{T}^{p-1},R^{p-1},D^{p-1},V^{p-1} )\end{bmatrix} \\ &{}+\frac{\alpha }{M ( \alpha ) } \int _{t_{p}}^{t_{p+1}} \widetilde{R} ( \tau,S,I,I_{A},I_{D},I_{R},I_{T},R,D,V ) \,d\tau, \\ D ( t_{p+1} ) ={}& D ( t_{p} ) + \frac{1-\alpha }{M ( \alpha ) } \begin{bmatrix} \widetilde{D} ( t_{p},S^{p},I^{p},I_{A}^{p},I_{D}^{ p},I_{R}^{p},I_{T}^{p},R^{p},D^{p},V^{p} ) \\ -\widetilde{D} ( t_{p-1},S^{p-1},I^{p-1},I_{A}^{p-1},I_{D}^{p-1},I_{R}^{p-1},I_{T}^{p-1},R^{p-1},D^{p-1},V^{p-1} )\end{bmatrix} \\ &{}+\frac{\alpha }{M ( \alpha ) } \int _{t_{p}}^{t_{p+1}} \widetilde{D} ( \tau,S,I,I_{A},I_{D},I_{R},I_{T},R,D,V ) \,d\tau, \\ V ( t_{p+1} ) = {}&V ( t_{p} ) + \frac{1-\alpha }{M ( \alpha ) } \begin{bmatrix} \widetilde{V} ( t_{p},S^{p},I^{p},I_{A}^{p},I_{D}^{ p},I_{R}^{p},I_{T}^{p},R^{p},D^{p},V^{p} ) \\ -\widetilde{V} ( t_{p-1},S^{p-1},I^{p-1},I_{A}^{p-1},I_{D}^{p-1},I_{R}^{p-1},I_{T}^{p-1},R^{p-1},D^{p-1},V^{p-1} )\end{bmatrix} \\ &{}+\frac{\alpha }{M ( \alpha ) } \int _{t_{p}}^{t_{p+1}} \widetilde{V} ( \tau,S,I,I_{A},I_{D},I_{R},I_{T},R,D,V ) \,d\tau. \end{aligned}$$

We can have the following scheme for this model:

5.14 $$\begin{array}{rl} S^{p+1}= & S^{p}+\frac{1-\alpha }{M(\alpha )}\left[\begin{array}{c} \tilde{S}(t_{p},S^{p},I^{p},I_{A}^{p},I_{D}^{p},I_{R}^{p},I_{T}^{p},R^{p},D^{p},V^{p})\\ -\tilde{S}(t_{p-1},S^{p-1},I^{p-1},I_{A}^{p-1},I_{D}^{p-1},I_{R}^{p-1},I_{T}^{p-1},R^{p-1},D^{p-1},V^{p-1}) \end{array}\right]\\ & +\frac{\alpha }{M(\alpha )}\left\{\begin{array}{c} \frac{23}{12}\tilde{S}(t_{p},S^{p},I^{p},I_{A}^{p},I_{D}^{p},I_{R}^{p},I_{T}^{p},R^{p},D^{p},V^{p})\Delta t\\ -\frac{4}{3}\tilde{S}(t_{p-1},S^{p-1},I^{p-1},I_{A}^{p-1},I_{D}^{p-1},I_{R}^{p-1},I_{T}^{p-1},R^{p-1},D^{p-1},V^{p-1})\Delta t\\ +\frac{5}{12}\tilde{S}(t_{p-2},S^{p-2},I^{p-2},I_{A}^{p-2},I_{D}^{p-2},I_{R}^{p-2},I_{T}^{p-2},R^{p-2},D^{p-2},V^{p-2})\Delta t \end{array}\right\},\\ I^{p+1}= & I^{p}+\frac{1-\alpha }{M(\alpha )}\left[\begin{array}{c} \tilde{I}(t_{p},S^{p},I^{p},I_{A}^{p},I_{D}^{p},I_{R}^{p},I_{T}^{p},R^{p},D^{p},V^{p})\\ -\tilde{I}(t_{p-1},S^{p-1},I^{p-1},I_{A}^{p-1},I_{D}^{p-1},I_{R}^{p-1},I_{T}^{p-1},R^{p-1},D^{p-1},V^{p-1}) \end{array}\right]\\ & +\frac{\alpha }{M(\alpha )}\left\{\begin{array}{c} \frac{23}{12}\tilde{I}(t_{p},S^{p},I^{p},I_{A}^{p},I_{D}^{p},I_{R}^{p},I_{T}^{p},R^{p},D^{p},V^{p})\Delta t\\ -\frac{4}{3}\tilde{I}(t_{p-1},S^{p-1},I^{p-1},I_{A}^{p-1},I_{D}^{p-1},I_{R}^{p-1},I_{T}^{p-1},R^{p-1},D^{p-1},V^{p-1})\Delta t\\ +\frac{5}{12}\tilde{I}(t_{p-2},S^{p-2},I^{p-2},I_{A}^{p-2},I_{D}^{p-2},I_{R}^{p-2},I_{T}^{p-2},R^{p-2},D^{p-2},V^{p-2})\Delta t \end{array}\right\},\\ I_{A}^{p+1}= & I_{A}^{p}+\frac{1-\alpha }{M(\alpha )}\left[\begin{array}{c} \tilde{I_{A}}(t_{p},S^{p},I^{p},I_{A}^{p},I_{D}^{p},I_{R}^{p},I_{T}^{p},R^{p},D^{p},V^{p})\\ -\tilde{I_{A}}(t_{p-1},S^{p-1},I^{p-1},I_{A}^{p-1},I_{D}^{p-1},I_{R}^{p-1},I_{T}^{p-1},R^{p-1},D^{p-1},V^{p-1}) \end{array}\right]\\ & +\frac{\alpha }{M(\alpha )}\left\{\begin{array}{c} \frac{23}{12}\tilde{I_{A}}(t_{p},S^{p},I^{p},I_{A}^{p},I_{D}^{p},I_{R}^{p},I_{T}^{p},R^{p},D^{p},V^{p})\Delta t\\ -\frac{4}{3}\tilde{I_{A}}(t_{p-1},S^{p-1},I^{p-1},I_{A}^{p-1},I_{D}^{p-1},I_{R}^{p-1},I_{T}^{p-1},R^{p-1},D^{p-1},V^{p-1})\Delta t\\ +\frac{5}{12}\tilde{I_{A}}(t_{p-2},S^{p-2},I^{p-2},I_{A}^{p-2},I_{D}^{p-2},I_{R}^{p-2},I_{T}^{p-2},R^{p-2},D^{p-2},V^{p-2})\Delta t \end{array}\right\},\\ I_{D}^{p+1}= & I_{D}^{p}+\frac{1-\alpha }{M(\alpha )}\left[\begin{array}{c} \tilde{I_{D}}(t_{p},S^{p},I^{p},I_{A}^{p},I_{D}^{p},I_{R}^{p},I_{T}^{p},R^{p},D^{p},V^{p})\\ -\tilde{I_{D}}(t_{p-1},S^{p-1},I^{p-1},I_{A}^{p-1},I_{D}^{p-1},I_{R}^{p-1},I_{T}^{p-1},R^{p-1},D^{p-1},V^{p-1}) \end{array}\right]\\ & +\frac{\alpha }{M(\alpha )}\left\{\begin{array}{c} \frac{23}{12}\tilde{I_{D}}(t_{p},S^{p},I^{p},I_{A}^{p},I_{D}^{p},I_{R}^{p},I_{T}^{p},R^{p},D^{p},V^{p})\Delta t\\ -\frac{4}{3}\tilde{I_{D}}(t_{p-1},S^{p-1},I^{p-1},I_{A}^{p-1},I_{D}^{p-1},I_{R}^{p-1},I_{T}^{p-1},R^{p-1},D^{p-1},V^{p-1})\Delta t\\ +\frac{5}{12}\tilde{I_{D}}(t_{p-2},S^{p-2},I^{p-2},I_{A}^{p-2},I_{D}^{p-2},I_{R}^{p-2},I_{T}^{p-2},R^{p-2},D^{p-2},V^{p-2})\Delta t \end{array}\right\},\\ I_{R}^{p+1}= & I_{R}^{p}+\frac{1-\alpha }{M(\alpha )}\left[\begin{array}{c} \tilde{I_{R}}(t_{p},S^{p},I^{p},I_{A}^{p},I_{D}^{p},I_{R}^{p},I_{T}^{p},R^{p},D^{p},V^{p})\\ -\tilde{I_{R}}(t_{p-1},S^{p-1},I^{p-1},I_{A}^{p-1},I_{D}^{p-1},I_{R}^{p-1},I_{T}^{p-1},R^{p-1},D^{p-1},V^{p-1}) \end{array}\right]\\ & +\frac{\alpha }{M(\alpha )}\left\{\begin{array}{c} \frac{23}{12}\tilde{I_{R}}(t_{p},S^{p},I^{p},I_{A}^{p},I_{D}^{p},I_{R}^{p},I_{T}^{p},R^{p},D^{p},V^{p})\Delta t\\ -\frac{4}{3}\tilde{I_{R}}(t_{p-1},S^{p-1},I^{p-1},I_{A}^{p-1},I_{D}^{p-1},I_{R}^{p-1},I_{T}^{p-1},R^{p-1},D^{p-1},V^{p-1})\Delta t\\ +\frac{5}{12}\tilde{I_{R}}(t_{p-2},S^{p-2},I^{p-2},I_{A}^{p-2},I_{D}^{p-2},I_{R}^{p-2},I_{T}^{p-2},R^{p-2},D^{p-2},V^{p-2})\Delta t \end{array}\right\},\\ I_{T}^{p+1}= & I_{T}^{p}+\frac{1-\alpha }{M(\alpha )}\left[\begin{array}{c} \tilde{I_{T}}(t_{p},S^{p},I^{p},I_{A}^{p},I_{D}^{p},I_{R}^{p},I_{T}^{p},R^{p},D^{p},V^{p})\\ -\tilde{I_{T}}(t_{p-1},S^{p-1},I^{p-1},I_{A}^{p-1},I_{D}^{p-1},I_{R}^{p-1},I_{T}^{p-1},R^{p-1},D^{p-1},V^{p-1}) \end{array}\right]\\ & +\frac{\alpha }{M(\alpha )}\left\{\begin{array}{c} \frac{23}{12}\tilde{I_{T}}(t_{p},S^{p},I^{p},I_{A}^{p},I_{D}^{p},I_{R}^{p},I_{T}^{p},R^{p},D^{p},V^{p})\Delta t\\ -\frac{4}{3}\tilde{I_{T}}(t_{p-1},S^{p-1},I^{p-1},I_{A}^{p-1},I_{D}^{p-1},I_{R}^{p-1},I_{T}^{p-1},R^{p-1},D^{p-1},V^{p-1})\Delta t\\ +\frac{5}{12}\tilde{I_{T}}(t_{p-2},S^{p-2},I^{p-2},I_{A}^{p-2},I_{D}^{p-2},I_{R}^{p-2},I_{T}^{p-2},R^{p-2},D^{p-2},V^{p-2})\Delta t \end{array}\right\},\\ R^{p+1}= & R^{p}+\frac{1-\alpha }{M(\alpha )}\left[\begin{array}{c} \tilde{R}(t_{p},S^{p},I^{p},I_{A}^{p},I_{D}^{p},I_{R}^{p},I_{T}^{p},R^{p},D^{p},V^{p})\\ -\tilde{R}(t_{p-1},S^{p-1},I^{p-1},I_{A}^{p-1},I_{D}^{p-1},I_{R}^{p-1},I_{T}^{p-1},R^{p-1},D^{p-1},V^{p-1}) \end{array}\right]\\ & +\frac{\alpha }{M(\alpha )}\left\{\begin{array}{c} \frac{23}{12}\tilde{R}(t_{p},S^{p},I^{p},I_{A}^{p},I_{D}^{p},I_{R}^{p},I_{T}^{p},R^{p},D^{p},V^{p})\Delta t\\ -\frac{4}{3}\tilde{R}(t_{p-1},S^{p-1},I^{p-1},I_{A}^{p-1},I_{D}^{p-1},I_{R}^{p-1},I_{T}^{p-1},R^{p-1},D^{p-1},V^{p-1})\Delta t\\ +\frac{5}{12}\tilde{R}(t_{p-2},S^{p-2},I^{p-2},I_{A}^{p-2},I_{D}^{p-2},I_{R}^{p-2},I_{T}^{p-2},R^{p-2},D^{p-2},V^{p-2})\Delta t \end{array}\right\},\\ D^{p+1}= & D^{p}+\frac{1-\alpha }{M(\alpha )}\left[\begin{array}{c} \tilde{D}(t_{p},S^{p},I^{p},I_{A}^{p},I_{D}^{p},I_{R}^{p},I_{T}^{p},R^{p},D^{p},V^{p})\\ -\tilde{D}(t_{p-1},S^{p-1},I^{p-1},I_{A}^{p-1},I_{D}^{p-1},I_{R}^{p-1},I_{T}^{p-1},R^{p-1},D^{p-1},V^{p-1}) \end{array}\right]\\ & +\frac{\alpha }{M(\alpha )}\left\{\begin{array}{c} \frac{23}{12}\tilde{D}(t_{p},S^{p},I^{p},I_{A}^{p},I_{D}^{p},I_{R}^{p},I_{T}^{p},R^{p},D^{p},V^{p})\Delta t\\ -\frac{4}{3}\tilde{D}(t_{p-1},S^{p-1},I^{p-1},I_{A}^{p-1},I_{D}^{p-1},I_{R}^{p-1},I_{T}^{p-1},R^{p-1},D^{p-1},V^{p-1})\Delta t\\ +\frac{5}{12}\tilde{D}(t_{p-2},S^{p-2},I^{p-2},I_{A}^{p-2},I_{D}^{p-2},I_{R}^{p-2},I_{T}^{p-2},R^{p-2},D^{p-2},V^{p-2})\Delta t \end{array}\right\},\\ V^{p+1}= & V^{p}+\frac{1-\alpha }{M(\alpha )}\left[\begin{array}{c} \tilde{V}(t_{p},S^{p},I^{p},I_{A}^{p},I_{D}^{p},I_{R}^{p},I_{T}^{p},R^{p},D^{p},V^{p})\\ -\tilde{V}(t_{p-1},S^{p-1},I^{p-1},I_{A}^{p-1},I_{D}^{p-1},I_{R}^{p-1},I_{T}^{p-1},R^{p-1},D^{p-1},V^{p-1}) \end{array}\right]\\ & +\frac{\alpha }{M(\alpha )}\left\{\begin{array}{c} \frac{23}{12}\tilde{V}(t_{p},S^{p},I^{p},I_{A}^{p},I_{D}^{p},I_{R}^{p},I_{T}^{p},R^{p},D^{p},V^{p})\Delta t\\ -\frac{4}{3}\tilde{V}(t_{p-1},S^{p-1},I^{p-1},I_{A}^{p-1},I_{D}^{p-1},I_{R}^{p-1},I_{T}^{p-1},R^{p-1},D^{p-1},V^{p-1})\Delta t\\ +\frac{5}{12}\tilde{V}(t_{p-2},S^{p-2},I^{p-2},I_{A}^{p-2},I_{D}^{p-2},I_{R}^{p-2},I_{T}^{p-2},R^{p-2},D^{p-2},V^{p-2})\Delta t \end{array}\right\}. \end{array}$$

For the Mittag-Leffler kernel, we have the following:

5.15 $$\begin{aligned} S^{p+1} ={}& S^{p}+\frac{1-\alpha }{AB ( \alpha ) } \widetilde{S} \bigl( t_{p},S^{p},I^{p},I_{A}^{p},I_{D}^{p},I_{R}^{ p},I_{T}^{p},R^{p},D^{p},V^{p} \bigr) \\ &{}\times\frac{\alpha }{AB ( \alpha ) \Gamma ( \alpha ) }\sum_{r=2}^{p} \int _{t_{r}}^{t_{r+1}}\widetilde{S} ( \tau,S,I,I_{A},I_{D},I_{R},I_{T},R,D,V ) ( t_{p+1}-\tau ) ^{\alpha -1}\,d\tau, \\ I^{p+1} ={}& I^{p}+\frac{1-\alpha }{AB ( \alpha ) } \widetilde{I} \bigl( t_{p},S^{p},I^{p},I_{A}^{p},I_{D}^{p},I_{R}^{ p},I_{T}^{p},R^{p},D^{p},V^{p} \bigr) \\ &{}\times\frac{\alpha }{AB ( \alpha ) \Gamma ( \alpha ) }\sum_{r=2}^{p} \int _{t_{r}}^{t_{r+1}}\widetilde{I} ( \tau,S,I,I_{A},I_{D},I_{R},I_{T},R,D,V ) ( t_{p+1}-\tau ) ^{\alpha -1}\,d\tau, \\ I_{A}^{p+1} ={}& I_{A}^{p}+ \frac{1-\alpha }{AB ( \alpha ) }\widetilde{I_{A}} \bigl( t_{p},S^{p},I^{p},I_{A}^{p},I_{D}^{ p},I_{R}^{p},I_{T}^{p},R^{p},D^{p},V^{p} \bigr) \\ &{}\times\frac{\alpha }{AB ( \alpha ) \Gamma ( \alpha ) }\sum_{r=2}^{p} \int _{t_{r}}^{t_{r+1}}\widetilde{I_{A}} ( \tau,S,I,I_{A},I_{D},I_{R},I_{T},R,D,V ) ( t_{p+1}-\tau ) ^{\alpha -1}\,d\tau, \\ I_{D}^{p+1} ={}& I_{D}^{p}+ \frac{1-\alpha }{AB ( \alpha ) }\widetilde{I_{D}} \bigl( t_{p},S^{p},I^{p},I_{A}^{p},I_{D}^{ p},I_{R}^{p},I_{T}^{p},R^{p},D^{p},V^{p} \bigr) \\ &{}\times\frac{\alpha }{AB ( \alpha ) \Gamma ( \alpha ) }\sum_{r=2}^{p} \int _{t_{r}}^{t_{r+1}}\widetilde{I_{D}} ( \tau,S,I,I_{A},I_{D},I_{R},I_{T},R,D,V ) ( t_{p+1}-\tau ) ^{\alpha -1}\,d\tau, \\ I_{R}^{p+1} ={}& I_{R}^{p}+ \frac{1-\alpha }{AB ( \alpha ) }\widetilde{I_{R}} \bigl( t_{p},S^{p},I^{p},I_{A}^{p},I_{D}^{ p},I_{R}^{p},I_{T}^{p},R^{p},D^{p},V^{p} \bigr) \\ &{}\times\frac{\alpha }{AB ( \alpha ) \Gamma ( \alpha ) }\sum_{r=2}^{p} \int _{t_{r}}^{t_{r+1}}\widetilde{I_{R}} ( \tau,S,I,I_{A},I_{D},I_{R},I_{T},R,D,V ) ( t_{p+1}-\tau ) ^{\alpha -1}\,d\tau, \\ I_{T}^{p+1} ={}& I_{T}^{p}+ \frac{1-\alpha }{AB ( \alpha ) }\widetilde{I_{T}} \bigl( t_{p},S^{p},I^{p},I_{A}^{p},I_{D}^{ p},I_{R}^{p},I_{T}^{p},R^{p},D^{p},V^{p} \bigr) \\ &{}\times\frac{\alpha }{AB ( \alpha ) \Gamma ( \alpha ) }\sum_{r=2}^{p} \int _{t_{r}}^{t_{r+1}}\widetilde{I_{T}} ( \tau,S,I,I_{A},I_{D},I_{R},I_{T},R,D,V ) ( t_{p+1}-\tau ) ^{\alpha -1}\,d\tau, \\ R^{p+1} ={}& R^{p}+\frac{1-\alpha }{AB ( \alpha ) } \widetilde{R} \bigl( t_{p},S^{p},I^{p},I_{A}^{p},I_{D}^{p},I_{R}^{ p},I_{T}^{p},R^{p},D^{p},V^{p} \bigr) \\ &{}\times\frac{\alpha }{AB ( \alpha ) \Gamma ( \alpha ) }\sum_{r=2}^{p} \int _{t_{r}}^{t_{r+1}}\widetilde{R} ( \tau,S,I,I_{A},I_{D},I_{R},I_{T},R,D,V ) ( t_{p+1}-\tau ) ^{\alpha -1}\,d\tau, \\ D^{p+1} ={}& D^{p}+\frac{1-\alpha }{AB ( \alpha ) } \widetilde{D} \bigl( t_{p},S^{p},I^{p},I_{A}^{p},I_{D}^{p},I_{R}^{ p},I_{T}^{p},R^{p},D^{p},V^{p} \bigr) \\ &{}\times\frac{\alpha }{AB ( \alpha ) \Gamma ( \alpha ) }\sum_{r=2}^{p} \int _{t_{r}}^{t_{r+1}}\widetilde{D} ( \tau,S,I,I_{A},I_{D},I_{R},I_{T},R,D,V ) ( t_{p+1}-\tau ) ^{\alpha -1}\,d\tau, \\ V^{p+1} ={}& V^{p}+\frac{1-\alpha }{AB ( \alpha ) } \widetilde{V} \bigl( t_{p},S^{p},I^{p},I_{A}^{p},I_{D}^{p},I_{R}^{ p},I_{T}^{p},R^{p},D^{p},V^{p} \bigr) \\ &{}\frac{\alpha }{AB ( \alpha ) \Gamma ( \alpha ) }\sum_{r=2}^{p} \int _{t_{r}}^{t_{r+1}}\widetilde{V} ( \tau,S,I,I_{A},I_{D},I_{R},I_{T},R,D,V ) ( t_{p+1}-\tau ) ^{\alpha -1}\,d\tau. \end{aligned}$$

We can get the following numerical scheme:

5.16 $$\begin{aligned} S^{p+1} = {}&\frac{1-\alpha }{AB ( \alpha ) }\widetilde{S} \bigl( t_{p},S^{p},I^{p},I_{A}^{p},I_{D}^{p},I_{R}^{ p},I_{T}^{p},R^{p},D^{p},V^{p} \bigr) \\ &{}+ \frac{\alpha ( \Delta t ) ^{\alpha }}{AB ( \alpha ) \Gamma ( \alpha +1 ) }\sum_{r=2}^{p} \widetilde{S} \bigl( t_{r-2},S^{r-2},I^{r-2},I_{A}^{ r-2},I_{D}^{r-2},I_{R}^{r-2},I_{T}^{r-2},R^{r-2},D^{r-2},V^{r-2} \bigr) \\ &{}\times \bigl[ ( p-r+1 ) ^{\alpha }- ( p-r ) ^{ \alpha } \bigr] \\ &{}+ \frac{\alpha ( \Delta t ) ^{\alpha }}{AB ( \alpha ) \Gamma ( \alpha +2 ) }\sum_{r=2}^{p} \begin{bmatrix} \widetilde{S} ( t_{r-1},S^{r-1},I^{r-1},I_{A}^{r-1},I_{D}^{ r-1},I_{R}^{r-1},I_{T}^{r-1},R^{r-1},D^{r-1},V^{r-1} ) \\ -\widetilde{S} ( t_{r-2},S^{r-2},I^{r-2},I_{A}^{r-2},I_{D}^{r-2},I_{R}^{r-2},I_{T}^{r-2},R^{r-2},D^{r-2},V^{r-2} )\end{bmatrix} \\ &{}\times \begin{bmatrix} ( p-r+1 ) ^{\alpha } ( p-r+3+2\alpha ) \\ - ( p-r ) ^{\alpha } ( p-r+3+3\alpha )\end{bmatrix} \\ &{}+ \frac{\alpha ( \Delta t ) ^{\alpha }}{2AB ( \alpha ) \Gamma ( \alpha +3 ) } \\ &{}\times \sum_{r=2}^{p} \begin{bmatrix} \widetilde{S} ( t_{r},S^{r},I^{r},I_{A}^{r},I_{D}^{ r},I_{R}^{r},I_{T}^{r},R^{r},D^{r},V^{r} ) \\ -2\widetilde{S} ( t_{r-1},S^{r-1},I^{r-1},I_{A}^{r-1},I_{D}^{r-1},I_{R}^{r-1},I_{T}^{r-1},R^{r-1},D^{r-1},V^{r-1} ) \\ +\widetilde{S} ( t_{r-2},S^{r-2},I^{r-2},I_{A}^{r-2},I_{D}^{r-2},I_{R}^{r-2},I_{T}^{r-2},R^{r-2},D^{r-2},V^{r-2} )\end{bmatrix} \\ &{}\times \begin{bmatrix} ( p-r+1 ) ^{\alpha } \begin{bmatrix} 2 ( p-r ) ^{2}+ ( 3\alpha +10 ) ( p-r ) \\ +2\alpha ^{2}+9\alpha +12\end{bmatrix} \\ - ( p-r ) ^{\alpha } \begin{bmatrix} 2 ( p-r ) ^{2}+ ( 5\alpha +10 ) ( p-r ) \\ +6\alpha ^{2}+18\alpha +12\end{bmatrix}\end{bmatrix}, \\ I^{p+1} ={}& \frac{1-\alpha }{AB ( \alpha ) }\widetilde{I} \bigl( t_{p},S^{p},I^{p},I_{A}^{p},I_{D}^{p},I_{R}^{ p},I_{T}^{p},R^{p},D^{p},V^{p} \bigr) \\ &{}+ \frac{\alpha ( \Delta t ) ^{\alpha }}{AB ( \alpha ) \Gamma ( \alpha +1 ) }\sum_{r=2}^{p} \widetilde{I} \bigl( t_{r-2},S^{r-2},I^{r-2},I_{A}^{ r-2},I_{D}^{r-2},I_{R}^{r-2},I_{T}^{r-2},R^{r-2},D^{r-2},V^{r-2} \bigr) \\ &{}\times \bigl[ ( p-r+1 ) ^{\alpha }- ( p-r ) ^{ \alpha } \bigr] \\ &{}+ \frac{\alpha ( \Delta t ) ^{\alpha }}{AB ( \alpha ) \Gamma ( \alpha +2 ) }\sum_{r=2}^{p} \begin{bmatrix} \widetilde{I} ( t_{r-1},S^{r-1},I^{r-1},I_{A}^{r-1},I_{D}^{ r-1},I_{R}^{r-1},I_{T}^{r-1},R^{r-1},D^{r-1},V^{r-1} ) \\ -\widetilde{I} ( t_{r-2},S^{r-2},I^{r-2},I_{A}^{r-2},I_{D}^{r-2},I_{R}^{r-2},I_{T}^{r-2},R^{r-2},D^{r-2},V^{r-2} )\end{bmatrix} \end{aligned}$$

$$\begin{aligned} &{}\times \begin{bmatrix} ( p-r+1 ) ^{\alpha } ( p-r+3+2\alpha ) \\ - ( p-r ) ^{\alpha } ( p-r+3+3\alpha )\end{bmatrix} \\ &{}+ \frac{\alpha ( \Delta t ) ^{\alpha }}{2AB ( \alpha ) \Gamma ( \alpha +3 ) }\sum_{r=2}^{p} \\ &{}\times \begin{bmatrix} \widetilde{I} ( t_{r},S^{r},I^{r},I_{A}^{r},I_{D}^{ r},I_{R}^{r},I_{T}^{r},R^{r},D^{r},V^{r} ) \\ -2\widetilde{I} ( t_{r-1},S^{r-1},I^{r-1},I_{A}^{r-1},I_{D}^{r-1},I_{R}^{r-1},I_{T}^{r-1},R^{r-1},D^{r-1},V^{r-1} ) \\ +\widetilde{I} ( t_{r-2},S^{r-2},I^{r-2},I_{A}^{r-2},I_{D}^{r-2},I_{R}^{r-2},I_{T}^{r-2},R^{r-2},D^{r-2},V^{r-2} )\end{bmatrix} \\ &{}\times \begin{bmatrix} ( p-r+1 ) ^{\alpha } \begin{bmatrix} 2 ( p-r ) ^{2}+ ( 3\alpha +10 ) ( p-r ) \\ +2\alpha ^{2}+9\alpha +12\end{bmatrix} \\ - ( p-r ) ^{\alpha } \begin{bmatrix} 2 ( p-r ) ^{2}+ ( 5\alpha +10 ) ( p-r ) \\ +6\alpha ^{2}+18\alpha +12\end{bmatrix}\end{bmatrix}, \\ I_{A}^{p+1} ={}& \frac{1-\alpha }{AB ( \alpha ) } \widetilde{I_{A}} \bigl( t_{p},S^{p},I^{p},I_{A}^{p},I_{D}^{p},I_{R}^{ p},I_{T}^{p},R^{p},D^{p},V^{p} \bigr) \\ &{}+ \frac{\alpha ( \Delta t ) ^{\alpha }}{AB ( \alpha ) \Gamma ( \alpha +1 ) }\sum_{r=2}^{p} \widetilde{I_{A}} \bigl( t_{r-2},S^{r-2},I^{r-2},I_{A}^{r-2},I_{D}^{r-2},I_{R}^{ r-2},I_{T}^{r-2},R^{r-2},D^{r-2},V^{r-2} \bigr) \\ &{}\times \bigl[ ( p-r+1 ) ^{\alpha }- ( p-r ) ^{ \alpha } \bigr] \\ &{}+ \frac{\alpha ( \Delta t ) ^{\alpha }}{AB ( \alpha ) \Gamma ( \alpha +2 ) }\sum_{r=2}^{p} \begin{bmatrix} \widetilde{I_{A}} ( t_{r-1},S^{r-1},I^{r-1},I_{A}^{r-1},I_{D}^{r-1},I_{R}^{r-1},I_{T}^{r-1},R^{r-1},D^{r-1},V^{r-1} ) \\ -\widetilde{I_{A}} ( t_{r-2},S^{r-2},I^{r-2},I_{A}^{r-2},I_{D}^{r-2},I_{R}^{r-2},I_{T}^{r-2},R^{r-2},D^{r-2},V^{r-2} )\end{bmatrix} \\ &{}\times \begin{bmatrix} ( p-r+1 ) ^{\alpha } ( p-r+3+2\alpha ) \\ - ( p-r ) ^{\alpha } ( p-r+3+3\alpha )\end{bmatrix} \\ &{}+ \frac{\alpha ( \Delta t ) ^{\alpha }}{2AB ( \alpha ) \Gamma ( \alpha +3 ) }\sum_{r=2}^{p} \\ &{}\times \begin{bmatrix} \widetilde{I_{A}} ( t_{r},S^{r},I^{r},I_{A}^{r},I_{D}^{ r},I_{R}^{r},I_{T}^{r},R^{r},D^{r},V^{r} ) \\ -2\widetilde{I_{A}} ( t_{r-1},S^{r-1},I^{r-1},I_{A}^{r-1},I_{D}^{r-1},I_{R}^{r-1},I_{T}^{r-1},R^{r-1},D^{r-1},V^{r-1} ) \\ +\widetilde{I_{A}} ( t_{r-2},S^{r-2},I^{r-2},I_{A}^{r-2},I_{D}^{r-2},I_{R}^{r-2},I_{T}^{r-2},R^{r-2},D^{r-2},V^{r-2} )\end{bmatrix} \\ &{}\times \begin{bmatrix} ( p-r+1 ) ^{\alpha } \begin{bmatrix} 2 ( p-r ) ^{2}+ ( 3\alpha +10 ) ( p-r ) \\ +2\alpha ^{2}+9\alpha +12\end{bmatrix} \\ - ( p-r ) ^{\alpha } \begin{bmatrix} 2 ( p-r ) ^{2}+ ( 5\alpha +10 ) ( p-r ) \\ +6\alpha ^{2}+18\alpha +12\end{bmatrix}\end{bmatrix}, \\ I_{D}^{p+1} ={}& \frac{1-\alpha }{AB ( \alpha ) } \widetilde{I_{D}} \bigl( t_{p},S^{p},I^{p},I_{A}^{p},I_{D}^{p},I_{R}^{ p},I_{T}^{p},R^{p},D^{p},V^{p} \bigr) \\ &{}+ \frac{\alpha ( \Delta t ) ^{\alpha }}{AB ( \alpha ) \Gamma ( \alpha +1 ) }\sum_{r=2}^{p} \widetilde{I_{D}} \bigl( t_{r-2},S^{r-2},I^{r-2},I_{A}^{r-2},I_{D}^{r-2},I_{R}^{ r-2},I_{T}^{r-2},R^{r-2},D^{r-2},V^{r-2} \bigr) \\ &{}\times \bigl[ ( p-r+1 ) ^{\alpha }- ( p-r ) ^{ \alpha } \bigr] \\ &{}+ \frac{\alpha ( \Delta t ) ^{\alpha }}{AB ( \alpha ) \Gamma ( \alpha +2 ) }\sum_{r=2}^{p} \begin{bmatrix} \widetilde{I_{D}} ( t_{r-1},S^{r-1},I^{r-1},I_{A}^{r-1},I_{D}^{r-1},I_{R}^{r-1},I_{T}^{r-1},R^{r-1},D^{r-1},V^{r-1} ) \\ -\widetilde{I_{D}} ( t_{r-2},S^{r-2},I^{r-2},I_{A}^{r-2},I_{D}^{r-2},I_{R}^{r-2},I_{T}^{r-2},R^{r-2},D^{r-2},V^{r-2} )\end{bmatrix} \\ &{}\times \begin{bmatrix} ( p-r+1 ) ^{\alpha } ( p-r+3+2\alpha ) \\ - ( p-r ) ^{\alpha } ( p-r+3+3\alpha )\end{bmatrix} \end{aligned}$$

$$\begin{aligned} &{}+ \frac{\alpha ( \Delta t ) ^{\alpha }}{2AB ( \alpha ) \Gamma ( \alpha +3 ) } \\ &{}\times \sum_{r=2}^{p} \begin{bmatrix} \widetilde{I_{D}} ( t_{r},S^{r},I^{r},I_{A}^{r},I_{D}^{ r},I_{R}^{r},I_{T}^{r},R^{r},D^{r},V^{r} ) \\ -2\widetilde{I_{D}} ( t_{r-1},S^{r-1},I^{r-1},I_{A}^{r-1},I_{D}^{r-1},I_{R}^{r-1},I_{T}^{r-1},R^{r-1},D^{r-1},V^{r-1} ) \\ +\widetilde{I_{D}} ( t_{r-2},S^{r-2},I^{r-2},I_{A}^{r-2},I_{D}^{r-2},I_{R}^{r-2},I_{T}^{r-2},R^{r-2},D^{r-2},V^{r-2} )\end{bmatrix} \\ &{}\times \begin{bmatrix} ( p-r+1 ) ^{\alpha } \begin{bmatrix} 2 ( p-r ) ^{2}+ ( 3\alpha +10 ) ( p-r ) \\ +2\alpha ^{2}+9\alpha +12\end{bmatrix} \\ - ( p-r ) ^{\alpha } \begin{bmatrix} 2 ( p-r ) ^{2}+ ( 5\alpha +10 ) ( p-r ) \\ +6\alpha ^{2}+18\alpha +12\end{bmatrix}\end{bmatrix}, \\ I_{R}^{p+1} = {}&\frac{1-\alpha }{AB ( \alpha ) } \widetilde{I_{R}} \bigl( t_{p},S^{p},I^{p},I_{A}^{p},I_{D}^{p},I_{R}^{ p},I_{T}^{p},R^{p},D^{p},V^{p} \bigr) \\ &{}+ \frac{\alpha ( \Delta t ) ^{\alpha }}{AB ( \alpha ) \Gamma ( \alpha +1 ) }\sum_{r=2}^{p} \widetilde{I_{R}} \bigl( t_{r-2},S^{r-2},I^{r-2},I_{A}^{r-2},I_{D}^{r-2},I_{R}^{ r-2},I_{T}^{r-2},R^{r-2},D^{r-2},V^{r-2} \bigr) \\ &{}\times \bigl[ ( p-r+1 ) ^{\alpha }- ( p-r ) ^{ \alpha } \bigr] \\ &{}+ \frac{\alpha ( \Delta t ) ^{\alpha }}{AB ( \alpha ) \Gamma ( \alpha +2 ) }\sum_{r=2}^{p} \begin{bmatrix} \widetilde{I_{R}} ( t_{r-1},S^{r-1},I^{r-1},I_{A}^{r-1},I_{D}^{r-1},I_{R}^{r-1},I_{T}^{r-1},R^{r-1},D^{r-1},V^{r-1} ) \\ -\widetilde{I_{R}} ( t_{r-2},S^{r-2},I^{r-2},I_{A}^{r-2},I_{D}^{r-2},I_{R}^{r-2},I_{T}^{r-2},R^{r-2},D^{r-2},V^{r-2} )\end{bmatrix} \\ &{}\times \begin{bmatrix} ( p-r+1 ) ^{\alpha } ( p-r+3+2\alpha ) \\ - ( p-r ) ^{\alpha } ( p-r+3+3\alpha )\end{bmatrix} \\ &{}+ \frac{\alpha ( \Delta t ) ^{\alpha }}{2AB ( \alpha ) \Gamma ( \alpha +3 ) } \\ &{}\times \sum_{r=2}^{p} \begin{bmatrix} \widetilde{I_{R}} ( t_{r},S^{r},I^{r},I_{A}^{r},I_{D}^{ r},I_{R}^{r},I_{T}^{r},R^{r},D^{r},V^{r} ) \\ -2\widetilde{I_{R}} ( t_{r-1},S^{r-1},I^{r-1},I_{A}^{r-1},I_{D}^{r-1},I_{R}^{r-1},I_{T}^{r-1},R^{r-1},D^{r-1},V^{r-1} ) \\ +\widetilde{I_{R}} ( t_{r-2},S^{r-2},I^{r-2},I_{A}^{r-2},I_{D}^{r-2},I_{R}^{r-2},I_{T}^{r-2},R^{r-2},D^{r-2},V^{r-2} )\end{bmatrix} \\ &{}\times \begin{bmatrix} ( p-r+1 ) ^{\alpha } \begin{bmatrix} 2 ( p-r ) ^{2}+ ( 3\alpha +10 ) ( p-r ) \\ +2\alpha ^{2}+9\alpha +12\end{bmatrix} \\ - ( p-r ) ^{\alpha } \begin{bmatrix} 2 ( p-r ) ^{2}+ ( 5\alpha +10 ) ( p-r ) \\ &{}+6\alpha ^{2}+18\alpha +12\end{bmatrix}\end{bmatrix}, \\ I_{T}^{p+1} ={}& \frac{1-\alpha }{AB ( \alpha ) } \widetilde{I_{T}} \bigl( t_{p},S^{p},I^{p},I_{A}^{p},I_{D}^{p},I_{R}^{ p},I_{T}^{p},R^{p},D^{p},V^{p} \bigr) \\ &{}+ \frac{\alpha ( \Delta t ) ^{\alpha }}{AB ( \alpha ) \Gamma ( \alpha +1 ) }\sum_{r=2}^{p} \widetilde{I_{T}} \bigl( t_{r-2},S^{r-2},I^{r-2},I_{A}^{r-2},I_{D}^{r-2},I_{R}^{ r-2},I_{T}^{r-2},R^{r-2},D^{r-2},V^{r-2} \bigr) \\ &{}\times \bigl[ ( p-r+1 ) ^{\alpha }- ( p-r ) ^{ \alpha } \bigr] \\ &{}+ \frac{\alpha ( \Delta t ) ^{\alpha }}{AB ( \alpha ) \Gamma ( \alpha +2 ) }\sum_{r=2}^{p} \begin{bmatrix} \widetilde{I_{T}} ( t_{r-1},S^{r-1},I^{r-1},I_{A}^{r-1},I_{D}^{r-1},I_{R}^{r-1},I_{T}^{r-1},R^{r-1},D^{r-1},V^{r-1} ) \\ -\widetilde{I_{T}} ( t_{r-2},S^{r-2},I^{r-2},I_{A}^{r-2},I_{D}^{r-2},I_{R}^{r-2},I_{T}^{r-2},R^{r-2},D^{r-2},V^{r-2} )\end{bmatrix} \\ &{}\times \begin{bmatrix} ( p-r+1 ) ^{\alpha } ( p-r+3+2\alpha ) \\ - ( p-r ) ^{\alpha } ( p-r+3+3\alpha )\end{bmatrix} \\ &{}+ \frac{\alpha ( \Delta t ) ^{\alpha }}{2AB ( \alpha ) \Gamma ( \alpha +3 ) } \\ &{}\times \sum_{r=2}^{p} \begin{bmatrix} \widetilde{I_{T}} ( t_{r},S^{r},I^{r},I_{A}^{r},I_{D}^{ r},I_{R}^{r},I_{T}^{r},R^{r},D^{r},V^{r} ) \\ -2\widetilde{I_{T}} ( t_{r-1},S^{r-1},I^{r-1},I_{A}^{r-1},I_{D}^{r-1},I_{R}^{r-1},I_{T}^{r-1},R^{r-1},D^{r-1},V^{r-1} ) \\ +\widetilde{I_{T}} ( t_{r-2},S^{r-2},I^{r-2},I_{A}^{r-2},I_{D}^{r-2},I_{R}^{r-2},I_{T}^{r-2},R^{r-2},D^{r-2},V^{r-2} )\end{bmatrix} \\ &{}\times \begin{bmatrix} ( p-r+1 ) ^{\alpha } \begin{bmatrix} 2 ( p-r ) ^{2}+ ( 3\alpha +10 ) ( p-r ) \\ +2\alpha ^{2}+9\alpha +12\end{bmatrix} \\ - ( p-r ) ^{\alpha } \begin{bmatrix} 2 ( p-r ) ^{2}+ ( 5\alpha +10 ) ( p-r ) \\ +6\alpha ^{2}+18\alpha +12\end{bmatrix}\end{bmatrix}, \\ R^{p+1} ={}& \frac{1-\alpha }{AB ( \alpha ) }\widetilde{R} \bigl( t_{p},S^{p},I^{p},I_{A}^{p},I_{D}^{p},I_{R}^{ p},I_{T}^{p},R^{p},D^{p},V^{p} \bigr) \\ &{}+ \frac{\alpha ( \Delta t ) ^{\alpha }}{AB ( \alpha ) \Gamma ( \alpha +1 ) }\sum_{r=2}^{p} \widetilde{R} \bigl( t_{r-2},S^{r-2},I^{r-2},I_{A}^{ r-2},I_{D}^{r-2},I_{R}^{r-2},I_{T}^{r-2},R^{r-2},D^{r-2},V^{r-2} \bigr) \\ &{}\times \bigl[ ( p-r+1 ) ^{\alpha }- ( p-r ) ^{ \alpha } \bigr] \\ &{}+ \frac{\alpha ( \Delta t ) ^{\alpha }}{AB ( \alpha ) \Gamma ( \alpha +2 ) }\sum_{r=2}^{p} \begin{bmatrix} \widetilde{R} ( t_{r-1},S^{r-1},I^{r-1},I_{A}^{r-1},I_{D}^{ r-1},I_{R}^{r-1},I_{T}^{r-1},R^{r-1},D^{r-1},V^{r-1} ) \\ -\widetilde{R} ( t_{r-2},S^{r-2},I^{r-2},I_{A}^{r-2},I_{D}^{r-2},I_{R}^{r-2},I_{T}^{r-2},R^{r-2},D^{r-2},V^{r-2} )\end{bmatrix} \\ &{}\times \begin{bmatrix} ( p-r+1 ) ^{\alpha } ( p-r+3+2\alpha ) \\ - ( p-r ) ^{\alpha } ( p-r+3+3\alpha )\end{bmatrix} \\ &{}+ \frac{\alpha ( \Delta t ) ^{\alpha }}{2AB ( \alpha ) \Gamma ( \alpha +3 ) } \\ &{}\times \sum_{r=2}^{p} \begin{bmatrix} \widetilde{R} ( t_{r},S^{r},I^{r},I_{A}^{r},I_{D}^{ r},I_{R}^{r},I_{T}^{r},R^{r},D^{r},V^{r} ) \\ -2\widetilde{R} ( t_{r-1},S^{r-1},I^{r-1},I_{A}^{r-1},I_{D}^{r-1},I_{R}^{r-1},I_{T}^{r-1},R^{r-1},D^{r-1},V^{r-1} ) \\ +\widetilde{R} ( t_{r-2},S^{r-2},I^{r-2},I_{A}^{r-2},I_{D}^{r-2},I_{R}^{r-2},I_{T}^{r-2},R^{r-2},D^{r-2},V^{r-2} )\end{bmatrix} \\ &{}\times \begin{bmatrix} ( p-r+1 ) ^{\alpha } \begin{bmatrix} 2 ( p-r ) ^{2}+ ( 3\alpha +10 ) ( p-r ) \\ +2\alpha ^{2}+9\alpha +12\end{bmatrix} \\ - ( p-r ) ^{\alpha } \begin{bmatrix} 2 ( p-r ) ^{2}+ ( 5\alpha +10 ) ( p-r ) \\ +6\alpha ^{2}+18\alpha +12\end{bmatrix}\end{bmatrix}, \\ D^{p+1} ={}& \frac{1-\alpha }{AB ( \alpha ) }\widetilde{D} \bigl( t_{p},S^{p},I^{p},I_{A}^{p},I_{D}^{p},I_{R}^{ p},I_{T}^{p},R^{p},D^{p},V^{p} \bigr) \\ &{}+ \frac{\alpha ( \Delta t ) ^{\alpha }}{AB ( \alpha ) \Gamma ( \alpha +1 ) }\sum_{r=2}^{p} \widetilde{D} \bigl( t_{r-2},S^{r-2},I^{r-2},I_{A}^{ r-2},I_{D}^{r-2},I_{R}^{r-2},I_{T}^{r-2},R^{r-2},D^{r-2},V^{r-2} \bigr) \\ &{}\times \bigl[ ( p-r+1 ) ^{\alpha }- ( p-r ) ^{ \alpha } \bigr] \\ &{}+ \frac{\alpha ( \Delta t ) ^{\alpha }}{AB ( \alpha ) \Gamma ( \alpha +2 ) } \sum_{r=2}^{p} \begin{bmatrix} \widetilde{D} ( t_{r-1},S^{r-1},I^{r-1},I_{A}^{r-1},I_{D}^{ r-1},I_{R}^{r-1},I_{T}^{r-1},R^{r-1},D^{r-1},V^{r-1} ) \\ -\widetilde{D} ( t_{r-2},S^{r-2},I^{r-2},I_{A}^{r-2},I_{D}^{r-2},I_{R}^{r-2},I_{T}^{r-2},R^{r-2},D^{r-2},V^{r-2} )\end{bmatrix} \\ &{}\times \begin{bmatrix} ( p-r+1 ) ^{\alpha } ( p-r+3+2\alpha ) \\ - ( p-r ) ^{\alpha } ( p-r+3+3\alpha )\end{bmatrix} \\ &{}+ \frac{\alpha ( \Delta t ) ^{\alpha }}{2AB ( \alpha ) \Gamma ( \alpha +3 ) } \\ &{}\times \sum_{r=2}^{p} \begin{bmatrix} \widetilde{D} ( t_{r},S^{r},I^{r},I_{A}^{r},I_{D}^{ r},I_{R}^{r},I_{T}^{r},R^{r},D^{r},V^{r} ) \\ -2\widetilde{D} ( t_{r-1},S^{r-1},I^{r-1},I_{A}^{r-1},I_{D}^{r-1},I_{R}^{r-1},I_{T}^{r-1},R^{r-1},D^{r-1},V^{r-1} ) \\ +\widetilde{D} ( t_{r-2},S^{r-2},I^{r-2},I_{A}^{r-2},I_{D}^{r-2},I_{R}^{r-2},I_{T}^{r-2},R^{r-2},D^{r-2},V^{r-2} )\end{bmatrix} \end{aligned}$$

$$\begin{aligned} &{}\times \begin{bmatrix} ( p-r+1 ) ^{\alpha } \begin{bmatrix} 2 ( p-r ) ^{2}+ ( 3\alpha +10 ) ( p-r ) \\ +2\alpha ^{2}+9\alpha +12\end{bmatrix} \\ - ( p-r ) ^{\alpha } \begin{bmatrix} 2 ( p-r ) ^{2}+ ( 5\alpha +10 ) ( p-r ) \\ +6\alpha ^{2}+18\alpha +12\end{bmatrix}\end{bmatrix}, \\ V^{p+1} ={}& \frac{1-\alpha }{AB ( \alpha ) }\widetilde{V} \bigl( t_{p},S^{p},I^{p},I_{A}^{p},I_{D}^{p},I_{R}^{ p},I_{T}^{p},R^{p},D^{p},V^{p} \bigr) \\ &{}+ \frac{\alpha ( \Delta t ) ^{\alpha }}{AB ( \alpha ) \Gamma ( \alpha +1 ) }\sum_{r=2}^{p} \widetilde{V} \bigl( t_{r-2},S^{r-2},I^{r-2},I_{A}^{ r-2},I_{D}^{r-2},I_{R}^{r-2},I_{T}^{r-2},R^{r-2},D^{r-2},V^{r-2} \bigr) \\ &{}\times \bigl[ ( p-r+1 ) ^{\alpha }- ( p-r ) ^{ \alpha } \bigr] \\ &{}+ \frac{\alpha ( \Delta t ) ^{\alpha }}{AB ( \alpha ) \Gamma ( \alpha +2 ) }\sum_{r=2}^{p} \begin{bmatrix} \widetilde{V} ( t_{r-1},S^{r-1},I^{r-1},I_{A}^{r-1},I_{D}^{ r-1},I_{R}^{r-1},I_{T}^{r-1},R^{r-1},D^{r-1},V^{r-1} ) \\ -\widetilde{V} ( t_{r-2},S^{r-2},I^{r-2},I_{A}^{r-2},I_{D}^{r-2},I_{R}^{r-2},I_{T}^{r-2},R^{r-2},D^{r-2},V^{r-2} )\end{bmatrix} \\ &{}\times \begin{bmatrix} ( p-r+1 ) ^{\alpha } ( p-r+3+2\alpha ) \\ - ( p-r ) ^{\alpha } ( p-r+3+3\alpha )\end{bmatrix} \\ &{}+ \frac{\alpha ( \Delta t ) ^{\alpha }}{2AB ( \alpha ) \Gamma ( \alpha +3 ) } \\ &{}\times \sum_{r=2}^{p} \begin{bmatrix} \widetilde{V} ( t_{r},S^{r},I^{r},I_{A}^{r},I_{D}^{ r},I_{R}^{r},I_{T}^{r},R^{r},D^{r},V^{r} ) \\ -2\widetilde{V} ( t_{r-1},S^{r-1},I^{r-1},I_{A}^{r-1},I_{D}^{r-1},I_{R}^{r-1},I_{T}^{r-1},R^{r-1},D^{r-1},V^{r-1} ) \\ +\widetilde{V} ( t_{r-2},S^{r-2},I^{r-2},I_{A}^{r-2},I_{D}^{r-2},I_{R}^{r-2},I_{T}^{r-2},R^{r-2},D^{r-2},V^{r-2} )\end{bmatrix} \\ &{}\times \begin{bmatrix} ( p-r+1 ) ^{\alpha } \begin{bmatrix} 2 ( p-r ) ^{2}+ ( 3\alpha +10 ) ( p-r ) \\ +2\alpha ^{2}+9\alpha +12\end{bmatrix} \\ - ( p-r ) ^{\alpha } \begin{bmatrix} 2 ( p-r ) ^{2}+ ( 5\alpha +10 ) ( p-r ) \\ +6\alpha ^{2}+18\alpha +12\end{bmatrix}\end{bmatrix}. \end{aligned}$$

For the power-law kernel, we have the following:

5.17 $$\begin{aligned} &S^{p+1}=\frac{1}{\Gamma ( \alpha ) }\sum_{r=2}^{p}\int _{t_{r}}^{t_{r+1}}\widetilde{S} ( \tau,S,I,I_{A},I_{D},I_{R},I_{T},R,D,V ) ( t_{p+1}-\tau ) ^{\alpha -1}\,d\tau, \\ &I^{p+1}=\frac{1}{\Gamma ( \alpha ) }\sum_{r=2}^{p}\int _{t_{r}}^{t_{r+1}}\widetilde{I} ( \tau,S,I,I_{A},I_{D},I_{R},I_{T},R,D,V ) ( t_{p+1}-\tau ) ^{\alpha -1}\,d\tau, \\ &I_{A}^{p+1}=\frac{1}{\Gamma ( \alpha ) }\sum _{r=2}^{p}\int _{t_{r}}^{t_{r+1}}\widetilde{I_{A}} ( \tau,S,I,I_{A},I_{D},I_{R},I_{T},R,D,V ) ( t_{p+1}-\tau ) ^{\alpha -1}\,d\tau, \\ &I_{D}^{p+1}=\frac{1}{\Gamma ( \alpha ) }\sum _{r=2}^{p}\int _{t_{r}}^{t_{r+1}}\widetilde{I_{D}} ( \tau,S,I,I_{A},I_{D},I_{R},I_{T},R,D,V ) ( t_{p+1}-\tau ) ^{\alpha -1}\,d\tau, \\ &I_{R}^{p+1}=\frac{1}{\Gamma ( \alpha ) }\sum _{r=2}^{p}\int _{t_{r}}^{t_{r+1}}\widetilde{I_{R}} ( \tau,S,I,I_{A},I_{D},I_{R},I_{T},R,D,V ) ( t_{p+1}-\tau ) ^{\alpha -1}\,d\tau, \\ &I_{T}^{p+1}=\frac{1}{\Gamma ( \alpha ) }\sum _{r=2}^{p}\int _{t_{r}}^{t_{r+1}}\widetilde{I_{T}} ( \tau,S,I,I_{A},I_{D},I_{R},I_{T},R,D,V ) ( t_{p+1}-\tau ) ^{\alpha -1}\,d\tau, \\ &R^{p+1}=\frac{1}{\Gamma ( \alpha ) }\sum_{r=2}^{p}\int _{t_{r}}^{t_{r+1}}\widetilde{R} ( \tau,S,I,I_{A},I_{D},I_{R},I_{T},R,D,V ) ( t_{p+1}-\tau ) ^{\alpha -1}\,d\tau, \\ &D^{p+1}=\frac{1}{\Gamma ( \alpha ) }\sum_{r=2}^{p}\int _{t_{r}}^{t_{r+1}}\widetilde{D} ( \tau,S,I,I_{A},I_{D},I_{R},I_{T},R,D,V ) ( t_{p+1}-\tau ) ^{\alpha -1}\,d\tau, \\ &V^{p+1}=\frac{1}{\Gamma ( \alpha ) }\sum_{r=2}^{p}\int _{t_{r}}^{t_{r+1}}\widetilde{V} ( \tau,S,I,I_{A},I_{D},I_{R},I_{T},R,D,V ) ( t_{p+1}-\tau ) ^{\alpha -1}\,d\tau. \end{aligned}$$

We can get the following numerical scheme:

5.18 $$\begin{aligned} S^{p+1} ={}& \frac{ ( \Delta t ) ^{\alpha }}{\Gamma ( \alpha +1 ) } \sum_{r=2}^{p} \widetilde{S} \bigl( t_{r-2},S^{r-2},I^{r-2},I_{A}^{r-2},I_{D}^{r-2},I_{R}^{r-2},I_{T}^{r-2},R^{r-2},D^{r-2},V^{r-2} \bigr) \\ &{}\times \bigl[ ( p-r+1 ) ^{\alpha }- ( p-r ) ^{ \alpha } \bigr] \\ &{}+ \frac{ ( \Delta t ) ^{\alpha }}{\Gamma ( \alpha +2 ) }\sum_{r=2}^{p} \begin{bmatrix} \widetilde{S} ( t_{r-1},S^{r-1},I^{r-1},I_{A}^{r-1},I_{D}^{ r-1},I_{R}^{r-1},I_{T}^{r-1},R^{r-1},D^{r-1},V^{r-1} ) \\ -\widetilde{S} ( t_{r-2},S^{r-2},I^{r-2},I_{A}^{r-2},I_{D}^{r-2},I_{R}^{r-2},I_{T}^{r-2},R^{r-2},D^{r-2},V^{r-2} )\end{bmatrix} \\ &{}\times \begin{bmatrix} ( p-r+1 ) ^{\alpha } ( p-r+3+2\alpha ) \\ - ( p-r ) ^{\alpha } ( p-r+3+3\alpha )\end{bmatrix} \\ &{}+ \frac{ ( \Delta t ) ^{\alpha }}{2\Gamma ( \alpha +3 ) }\sum_{r=2}^{p} \begin{bmatrix} \widetilde{S} ( t_{r},S^{r},I^{r},I_{A}^{r},I_{D}^{ r},I_{R}^{r},I_{T}^{r},R^{r},D^{r},V^{r} ) \\ -2\widetilde{S} ( t_{r-1},S^{r-1},I^{r-1},I_{A}^{r-1},I_{D}^{r-1},I_{R}^{r-1},I_{T}^{r-1},R^{r-1},D^{r-1},V^{r-1} ) \\ +\widetilde{S} ( t_{r-2},S^{r-2},I^{r-2},I_{A}^{r-2},I_{D}^{r-2},I_{R}^{r-2},I_{T}^{r-2},R^{r-2},D^{r-2},V^{r-2} )\end{bmatrix} \\ &{}\times \begin{bmatrix} ( p-r+1 ) ^{\alpha } \begin{bmatrix} 2 ( p-r ) ^{2}+ ( 3\alpha +10 ) ( p-r ) \\ +2\alpha ^{2}+9\alpha +12\end{bmatrix} \\ - ( p-r ) ^{\alpha } \begin{bmatrix} 2 ( p-r ) ^{2}+ ( 5\alpha +10 ) ( p-r ) \\ +6\alpha ^{2}+18\alpha +12\end{bmatrix}\end{bmatrix}, \\ I^{p+1} ={}& \frac{ ( \Delta t ) ^{\alpha }}{\Gamma ( \alpha +1 ) } \sum_{r=2}^{p} \widetilde{I} \bigl( t_{r-2},S^{r-2},I^{r-2},I_{A}^{r-2},I_{D}^{r-2},I_{R}^{r-2},I_{T}^{r-2},R^{r-2},D^{r-2},V^{r-2} \bigr) \\ &{}\times \bigl[ ( p-r+1 ) ^{\alpha }- ( p-r ) ^{ \alpha } \bigr] \\ &{}+ \frac{ ( \Delta t ) ^{\alpha }}{\Gamma ( \alpha +2 ) }\sum_{r=2}^{p} \begin{bmatrix} \widetilde{I} ( t_{r-1},S^{r-1},I^{r-1},I_{A}^{r-1},I_{D}^{ r-1},I_{R}^{r-1},I_{T}^{r-1},R^{r-1},D^{r-1},V^{r-1} ) \\ -\widetilde{I} ( t_{r-2},S^{r-2},I^{r-2},I_{A}^{r-2},I_{D}^{r-2},I_{R}^{r-2},I_{T}^{r-2},R^{r-2},D^{r-2},V^{r-2} )\end{bmatrix} \\ &{}\times \begin{bmatrix} ( p-r+1 ) ^{\alpha } ( p-r+3+2\alpha ) \\ - ( p-r ) ^{\alpha } ( p-r+3+3\alpha )\end{bmatrix} \\ &{}+ \frac{ ( \Delta t ) ^{\alpha }}{2\Gamma ( \alpha +3 ) }\sum_{r=2}^{p} \begin{bmatrix} \widetilde{I} ( t_{r},S^{r},I^{r},I_{A}^{r},I_{D}^{ r},I_{R}^{r},I_{T}^{r},R^{r},D^{r},V^{r} ) \\ -2\widetilde{I} ( t_{r-1},S^{r-1},I^{r-1},I_{A}^{r-1},I_{D}^{r-1},I_{R}^{r-1},I_{T}^{r-1},R^{r-1},D^{r-1},V^{r-1} ) \\ +\widetilde{I} ( t_{r-2},S^{r-2},I^{r-2},I_{A}^{r-2},I_{D}^{r-2},I_{R}^{r-2},I_{T}^{r-2},R^{r-2},D^{r-2},V^{r-2} )\end{bmatrix} \\ &{}\times \begin{bmatrix} ( p-r+1 ) ^{\alpha } \begin{bmatrix} 2 ( p-r ) ^{2}+ ( 3\alpha +10 ) ( p-r ) \\ +2\alpha ^{2}+9\alpha +12\end{bmatrix} \\ - ( p-r ) ^{\alpha } \begin{bmatrix} 2 ( p-r ) ^{2}+ ( 5\alpha +10 ) ( p-r ) \\ +6\alpha ^{2}+18\alpha +12\end{bmatrix}\end{bmatrix}, \\ I_{A}^{p+1} ={}& \frac{ ( \Delta t ) ^{\alpha }}{\Gamma ( \alpha +1 ) } \sum _{r=2}^{p}\widetilde{I_{A}} \bigl( t_{r-2},S^{r-2},I^{r-2},I_{A}^{ r-2},I_{D}^{r-2},I_{R}^{r-2},I_{T}^{r-2},R^{r-2},D^{r-2},V^{r-2} \bigr) \\ &{}\times \bigl[ ( p-r+1 ) ^{\alpha }- ( p-r ) ^{ \alpha } \bigr] \\ &{}+ \frac{ ( \Delta t ) ^{\alpha }}{\Gamma ( \alpha +2 ) }\sum_{r=2}^{p} \begin{bmatrix} \widetilde{I_{A}} ( t_{r-1},S^{r-1},I^{r-1},I_{A}^{r-1},I_{D}^{r-1},I_{R}^{r-1},I_{T}^{r-1},R^{r-1},D^{r-1},V^{r-1} ) \\ -\widetilde{I_{A}} ( t_{r-2},S^{r-2},I^{r-2},I_{A}^{r-2},I_{D}^{r-2},I_{R}^{r-2},I_{T}^{r-2},R^{r-2},D^{r-2},V^{r-2} )\end{bmatrix} \\ &{}\times \begin{bmatrix} ( p-r+1 ) ^{\alpha } ( p-r+3+2\alpha ) \\ - ( p-r ) ^{\alpha } ( p-r+3+3\alpha )\end{bmatrix} \\ &{}+ \frac{ ( \Delta t ) ^{\alpha }}{2\Gamma ( \alpha +3 ) }\sum_{r=2}^{p} \begin{bmatrix} \widetilde{I_{A}} ( t_{r},S^{r},I^{r},I_{A}^{r},I_{D}^{ r},I_{R}^{r},I_{T}^{r},R^{r},D^{r},V^{r} ) \\ -2\widetilde{I_{A}} ( t_{r-1},S^{r-1},I^{r-1},I_{A}^{r-1},I_{D}^{r-1},I_{R}^{r-1},I_{T}^{r-1},R^{r-1},D^{r-1},V^{r-1} ) \\ +\widetilde{I_{A}} ( t_{r-2},S^{r-2},I^{r-2},I_{A}^{r-2},I_{D}^{r-2},I_{R}^{r-2},I_{T}^{r-2},R^{r-2},D^{r-2},V^{r-2} )\end{bmatrix} \\ &{}\times \begin{bmatrix} ( p-r+1 ) ^{\alpha } \begin{bmatrix} 2 ( p-r ) ^{2}+ ( 3\alpha +10 ) ( p-r ) \\ +2\alpha ^{2}+9\alpha +12\end{bmatrix} \\ - ( p-r ) ^{\alpha } \begin{bmatrix} 2 ( p-r ) ^{2}+ ( 5\alpha +10 ) ( p-r ) \\ +6\alpha ^{2}+18\alpha +12\end{bmatrix}\end{bmatrix}, \\ I_{D}^{p+1} ={}& \frac{ ( \Delta t ) ^{\alpha }}{\Gamma ( \alpha +1 ) } \sum _{r=2}^{p}\widetilde{I_{D}} \bigl( t_{r-2},S^{r-2},I^{r-2},I_{A}^{ r-2},I_{D}^{r-2},I_{R}^{r-2},I_{T}^{r-2},R^{r-2},D^{r-2},V^{r-2} \bigr) \\ &{}\times \bigl[ ( p-r+1 ) ^{\alpha }- ( p-r ) ^{ \alpha } \bigr] \\ &{}+ \frac{ ( \Delta t ) ^{\alpha }}{\Gamma ( \alpha +2 ) }\sum_{r=2}^{p} \begin{bmatrix} \widetilde{I_{D}} ( t_{r-1},S^{r-1},I^{r-1},I_{A}^{r-1},I_{D}^{r-1},I_{R}^{r-1},I_{T}^{r-1},R^{r-1},D^{r-1},V^{r-1} ) \\ -\widetilde{I_{D}} ( t_{r-2},S^{r-2},I^{r-2},I_{A}^{r-2},I_{D}^{r-2},I_{R}^{r-2},I_{T}^{r-2},R^{r-2},D^{r-2},V^{r-2} )\end{bmatrix} \\ &{}\times \begin{bmatrix} ( p-r+1 ) ^{\alpha } ( p-r+3+2\alpha ) \\ - ( p-r ) ^{\alpha } ( p-r+3+3\alpha )\end{bmatrix} \\ &{}+ \frac{ ( \Delta t ) ^{\alpha }}{2\Gamma ( \alpha +3 ) }\sum_{r=2}^{p} \begin{bmatrix} \widetilde{I_{D}} ( t_{r},S^{r},I^{r},I_{A}^{r},I_{D}^{ r},I_{R}^{r},I_{T}^{r},R^{r},D^{r},V^{r} ) \\ -2\widetilde{I_{D}} ( t_{r-1},S^{r-1},I^{r-1},I_{A}^{r-1},I_{D}^{r-1},I_{R}^{r-1},I_{T}^{r-1},R^{r-1},D^{r-1},V^{r-1} ) \\ +\widetilde{I_{D}} ( t_{r-2},S^{r-2},I^{r-2},I_{A}^{r-2},I_{D}^{r-2},I_{R}^{r-2},I_{T}^{r-2},R^{r-2},D^{r-2},V^{r-2} )\end{bmatrix} \\ &{}\times \begin{bmatrix} ( p-r+1 ) ^{\alpha } \begin{bmatrix} 2 ( p-r ) ^{2}+ ( 3\alpha +10 ) ( p-r ) \\ +2\alpha ^{2}+9\alpha +12\end{bmatrix} \\ - ( p-r ) ^{\alpha } \begin{bmatrix} 2 ( p-r ) ^{2}+ ( 5\alpha +10 ) ( p-r ) \\ +6\alpha ^{2}+18\alpha +12\end{bmatrix}\end{bmatrix}, \\ I_{R}^{p+1} = {}&\frac{ ( \Delta t ) ^{\alpha }}{\Gamma ( \alpha +1 ) } \sum _{r=2}^{p}\widetilde{I_{R}} \bigl( t_{r-2},S^{r-2},I^{r-2},I_{A}^{ r-2},I_{D}^{r-2},I_{R}^{r-2},I_{T}^{r-2},R^{r-2},D^{r-2},V^{r-2} \bigr) \\ &{}\times \bigl[ ( p-r+1 ) ^{\alpha }- ( p-r ) ^{ \alpha } \bigr] \\ &{}+ \frac{ ( \Delta t ) ^{\alpha }}{\Gamma ( \alpha +2 ) }\sum_{r=2}^{p} \begin{bmatrix} \widetilde{I_{R}} ( t_{r-1},S^{r-1},I^{r-1},I_{A}^{r-1},I_{D}^{r-1},I_{R}^{r-1},I_{T}^{r-1},R^{r-1},D^{r-1},V^{r-1} ) \\ -\widetilde{I_{R}} ( t_{r-2},S^{r-2},I^{r-2},I_{A}^{r-2},I_{D}^{r-2},I_{R}^{r-2},I_{T}^{r-2},R^{r-2},D^{r-2},V^{r-2} )\end{bmatrix} \\ &{}\times \begin{bmatrix} ( p-r+1 ) ^{\alpha } ( p-r+3+2\alpha ) \\ - ( p-r ) ^{\alpha } ( p-r+3+3\alpha )\end{bmatrix} \\ &{}+ \frac{ ( \Delta t ) ^{\alpha }}{2\Gamma ( \alpha +3 ) }\sum_{r=2}^{p} \begin{bmatrix} \widetilde{I_{R}} ( t_{r},S^{r},I^{r},I_{A}^{r},I_{D}^{ r},I_{R}^{r},I_{T}^{r},R^{r},D^{r},V^{r} ) \\ -2\widetilde{I_{R}} ( t_{r-1},S^{r-1},I^{r-1},I_{A}^{r-1},I_{D}^{r-1},I_{R}^{r-1},I_{T}^{r-1},R^{r-1},D^{r-1},V^{r-1} ) \\ +\widetilde{I_{R}} ( t_{r-2},S^{r-2},I^{r-2},I_{A}^{r-2},I_{D}^{r-2},I_{R}^{r-2},I_{T}^{r-2},R^{r-2},D^{r-2},V^{r-2} )\end{bmatrix} \\ &{}\times \begin{bmatrix} ( p-r+1 ) ^{\alpha } \begin{bmatrix} 2 ( p-r ) ^{2}+ ( 3\alpha +10 ) ( p-r ) \\ +2\alpha ^{2}+9\alpha +12\end{bmatrix} \\ - ( p-r ) ^{\alpha } \begin{bmatrix} 2 ( p-r ) ^{2}+ ( 5\alpha +10 ) ( p-r ) \\ +6\alpha ^{2}+18\alpha +12\end{bmatrix}\end{bmatrix}, \\ I_{T}^{p+1} ={}& \frac{\alpha ( \Delta t ) ^{\alpha }}{\Gamma ( \alpha +1 ) }\sum _{r=2}^{p}\widetilde{I_{T}} \bigl( t_{r-2},S^{r-2},I^{r-2},I_{A}^{ r-2},I_{D}^{r-2},I_{R}^{r-2},I_{T}^{r-2},R^{r-2},D^{r-2},V^{r-2} \bigr) \\ &{}\times \bigl[ ( p-r+1 ) ^{\alpha }- ( p-r ) ^{ \alpha } \bigr] \\ &{}+ \frac{ ( \Delta t ) ^{\alpha }}{\Gamma ( \alpha +2 ) }\sum_{r=2}^{p} \begin{bmatrix} \widetilde{I_{T}} ( t_{r-1},S^{r-1},I^{r-1},I_{A}^{r-1},I_{D}^{r-1},I_{R}^{r-1},I_{T}^{r-1},R^{r-1},D^{r-1},V^{r-1} ) \\ -\widetilde{I_{T}} ( t_{r-2},S^{r-2},I^{r-2},I_{A}^{r-2},I_{D}^{r-2},I_{R}^{r-2},I_{T}^{r-2},R^{r-2},D^{r-2},V^{r-2} )\end{bmatrix} \\ &{}\times \begin{bmatrix} ( p-r+1 ) ^{\alpha } ( p-r+3+2\alpha ) \\ - ( p-r ) ^{\alpha } ( p-r+3+3\alpha )\end{bmatrix} \\ &{}+ \frac{ ( \Delta t ) ^{\alpha }}{2\Gamma ( \alpha +3 ) }\sum_{r=2}^{p} \begin{bmatrix} \widetilde{I_{T}} ( t_{r},S^{r},I^{r},I_{A}^{r},I_{D}^{ r},I_{R}^{r},I_{T}^{r},R^{r},D^{r},V^{r} ) \\ -2\widetilde{I_{T}} ( t_{r-1},S^{r-1},I^{r-1},I_{A}^{r-1},I_{D}^{r-1},I_{R}^{r-1},I_{T}^{r-1},R^{r-1},D^{r-1},V^{r-1} ) \\ +\widetilde{I_{T}} ( t_{r-2},S^{r-2},I^{r-2},I_{A}^{r-2},I_{D}^{r-2},I_{R}^{r-2},I_{T}^{r-2},R^{r-2},D^{r-2},V^{r-2} )\end{bmatrix} \\ &{}\times \begin{bmatrix} ( p-r+1 ) ^{\alpha } \begin{bmatrix} 2 ( p-r ) ^{2}+ ( 3\alpha +10 ) ( p-r ) \\ +2\alpha ^{2}+9\alpha +12\end{bmatrix} \\ - ( p-r ) ^{\alpha } \begin{bmatrix} 2 ( p-r ) ^{2}+ ( 5\alpha +10 ) ( p-r ) \\ +6\alpha ^{2}+18\alpha +12\end{bmatrix}\end{bmatrix}, \\ R^{p+1} ={}& \frac{ ( \Delta t ) ^{\alpha }}{\Gamma ( \alpha +1 ) } \sum_{r=2}^{p} \widetilde{R} \bigl( t_{r-2},S^{r-2},I^{r-2},I_{A}^{r-2},I_{D}^{r-2},I_{R}^{r-2},I_{T}^{r-2},R^{r-2},D^{r-2},V^{r-2} \bigr) \\ &{}\times \bigl[ ( p-r+1 ) ^{\alpha }- ( p-r ) ^{ \alpha } \bigr] \\ &{}+ \frac{ ( \Delta t ) ^{\alpha }}{\Gamma ( \alpha +2 ) }\sum_{r=2}^{p} \begin{bmatrix} \widetilde{R} ( t_{r-1},S^{r-1},I^{r-1},I_{A}^{r-1},I_{D}^{ r-1},I_{R}^{r-1},I_{T}^{r-1},R^{r-1},D^{r-1},V^{r-1} ) \\ -\widetilde{R} ( t_{r-2},S^{r-2},I^{r-2},I_{A}^{r-2},I_{D}^{r-2},I_{R}^{r-2},I_{T}^{r-2},R^{r-2},D^{r-2},V^{r-2} )\end{bmatrix} \\ &{}\times \begin{bmatrix} ( p-r+1 ) ^{\alpha } ( p-r+3+2\alpha ) \\ - ( p-r ) ^{\alpha } ( p-r+3+3\alpha )\end{bmatrix} \\ &{}+ \frac{ ( \Delta t ) ^{\alpha }}{2\Gamma ( \alpha +3 ) }\sum_{r=2}^{p} \begin{bmatrix} \widetilde{R} ( t_{r},S^{r},I^{r},I_{A}^{r},I_{D}^{ r},I_{R}^{r},I_{T}^{r},R^{r},D^{r},V^{r} ) \\ -2\widetilde{R} ( t_{r-1},S^{r-1},I^{r-1},I_{A}^{r-1},I_{D}^{r-1},I_{R}^{r-1},I_{T}^{r-1},R^{r-1},D^{r-1},V^{r-1} ) \\ +\widetilde{R} ( t_{r-2},S^{r-2},I^{r-2},I_{A}^{r-2},I_{D}^{r-2},I_{R}^{r-2},I_{T}^{r-2},R^{r-2},D^{r-2},V^{r-2} )\end{bmatrix} \\ &{}\times \begin{bmatrix} ( p-r+1 ) ^{\alpha } \begin{bmatrix} 2 ( p-r ) ^{2}+ ( 3\alpha +10 ) ( p-r ) \\ +2\alpha ^{2}+9\alpha +12\end{bmatrix} \\ - ( p-r ) ^{\alpha } \begin{bmatrix} 2 ( p-r ) ^{2}+ ( 5\alpha +10 ) ( p-r ) \\ +6\alpha ^{2}+18\alpha +12\end{bmatrix}\end{bmatrix}, \\ D^{p+1} ={}& \frac{ ( \Delta t ) ^{\alpha }}{\Gamma ( \alpha +1 ) } \sum_{r=2}^{p} \widetilde{D} \bigl( t_{r-2},S^{r-2},I^{r-2},I_{A}^{r-2},I_{D}^{r-2},I_{R}^{r-2},I_{T}^{r-2},R^{r-2},D^{r-2},V^{r-2} \bigr) \\ &{}\times \bigl[ ( p-r+1 ) ^{\alpha }- ( p-r ) ^{ \alpha } \bigr] \\ &{}+ \frac{ ( \Delta t ) ^{\alpha }}{\Gamma ( \alpha +2 ) }\sum_{r=2}^{p} \begin{bmatrix} \widetilde{D} ( t_{r-1},S^{r-1},I^{r-1},I_{A}^{r-1},I_{D}^{ r-1},I_{R}^{r-1},I_{T}^{r-1},R^{r-1},D^{r-1},V^{r-1} ) \\ -\widetilde{D} ( t_{r-2},S^{r-2},I^{r-2},I_{A}^{r-2},I_{D}^{r-2},I_{R}^{r-2},I_{T}^{r-2},R^{r-2},D^{r-2},V^{r-2} )\end{bmatrix} \\ &{}\times \begin{bmatrix} ( p-r+1 ) ^{\alpha } ( p-r+3+2\alpha ) \\ - ( p-r ) ^{\alpha } ( p-r+3+3\alpha )\end{bmatrix} \\ &{}+ \frac{ ( \Delta t ) ^{\alpha }}{2\Gamma ( \alpha +3 ) }\sum_{r=2}^{p} \begin{bmatrix} \widetilde{D} ( t_{r},S^{r},I^{r},I_{A}^{r},I_{D}^{ r},I_{R}^{r},I_{T}^{r},R^{r},D^{r},V^{r} ) \\ -2\widetilde{D} ( t_{r-1},S^{r-1},I^{r-1},I_{A}^{r-1},I_{D}^{r-1},I_{R}^{r-1},I_{T}^{r-1},R^{r-1},D^{r-1},V^{r-1} ) \\ +\widetilde{D} ( t_{r-2},S^{r-2},I^{r-2},I_{A}^{r-2},I_{D}^{r-2},I_{R}^{r-2},I_{T}^{r-2},R^{r-2},D^{r-2},V^{r-2} )\end{bmatrix} \\ &{}\times \begin{bmatrix} ( p-r+1 ) ^{\alpha } \begin{bmatrix} 2 ( p-r ) ^{2}+ ( 3\alpha +10 ) ( p-r ) \\ +2\alpha ^{2}+9\alpha +12\end{bmatrix} \\ - ( p-r ) ^{\alpha } \begin{bmatrix} 2 ( p-r ) ^{2}+ ( 5\alpha +10 ) ( p-r ) \\ +6\alpha ^{2}+18\alpha +12\end{bmatrix}\end{bmatrix}, \\ V^{p+1} = {}&\frac{ ( \Delta t ) ^{\alpha }}{\Gamma ( \alpha +1 ) } \sum_{r=2}^{p} \widetilde{V} \bigl( t_{r-2},S^{r-2},I^{r-2},I_{A}^{r-2},I_{D}^{r-2},I_{R}^{r-2},I_{T}^{r-2},R^{r-2},D^{r-2},V^{r-2} \bigr) \\ &{}\times \bigl[ ( p-r+1 ) ^{\alpha }- ( p-r ) ^{ \alpha } \bigr] \\ &{}+ \frac{ ( \Delta t ) ^{\alpha }}{\Gamma ( \alpha +2 ) }\sum_{r=2}^{p} \begin{bmatrix} \widetilde{V} ( t_{r-1},S^{r-1},I^{r-1},I_{A}^{r-1},I_{D}^{ r-1},I_{R}^{r-1},I_{T}^{r-1},R^{r-1},D^{r-1},V^{r-1} ) \\ -\widetilde{V} ( t_{r-2},S^{r-2},I^{r-2},I_{A}^{r-2},I_{D}^{r-2},I_{R}^{r-2},I_{T}^{r-2},R^{r-2},D^{r-2},V^{r-2} )\end{bmatrix} \\ &{}\times \begin{bmatrix} ( p-r+1 ) ^{\alpha } ( p-r+3+2\alpha ) \\ - ( p-r ) ^{\alpha } ( p-r+3+3\alpha )\end{bmatrix} \\ &{}+ \frac{ ( \Delta t ) ^{\alpha }}{2\Gamma ( \alpha +3 ) }\sum_{r=2}^{p} \begin{bmatrix} \widetilde{V} ( t_{r},S^{r},I^{r},I_{A}^{r},I_{D}^{ r},I_{R}^{r},I_{T}^{r},R^{r},D^{r},V^{r} ) \\ -2\widetilde{V} ( t_{r-1},S^{r-1},I^{r-1},I_{A}^{r-1},I_{D}^{r-1},I_{R}^{r-1},I_{T}^{r-1},R^{r-1},D^{r-1},V^{r-1} ) \\ +\widetilde{V} ( t_{r-2},S^{r-2},I^{r-2},I_{A}^{r-2},I_{D}^{r-2},I_{R}^{r-2},I_{T}^{r-2},R^{r-2},D^{r-2},V^{r-2} )\end{bmatrix} \\ &{}\times \begin{bmatrix} ( p-r+1 ) ^{\alpha } \begin{bmatrix} 2 ( p-r ) ^{2}+ ( 3\alpha +10 ) ( p-r ) \\ +2\alpha ^{2}+9\alpha +12\end{bmatrix} \\ - ( p-r ) ^{\alpha } \begin{bmatrix} 2 ( p-r ) ^{2}+ ( 5\alpha +10 ) ( p-r ) \\ +6\alpha ^{2}+18\alpha +12\end{bmatrix}\end{bmatrix}. \end{aligned}$$

Now, we handle the following model:

5.19 $$\begin{aligned} &{}_{0}^{ABC}D_{t}^{\alpha }S = \Lambda - \bigl( \alpha ( x ) +\gamma _{1}+\mu _{1} \bigr) S, \\ &{}_{0}^{ABC}D_{t}^{\alpha }I = \alpha ( x ) S- ( \varepsilon +\xi +\lambda +\mu _{1} ) I, \\ &{}_{0}^{ABC}D_{t}^{\alpha }I_{A} = \xi I- ( \theta +\mu +\chi + \mu _{1} ) I_{A}, \\ &{}_{0}^{ABC}D_{t}^{\alpha }I_{D} = \varepsilon I- ( \eta + \varphi +\mu _{1} ) I_{D}, \\ &{}_{0}^{ABC}D_{t}^{\alpha }I_{R} = \eta I_{D}+\theta I_{A}- ( v+ \xi +\mu _{1} ) I_{R}, \\ &{}_{0}^{ABC}D_{t}^{\alpha }I_{T} = \mu I_{A}+vI_{R}- ( \sigma + \tau +\mu _{1} ) I_{T}, \\ &{}_{0}^{ABC}D_{t}^{\alpha }R = \lambda I+\varphi I_{D}+\chi I_{A}+ \xi I_{R}+\sigma I_{T}- ( \Phi +\mu _{1} ) R, \\ &{}_{0}^{ABC}D_{t}^{\alpha }D = \tau I_{T}, \\ &{}_{0}^{ABC}D_{t}^{\alpha }V = \gamma _{1}S+\Phi R-\mu _{1}V, \end{aligned}$$

where the initial conditions are

5.20 $$\begin{aligned} &S ( 0 ) = 57780000,\qquad I ( 0 ) =1,\qquad I_{A} ( 0 ) =1,\qquad I_{D} ( 0 ) =1,\qquad I_{R} ( 0 ) =1, \\ &I_{T} ( 0 ) = 1,\qquad R ( 0 ) =0,\qquad D ( 0 ) =0,\qquad V ( 0 ) =0. \end{aligned}$$

Also the parameters are chosen as follows:

5.21 $$\begin{aligned} \begin{aligned} &\Lambda = 57000000,\qquad k=3,\qquad p=0.5,\qquad \eta =0.12,\qquad \chi =0.015,\\ & v=0.027,\qquad x=0.4, \qquad \theta =0.301, \qquad \gamma = 0.09,\qquad \beta =0.013,\\&\gamma _{1}=0.4,\qquad\mu _{1}=0.3,\qquad \varepsilon =0.161, \qquad\xi =0.015,\qquad\sigma =0.015,\\ & \tau = 0.0199,\qquad\Phi =0.2,\qquad \lambda =0.0345,\qquad\varphi =0.0345,\qquad \delta _{1}=0.01. \end{aligned} \end{aligned}$$

**Figure 37:**
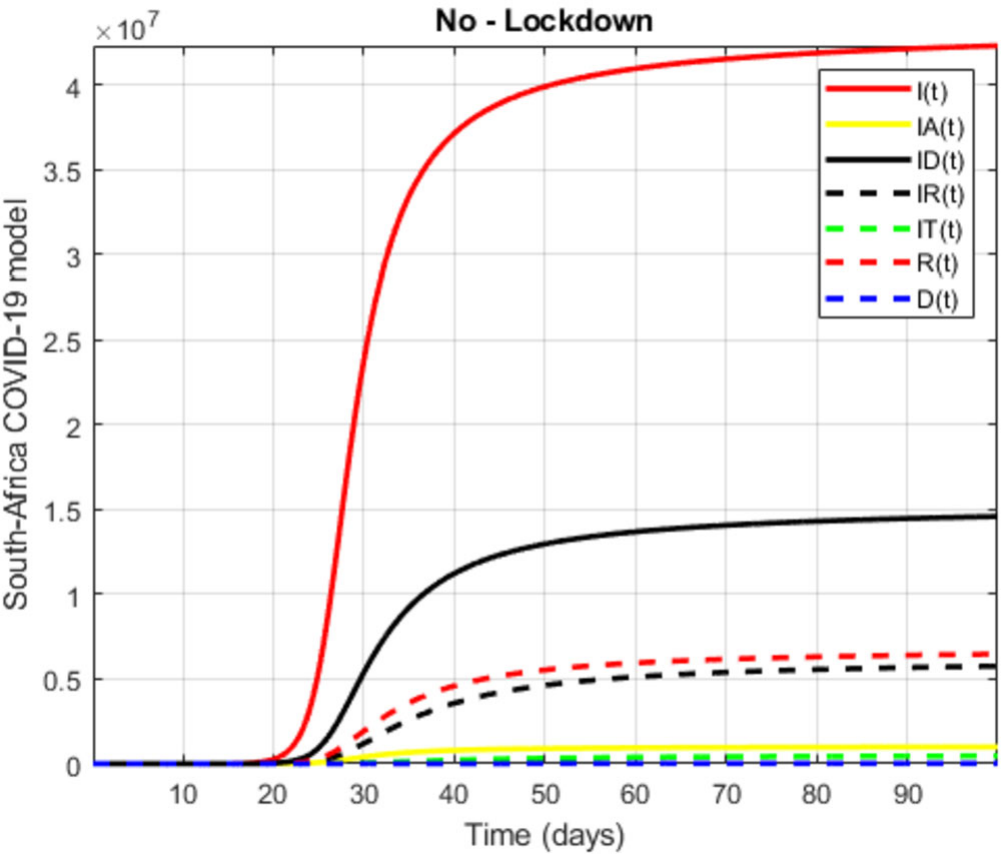
Numerical visualization for Covid-19 model in South Africa for $\alpha =0.75$

**Figure 38:**
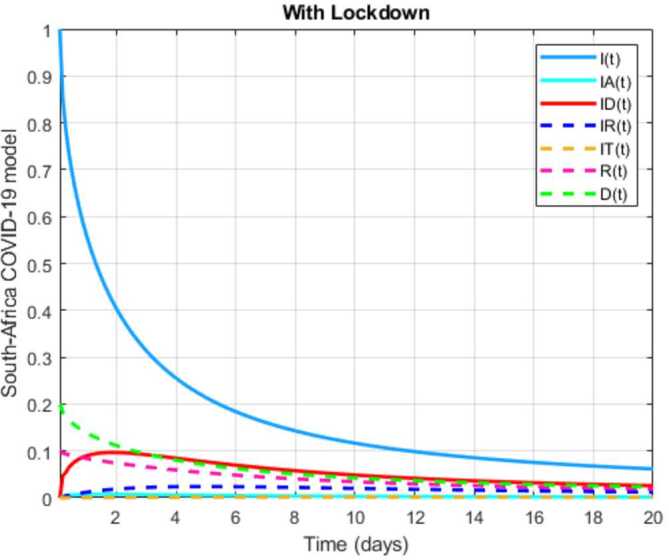
Numerical visualization for Covid-19 model in South Africa for $\alpha =0.75$

We present a numerical simulation for Covid-19 model in Figs. 37 and 38.

**Figure 39:**
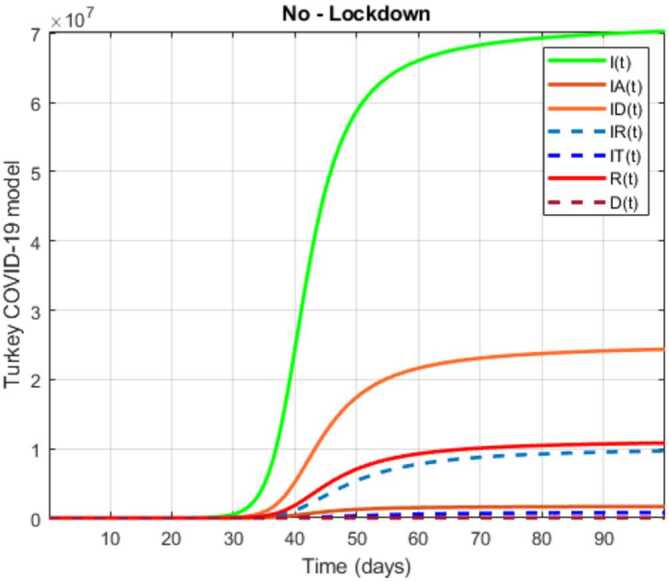
Numerical visualization for Covid-19 model in Turkey for $\alpha =0.8$

**Figure 40:**
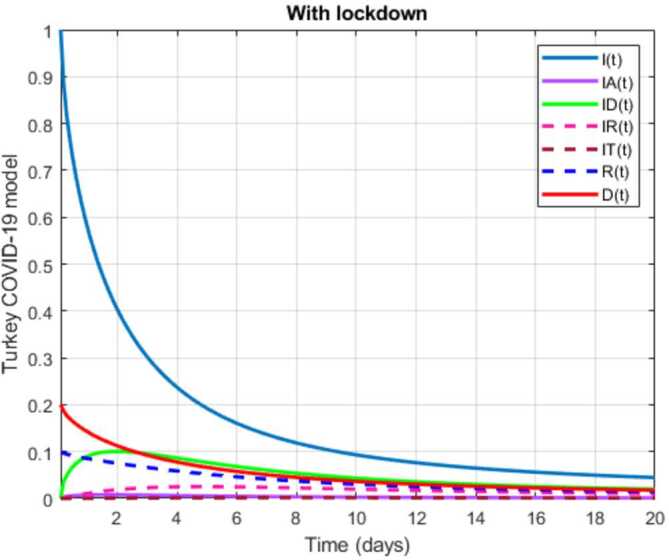
Numerical visualization for Covid-19 model in Turkey for $\alpha =0.8$

In Figs. 39 and 40, the initial conditions are chosen as

5.22 $$\begin{aligned} &S ( 0 ) = 81000000,\qquad I ( 0 ) =1,\qquad I_{A} ( 0 ) =1,\qquad I_{D} ( 0 ) =1,\qquad I_{R} ( 0 ) =1, \\ &I_{T} ( 0 ) = 1,\qquad R ( 0 ) =0,\qquad D ( 0 ) =0,\qquad V ( 0 ) =0. \end{aligned}$$

Also the parameters are

5.23 $$\begin{aligned} \begin{aligned} &\Lambda = 80000000,\qquad k=2,\qquad p=0.5,\qquad \eta =0.12,\qquad \chi =0.015,\\ & v=0.027,\qquad x=0.4, \qquad \theta =0.301, \qquad \gamma = 0.09,\qquad\beta =0.013,\\ & \gamma _{1}=0.4,\qquad \mu _{1}=0.3,\qquad \varepsilon =0.161,\qquad \xi =0.015,\qquad \sigma =0.015,\\ & \tau = 0.0199,\qquad \Phi =0.2,\qquad \lambda =0.0345,\qquad \varphi =0.0345,\qquad \delta _{1}=0.01. \end{aligned} \end{aligned}$$

We present a numerical simulation for Covid-19 model in Figs. 39 and 40.

Now, we replace the classical differential operator by the operator with power-law, exponential decay, and Mittag-Leffler kernels. We start with the exponential decay kernel:

5.24 $$\begin{aligned} &{}_{0}^{FFE}D_{t}^{\alpha,\beta }S = \Lambda - \bigl( \alpha ( x ) +\gamma _{1}+\mu _{1} \bigr) S, \\ &{}_{0}^{FFE}D_{t}^{\alpha,\beta }I= \alpha ( x ) S- ( \varepsilon +\xi +\lambda +\mu _{1} ) I, \\ &{}_{0}^{FFE}D_{t}^{\alpha,\beta }I_{A} = \xi I- ( \theta + \mu +\chi +\mu _{1} ) I_{A}, \\ &{}_{0}^{FFE}D_{t}^{\alpha,\beta }I_{D} = \varepsilon I- ( \eta +\varphi +\mu _{1} ) I_{D}, \\ &{}_{0}^{FFE}D_{t}^{\alpha,\beta }I_{R} = \eta I_{D}+\theta I_{A}- ( v+\xi +\mu _{1} ) I_{R}, \\ &{}_{0}^{FFE}D_{t}^{\alpha,\beta }I_{T} = \mu I_{A}+vI_{R}- ( \sigma +\tau +\mu _{1} ) I_{T}, \\ &{}_{0}^{FFE}D_{t}^{\alpha,\beta }R= \lambda I+\varphi I_{D}+ \chi I_{A}+\xi I_{R}+\sigma I_{T}- ( \Phi +\mu _{1} ) R, \\ &{}_{0}^{FFE}D_{t}^{\alpha,\beta }D= \tau I_{T}, \\ &{}_{0}^{FFE}D_{t}^{\alpha,\beta }V= \gamma _{1}S+\Phi R-\mu _{1}V. \end{aligned}$$

For simplicity, we write the above equations as follows:

5.25 $$\begin{aligned} &{}_{0}^{FFE}D_{t}^{\alpha,\beta }S = \widetilde{S} ( t,S,I,I_{A},I_{D},I_{R},I_{T},R,D,V ), \\ &{}_{0}^{FFE}D_{t}^{\alpha,\beta }I= \widetilde{I} ( t,S,I,I_{A},I_{D},I_{R},I_{T},R,D,V ), \\ &{}_{0}^{FFE}D_{t}^{\alpha,\beta }I_{A} = \widetilde{I_{A}} ( t,S,I,I_{A},I_{D},I_{R},I_{T},R,D,V ), \\ &{}_{0}^{FFE}D_{t}^{\alpha,\beta }I_{D} = \widetilde{I_{D}} ( t,S,I,I_{A},I_{D},I_{R},I_{T},R,D,V ), \\ &{}_{0}^{FFE}D_{t}^{\alpha,\beta }I_{R} = \widetilde{I_{R}} ( t,S,I,I_{A},I_{D},I_{R},I_{T},R,D,V ), \\ &{}_{0}^{FFE}D_{t}^{\alpha,\beta }I_{T} = \widetilde{I_{T}} ( t,S,I,I_{A},I_{D},I_{R},I_{T},R,D,V ), \\ &{}_{0}^{FFE}D_{t}^{\alpha,\beta }R= \widetilde{R} ( t,S,I,I_{A},I_{D},I_{R},I_{T},R,D,V ), \\ &{}_{0}^{FFE}D_{t}^{\alpha,\beta }D= \widetilde{D} ( t,S,I,I_{A},I_{D},I_{R},I_{T},R,D,V ), \\ &{}_{0}^{FFE}D_{t}^{\alpha,\beta }V= \widetilde{V} ( t,S,I,I_{A},I_{D},I_{R},I_{T},R,D,V ). \end{aligned}$$

After applying fractal-fractional integral with the exponential kernel, we have the following:

5.26 $$\begin{aligned} S ( t_{p+1} ) ={}& S ( t_{p} ) + \frac{1-\alpha }{M ( \alpha ) } \begin{bmatrix} t_{p}^{1-\beta }\widetilde{S} ( \tau,S^{p},I^{p},I_{A}^{ p},I_{D}^{p},I_{R}^{p},I_{T}^{p},R^{p},D^{p},V^{p} ) \\ -t_{p-1}^{1-\beta }\widetilde{S} ( \tau,S^{p-1},I^{p-1},I_{A}^{ p-1},I_{D}^{p-1},I_{R}^{p-1},I_{T}^{p-1},R^{p-1},D^{p-1},V^{p-1} )\end{bmatrix} \\ &{}+\frac{\alpha }{M ( \alpha ) } \int _{t_{p}}^{t_{p+1}} \widetilde{S} ( \tau,S,I,I_{A},I_{D},I_{R},I_{T},R,D,V ) \tau ^{1- \beta }\,d\tau, \\ I ( t_{p+1} ) ={}& I ( t_{p} ) + \frac{1-\alpha }{M ( \alpha ) } \begin{bmatrix} t_{p}^{1-\beta }\widetilde{I} ( \tau,S^{p},I^{p},I_{A}^{ p},I_{D}^{p},I_{R}^{p},I_{T}^{p},R^{p},D^{p},V^{p} ) \\ -t_{p-1}^{1-\beta }\widetilde{I} ( \tau,S^{p-1},I^{p-1},I_{A}^{ p-1},I_{D}^{p-1},I_{R}^{p-1},I_{T}^{p-1},R^{p-1},D^{p-1},V^{p-1} )\end{bmatrix} \\ &{}+\frac{\alpha }{M ( \alpha ) } \int _{t_{p}}^{t_{p+1}} \widetilde{I} ( \tau,S,I,I_{A},I_{D},I_{R},I_{T},R,D,V ) \tau ^{1- \beta }\,d\tau, \\ I_{A} ( t_{p+1} ) ={}& I_{A} ( t_{p} ) + \frac{1-\alpha }{M ( \alpha ) } \begin{bmatrix} t_{p}^{1-\beta }\widetilde{I_{A}} ( \tau,S^{p},I^{p},I_{A}^{p},I_{D}^{p},I_{R}^{p},I_{T}^{p},R^{p},D^{p},V^{p} ) \\ -t_{p-1}^{1-\beta }\widetilde{I_{A}} ( \tau,S^{p-1},I^{p-1},I_{A}^{ p-1},I_{D}^{p-1},I_{R}^{p-1},I_{T}^{p-1},R^{p-1},D^{p-1},V^{p-1} )\end{bmatrix} \\ &{}+\frac{\alpha }{M ( \alpha ) } \int _{t_{p}}^{t_{p+1}} \widetilde{I_{A}} ( \tau,S,I,I_{A},I_{D},I_{R},I_{T},R,D,V ) \tau ^{1-\beta }\,d\tau, \\ I_{D} ( t_{p+1} ) ={}& I_{D} ( t_{p} ) + \frac{1-\alpha }{M ( \alpha ) } \begin{bmatrix} t_{p}^{1-\beta }\widetilde{I_{D}} ( \tau,S^{p},I^{p},I_{A}^{ p},I_{D}^{p},I_{R}^{p},I_{T}^{p},R^{p},D^{p},V^{p} ) \\ -t_{p-1}^{1-\beta }\widetilde{I_{D}} ( \tau,S^{p-1},I^{p-1},I_{A}^{p-1},I_{D}^{p-1},I_{R}^{p-1},I_{T}^{p-1},R^{p-1},D^{p-1},V^{p-1} )\end{bmatrix} \\ &{}+\frac{\alpha }{M ( \alpha ) } \int _{t_{p}}^{t_{p+1}} \widetilde{I_{D}} ( \tau,S,I,I_{A},I_{D},I_{R},I_{T},R,D,V ) \tau ^{1- \beta }\,d\tau, \\ I_{R} ( t_{p+1} ) ={}& I_{R} ( t_{p} ) + \frac{1-\alpha }{M ( \alpha ) } \begin{bmatrix} t_{p}^{1-\beta }\widetilde{I_{R}} ( \tau,S^{p},I^{p},I_{A}^{ p},I_{D}^{p},I_{R}^{p},I_{T}^{p},R^{p},D^{p},V^{p} ) \\ -t_{p-1}^{1-\beta }\widetilde{I_{R}} ( \tau,S^{p-1},I^{p-1},I_{A}^{p-1},I_{D}^{p-1},I_{R}^{p-1},I_{T}^{p-1},R^{p-1},D^{p-1},V^{p-1} )\end{bmatrix} \\ &{}+\frac{\alpha }{M ( \alpha ) } \int _{t_{p}}^{t_{p+1}} \widetilde{I_{R}} ( \tau,S,I,I_{A},I_{D},I_{R},I_{T},R,D,V ) \tau ^{1- \beta }\,d\tau, \\ I_{T} ( t_{p+1} ) ={}& I_{T} ( t_{p} ) + \frac{1-\alpha }{M ( \alpha ) } \begin{bmatrix} t_{p}^{1-\beta }\widetilde{I_{T}} ( \tau,S^{p},I^{p},I_{A}^{ p},I_{D}^{p},I_{R}^{p},I_{T}^{p},R^{p},D^{p},V^{p} ) \\ -t_{p-1}^{1-\beta }\widetilde{I_{T}} ( \tau,S^{p-1},I^{p-1},I_{A}^{p-1},I_{D}^{p-1},I_{R}^{p-1},I_{T}^{p-1},R^{p-1},D^{p-1},V^{p-1} )\end{bmatrix} \\ &{}+\frac{\alpha }{M ( \alpha ) } \int _{t_{p}}^{t_{p+1}} \widetilde{I_{T}} ( \tau,S,I,I_{A},I_{D},I_{R},I_{T},R,D,V ) \tau ^{1- \beta }\,d\tau, \\ R ( t_{p+1} ) ={}& R ( t_{p} ) + \frac{1-\alpha }{M ( \alpha ) } \begin{bmatrix} t_{p}^{1-\beta }\widetilde{R} ( \tau,S^{p},I^{p},I_{A}^{ p},I_{D}^{p},I_{R}^{p},I_{T}^{p},R^{p},D^{p},V^{p} ) \\ -t_{p-1}^{1-\beta }\widetilde{R} ( \tau,S^{p-1},I^{p-1},I_{A}^{ p-1},I_{D}^{p-1},I_{R}^{p-1},I_{T}^{p-1},R^{p-1},D^{p-1},V^{p-1} )\end{bmatrix} \\ &{}+\frac{\alpha }{M ( \alpha ) } \int _{t_{p}}^{t_{p+1}} \widetilde{R} ( \tau,S,I,I_{A},I_{D},I_{R},I_{T},R,D,V ) \tau ^{1- \beta }\,d\tau, \\ D ( t_{p+1} ) ={}& D ( t_{p} ) + \frac{1-\alpha }{M ( \alpha ) } \begin{bmatrix} t_{p}^{1-\beta }\widetilde{D} ( \tau,S^{p},I^{p},I_{A}^{ p},I_{D}^{p},I_{R}^{p},I_{T}^{p},R^{p},D^{p},V^{p} ) \\ -t_{p-1}^{1-\beta }\widetilde{D} ( \tau,S^{p-1},I^{p-1},I_{A}^{ p-1},I_{D}^{p-1},I_{R}^{p-1},I_{T}^{p-1},R^{p-1},D^{p-1},V^{p-1} )\end{bmatrix} \\ &{}+\frac{\alpha }{M ( \alpha ) } \int _{t_{p}}^{t_{p+1}} \widetilde{D} ( \tau,S,I,I_{A},I_{D},I_{R},I_{T},R,D,V ) \tau ^{1- \beta }\,d\tau, \\ V ( t_{p+1} ) ={}& V ( t_{p} ) + \frac{1-\alpha }{M ( \alpha ) } \begin{bmatrix} t_{p}^{1-\beta }\widetilde{V} ( \tau,S^{p},I^{p},I_{A}^{ p},I_{D}^{p},I_{R}^{p},I_{T}^{p},R^{p},D^{p},V^{p} ) \\ -t_{p-1}^{1-\beta }\widetilde{V} ( \tau,S^{p-1},I^{p-1},I_{A}^{ p-1},I_{D}^{p-1},I_{R}^{p-1},I_{T}^{p-1},R^{p-1},D^{p-1},V^{p-1} )\end{bmatrix} \\ &{}+\frac{\alpha }{M ( \alpha ) } \int _{t_{p}}^{t_{p+1}} \widetilde{V} ( \tau,S,I,I_{A},I_{D},I_{R},I_{T},R,D,V ) \tau ^{1- \beta }\,d\tau. \end{aligned}$$

We can have the following scheme for this model:

5.27 $$\begin{array}{rl} S^{p+1}= & S^{p}\\ & +\frac{1-\alpha }{M(\alpha )}\left[\begin{array}{c} t_{p}^{1-\beta }\tilde{S}(t_{p},S^{p},I^{p},I_{A}^{p},I_{D}^{p},I_{R}^{p},I_{T}^{p},R^{p},D^{p},V^{p})\\ -t_{p-1}^{1-\beta }\tilde{S}(t_{p-1},S^{p-1},I^{p-1},I_{A}^{p-1},I_{D}^{p-1},I_{R}^{p-1},I_{T}^{p-1},R^{p-1},D^{p-1},V^{p-1}) \end{array}\right]\\ & +\frac{\alpha }{M(\alpha )}\left\{\begin{array}{c} \frac{23}{12}t_{p}^{1-\beta }\tilde{S}(t_{p},S^{p},I^{p},I_{A}^{p},I_{D}^{p},I_{R}^{p},I_{T}^{p},R^{p},D^{p},V^{p})\Delta t\\ -\frac{4}{3}t_{p-1}^{1-\beta }\tilde{S}(t_{p-1},S^{p-1},I^{p-1},I_{A}^{p-1},I_{D}^{p-1},I_{R}^{p-1},I_{T}^{p-1},R^{p-1},D^{p-1},V^{p-1})\Delta t\\ +\frac{5}{12}t_{p-2}^{1-\beta }\tilde{S}(t_{p-2},S^{p-2},I^{p-2},I_{A}^{p-2},I_{D}^{p-2},I_{R}^{p-2},I_{T}^{p-2},R^{p-2},D^{p-2},V^{p-2})\Delta t \end{array}\right\},\\ I^{p+1}= & I^{p}+\frac{1-\alpha }{M(\alpha )}\left[\begin{array}{c} t_{p}^{1-\beta }\tilde{I}(t_{p},S^{p},I^{p},I_{A}^{p},I_{D}^{p},I_{R}^{p},I_{T}^{p},R^{p},D^{p},V^{p})\\ -t_{p-1}^{1-\beta }\tilde{I}(t_{p-1},S^{p-1},I^{p-1},I_{A}^{p-1},I_{D}^{p-1},I_{R}^{p-1},I_{T}^{p-1},R^{p-1},D^{p-1},V^{p-1}) \end{array}\right]\\ & +\frac{\alpha }{M(\alpha )}\left\{\begin{array}{c} \frac{23}{12}t_{p}^{1-\beta }\tilde{I}(t_{p},S^{p},I^{p},I_{A}^{p},I_{D}^{p},I_{R}^{p},I_{T}^{p},R^{p},D^{p},V^{p})\Delta t\\ -\frac{4}{3}t_{p-1}^{1-\beta }\tilde{I}(t_{p-1},S^{p-1},I^{p-1},I_{A}^{p-1},I_{D}^{p-1},I_{R}^{p-1},I_{T}^{p-1},R^{p-1},D^{p-1},V^{p-1})\Delta t\\ +\frac{5}{12}t_{p-2}^{1-\beta }\tilde{I}(t_{p-2},S^{p-2},I^{p-2},I_{A}^{p-2},I_{D}^{p-2},I_{R}^{p-2},I_{T}^{p-2},R^{p-2},D^{p-2},V^{p-2})\Delta t \end{array}\right\},\\ I_{A}^{p+1}= & I_{A}^{p}+\frac{1-\alpha }{M(\alpha )}\left[\begin{array}{c} t_{p}^{1-\beta }\tilde{I_{A}}(t_{p},S^{p},I^{p},I_{A}^{p},I_{D}^{p},I_{R}^{p},I_{T}^{p},R^{p},D^{p},V^{p})\\ -t_{p-1}^{1-\beta }\tilde{I_{A}}(t_{p-1},S^{p-1},I^{p-1},I_{A}^{p-1},I_{D}^{p-1},I_{R}^{p-1},I_{T}^{p-1},R^{p-1},D^{p-1},V^{p-1}) \end{array}\right]\\ & +\frac{\alpha }{M(\alpha )}\left\{\begin{array}{c} \frac{23}{12}t_{p}^{1-\beta }\tilde{I_{A}}(t_{p},S^{p},I^{p},I_{A}^{p},I_{D}^{p},I_{R}^{p},I_{T}^{p},R^{p},D^{p},V^{p})\Delta t\\ -\frac{4}{3}t_{p-1}^{1-\beta }\tilde{I_{A}}(t_{p-1},S^{p-1},I^{p-1},I_{A}^{p-1},I_{D}^{p-1},I_{R}^{p-1},I_{T}^{p-1},R^{p-1},D^{p-1},V^{p-1})\Delta t\\ +\frac{5}{12}t_{p-2}^{1-\beta }\tilde{I_{A}}(t_{p-2},S^{p-2},I^{p-2},I_{A}^{p-2},I_{D}^{p-2},I_{R}^{p-2},I_{T}^{p-2},R^{p-2},D^{p-2},V^{p-2})\Delta t \end{array}\right\},\\ I_{D}^{p+1}= & I_{D}^{p}+\frac{1-\alpha }{M(\alpha )}\left[\begin{array}{c} t_{p}^{1-\beta }\tilde{I_{D}}(t_{p},S^{p},I^{p},I_{A}^{p},I_{D}^{p},I_{R}^{p},I_{T}^{p},R^{p},D^{p},V^{p})\\ -t_{p-1}^{1-\beta }\tilde{I_{D}}(t_{p-1},S^{p-1},I^{p-1},I_{A}^{p-1},I_{D}^{p-1},I_{R}^{p-1},I_{T}^{p-1},R^{p-1},D^{p-1},V^{p-1}) \end{array}\right]\\ & +\frac{\alpha }{M(\alpha )}\left\{\begin{array}{c} \frac{23}{12}t_{p}^{1-\beta }\tilde{I_{D}}(t_{p},S^{p},I^{p},I_{A}^{p},I_{D}^{p},I_{R}^{p},I_{T}^{p},R^{p},D^{p},V^{p})\Delta t\\ -\frac{4}{3}t_{p-1}^{1-\beta }\tilde{I_{D}}(t_{p-1},S^{p-1},I^{p-1},I_{A}^{p-1},I_{D}^{p-1},I_{R}^{p-1},I_{T}^{p-1},R^{p-1},D^{p-1},V^{p-1})\Delta t\\ +\frac{5}{12}t_{p-2}^{1-\beta }\tilde{I_{D}}(t_{p-2},S^{p-2},I^{p-2},I_{A}^{p-2},I_{D}^{p-2},I_{R}^{p-2},I_{T}^{p-2},R^{p-2},D^{p-2},V^{p-2})\Delta t \end{array}\right\},\\ I_{R}^{p+1}= & I_{R}^{p}+\frac{1-\alpha }{M(\alpha )}\left[\begin{array}{c} t_{p}^{1-\beta }\tilde{I_{R}}(t_{p},S^{p},I^{p},I_{A}^{p},I_{D}^{p},I_{R}^{p},I_{T}^{p},R^{p},D^{p},V^{p})\\ -t_{p-1}^{1-\beta }\tilde{I_{R}}(t_{p-1},S^{p-1},I^{p-1},I_{A}^{p-1},I_{D}^{p-1},I_{R}^{p-1},I_{T}^{p-1},R^{p-1},D^{p-1},V^{p-1}) \end{array}\right]\\ & +\frac{\alpha }{M(\alpha )}\left\{\begin{array}{c} \frac{23}{12}t_{p}^{1-\beta }\tilde{I_{R}}(t_{p},S^{p},I^{p},I_{A}^{p},I_{D}^{p},I_{R}^{p},I_{T}^{p},R^{p},D^{p},V^{p})\Delta t\\ -\frac{4}{3}t_{p-1}^{1-\beta }\tilde{I_{R}}(t_{p-1},S^{p-1},I^{p-1},I_{A}^{p-1},I_{D}^{p-1},I_{R}^{p-1},I_{T}^{p-1},R^{p-1},D^{p-1},V^{p-1})\Delta t\\ +\frac{5}{12}t_{p-2}^{1-\beta }\tilde{I_{R}}(t_{p-2},S^{p-2},I^{p-2},I_{A}^{p-2},I_{D}^{p-2},I_{R}^{p-2},I_{T}^{p-2},R^{p-2},D^{p-2},V^{p-2})\Delta t \end{array}\right\}\\ I_{T}^{p+1}= & I_{T}^{p}+\frac{1-\alpha }{M(\alpha )}\left[\begin{array}{c} t_{p}^{1-\beta }\tilde{I_{T}}(t_{p},S^{p},I^{p},I_{A}^{p},I_{D}^{p},I_{R}^{p},I_{T}^{p},R^{p},D^{p},V^{p})\\ -t_{p-1}^{1-\beta }\tilde{I_{T}}(t_{p-1},S^{p-1},I^{p-1},I_{A}^{p-1},I_{D}^{p-1},I_{R}^{p-1},I_{T}^{p-1},R^{p-1},D^{p-1},V^{p-1}) \end{array}\right]\\ & +\frac{\alpha }{M(\alpha )}\left\{\begin{array}{c} \frac{23}{12}t_{p}^{1-\beta }\tilde{I_{T}}(t_{p},S^{p},I^{p},I_{A}^{p},I_{D}^{p},I_{R}^{p},I_{T}^{p},R^{p},D^{p},V^{p})\Delta t\\ -\frac{4}{3}t_{p-1}^{1-\beta }\tilde{I_{T}}(t_{p-1},S^{p-1},I^{p-1},I_{A}^{p-1},I_{D}^{p-1},I_{R}^{p-1},I_{T}^{p-1},R^{p-1},D^{p-1},V^{p-1})\Delta t\\ +\frac{5}{12}t_{p-2}^{1-\beta }\tilde{I_{T}}(t_{p-2},S^{p-2},I^{p-2},I_{A}^{p-2},I_{D}^{p-2},I_{R}^{p-2},I_{T}^{p-2},R^{p-2},D^{p-2},V^{p-2})\Delta t \end{array}\right\},\\ R^{p+1}= & R^{p}+\frac{1-\alpha }{M(\alpha )}\left[\begin{array}{c} t_{p}^{1-\beta }\tilde{R}(t_{p},S^{p},I^{p},I_{A}^{p},I_{D}^{p},I_{R}^{p},I_{T}^{p},R^{p},D^{p},V^{p})\\ -t_{p-1}^{1-\beta }\tilde{R}(t_{p-1},S^{p-1},I^{p-1},I_{A}^{p-1},I_{D}^{p-1},I_{R}^{p-1},I_{T}^{p-1},R^{p-1},D^{p-1},V^{p-1}) \end{array}\right]\\ & +\frac{\alpha }{M(\alpha )}\left\{\begin{array}{c} \frac{23}{12}t_{p}^{1-\beta }\tilde{R}(t_{p},S^{p},I^{p},I_{A}^{p},I_{D}^{p},I_{R}^{p},I_{T}^{p},R^{p},D^{p},V^{p})\Delta t\\ -\frac{4}{3}t_{p-1}^{1-\beta }\tilde{R}(t_{p-1},S^{p-1},I^{p-1},I_{A}^{p-1},I_{D}^{p-1},I_{R}^{p-1},I_{T}^{p-1},R^{p-1},D^{p-1},V^{p-1})\Delta t\\ +\frac{5}{12}t_{p-2}^{1-\beta }\tilde{R}(t_{p-2},S^{p-2},I^{p-2},I_{A}^{p-2},I_{D}^{p-2},I_{R}^{p-2},I_{T}^{p-2},R^{p-2},D^{p-2},V^{p-2})\Delta t \end{array}\right\},\\ D^{p+1}= & D^{p}+\frac{1-\alpha }{M(\alpha )}\left[\begin{array}{c} t_{p}^{1-\beta }\tilde{D}(t_{p},S^{p},I^{p},I_{A}^{p},I_{D}^{p},I_{R}^{p},I_{T}^{p},R^{p},D^{p},V^{p})\\ -t_{p-1}^{1-\beta }\tilde{D}(t_{p-1},S^{p-1},I^{p-1},I_{A}^{p-1},I_{D}^{p-1},I_{R}^{p-1},I_{T}^{p-1},R^{p-1},D^{p-1},V^{p-1}) \end{array}\right]\\ & +\frac{\alpha }{M(\alpha )}\left\{\begin{array}{c} \frac{23}{12}t_{p}^{1-\beta }\tilde{D}(t_{p},S^{p},I^{p},I_{A}^{p},I_{D}^{p},I_{R}^{p},I_{T}^{p},R^{p},D^{p},V^{p})\Delta t\\ -\frac{4}{3}t_{p-1}^{1-\beta }\tilde{D}(t_{p-1},S^{p-1},I^{p-1},I_{A}^{p-1},I_{D}^{p-1},I_{R}^{p-1},I_{T}^{p-1},R^{p-1},D^{p-1},V^{p-1})\Delta t\\ +\frac{5}{12}t_{p-2}^{1-\beta }\tilde{D}(t_{p-2},S^{p-2},I^{p-2},I_{A}^{p-2},I_{D}^{p-2},I_{R}^{p-2},I_{T}^{p-2},R^{p-2},D^{p-2},V^{p-2})\Delta t \end{array}\right\},\\ V^{p+1}= & V^{p}+\frac{1-\alpha }{M(\alpha )}\left[\begin{array}{c} t_{p}^{1-\beta }\tilde{V}(t_{p},S^{p},I^{p},I_{A}^{p},I_{D}^{p},I_{R}^{p},I_{T}^{p},R^{p},D^{p},V^{p})\\ -t_{p-1}^{1-\beta }\tilde{V}(t_{p-1},S^{p-1},I^{p-1},I_{A}^{p-1},I_{D}^{p-1},I_{R}^{p-1},I_{T}^{p-1},R^{p-1},D^{p-1},V^{p-1}) \end{array}\right]\\ & +\frac{\alpha }{M(\alpha )}\left\{\begin{array}{c} \frac{23}{12}t_{p}^{1-\beta }\tilde{V}(t_{p},S^{p},I^{p},I_{A}^{p},I_{D}^{p},I_{R}^{p},I_{T}^{p},R^{p},D^{p},V^{p})\Delta t\\ -\frac{4}{3}t_{p-1}^{1-\beta }\tilde{V}(t_{p-1},S^{p-1},I^{p-1},I_{A}^{p-1},I_{D}^{p-1},I_{R}^{p-1},I_{T}^{p-1},R^{p-1},D^{p-1},V^{p-1})\Delta t\\ +\frac{5}{12}t_{p-2}^{1-\beta }\tilde{V}(t_{p-2},S^{p-2},I^{p-2},I_{A}^{p-2},I_{D}^{p-2},I_{R}^{p-2},I_{T}^{p-2},R^{p-2},D^{p-2},V^{p-2})\Delta t \end{array}\right\}. \end{array}$$

For the Mittag-Leffler kernel, we have the following:

5.28 $$\begin{aligned} S^{p+1} ={}& S^{p}+\frac{1-\alpha }{AB ( \alpha ) }t_{p}^{1- \beta }\widetilde{S} \bigl( t_{p},S^{p},I^{p},I_{A}^{p},I_{D}^{ p},I_{R}^{p},I_{T}^{p},R^{p},D^{p},V^{p} \bigr)\\ &{}\times\frac{\alpha }{AB ( \alpha ) \Gamma ( \alpha ) }\sum_{r=2}^{p} \int _{t_{r}}^{t_{r+1}}\widetilde{S} ( \tau,S,I,I_{A},I_{D},I_{R},I_{T},R,D,V ) ( t_{p+1}-\tau ) ^{\alpha -1}\tau ^{1-\beta }\,d \tau, \\ I^{p+1} ={}& I^{p}+\frac{1-\alpha }{AB ( \alpha ) }t_{p}^{1- \beta }\widetilde{I} \bigl( t_{p},S^{p},I^{p},I_{A}^{p},I_{D}^{ p},I_{R}^{p},I_{T}^{p},R^{p},D^{p},V^{p} \bigr) \\ &{}\times\frac{\alpha }{AB ( \alpha ) \Gamma ( \alpha ) }\sum_{r=2}^{p} \int _{t_{r}}^{t_{r+1}}\widetilde{I} ( \tau,S,I,I_{A},I_{D},I_{R},I_{T},R,D,V ) ( t_{p+1}-\tau ) ^{\alpha -1}\tau ^{1-\beta }\,d \tau, \\ I_{A}^{p+1} ={}& I_{A}^{p}+ \frac{1-\alpha }{AB ( \alpha ) }t_{p}^{1-\beta } \widetilde{I_{A}} \bigl( t_{p},S^{p},I^{p},I_{A}^{ p},I_{D}^{p},I_{R}^{p},I_{T}^{p},R^{p},D^{p},V^{p} \bigr) \\ &{}\times\frac{\alpha }{AB ( \alpha ) \Gamma ( \alpha ) }\sum_{r=2}^{p} \int _{t_{r}}^{t_{r+1}}\widetilde{I_{A}} ( \tau,S,I,I_{A},I_{D},I_{R},I_{T},R,D,V ) ( t_{p+1}-\tau ) ^{\alpha -1}\tau ^{1-\beta }\,d\tau, \\ I_{D}^{p+1} ={}& I_{D}^{p}+ \frac{1-\alpha }{AB ( \alpha ) }t_{p}^{1-\beta } \widetilde{I_{D}} \bigl( t_{p},S^{p},I^{p},I_{A}^{ p},I_{D}^{p},I_{R}^{p},I_{T}^{p},R^{p},D^{p},V^{p} \bigr) \\ &{}\times\frac{\alpha }{AB ( \alpha ) \Gamma ( \alpha ) }\sum_{r=2}^{p} \int _{t_{r}}^{t_{r+1}}\widetilde{I_{D}} ( \tau,S,I,I_{A},I_{D},I_{R},I_{T},R,D,V ) ( t_{p+1}-\tau ) ^{\alpha -1}\tau ^{1-\beta }\,d \tau, \\ I_{R}^{p+1} ={}& I_{R}^{p}+ \frac{1-\alpha }{AB ( \alpha ) }t_{p}^{1-\beta } \widetilde{I_{R}} \bigl( t_{p},S^{p},I^{p},I_{A}^{ p},I_{D}^{p},I_{R}^{p},I_{T}^{p},R^{p},D^{p},V^{p} \bigr) \\ &{}\times\frac{\alpha }{AB ( \alpha ) \Gamma ( \alpha ) }\sum_{r=2}^{p} \int _{t_{r}}^{t_{r+1}}\widetilde{I_{R}} ( \tau,S,I,I_{A},I_{D},I_{R},I_{T},R,D,V ) ( t_{p+1}-\tau ) ^{\alpha -1}\tau ^{1-\beta }\,d\tau, \\ I_{T}^{p+1} ={}& I_{T}^{p}+ \frac{1-\alpha }{AB ( \alpha ) }t_{p}^{1-\beta } \widetilde{I_{T}} \bigl( t_{p},S^{p},I^{p},I_{A}^{ p},I_{D}^{p},I_{R}^{p},I_{T}^{p},R^{p},D^{p},V^{p} \bigr) \\ &{}\times\frac{\alpha }{AB ( \alpha ) \Gamma ( \alpha ) }\sum_{r=2}^{p} \int _{t_{r}}^{t_{r+1}}\widetilde{I_{T}} ( \tau,S,I,I_{A},I_{D},I_{R},I_{T},R,D,V ) ( t_{p+1}-\tau ) ^{\alpha -1}\tau ^{1-\beta }\,d \tau, \\ R^{p+1} = {}&R^{p}+\frac{1-\alpha }{AB ( \alpha ) }t_{p}^{1- \beta }\widetilde{R} \bigl( t_{p},S^{p},I^{p},I_{A}^{p},I_{D}^{ p},I_{R}^{p},I_{T}^{p},R^{p},D^{p},V^{p} \bigr) \\ &{}\times\frac{\alpha }{AB ( \alpha ) \Gamma ( \alpha ) }\sum_{r=2}^{p} \int _{t_{r}}^{t_{r+1}}\widetilde{R} ( \tau,S,I,I_{A},I_{D},I_{R},I_{T},R,D,V ) ( t_{p+1}-\tau ) ^{\alpha -1}\tau ^{1-\beta }\,d\tau, \\ D^{p+1} ={}& D^{p}+\frac{1-\alpha }{AB ( \alpha ) }t_{p}^{1- \beta }\widetilde{D} \bigl( t_{p},S^{p},I^{p},I_{A}^{p},I_{D}^{ p},I_{R}^{p},I_{T}^{p},R^{p},D^{p},V^{p} \bigr) \\ &{}\times\frac{\alpha }{AB ( \alpha ) \Gamma ( \alpha ) }\sum_{r=2}^{p} \int _{t_{r}}^{t_{r+1}}\widetilde{D} ( \tau,S,I,I_{A},I_{D},I_{R},I_{T},R,D,V ) ( t_{p+1}-\tau ) ^{\alpha -1}\tau ^{1-\beta }\,d \tau, \\ V^{p+1} ={}& V^{p}+\frac{1-\alpha }{AB ( \alpha ) }t_{p}^{1- \beta }\widetilde{V} \bigl( t_{p},S^{p},I^{p},I_{A}^{p},I_{D}^{ p},I_{R}^{p},I_{T}^{p},R^{p},D^{p},V^{p} \bigr) \\ &{}\times\frac{\alpha }{AB ( \alpha ) \Gamma ( \alpha ) }\sum_{r=2}^{p} \int _{t_{r}}^{t_{r+1}}\widetilde{V} ( \tau,S,I,I_{A},I_{D},I_{R},I_{T},R,D,V ) ( t_{p+1}-\tau ) ^{\alpha -1}\tau ^{1-\beta }\,d \tau. \end{aligned}$$

We can get the following numerical scheme:

5.29 $$\begin{aligned} S^{p+1} ={}& \frac{1-\alpha }{AB ( \alpha ) }t_{p}^{1- \beta }\widetilde{S} \bigl( t_{p},S^{p},I^{p},I_{A}^{p},I_{D}^{ p},I_{R}^{p},I_{T}^{p},R^{p},D^{p},V^{p} \bigr) \\ &{}+ \frac{\alpha ( \Delta t ) ^{\alpha }}{AB ( \alpha ) \Gamma ( \alpha +1 ) }\sum_{r=2}^{p}t_{r-2}^{1-\beta } \widetilde{S} \bigl( t_{r-2},S^{r-2},I^{r-2},I_{A}^{r-2},I_{D}^{r-2},I_{R}^{r-2},I_{T}^{r-2},R^{r-2},D^{r-2},V^{r-2} \bigr) \\ &{}\times \bigl[ ( p-r+1 ) ^{\alpha }- ( p-r ) ^{ \alpha } \bigr] \\ &{}+ \frac{\alpha ( \Delta t ) ^{\alpha }}{AB ( \alpha ) \Gamma ( \alpha +2 ) } \\ &{}\times \sum_{r=2}^{p} \begin{bmatrix} t_{r-1}^{1-\beta }\widetilde{S} ( t_{r-1},S^{r-1},I^{r-1},I_{A}^{ r-1},I_{D}^{r-1},I_{R}^{r-1},I_{T}^{r-1},R^{r-1},D^{r-1},V^{r-1} ) \\ -t_{r-2}^{1-\beta }\widetilde{S} ( t_{r-2},S^{r-2},I^{r-2},I_{A}^{ r-2},I_{D}^{r-2},I_{R}^{r-2},I_{T}^{r-2},R^{r-2},D^{r-2},V^{r-2} )\end{bmatrix} \\ &{}\times \begin{bmatrix} ( p-r+1 ) ^{\alpha } ( p-r+3+2\alpha ) \\ - ( p-r ) ^{\alpha } ( p-r+3+3\alpha )\end{bmatrix} \\ &{}+ \frac{\alpha ( \Delta t ) ^{\alpha }}{2AB ( \alpha ) \Gamma ( \alpha +3 ) } \\ &{}\times \sum_{r=2}^{p} \begin{bmatrix} t_{r}^{1-\beta }\widetilde{S} ( t_{r},S^{r},I^{r},I_{A}^{ r},I_{D}^{r},I_{R}^{r},I_{T}^{r},R^{r},D^{r},V^{r} ) \\ -2t_{r-1}^{1-\beta }\widetilde{S} ( t_{r-1},S^{r-1},I^{r-1},I_{A}^{ r-1},I_{D}^{r-1},I_{R}^{r-1},I_{T}^{r-1},R^{r-1},D^{r-1},V^{r-1} ) \\ +t_{r-2}^{1-\beta }\widetilde{S} ( t_{r-2},S^{r-2},I^{r-2},I_{A}^{ r-2},I_{D}^{r-2},I_{R}^{r-2},I_{T}^{r-2},R^{r-2},D^{r-2},V^{r-2} )\end{bmatrix} \\ &{}\times \begin{bmatrix} ( p-r+1 ) ^{\alpha } \begin{bmatrix} 2 ( p-r ) ^{2}+ ( 3\alpha +10 ) ( p-r ) \\ +2\alpha ^{2}+9\alpha +12\end{bmatrix} \\ - ( p-r ) ^{\alpha } \begin{bmatrix} 2 ( p-r ) ^{2}+ ( 5\alpha +10 ) ( p-r ) \\ +6\alpha ^{2}+18\alpha +12\end{bmatrix}\end{bmatrix}, \\ I^{p+1} ={}& \frac{1-\alpha }{AB ( \alpha ) }t_{p}^{1- \beta }\widetilde{I} \bigl( t_{p},S^{p},I^{p},I_{A}^{p},I_{D}^{ p},I_{R}^{p},I_{T}^{p},R^{p},D^{p},V^{p} \bigr) \\ &{}+ \frac{\alpha ( \Delta t ) ^{\alpha }}{AB ( \alpha ) \Gamma ( \alpha +1 ) }\sum_{r=2}^{p}t_{r-2}^{1-\beta } \widetilde{I} \bigl( t_{r-2},S^{r-2},I^{r-2},I_{A}^{r-2},I_{D}^{r-2},I_{R}^{r-2},I_{T}^{r-2},R^{r-2},D^{r-2},V^{r-2} \bigr) \\ &{}\times \bigl[ ( p-r+1 ) ^{\alpha }- ( p-r ) ^{ \alpha } \bigr] \\ &{}+ \frac{\alpha ( \Delta t ) ^{\alpha }}{AB ( \alpha ) \Gamma ( \alpha +2 ) } \\ &{}\times \sum_{r=2}^{p} \begin{bmatrix} t_{r-1}^{1-\beta }\widetilde{I} ( t_{r-1},S^{r-1},I^{r-1},I_{A}^{ r-1},I_{D}^{r-1},I_{R}^{r-1},I_{T}^{r-1},R^{r-1},D^{r-1},V^{r-1} ) \\ -t_{r-2}^{1-\beta }\widetilde{I} ( t_{r-2},S^{r-2},I^{r-2},I_{A}^{ r-2},I_{D}^{r-2},I_{R}^{r-2},I_{T}^{r-2},R^{r-2},D^{r-2},V^{r-2} )\end{bmatrix} \\ &{}\times \begin{bmatrix} ( p-r+1 ) ^{\alpha } ( p-r+3+2\alpha ) \\ - ( p-r ) ^{\alpha } ( p-r+3+3\alpha )\end{bmatrix} \\ &{}+ \frac{\alpha ( \Delta t ) ^{\alpha }}{2AB ( \alpha ) \Gamma ( \alpha +3 ) } \end{aligned}$$

$$\begin{aligned} &{}\times \sum_{r=2}^{p} \begin{bmatrix} t_{r}^{1-\beta }\widetilde{I} ( t_{r},S^{r},I^{r},I_{A}^{ r},I_{D}^{r},I_{R}^{r},I_{T}^{r},R^{r},D^{r},V^{r} ) \\ -2t_{r-1}^{1-\beta }\widetilde{I} ( t_{r-1},S^{r-1},I^{r-1},I_{A}^{ r-1},I_{D}^{r-1},I_{R}^{r-1},I_{T}^{r-1},R^{r-1},D^{r-1},V^{r-1} ) \\ +t_{r-2}^{1-\beta }\widetilde{I} ( t_{r-2},S^{r-2},I^{r-2},I_{A}^{ r-2},I_{D}^{r-2},I_{R}^{r-2},I_{T}^{r-2},R^{r-2},D^{r-2},V^{r-2} )\end{bmatrix} \\ &{}\times \begin{bmatrix} ( p-r+1 ) ^{\alpha } \begin{bmatrix} 2 ( p-r ) ^{2}+ ( 3\alpha +10 ) ( p-r ) \\ +2\alpha ^{2}+9\alpha +12\end{bmatrix} \\ - ( p-r ) ^{\alpha } \begin{bmatrix} 2 ( p-r ) ^{2}+ ( 5\alpha +10 ) ( p-r ) \\ +6\alpha ^{2}+18\alpha +12\end{bmatrix}\end{bmatrix}, \\ I_{A}^{p+1} ={}& \frac{1-\alpha }{AB ( \alpha ) }t_{p}^{1- \beta }\widetilde{I_{A}} \bigl( t_{p},S^{p},I^{p},I_{A}^{p},I_{D}^{ p},I_{R}^{p},I_{T}^{p},R^{p},D^{p},V^{p} \bigr) \\ &{}+ \frac{\alpha ( \Delta t ) ^{\alpha }}{AB ( \alpha ) \Gamma ( \alpha +1 ) }\sum_{r=2}^{p}t_{r-2}^{1-\beta } \widetilde{I_{A}} \bigl( t_{r-2},S^{r-2},I^{r-2},I_{A}^{r-2},I_{D}^{ r-2},I_{R}^{r-2},I_{T}^{r-2},R^{r-2},D^{r-2},V^{r-2} \bigr) \\ &{}\times \bigl[ ( p-r+1 ) ^{\alpha }- ( p-r ) ^{ \alpha } \bigr] \\ &{}+ \frac{\alpha ( \Delta t ) ^{\alpha }}{AB ( \alpha ) \Gamma ( \alpha +2 ) } \\ &{}\times \sum_{r=2}^{p} \begin{bmatrix} t_{r-1}^{1-\beta }\widetilde{I_{A}} ( t_{r-1},S^{r-1},I^{r-1},I_{A}^{r-1},I_{D}^{r-1},I_{R}^{r-1},I_{T}^{r-1},R^{r-1},D^{r-1},V^{r-1} ) \\ -t_{r-2}^{1-\beta }\widetilde{I_{A}} ( t_{r-2},S^{r-2},I^{r-2},I_{A}^{r-2},I_{D}^{r-2},I_{R}^{r-2},I_{T}^{r-2},R^{r-2},D^{r-2},V^{r-2} )\end{bmatrix} \\ &{}\times \begin{bmatrix} ( p-r+1 ) ^{\alpha } ( p-r+3+2\alpha ) \\ - ( p-r ) ^{\alpha } ( p-r+3+3\alpha )\end{bmatrix} \\ &{}+ \frac{\alpha ( \Delta t ) ^{\alpha }}{2AB ( \alpha ) \Gamma ( \alpha +3 ) } \\ &{}\times \sum_{r=2}^{p} \begin{bmatrix} t_{r}^{1-\beta }\widetilde{I_{A}} ( t_{r},S^{r},I^{r},I_{A}^{ r},I_{D}^{r},I_{R}^{r},I_{T}^{r},R^{r},D^{r},V^{r} ) \\ -2t_{r-1}^{1-\beta }\widetilde{I_{A}} ( t_{r-1},S^{r-1},I^{r-1},I_{A}^{r-1},I_{D}^{r-1},I_{R}^{r-1},I_{T}^{r-1},R^{r-1},D^{r-1},V^{r-1} ) \\ +t_{r-2}^{1-\beta }\widetilde{I_{A}} ( t_{r-2},S^{r-2},I^{r-2},I_{A}^{r-2},I_{D}^{r-2},I_{R}^{r-2},I_{T}^{r-2},R^{r-2},D^{r-2},V^{r-2} )\end{bmatrix} \\ &{}\times \begin{bmatrix} ( p-r+1 ) ^{\alpha } \begin{bmatrix} 2 ( p-r ) ^{2}+ ( 3\alpha +10 ) ( p-r ) \\ +2\alpha ^{2}+9\alpha +12\end{bmatrix} \\ - ( p-r ) ^{\alpha } \begin{bmatrix} 2 ( p-r ) ^{2}+ ( 5\alpha +10 ) ( p-r ) \\ +6\alpha ^{2}+18\alpha +12\end{bmatrix}\end{bmatrix}, \\ I_{D}^{p+1} ={}& \frac{1-\alpha }{AB ( \alpha ) }t_{p}^{1- \beta }\widetilde{I_{D}} \bigl( t_{p},S^{p},I^{p},I_{A}^{p},I_{D}^{ p},I_{R}^{p},I_{T}^{p},R^{p},D^{p},V^{p} \bigr) \\ &{}+ \frac{\alpha ( \Delta t ) ^{\alpha }}{AB ( \alpha ) \Gamma ( \alpha +1 ) }\sum_{r=2}^{p}t_{r-2}^{1-\beta } \widetilde{I_{D}} \bigl( t_{r-2},S^{r-2},I^{r-2},I_{A}^{r-2},I_{D}^{ r-2},I_{R}^{r-2},I_{T}^{r-2},R^{r-2},D^{r-2},V^{r-2} \bigr) \\ &{}\times \bigl[ ( p-r+1 ) ^{\alpha }- ( p-r ) ^{ \alpha } \bigr] \\ &{}+ \frac{\alpha ( \Delta t ) ^{\alpha }}{AB ( \alpha ) \Gamma ( \alpha +2 ) } \\ &{}\times \sum_{r=2}^{p} \begin{bmatrix} t_{r-1}^{1-\beta }\widetilde{I_{D}} ( t_{r-1},S^{r-1},I^{r-1},I_{A}^{r-1},I_{D}^{r-1},I_{R}^{r-1},I_{T}^{r-1},R^{r-1},D^{r-1},V^{r-1} ) \\ -t_{r-2}^{1-\beta }\widetilde{I_{D}} ( t_{r-2},S^{r-2},I^{r-2},I_{A}^{r-2},I_{D}^{r-2},I_{R}^{r-2},I_{T}^{r-2},R^{r-2},D^{r-2},V^{r-2} )\end{bmatrix} \\ &{}\times \begin{bmatrix} ( p-r+1 ) ^{\alpha } ( p-r+3+2\alpha ) \\ - ( p-r ) ^{\alpha } ( p-r+3+3\alpha )\end{bmatrix} \end{aligned}$$

$$\begin{aligned} &{}+ \frac{\alpha ( \Delta t ) ^{\alpha }}{2AB ( \alpha ) \Gamma ( \alpha +3 ) } \\ &{}\times \sum_{r=2}^{p} \begin{bmatrix} t_{r}^{1-\beta }\widetilde{I_{D}} ( t_{r},S^{r},I^{r},I_{A}^{ r},I_{D}^{r},I_{R}^{r},I_{T}^{r},R^{r},D^{r},V^{r} ) \\ -2t_{r-1}^{1-\beta }\widetilde{I_{D}} ( t_{r-1},S^{r-1},I^{r-1},I_{A}^{r-1},I_{D}^{r-1},I_{R}^{r-1},I_{T}^{r-1},R^{r-1},D^{r-1},V^{r-1} ) \\ +t_{r-2}^{1-\beta }\widetilde{I_{D}} ( t_{r-2},S^{r-2},I^{r-2},I_{A}^{r-2},I_{D}^{r-2},I_{R}^{r-2},I_{T}^{r-2},R^{r-2},D^{r-2},V^{r-2} )\end{bmatrix} \\ &{}\times \begin{bmatrix} ( p-r+1 ) ^{\alpha } \begin{bmatrix} 2 ( p-r ) ^{2}+ ( 3\alpha +10 ) ( p-r ) \\ +2\alpha ^{2}+9\alpha +12\end{bmatrix} \\ - ( p-r ) ^{\alpha } \begin{bmatrix} 2 ( p-r ) ^{2}+ ( 5\alpha +10 ) ( p-r ) \\ +6\alpha ^{2}+18\alpha +12\end{bmatrix}\end{bmatrix}, \\ I_{R}^{p+1} ={}& \frac{1-\alpha }{AB ( \alpha ) }t_{p}^{1- \beta }\widetilde{I_{R}} \bigl( t_{p},S^{p},I^{p},I_{A}^{p},I_{D}^{ p},I_{R}^{p},I_{T}^{p},R^{p},D^{p},V^{p} \bigr) \\ &{}+ \frac{\alpha ( \Delta t ) ^{\alpha }}{AB ( \alpha ) \Gamma ( \alpha +1 ) }\sum_{r=2}^{p}t_{r-2}^{1-\beta } \widetilde{I_{R}} \bigl( t_{r-2},S^{r-2},I^{r-2},I_{A}^{r-2},I_{D}^{ r-2},I_{R}^{r-2},I_{T}^{r-2},R^{r-2},D^{r-2},V^{r-2} \bigr) \\ &{}\times \bigl[ ( p-r+1 ) ^{\alpha }- ( p-r ) ^{ \alpha } \bigr] \\ &{}+ \frac{\alpha ( \Delta t ) ^{\alpha }}{AB ( \alpha ) \Gamma ( \alpha +2 ) } \\ &{}\times \sum_{r=2}^{p} \begin{bmatrix} t_{r-1}^{1-\beta }\widetilde{I_{R}} ( t_{r-1},S^{r-1},I^{r-1},I_{A}^{r-1},I_{D}^{r-1},I_{R}^{r-1},I_{T}^{r-1},R^{r-1},D^{r-1},V^{r-1} ) \\ -t_{r-2}^{1-\beta }\widetilde{I_{R}} ( t_{r-2},S^{r-2},I^{r-2},I_{A}^{r-2},I_{D}^{r-2},I_{R}^{r-2},I_{T}^{r-2},R^{r-2},D^{r-2},V^{r-2} )\end{bmatrix} \\ &{}\times \begin{bmatrix} ( p-r+1 ) ^{\alpha } ( p-r+3+2\alpha ) \\ - ( p-r ) ^{\alpha } ( p-r+3+3\alpha )\end{bmatrix} \\ &{}+ \frac{\alpha ( \Delta t ) ^{\alpha }}{2AB ( \alpha ) \Gamma ( \alpha +3 ) } \\ &{}\times \sum_{r=2}^{p} \begin{bmatrix} t_{r}^{1-\beta }\widetilde{I_{R}} ( t_{r},S^{r},I^{r},I_{A}^{ r},I_{D}^{r},I_{R}^{r},I_{T}^{r},R^{r},D^{r},V^{r} ) \\ -2t_{r-1}^{1-\beta }\widetilde{I_{R}} ( t_{r-1},S^{r-1},I^{r-1},I_{A}^{r-1},I_{D}^{r-1},I_{R}^{r-1},I_{T}^{r-1},R^{r-1},D^{r-1},V^{r-1} ) \\ +t_{r-2}^{1-\beta }\widetilde{I_{R}} ( t_{r-2},S^{r-2},I^{r-2},I_{A}^{r-2},I_{D}^{r-2},I_{R}^{r-2},I_{T}^{r-2},R^{r-2},D^{r-2},V^{r-2} )\end{bmatrix} \\ &{}\times \begin{bmatrix} ( p-r+1 ) ^{\alpha } \begin{bmatrix} 2 ( p-r ) ^{2}+ ( 3\alpha +10 ) ( p-r ) \\ +2\alpha ^{2}+9\alpha +12\end{bmatrix} \\ - ( p-r ) ^{\alpha } \begin{bmatrix} 2 ( p-r ) ^{2}+ ( 5\alpha +10 ) ( p-r ) \\ +6\alpha ^{2}+18\alpha +12\end{bmatrix}\end{bmatrix}, \\ I_{T}^{p+1} ={}& \frac{1-\alpha }{AB ( \alpha ) }t_{p}^{1- \beta }\widetilde{I_{T}} \bigl( t_{p},S^{p},I^{p},I_{A}^{p},I_{D}^{ p},I_{R}^{p},I_{T}^{p},R^{p},D^{p},V^{p} \bigr) \\ &{}+ \frac{\alpha ( \Delta t ) ^{\alpha }}{AB ( \alpha ) \Gamma ( \alpha +1 ) }\sum_{r=2}^{p}t_{r-2}^{1-\beta } \widetilde{I_{T}} \bigl( t_{r-2},S^{r-2},I^{r-2},I_{A}^{r-2},I_{D}^{ r-2},I_{R}^{r-2},I_{T}^{r-2},R^{r-2},D^{r-2},V^{r-2} \bigr) \\ &{}\times \bigl[ ( p-r+1 ) ^{\alpha }- ( p-r ) ^{ \alpha } \bigr] \\ &{}+ \frac{\alpha ( \Delta t ) ^{\alpha }}{AB ( \alpha ) \Gamma ( \alpha +2 ) } \\ &{}\times \sum_{r=2}^{p} \begin{bmatrix} t_{r-1}^{1-\beta }\widetilde{I_{T}} ( t_{r-1},S^{r-1},I^{r-1},I_{A}^{r-1},I_{D}^{r-1},I_{R}^{r-1},I_{T}^{r-1},R^{r-1},D^{r-1},V^{r-1} ) \\ -t_{r-2}^{1-\beta }\widetilde{I_{T}} ( t_{r-2},S^{r-2},I^{r-2},I_{A}^{r-2},I_{D}^{r-2},I_{R}^{r-2},I_{T}^{r-2},R^{r-2},D^{r-2},V^{r-2} )\end{bmatrix} \end{aligned}$$

$$\begin{aligned} &{}\times \begin{bmatrix} ( p-r+1 ) ^{\alpha } ( p-r+3+2\alpha ) \\ - ( p-r ) ^{\alpha } ( p-r+3+3\alpha )\end{bmatrix} \\ &{}+ \frac{\alpha ( \Delta t ) ^{\alpha }}{2AB ( \alpha ) \Gamma ( \alpha +3 ) } \\ &{}\times \sum_{r=2}^{p} \begin{bmatrix} t_{r}^{1-\beta }\widetilde{I_{T}} ( t_{r},S^{r},I^{r},I_{A}^{ r},I_{D}^{r},I_{R}^{r},I_{T}^{r},R^{r},D^{r},V^{r} ) \\ -2t_{r-1}^{1-\beta }\widetilde{I_{T}} ( t_{r-1},S^{r-1},I^{r-1},I_{A}^{r-1},I_{D}^{r-1},I_{R}^{r-1},I_{T}^{r-1},R^{r-1},D^{r-1},V^{r-1} ) \\ +t_{r-2}^{1-\beta }\widetilde{I_{T}} ( t_{r-2},S^{r-2},I^{r-2},I_{A}^{r-2},I_{D}^{r-2},I_{R}^{r-2},I_{T}^{r-2},R^{r-2},D^{r-2},V^{r-2} )\end{bmatrix} \\ &{}\times \begin{bmatrix} ( p-r+1 ) ^{\alpha } \begin{bmatrix} 2 ( p-r ) ^{2}+ ( 3\alpha +10 ) ( p-r ) \\ +2\alpha ^{2}+9\alpha +12\end{bmatrix} \\ - ( p-r ) ^{\alpha } \begin{bmatrix} 2 ( p-r ) ^{2}+ ( 5\alpha +10 ) ( p-r ) \\ +6\alpha ^{2}+18\alpha +12\end{bmatrix}\end{bmatrix}, \\ R^{p+1} ={}& \frac{1-\alpha }{AB ( \alpha ) }t_{p}^{1- \beta }\widetilde{R} \bigl( t_{p},S^{p},I^{p},I_{A}^{p},I_{D}^{ p},I_{R}^{p},I_{T}^{p},R^{p},D^{p},V^{p} \bigr) \\ &{}+ \frac{\alpha ( \Delta t ) ^{\alpha }}{AB ( \alpha ) \Gamma ( \alpha +1 ) }\sum_{r=2}^{p}t_{r-2}^{1-\beta } \widetilde{R} \bigl( t_{r-2},S^{r-2},I^{r-2},I_{A}^{r-2},I_{D}^{r-2},I_{R}^{r-2},I_{T}^{r-2},R^{r-2},D^{r-2},V^{r-2} \bigr) \\ &{}\times \bigl[ ( p-r+1 ) ^{\alpha }- ( p-r ) ^{ \alpha } \bigr] \\ &{}+ \frac{\alpha ( \Delta t ) ^{\alpha }}{AB ( \alpha ) \Gamma ( \alpha +2 ) } \\ &{}\times \sum_{r=2}^{p} \begin{bmatrix} t_{r-1}^{1-\beta }\widetilde{R} ( t_{r-1},S^{r-1},I^{r-1},I_{A}^{ r-1},I_{D}^{r-1},I_{R}^{r-1},I_{T}^{r-1},R^{r-1},D^{r-1},V^{r-1} ) \\ -t_{r-2}^{1-\beta }\widetilde{R} ( t_{r-2},S^{r-2},I^{r-2},I_{A}^{ r-2},I_{D}^{r-2},I_{R}^{r-2},I_{T}^{r-2},R^{r-2},D^{r-2},V^{r-2} )\end{bmatrix} \\ &{}\times \begin{bmatrix} ( p-r+1 ) ^{\alpha } ( p-r+3+2\alpha ) \\ - ( p-r ) ^{\alpha } ( p-r+3+3\alpha )\end{bmatrix} \\ &{}+ \frac{\alpha ( \Delta t ) ^{\alpha }}{2AB ( \alpha ) \Gamma ( \alpha +3 ) } \\ &{}\times \sum_{r=2}^{p} \begin{bmatrix} t_{r}^{1-\beta }\widetilde{R} ( t_{r},S^{r},I^{r},I_{A}^{ r},I_{D}^{r},I_{R}^{r},I_{T}^{r},R^{r},D^{r},V^{r} ) \\ -2t_{r-1}^{1-\beta }\widetilde{R} ( t_{r-1},S^{r-1},I^{r-1},I_{A}^{ r-1},I_{D}^{r-1},I_{R}^{r-1},I_{T}^{r-1},R^{r-1},D^{r-1},V^{r-1} ) \\ +t_{r-2}^{1-\beta }\widetilde{R} ( t_{r-2},S^{r-2},I^{r-2},I_{A}^{ r-2},I_{D}^{r-2},I_{R}^{r-2},I_{T}^{r-2},R^{r-2},D^{r-2},V^{r-2} )\end{bmatrix} \end{aligned}$$

$$\begin{aligned} &{}\times \begin{bmatrix} ( p-r+1 ) ^{\alpha } \begin{bmatrix} 2 ( p-r ) ^{2}+ ( 3\alpha +10 ) ( p-r ) \\ +2\alpha ^{2}+9\alpha +12\end{bmatrix} \\ - ( p-r ) ^{\alpha } \begin{bmatrix} 2 ( p-r ) ^{2}+ ( 5\alpha +10 ) ( p-r ) \\ +6\alpha ^{2}+18\alpha +12\end{bmatrix}\end{bmatrix}, \\ D^{p+1} ={}& \frac{1-\alpha }{AB ( \alpha ) }t_{p}^{1- \beta }\widetilde{D} \bigl( t_{p},S^{p},I^{p},I_{A}^{p},I_{D}^{ p},I_{R}^{p},I_{T}^{p},R^{p},D^{p},V^{p} \bigr) \\ &{}+ \frac{\alpha ( \Delta t ) ^{\alpha }}{AB ( \alpha ) \Gamma ( \alpha +1 ) }\sum_{r=2}^{p}t_{r-2}^{1-\beta } \widetilde{D} \bigl( t_{r-2},S^{r-2},I^{r-2},I_{A}^{r-2},I_{D}^{r-2},I_{R}^{r-2},I_{T}^{r-2},R^{r-2},D^{r-2},V^{r-2} \bigr) \\ &{}\times \bigl[ ( p-r+1 ) ^{\alpha }- ( p-r ) ^{ \alpha } \bigr] \\ &{}+ \frac{\alpha ( \Delta t ) ^{\alpha }}{AB ( \alpha ) \Gamma ( \alpha +2 ) } \end{aligned}$$

$$\begin{aligned} &{}\times \sum_{r=2}^{p} \begin{bmatrix} t_{r-1}^{1-\beta }\widetilde{D} ( t_{r-1},S^{r-1},I^{r-1},I_{A}^{ r-1},I_{D}^{r-1},I_{R}^{r-1},I_{T}^{r-1},R^{r-1},D^{r-1},V^{r-1} ) \\ -t_{r-2}^{1-\beta }\widetilde{D} ( t_{r-2},S^{r-2},I^{r-2},I_{A}^{ r-2},I_{D}^{r-2},I_{R}^{r-2},I_{T}^{r-2},R^{r-2},D^{r-2},V^{r-2} )\end{bmatrix} \\ &{}\times \begin{bmatrix} ( p-r+1 ) ^{\alpha } ( p-r+3+2\alpha ) \\ - ( p-r ) ^{\alpha } ( p-r+3+3\alpha )\end{bmatrix} \\ &{}+ \frac{\alpha ( \Delta t ) ^{\alpha }}{2AB ( \alpha ) \Gamma ( \alpha +3 ) } \\ &{}\times \sum_{r=2}^{p} \begin{bmatrix} t_{r}^{1-\beta }\widetilde{D} ( t_{r},S^{r},I^{r},I_{A}^{ r},I_{D}^{r},I_{R}^{r},I_{T}^{r},R^{r},D^{r},V^{r} ) \\ -2t_{r-1}^{1-\beta }\widetilde{D} ( t_{r-1},S^{r-1},I^{r-1},I_{A}^{ r-1},I_{D}^{r-1},I_{R}^{r-1},I_{T}^{r-1},R^{r-1},D^{r-1},V^{r-1} ) \\ +t_{r-2}^{1-\beta }\widetilde{D} ( t_{r-2},S^{r-2},I^{r-2},I_{A}^{ r-2},I_{D}^{r-2},I_{R}^{r-2},I_{T}^{r-2},R^{r-2},D^{r-2},V^{r-2} )\end{bmatrix} \\ &{}\times \begin{bmatrix} ( p-r+1 ) ^{\alpha } \begin{bmatrix} 2 ( p-r ) ^{2}+ ( 3\alpha +10 ) ( p-r ) \\ +2\alpha ^{2}+9\alpha +12\end{bmatrix} \\ - ( p-r ) ^{\alpha } \begin{bmatrix} 2 ( p-r ) ^{2}+ ( 5\alpha +10 ) ( p-r ) \\ +6\alpha ^{2}+18\alpha +12\end{bmatrix}\end{bmatrix}, \\ V^{p+1} ={}& \frac{1-\alpha }{AB ( \alpha ) }t_{p}^{1- \beta }\widetilde{V} \bigl( t_{p},S^{p},I^{p},I_{A}^{p},I_{D}^{ p},I_{R}^{p},I_{T}^{p},R^{p},D^{p},V^{p} \bigr) \\ &{}+ \frac{\alpha ( \Delta t ) ^{\alpha }}{AB ( \alpha ) \Gamma ( \alpha +1 ) }\sum_{r=2}^{p}t_{r-2}^{1-\beta } \widetilde{V} \bigl( t_{r-2},S^{r-2},I^{r-2},I_{A}^{r-2},I_{D}^{r-2},I_{R}^{r-2},I_{T}^{r-2},R^{r-2},D^{r-2},V^{r-2} \bigr) \\ &{}\times \bigl[ ( p-r+1 ) ^{\alpha }- ( p-r ) ^{ \alpha } \bigr] \\ &{}+ \frac{\alpha ( \Delta t ) ^{\alpha }}{AB ( \alpha ) \Gamma ( \alpha +2 ) } \\ &{}\times \sum_{r=2}^{p} \begin{bmatrix} t_{r-1}^{1-\beta }\widetilde{V} ( t_{r-1},S^{r-1},I^{r-1},I_{A}^{ r-1},I_{D}^{r-1},I_{R}^{r-1},I_{T}^{r-1},R^{r-1},D^{r-1},V^{r-1} ) \\ -t_{r-2}^{1-\beta }\widetilde{V} ( t_{r-2},S^{r-2},I^{r-2},I_{A}^{ r-2},I_{D}^{r-2},I_{R}^{r-2},I_{T}^{r-2},R^{r-2},D^{r-2},V^{r-2} )\end{bmatrix} \\ &{}\times \begin{bmatrix} ( p-r+1 ) ^{\alpha } ( p-r+3+2\alpha ) \\ - ( p-r ) ^{\alpha } ( p-r+3+3\alpha )\end{bmatrix} \\ &{}+ \frac{\alpha ( \Delta t ) ^{\alpha }}{2AB ( \alpha ) \Gamma ( \alpha +3 ) } \\ &{}\times \sum_{r=2}^{p} \begin{bmatrix} t_{r}^{1-\beta }\widetilde{V} ( t_{r},S^{r},I^{r},I_{A}^{ r},I_{D}^{r},I_{R}^{r},I_{T}^{r},R^{r},D^{r},V^{r} ) \\ -2t_{r-1}^{1-\beta }\widetilde{V} ( t_{r-1},S^{r-1},I^{r-1},I_{A}^{ r-1},I_{D}^{r-1},I_{R}^{r-1},I_{T}^{r-1},R^{r-1},D^{r-1},V^{r-1} ) \\ +t_{r-2}^{1-\beta }\widetilde{V} ( t_{r-2},S^{r-2},I^{r-2},I_{A}^{ r-2},I_{D}^{r-2},I_{R}^{r-2},I_{T}^{r-2},R^{r-2},D^{r-2},V^{r-2} )\end{bmatrix} \\ &{}\times \begin{bmatrix} ( p-r+1 ) ^{\alpha } \begin{bmatrix} 2 ( p-r ) ^{2}+ ( 3\alpha +10 ) ( p-r ) \\ +2\alpha ^{2}+9\alpha +12\end{bmatrix} \\ - ( p-r ) ^{\alpha } \begin{bmatrix} 2 ( p-r ) ^{2}+ ( 5\alpha +10 ) ( p-r ) \\ +6\alpha ^{2}+18\alpha +12\end{bmatrix}\end{bmatrix}. \end{aligned}$$

For the power-law kernel, we have the following:

5.30 $$\begin{aligned} &S^{p+1}=\frac{1}{\Gamma ( \alpha ) }\sum_{r=2}^{p}\int _{t_{r}}^{t_{r+1}}\widetilde{S} ( \tau,S,I,I_{A},I_{D},I_{R},I_{T},R,D,V ) ( t_{p+1}-\tau ) ^{\alpha -1}\tau ^{1-\beta }\,d \tau, \\ &I^{p+1}=\frac{1}{\Gamma ( \alpha ) }\sum_{r=2}^{p}\int _{t_{r}}^{t_{r+1}}\widetilde{I} ( \tau,S,I,I_{A},I_{D},I_{R},I_{T},R,D,V ) ( t_{p+1}-\tau ) ^{\alpha -1}\tau ^{1-\beta }\,d \tau, \\ &I_{A}^{p+1}=\frac{1}{\Gamma ( \alpha ) }\sum _{r=2}^{p}\int _{t_{r}}^{t_{r+1}}\widetilde{I_{A}} ( \tau,S,I,I_{A},I_{D},I_{R},I_{T},R,D,V ) ( t_{p+1}-\tau ) ^{\alpha -1}\tau ^{1-\beta }\,d \tau, \\ &I_{D}^{p+1}=\frac{1}{\Gamma ( \alpha ) }\sum _{r=2}^{p}\int _{t_{r}}^{t_{r+1}}\widetilde{I_{D}} ( \tau,S,I,I_{A},I_{D},I_{R},I_{T},R,D,V ) ( t_{p+1}-\tau ) ^{\alpha -1}\tau ^{1-\beta }\,d \tau, \\ &I_{R}^{p+1}=\frac{1}{\Gamma ( \alpha ) }\sum _{r=2}^{p}\int _{t_{r}}^{t_{r+1}}\widetilde{I_{R}} ( \tau,S,I,I_{A},I_{D},I_{R},I_{T},R,D,V ) ( t_{p+1}-\tau ) ^{\alpha -1}\tau ^{1-\beta }\,d \tau, \\ &I_{T}^{p+1}=\frac{1}{\Gamma ( \alpha ) }\sum _{r=2}^{p}\int _{t_{r}}^{t_{r+1}}\widetilde{I_{T}} ( \tau,S,I,I_{A},I_{D},I_{R},I_{T},R,D,V ) ( t_{p+1}-\tau ) ^{\alpha -1}\tau ^{1-\beta }\,d \tau, \\ &R^{p+1}=\frac{1}{\Gamma ( \alpha ) }\sum_{r=2}^{p}\int _{t_{r}}^{t_{r+1}}\widetilde{R} ( \tau,S,I,I_{A},I_{D},I_{R},I_{T},R,D,V ) ( t_{p+1}-\tau ) ^{\alpha -1}\tau ^{1-\beta }\,d \tau, \\ &D^{p+1}=\frac{1}{\Gamma ( \alpha ) }\sum_{r=2}^{p}\int _{t_{r}}^{t_{r+1}}\widetilde{D} ( \tau,S,I,I_{A},I_{D},I_{R},I_{T},R,D,V ) ( t_{p+1}-\tau ) ^{\alpha -1}\tau ^{1-\beta }\,d \tau, \\ &V^{p+1}=\frac{1}{\Gamma ( \alpha ) }\sum_{r=2}^{p}\int _{t_{r}}^{t_{r+1}}\widetilde{V} ( \tau,S,I,I_{A},I_{D},I_{R},I_{T},R,D,V ) ( t_{p+1}-\tau ) ^{\alpha -1}\tau ^{1-\beta }\,d \tau. \end{aligned}$$

We can get the following numerical scheme:

5.31 $$\begin{aligned} S^{p+1} ={}& \frac{ ( \Delta t ) ^{\alpha }}{\Gamma ( \alpha +1 ) } \sum_{r=2}^{p}t_{r-2}^{1-\beta } \widetilde{S} \bigl( t_{r-2},S^{r-2},I^{r-2},I_{A}^{ r-2},I_{D}^{r-2},I_{R}^{r-2},I_{T}^{r-2},R^{r-2},D^{r-2},V^{r-2} \bigr) \\ &{}\times \bigl[ ( p-r+1 ) ^{\alpha }- ( p-r ) ^{ \alpha } \bigr] \\ &{}+ \frac{ ( \Delta t ) ^{\alpha }}{\Gamma ( \alpha +2 ) }\sum_{r=2}^{p} \begin{bmatrix} t_{r-1}^{1-\beta }\widetilde{S} ( t_{r-1},S^{r-1},I^{r-1},I_{A}^{ r-1},I_{D}^{r-1},I_{R}^{r-1},I_{T}^{r-1},R^{r-1},D^{r-1},V^{r-1} ) \\ -t_{r-2}^{1-\beta }\widetilde{S} ( t_{r-2},S^{r-2},I^{r-2},I_{A}^{ r-2},I_{D}^{r-2},I_{R}^{r-2},I_{T}^{r-2},R^{r-2},D^{r-2},V^{r-2} )\end{bmatrix} \\ &{}\times \begin{bmatrix} ( p-r+1 ) ^{\alpha } ( p-r+3+2\alpha ) \\ - ( p-r ) ^{\alpha } ( p-r+3+3\alpha )\end{bmatrix} \\ &{}+ \frac{ ( \Delta t ) ^{\alpha }}{2\Gamma ( \alpha +3 ) }\sum_{r=2}^{p} \begin{bmatrix} t_{r}^{1-\beta }\widetilde{S} ( t_{r},S^{r},I^{r},I_{A}^{ r},I_{D}^{r},I_{R}^{r},I_{T}^{r},R^{r},D^{r},V^{r} ) \\ -2t_{r-1}^{1-\beta }\widetilde{S} ( t_{r-1},S^{r-1},I^{r-1},I_{A}^{ r-1},I_{D}^{r-1},I_{R}^{r-1},I_{T}^{r-1},R^{r-1},D^{r-1},V^{r-1} ) \\ +t_{r-2}^{1-\beta }\widetilde{S} ( t_{r-2},S^{r-2},I^{r-2},I_{A}^{ r-2},I_{D}^{r-2},I_{R}^{r-2},I_{T}^{r-2},R^{r-2},D^{r-2},V^{r-2} )\end{bmatrix} \\ &{}\times \begin{bmatrix} ( p-r+1 ) ^{\alpha } \begin{bmatrix} 2 ( p-r ) ^{2}+ ( 3\alpha +10 ) ( p-r ) \\ +2\alpha ^{2}+9\alpha +12\end{bmatrix} \\ - ( p-r ) ^{\alpha } \begin{bmatrix} 2 ( p-r ) ^{2}+ ( 5\alpha +10 ) ( p-r ) \\ +6\alpha ^{2}+18\alpha +12\end{bmatrix}\end{bmatrix}, \\ I^{p+1} ={}& \frac{ ( \Delta t ) ^{\alpha }}{\Gamma ( \alpha +1 ) } \sum_{r=2}^{p}t_{r-2}^{1-\beta } \widetilde{I} \bigl( t_{r-2},S^{r-2},I^{r-2},I_{A}^{ r-2},I_{D}^{r-2},I_{R}^{r-2},I_{T}^{r-2},R^{r-2},D^{r-2},V^{r-2} \bigr) \\ &{}\times \bigl[ ( p-r+1 ) ^{\alpha }- ( p-r ) ^{ \alpha } \bigr] \\ &{}+ \frac{ ( \Delta t ) ^{\alpha }}{\Gamma ( \alpha +2 ) }\sum_{r=2}^{p} \begin{bmatrix} t_{r-1}^{1-\beta }\widetilde{I} ( t_{r-1},S^{r-1},I^{r-1},I_{A}^{ r-1},I_{D}^{r-1},I_{R}^{r-1},I_{T}^{r-1},R^{r-1},D^{r-1},V^{r-1} ) \\ -t_{r-2}^{1-\beta }\widetilde{I} ( t_{r-2},S^{r-2},I^{r-2},I_{A}^{ r-2},I_{D}^{r-2},I_{R}^{r-2},I_{T}^{r-2},R^{r-2},D^{r-2},V^{r-2} )\end{bmatrix} \\ &{}\times \begin{bmatrix} ( p-r+1 ) ^{\alpha } ( p-r+3+2\alpha ) \\ - ( p-r ) ^{\alpha } ( p-r+3+3\alpha )\end{bmatrix} \\ &{}+ \frac{ ( \Delta t ) ^{\alpha }}{2\Gamma ( \alpha +3 ) }\sum_{r=2}^{p} \begin{bmatrix} t_{r}^{1-\beta }\widetilde{I} ( t_{r},S^{r},I^{r},I_{A}^{ r},I_{D}^{r},I_{R}^{r},I_{T}^{r},R^{r},D^{r},V^{r} ) \\ -2t_{r-1}^{1-\beta }\widetilde{I} ( t_{r-1},S^{r-1},I^{r-1},I_{A}^{ r-1},I_{D}^{r-1},I_{R}^{r-1},I_{T}^{r-1},R^{r-1},D^{r-1},V^{r-1} ) \\ +t_{r-2}^{1-\beta }\widetilde{I} ( t_{r-2},S^{r-2},I^{r-2},I_{A}^{ r-2},I_{D}^{r-2},I_{R}^{r-2},I_{T}^{r-2},R^{r-2},D^{r-2},V^{r-2} )\end{bmatrix} \\ &{}\times \begin{bmatrix} ( p-r+1 ) ^{\alpha } \begin{bmatrix} 2 ( p-r ) ^{2}+ ( 3\alpha +10 ) ( p-r ) \\ +2\alpha ^{2}+9\alpha +12\end{bmatrix} \\ - ( p-r ) ^{\alpha } \begin{bmatrix} 2 ( p-r ) ^{2}+ ( 5\alpha +10 ) ( p-r ) \\ +6\alpha ^{2}+18\alpha +12\end{bmatrix}\end{bmatrix}, \\ I_{A}^{p+1} ={}& \frac{ ( \Delta t ) ^{\alpha }}{\Gamma ( \alpha +1 ) } \sum _{r=2}^{p}t_{r-2}^{1-\beta } \widetilde{I_{A}} \bigl( t_{r-2},S^{r-2},I^{r-2},I_{A}^{ r-2},I_{D}^{r-2},I_{R}^{r-2},I_{T}^{r-2},R^{r-2},D^{r-2},V^{r-2} \bigr) \\ &{}\times \bigl[ ( p-r+1 ) ^{\alpha }- ( p-r ) ^{ \alpha } \bigr] \\ &{}+ \frac{ ( \Delta t ) ^{\alpha }}{\Gamma ( \alpha +2 ) }\sum_{r=2}^{p} \begin{bmatrix} t_{r-1}^{1-\beta }\widetilde{I_{A}} ( t_{r-1},S^{r-1},I^{r-1},I_{A}^{r-1},I_{D}^{r-1},I_{R}^{r-1},I_{T}^{r-1},R^{r-1},D^{r-1},V^{r-1} ) \\ -t_{r-2}^{1-\beta }\widetilde{I_{A}} ( t_{r-2},S^{r-2},I^{r-2},I_{A}^{r-2},I_{D}^{r-2},I_{R}^{r-2},I_{T}^{r-2},R^{r-2},D^{r-2},V^{r-2} )\end{bmatrix} \\ &{}\times \begin{bmatrix} ( p-r+1 ) ^{\alpha } ( p-r+3+2\alpha ) \\ - ( p-r ) ^{\alpha } ( p-r+3+3\alpha )\end{bmatrix} \\ &{}+ \frac{ ( \Delta t ) ^{\alpha }}{2\Gamma ( \alpha +3 ) }\sum_{r=2}^{p} \begin{bmatrix} t_{r}^{1-\beta }\widetilde{I_{A}} ( t_{r},S^{r},I^{r},I_{A}^{ r},I_{D}^{r},I_{R}^{r},I_{T}^{r},R^{r},D^{r},V^{r} ) \\ -2t_{r-1}^{1-\beta }\widetilde{I_{A}} ( t_{r-1},S^{r-1},I^{r-1},I_{A}^{r-1},I_{D}^{r-1},I_{R}^{r-1},I_{T}^{r-1},R^{r-1},D^{r-1},V^{r-1} ) \\ +t_{r-2}^{1-\beta }\widetilde{I_{A}} ( t_{r-2},S^{r-2},I^{r-2},I_{A}^{r-2},I_{D}^{r-2},I_{R}^{r-2},I_{T}^{r-2},R^{r-2},D^{r-2},V^{r-2} )\end{bmatrix} \\ &{}\times \begin{bmatrix} ( p-r+1 ) ^{\alpha } \begin{bmatrix} 2 ( p-r ) ^{2}+ ( 3\alpha +10 ) ( p-r ) \\ +2\alpha ^{2}+9\alpha +12\end{bmatrix} \\ - ( p-r ) ^{\alpha } \begin{bmatrix} 2 ( p-r ) ^{2}+ ( 5\alpha +10 ) ( p-r ) \\ +6\alpha ^{2}+18\alpha +12\end{bmatrix}\end{bmatrix}, \\ I_{D}^{p+1} ={}& \frac{ ( \Delta t ) ^{\alpha }}{\Gamma ( \alpha +1 ) } \sum _{r=2}^{p}t_{r-2}^{1-\beta } \widetilde{I_{D}} \bigl( t_{r-2},S^{r-2},I^{r-2},I_{A}^{ r-2},I_{D}^{r-2},I_{R}^{r-2},I_{T}^{r-2},R^{r-2},D^{r-2},V^{r-2} \bigr) \\ &{}\times \bigl[ ( p-r+1 ) ^{\alpha }- ( p-r ) ^{ \alpha } \bigr] \\ &{}+ \frac{ ( \Delta t ) ^{\alpha }}{\Gamma ( \alpha +2 ) }\sum_{r=2}^{p} \begin{bmatrix} t_{r-1}^{1-\beta }\widetilde{I_{D}} ( t_{r-1},S^{r-1},I^{r-1},I_{A}^{r-1},I_{D}^{r-1},I_{R}^{r-1},I_{T}^{r-1},R^{r-1},D^{r-1},V^{r-1} ) \\ -t_{r-2}^{1-\beta }\widetilde{I_{D}} ( t_{r-2},S^{r-2},I^{r-2},I_{A}^{r-2},I_{D}^{r-2},I_{R}^{r-2},I_{T}^{r-2},R^{r-2},D^{r-2},V^{r-2} )\end{bmatrix} \\ &{}\times \begin{bmatrix} ( p-r+1 ) ^{\alpha } ( p-r+3+2\alpha ) \\ - ( p-r ) ^{\alpha } ( p-r+3+3\alpha )\end{bmatrix} \\ &{}+ \frac{ ( \Delta t ) ^{\alpha }}{2\Gamma ( \alpha +3 ) }\sum_{r=2}^{p} \begin{bmatrix} t_{r}^{1-\beta }\widetilde{I_{D}} ( t_{r},S^{r},I^{r},I_{A}^{ r},I_{D}^{r},I_{R}^{r},I_{T}^{r},R^{r},D^{r},V^{r} ) \\ -2t_{r-1}^{1-\beta }\widetilde{I_{D}} ( t_{r-1},S^{r-1},I^{r-1},I_{A}^{r-1},I_{D}^{r-1},I_{R}^{r-1},I_{T}^{r-1},R^{r-1},D^{r-1},V^{r-1} ) \\ +t_{r-2}^{1-\beta }\widetilde{I_{D}} ( t_{r-2},S^{r-2},I^{r-2},I_{A}^{r-2},I_{D}^{r-2},I_{R}^{r-2},I_{T}^{r-2},R^{r-2},D^{r-2},V^{r-2} )\end{bmatrix} \\ &{}\times \begin{bmatrix} ( p-r+1 ) ^{\alpha } \begin{bmatrix} 2 ( p-r ) ^{2}+ ( 3\alpha +10 ) ( p-r ) \\ +2\alpha ^{2}+9\alpha +12\end{bmatrix} \\ - ( p-r ) ^{\alpha } \begin{bmatrix} 2 ( p-r ) ^{2}+ ( 5\alpha +10 ) ( p-r ) \\ +6\alpha ^{2}+18\alpha +12\end{bmatrix}\end{bmatrix}, \\ I_{R}^{p+1} ={}& \frac{ ( \Delta t ) ^{\alpha }}{\Gamma ( \alpha +1 ) } \sum _{r=2}^{p}t_{r-2}^{1-\beta } \widetilde{I_{R}} \bigl( t_{r-2},S^{r-2},I^{r-2},I_{A}^{ r-2},I_{D}^{r-2},I_{R}^{r-2},I_{T}^{r-2},R^{r-2},D^{r-2},V^{r-2} \bigr) \\ &{}\times \bigl[ ( p-r+1 ) ^{\alpha }- ( p-r ) ^{ \alpha } \bigr] \\ &{}+ \frac{ ( \Delta t ) ^{\alpha }}{\Gamma ( \alpha +2 ) }\sum_{r=2}^{p} \begin{bmatrix} t_{r-1}^{1-\beta }\widetilde{I_{R}} ( t_{r-1},S^{r-1},I^{r-1},I_{A}^{r-1},I_{D}^{r-1},I_{R}^{r-1},I_{T}^{r-1},R^{r-1},D^{r-1},V^{r-1} ) \\ -t_{r-2}^{1-\beta }\widetilde{I_{R}} ( t_{r-2},S^{r-2},I^{r-2},I_{A}^{r-2},I_{D}^{r-2},I_{R}^{r-2},I_{T}^{r-2},R^{r-2},D^{r-2},V^{r-2} )\end{bmatrix} \\ &{}\times \begin{bmatrix} ( p-r+1 ) ^{\alpha } ( p-r+3+2\alpha ) \\ - ( p-r ) ^{\alpha } ( p-r+3+3\alpha )\end{bmatrix} \\ &{}+ \frac{ ( \Delta t ) ^{\alpha }}{2\Gamma ( \alpha +3 ) }\sum_{r=2}^{p} \begin{bmatrix} t_{r}^{1-\beta }\widetilde{I_{R}} ( t_{r},S^{r},I^{r},I_{A}^{ r},I_{D}^{r},I_{R}^{r},I_{T}^{r},R^{r},D^{r},V^{r} ) \\ -2t_{r-1}^{1-\beta }\widetilde{I_{R}} ( t_{r-1},S^{r-1},I^{r-1},I_{A}^{r-1},I_{D}^{r-1},I_{R}^{r-1},I_{T}^{r-1},R^{r-1},D^{r-1},V^{r-1} ) \\ +t_{r-2}^{1-\beta }\widetilde{I_{R}} ( t_{r-2},S^{r-2},I^{r-2},I_{A}^{r-2},I_{D}^{r-2},I_{R}^{r-2},I_{T}^{r-2},R^{r-2},D^{r-2},V^{r-2} )\end{bmatrix} \\ &{}\times \begin{bmatrix} ( p-r+1 ) ^{\alpha } \begin{bmatrix} 2 ( p-r ) ^{2}+ ( 3\alpha +10 ) ( p-r ) \\ +2\alpha ^{2}+9\alpha +12\end{bmatrix} \\ - ( p-r ) ^{\alpha } \begin{bmatrix} 2 ( p-r ) ^{2}+ ( 5\alpha +10 ) ( p-r ) \\ +6\alpha ^{2}+18\alpha +12\end{bmatrix}\end{bmatrix}, \\ I_{T}^{p+1} ={}& \frac{\alpha ( \Delta t ) ^{\alpha }}{\Gamma ( \alpha +1 ) }\sum _{r=2}^{p}t_{r-2}^{1-\beta } \widetilde{I_{T}} \bigl( t_{r-2},S^{r-2},I^{r-2},I_{A}^{r-2},I_{D}^{r-2},I_{R}^{ r-2},I_{T}^{r-2},R^{r-2},D^{r-2},V^{r-2} \bigr) \\ &{}\times \bigl[ ( p-r+1 ) ^{\alpha }- ( p-r ) ^{ \alpha } \bigr] \\ &{}+ \frac{ ( \Delta t ) ^{\alpha }}{\Gamma ( \alpha +2 ) }\sum_{r=2}^{p} \begin{bmatrix} t_{r-1}^{1-\beta }\widetilde{I_{T}} ( t_{r-1},S^{r-1},I^{r-1},I_{A}^{r-1},I_{D}^{r-1},I_{R}^{r-1},I_{T}^{r-1},R^{r-1},D^{r-1},V^{r-1} ) \\ -t_{r-2}^{1-\beta }\widetilde{I_{T}} ( t_{r-2},S^{r-2},I^{r-2},I_{A}^{r-2},I_{D}^{r-2},I_{R}^{r-2},I_{T}^{r-2},R^{r-2},D^{r-2},V^{r-2} )\end{bmatrix} \\ &{}\times \begin{bmatrix} ( p-r+1 ) ^{\alpha } ( p-r+3+2\alpha ) \\ - ( p-r ) ^{\alpha } ( p-r+3+3\alpha )\end{bmatrix} \\ &{}+ \frac{ ( \Delta t ) ^{\alpha }}{2\Gamma ( \alpha +3 ) }\sum_{r=2}^{p} \begin{bmatrix} t_{r}^{1-\beta }\widetilde{I_{T}} ( t_{r},S^{r},I^{r},I_{A}^{ r},I_{D}^{r},I_{R}^{r},I_{T}^{r},R^{r},D^{r},V^{r} ) \\ -2t_{r-1}^{1-\beta }\widetilde{I_{T}} ( t_{r-1},S^{r-1},I^{r-1},I_{A}^{r-1},I_{D}^{r-1},I_{R}^{r-1},I_{T}^{r-1},R^{r-1},D^{r-1},V^{r-1} ) \\ +t_{r-2}^{1-\beta }\widetilde{I_{T}} ( t_{r-2},S^{r-2},I^{r-2},I_{A}^{r-2},I_{D}^{r-2},I_{R}^{r-2},I_{T}^{r-2},R^{r-2},D^{r-2},V^{r-2} )\end{bmatrix} \\ &{}\times \begin{bmatrix} ( p-r+1 ) ^{\alpha } \begin{bmatrix} 2 ( p-r ) ^{2}+ ( 3\alpha +10 ) ( p-r ) \\ +2\alpha ^{2}+9\alpha +12\end{bmatrix} \\ - ( p-r ) ^{\alpha } \begin{bmatrix} 2 ( p-r ) ^{2}+ ( 5\alpha +10 ) ( p-r ) \\ +6\alpha ^{2}+18\alpha +12\end{bmatrix}\end{bmatrix}, \\ R^{p+1} ={}& \frac{ ( \Delta t ) ^{\alpha }}{\Gamma ( \alpha +1 ) } \sum_{r=2}^{p}t_{r-2}^{1-\beta } \widetilde{R} \bigl( t_{r-2},S^{r-2},I^{r-2},I_{A}^{ r-2},I_{D}^{r-2},I_{R}^{r-2},I_{T}^{r-2},R^{r-2},D^{r-2},V^{r-2} \bigr) \\ &{}\times \bigl[ ( p-r+1 ) ^{\alpha }- ( p-r ) ^{ \alpha } \bigr] \\ &{}+ \frac{ ( \Delta t ) ^{\alpha }}{\Gamma ( \alpha +2 ) }\sum_{r=2}^{p} \begin{bmatrix} t_{r-1}^{1-\beta }\widetilde{R} ( t_{r-1},S^{r-1},I^{r-1},I_{A}^{ r-1},I_{D}^{r-1},I_{R}^{r-1},I_{T}^{r-1},R^{r-1},D^{r-1},V^{r-1} ) \\ -t_{r-2}^{1-\beta }\widetilde{R} ( t_{r-2},S^{r-2},I^{r-2},I_{A}^{ r-2},I_{D}^{r-2},I_{R}^{r-2},I_{T}^{r-2},R^{r-2},D^{r-2},V^{r-2} )\end{bmatrix} \\ &{}\times \begin{bmatrix} ( p-r+1 ) ^{\alpha } ( p-r+3+2\alpha ) \\ - ( p-r ) ^{\alpha } ( p-r+3+3\alpha )\end{bmatrix} \\ &{}+ \frac{ ( \Delta t ) ^{\alpha }}{2\Gamma ( \alpha +3 ) }\sum_{r=2}^{p} \begin{bmatrix} t_{r}^{1-\beta }\widetilde{R} ( t_{r},S^{r},I^{r},I_{A}^{ r},I_{D}^{r},I_{R}^{r},I_{T}^{r},R^{r},D^{r},V^{r} ) \\ -2t_{r-1}^{1-\beta }\widetilde{R} ( t_{r-1},S^{r-1},I^{r-1},I_{A}^{ r-1},I_{D}^{r-1},I_{R}^{r-1},I_{T}^{r-1},R^{r-1},D^{r-1},V^{r-1} ) \\ +t_{r-2}^{1-\beta }\widetilde{R} ( t_{r-2},S^{r-2},I^{r-2},I_{A}^{ r-2},I_{D}^{r-2},I_{R}^{r-2},I_{T}^{r-2},R^{r-2},D^{r-2},V^{r-2} )\end{bmatrix} \\ &{}\times \begin{bmatrix} ( p-r+1 ) ^{\alpha } \begin{bmatrix} 2 ( p-r ) ^{2}+ ( 3\alpha +10 ) ( p-r ) \\ +2\alpha ^{2}+9\alpha +12\end{bmatrix} \\ - ( p-r ) ^{\alpha } \begin{bmatrix} 2 ( p-r ) ^{2}+ ( 5\alpha +10 ) ( p-r ) \\ +6\alpha ^{2}+18\alpha +12\end{bmatrix}\end{bmatrix}, \\ D^{p+1} ={}& \frac{ ( \Delta t ) ^{\alpha }}{\Gamma ( \alpha +1 ) } \sum_{r=2}^{p}t_{r-2}^{1-\beta } \widetilde{D} \bigl( t_{r-2},S^{r-2},I^{r-2},I_{A}^{ r-2},I_{D}^{r-2},I_{R}^{r-2},I_{T}^{r-2},R^{r-2},D^{r-2},V^{r-2} \bigr) \\ &{}\times \bigl[ ( p-r+1 ) ^{\alpha }- ( p-r ) ^{ \alpha } \bigr] \\ &{}+ \frac{ ( \Delta t ) ^{\alpha }}{\Gamma ( \alpha +2 ) }\sum_{r=2}^{p} \begin{bmatrix} t_{r-1}^{1-\beta }\widetilde{D} ( t_{r-1},S^{r-1},I^{r-1},I_{A}^{ r-1},I_{D}^{r-1},I_{R}^{r-1},I_{T}^{r-1},R^{r-1},D^{r-1},V^{r-1} ) \\ -t_{r-2}^{1-\beta }\widetilde{D} ( t_{r-2},S^{r-2},I^{r-2},I_{A}^{ r-2},I_{D}^{r-2},I_{R}^{r-2},I_{T}^{r-2},R^{r-2},D^{r-2},V^{r-2} )\end{bmatrix} \\ &{}\times \begin{bmatrix} ( p-r+1 ) ^{\alpha } ( p-r+3+2\alpha ) \\ - ( p-r ) ^{\alpha } ( p-r+3+3\alpha )\end{bmatrix} \\ &{}+ \frac{ ( \Delta t ) ^{\alpha }}{2\Gamma ( \alpha +3 ) }\sum_{r=2}^{p} \begin{bmatrix} t_{r}^{1-\beta }\widetilde{D} ( t_{r},S^{r},I^{r},I_{A}^{ r},I_{D}^{r},I_{R}^{r},I_{T}^{r},R^{r},D^{r},V^{r} ) \\ -2t_{r-1}^{1-\beta }\widetilde{D} ( t_{r-1},S^{r-1},I^{r-1},I_{A}^{ r-1},I_{D}^{r-1},I_{R}^{r-1},I_{T}^{r-1},R^{r-1},D^{r-1},V^{r-1} ) \\ +t_{r-2}^{1-\beta }\widetilde{D} ( t_{r-2},S^{r-2},I^{r-2},I_{A}^{ r-2},I_{D}^{r-2},I_{R}^{r-2},I_{T}^{r-2},R^{r-2},D^{r-2},V^{r-2} )\end{bmatrix} \\ &{}\times \begin{bmatrix} ( p-r+1 ) ^{\alpha } \begin{bmatrix} 2 ( p-r ) ^{2}+ ( 3\alpha +10 ) ( p-r ) \\ +2\alpha ^{2}+9\alpha +12\end{bmatrix} \\ - ( p-r ) ^{\alpha } \begin{bmatrix} 2 ( p-r ) ^{2}+ ( 5\alpha +10 ) ( p-r ) \\ +6\alpha ^{2}+18\alpha +12\end{bmatrix}\end{bmatrix}, \\ V^{p+1} ={}& \frac{ ( \Delta t ) ^{\alpha }}{\Gamma ( \alpha +1 ) } \sum_{r=2}^{p}t_{r-2}^{1-\beta } \widetilde{V} \bigl( t_{r-2},S^{r-2},I^{r-2},I_{A}^{ r-2},I_{D}^{r-2},I_{R}^{r-2},I_{T}^{r-2},R^{r-2},D^{r-2},V^{r-2} \bigr) \\ &{}\times \bigl[ ( p-r+1 ) ^{\alpha }- ( p-r ) ^{ \alpha } \bigr] \\ &{}+ \frac{ ( \Delta t ) ^{\alpha }}{\Gamma ( \alpha +2 ) }\sum_{r=2}^{p} \begin{bmatrix} t_{r-1}^{1-\beta }\widetilde{V} ( t_{r-1},S^{r-1},I^{r-1},I_{A}^{ r-1},I_{D}^{r-1},I_{R}^{r-1},I_{T}^{r-1},R^{r-1},D^{r-1},V^{r-1} ) \\ -t_{r-2}^{1-\beta }\widetilde{V} ( t_{r-2},S^{r-2},I^{r-2},I_{A}^{ r-2},I_{D}^{r-2},I_{R}^{r-2},I_{T}^{r-2},R^{r-2},D^{r-2},V^{r-2} )\end{bmatrix} \\ &{}\times \begin{bmatrix} ( p-r+1 ) ^{\alpha } ( p-r+3+2\alpha ) \\ - ( p-r ) ^{\alpha } ( p-r+3+3\alpha )\end{bmatrix} \\ &{}+ \frac{ ( \Delta t ) ^{\alpha }}{2\Gamma ( \alpha +3 ) }\sum_{r=2}^{p} \begin{bmatrix} t_{r}^{1-\beta }\widetilde{V} ( t_{r},S^{r},I^{r},I_{A}^{ r},I_{D}^{r},I_{R}^{r},I_{T}^{r},R^{r},D^{r},V^{r} ) \\ -2t_{r-1}^{1-\beta }\widetilde{V} ( t_{r-1},S^{r-1},I^{r-1},I_{A}^{ r-1},I_{D}^{r-1},I_{R}^{r-1},I_{T}^{r-1},R^{r-1},D^{r-1},V^{r-1} ) \\ +t_{r-2}^{1-\beta }\widetilde{V} ( t_{r-2},S^{r-2},I^{r-2},I_{A}^{ r-2},I_{D}^{r-2},I_{R}^{r-2},I_{T}^{r-2},R^{r-2},D^{r-2},V^{r-2} )\end{bmatrix} \\ &{}\times \begin{bmatrix} ( p-r+1 ) ^{\alpha } \begin{bmatrix} 2 ( p-r ) ^{2}+ ( 3\alpha +10 ) ( p-r ) \\ +2\alpha ^{2}+9\alpha +12\end{bmatrix} \\ - ( p-r ) ^{\alpha } \begin{bmatrix} 2 ( p-r ) ^{2}+ ( 5\alpha +10 ) ( p-r ) \\ +6\alpha ^{2}+18\alpha +12\end{bmatrix}\end{bmatrix}. \end{aligned}$$

Now, we handle the following model:

5.32 $$\begin{aligned} &{}_{0}^{FFM}D_{t}^{\alpha,\beta }S = \Lambda - \bigl( \alpha ( x ) +\gamma _{1}+\mu _{1} \bigr) S, \\ &{}_{0}^{FFM}D_{t}^{\alpha,\beta }I = \alpha ( x ) S- ( \varepsilon +\xi +\lambda +\mu _{1} ) I, \\ &{}_{0}^{FFM}D_{t}^{\alpha,\beta }I_{A} = \xi I- ( \theta +\mu + \chi +\mu _{1} ) I_{A}, \\ &{}_{0}^{FFM}D_{t}^{\alpha,\beta }I_{D} = \varepsilon I- ( \eta + \varphi +\mu _{1} ) I_{D}, \\ &{}_{0}^{FFM}D_{t}^{\alpha,\beta }I_{R} = \eta I_{D}+\theta I_{A}- ( v+\xi +\mu _{1} ) I_{R}, \\ &{}_{0}^{FFM}D_{t}^{\alpha,\beta }I_{T} = \mu I_{A}+vI_{R}- ( \sigma +\tau +\mu _{1} ) I_{T}, \\ &{}_{0}^{FFM}D_{t}^{\alpha,\beta }R = \lambda I+\varphi I_{D}+ \chi I_{A}+\xi I_{R}+\sigma I_{T}- ( \Phi +\mu _{1} ) R, \\ &{}_{0}^{FFM}D_{t}^{\alpha,\beta }D = \tau I_{T}, \\ &{}_{0}^{FFM}D_{t}^{\alpha,\beta }V = \gamma _{1}S+\Phi R-\mu _{1}V, \end{aligned}$$

where the initial conditions are

5.33 $$\begin{aligned} &S ( 0 ) = 57780000,\qquad I ( 0 ) =1,\qquad I_{A} ( 0 ) =1,\qquad I_{D} ( 0 ) =1,\qquad I_{R} ( 0 ) =1, \\ &I_{T} ( 0 ) = 1,\qquad R ( 0 ) =0,\qquad D ( 0 ) =0,\qquad V ( 0 ) =0. \end{aligned}$$

Also the parameters are chosen as follows:

5.34 $$\begin{aligned} \begin{aligned} &\Lambda = 57000000,\qquad k=3,\qquad p=0.5,\qquad \eta =0.12,\qquad \chi =0.015,\\ & v=0.027,\qquad x=0.4, \qquad \theta =0.301, \qquad \gamma = 0.09,\qquad \beta =0.013,\\ & \gamma _{1}=0.4,\qquad \mu _{1}=0.3,\qquad \varepsilon =0.161,\qquad \xi =0.015,\qquad \sigma =0.015,\\ & \tau = 0.0199,\qquad \Phi =0.2,\qquad \lambda =0.0345,\qquad\varphi =0.0345,\qquad \delta _{1}=0.01. \end{aligned} \end{aligned}$$

**Figure 41:**
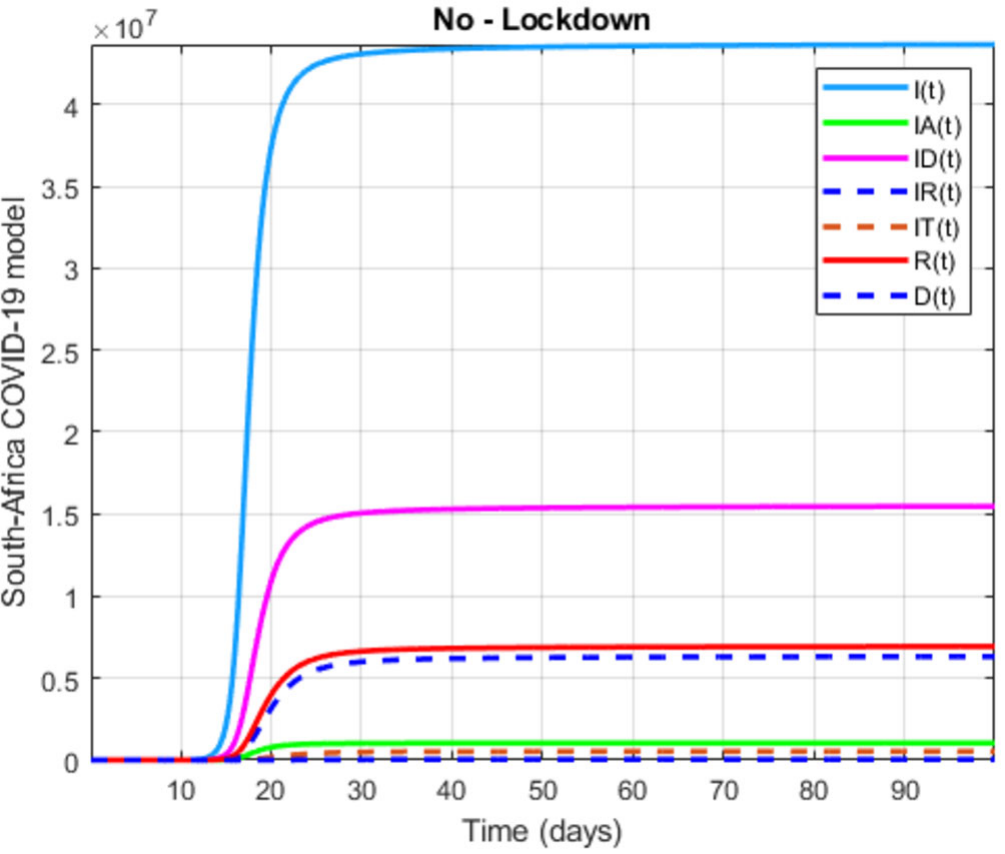
Numerical visualization for Covid-19 model in South Africa for $\alpha =0.9,\beta =0.75$

**Figure 42:**
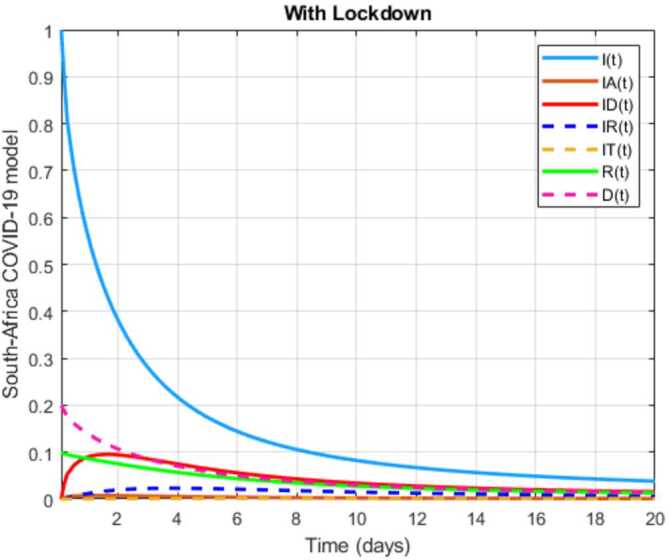
Numerical visualization for Covid-19 model in South Africa for $\alpha =0.7,\beta =0.75$

We present a numerical simulation for Covid-19 model in Figs. 41 and 42.

**Figure 43:**
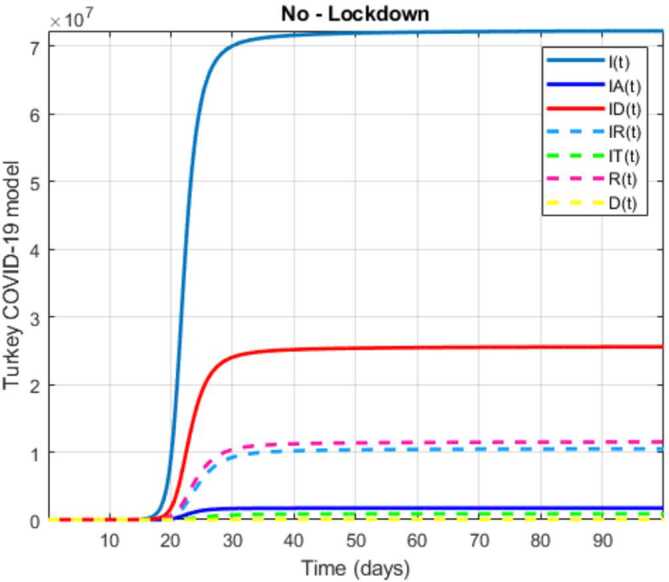
Numerical visualization for Covid-19 model in Turkey for $\alpha =0.9,\beta =0.75$

**Figure 44:**
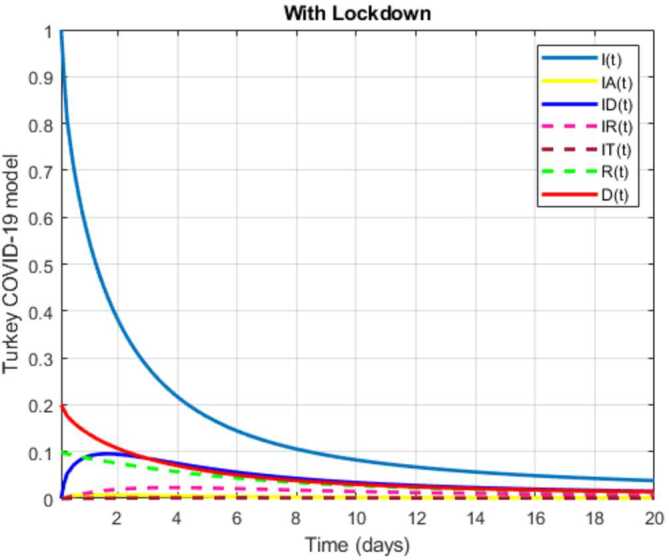
Numerical visualization for Covid-19 model in Turkey for $\alpha =0.7,\beta =0.75$

In Figs. 43 and 44, the initial conditions are chosen as

5.35 $$\begin{aligned} &S ( 0 ) = 81000000,\qquad I ( 0 ) =1,\qquad I_{A} ( 0 ) =1,\qquad I_{D} ( 0 ) =1,\qquad I_{R} ( 0 ) =1, \\ &I_{T} ( 0 ) = 1,\qquad R ( 0 ) =0,\qquad D ( 0 ) =0,\qquad V ( 0 ) =0. \end{aligned}$$

Also the parameters are

5.36 $$\begin{aligned} \begin{aligned} &\Lambda =80000000,\qquad k=2,\qquad p=0.5,\qquad \eta =0.12,\qquad \chi =0.015,\\ & v=0.027,\qquad x=0.4,\qquad \theta =0.301, \qquad \gamma = 0.09,\qquad \beta =0.013,\\ & \gamma _{1}=0.4,\qquad \mu _{1}=0.3,\qquad \varepsilon =0.161,\qquad \xi =0.015,\qquad\sigma =0.015,\\ & \tau = 0.0199,\qquad\Phi =0.2,\qquad \lambda =0.0345,\qquad\varphi =0.0345, \qquad\delta _{1}=0.01. \end{aligned} \end{aligned}$$

We present a numerical simulation for Covid-19 model in Figs. 43 and 44.

## Optimal control for Covid-19 model

Optimal control theory provides us important contributions in controlling Covid-19 outbreak. In this section, we will use 7 control variables as 7 possible control strategies to reach our aim. The control variable $u_{1}$ is the partial lockdown of schools, travel, universities, some businesses in Turkey. Also the government applies partial lockdown differentiated by age of people and sometimes states where the spread of virus is high. The control variable $u_{2}$ is the vaccination which is applied to susceptible individuals. The control variable $u_{3}$ is the information campaign to people that have symptoms but not have been tested. The control variable $u_{4}$ is the treatment for the infected individuals. The control variable $u_{5}$ is the personal protection which is achieved with masks, sanitizer, and other means. The control variable $u_{6}$ is the self-quarantine of exposed people. The control variable $u_{7}$ is the isolation of infected people.

We modify our model by adding these control variables:

6.1 $$\begin{aligned} &\overset{\cdot }{S} = \Lambda - \biggl( \frac{k_{1}pe^{-x}}{N} ( 1-u_{1} ) \bigl( I+w ( \beta I_{D}+\gamma I_{A}+\delta _{1}I_{R} ) \bigr) +\gamma _{1}+ \mu _{1} \biggr) S \\ &\phantom{\overset{\cdot }{S} =}{}-u_{5}S-u_{2}S+u_{7}I_{R}-u_{6}S, \\ &\overset{\cdot }{I}= \frac{k_{1}pe^{-x}}{N} ( 1-u_{1} ) \bigl( I+w ( \beta I_{D}+\gamma I_{A}+\delta _{1}I_{R} ) \bigr) S- ( \varepsilon +\xi +\lambda +\mu _{1} ) I, \\ &\overset{\cdot }{I_{A}}= \xi I- ( \theta +\mu +\chi + \mu _{1} ) I_{A}-u_{3}I_{A}, \\ &\overset{\cdot }{I_{D}} = \varepsilon I- ( \eta + \varphi + \mu _{1} ) I_{D}, \\ &\overset{\cdot }{I_{R}} = \eta I_{D}+\theta I_{A}- ( v+ \xi +\mu _{1} ) I_{R}-u_{7}I_{R}-u_{4}I_{R}+u_{6}S, \\ &\overset{\cdot }{I_{T}}= \mu I_{A}+vI_{R}- ( \sigma + \tau +\mu _{1} ) I_{T}, \\ &\overset{\cdot }{R}= \lambda I+\varphi I_{D}+\chi I_{A}+ \xi I_{R}+\sigma I_{T}- ( \Phi +\mu _{1} ) R+u_{4}I_{R}+u_{5}S+u_{3}I_{A} , \\ &\overset{\cdot }{D}= \tau I_{T}, \\ &\overset{\cdot }{V}= \gamma _{1}S+\Phi R-\mu _{1}V+u_{2}S. \end{aligned}$$

In this paper, we aim to minimize susceptible, infected, critically infected, asymptomatic people, and to maximize recovered people while minimizing the costs caused by the partial lockdown, vaccination, information campaign, treatment, personal protection, self-quarantine, and isolation. Thus, we construct the cost functional as follows:

6.2 $$\begin{aligned} &\min_{ ( u_{1},u_{2},u_{3},u_{4},u_{5},u_{6},u_{7} ) \in U}{ J} ( u_{1},u_{2},u_{3},u_{4},u_{5},u_{6},u_{7} ) \\ &\quad = \int _{0}^{T} \begin{pmatrix} \rho _{1}S+\rho _{2}I+\rho _{3}I_{R}+\rho _{4}I_{A}-\rho _{5}R+\pi _{1}u_{1}^{2}+ \pi _{2}u_{2}^{2} \\ +\pi _{3}u_{3}^{2}+\pi _{4}u_{4}^{2}+\pi _{5}u_{5}^{2}+\pi _{6}u_{6}^{2}+ \pi _{7}u_{7}^{2}\end{pmatrix} \,dt \end{aligned}$$

on the set of admissible controls

6.3 $$U=\left\{\begin{array}{c} \left(u_{1},u_{2},u_{3},u_{4},u_{5},u_{6},u_{7})\in L^{\infty }(0,T)\times L^{\infty }(0,T)\times L^{\infty }(0,T\right)\\ \times L^{\infty }(0,T)\times L^{\infty }(0,T)\times L^{\infty }(0,T)\times L^{\infty }(0,T):\\ 0\leq u_{1}(t)\leq {\tilde{u}}_{1},0\leq u_{2}(t)\leq {\tilde{u}}_{2},0\leq u_{3}(t)\leq {\tilde{u}}_{3},\\ 0\leq u_{4}(t)\leq {\tilde{u}}_{4},0\leq u_{5}(t)\leq {\tilde{u}}_{5},0\leq u_{6}(t)\leq {\tilde{u}}_{6},0\leq u_{7}(t)\leq {\tilde{u}}_{7} \end{array}\right\}.$$

The parameters $\rho _{1},\rho _{2},\rho _{3},\rho _{4},\rho _{5},\pi _{1},\pi _{2}, \pi _{3},\pi _{4},\pi _{5},\pi _{6},\pi _{7}$ represent the weighted parameters.

To show the existence of the optimal control for the problem under consideration, we notice that the set of admissible controls *U* is, by definition, closed and bounded. It is obvious that there is an admissible pair $( u_{1},u_{2},u_{3},u_{4},u_{5},u_{6},u_{7} ) $ for the problem. Hence, the existence of the optimal control comes as a direct result from the Filippove–Cesari theorem [17, 18].

We prove that the existence of an optimal control of an optimal control is guaranteed by providing the following conditions:

- The set of admissible controls is convex, bounded and closed.
- The set of controls and corresponding state variables is nonempty.
- The right-hand side of the state ODE system is bounded by a linear function in the state and control variables.
- The convexity of the integrand of cost functional with respect to *u* on the set *U*. The Hessian matrix of this functional is given by
  6.4 $$ H= \begin{bmatrix} 2\pi _{1} & 0 & 0 & 0 & 0 & 0 & 0 \\ 0 & 2\pi _{2} & 0 & 0 & 0 & 0 & 0 \\ 0 & 0 & 2\pi _{3} & 0 & 0 & 0 & 0 \\ 0 & 0 & 0 & 2\pi _{4} & 0 & 0 & 0 \\ 0 & 0 & 0 & 0 & 2\pi _{5} & 0 & 0 \\ 0 & 0 & 0 & 0 & 0 & 2\pi _{6} & 0 \\ 0 & 0 & 0 & 0 & 0 & 0 & 2\pi _{7}\end{bmatrix}. $$
  Since the Hessian of of this functional is everywhere positive definite, the functional $J ( u_{1},u_{2},u_{3},u_{4},u_{5},u_{6},u_{7} ) $ is strictly convex.

There exist a constant $\pi =\min \{ \pi _{1},\pi _{2,}\pi _{3},\pi _{4},\pi _{5},\pi _{6}, \pi _{7} \} >0$ such that the integrand of the cost functional satisfies

6.5 $$\begin{aligned} \widetilde{J} ( U ) ={}& \pi _{1}u_{1}^{2}+\pi _{2}u_{2}^{2}+ \pi _{3}u_{3}^{2}+ \pi _{4}u_{4}^{2}+\pi _{5}u_{5}^{2}+ \pi _{6}u_{6}^{2}+ \pi _{7}u_{7}^{2} \\ &{}+\rho _{1}S+\rho _{2}I+\rho _{3}I_{R}+ \rho _{4}I_{A}-\rho _{5}R \\ \geq{}& \pi _{1}u_{1}^{2}+\pi _{2}u_{2}^{2}+\pi _{3}u_{3}^{2}+ \pi _{4}u_{4}^{2}+ \pi _{5}u_{5}^{2}+ \pi _{6}u_{6}^{2}+\pi _{7}u_{7}^{2} \\ \geq{}& \pi \bigl( u_{1}^{2}+u_{2}^{2}+u_{3}^{2}+u_{4}^{2}+u_{5}^{2}+u_{6}^{2}+u_{7}^{2} \bigr) \end{aligned}$$

under the condition $\rho _{1}S+\rho _{2}I+\rho _{3}I_{R}+\rho _{4}I_{A}>\rho _{5}R$. Applying the Pontryagin’s maximum principle, we present the first order necessary conditions for an optimal solution for the considered optimal control problem. To achieve this, we construct the Hamiltonian *H*, which is given as

6.6 $$\begin{aligned} H ={}& \pi _{1}u_{1}^{2}+\pi _{2}u_{2}^{2}+ \pi _{3}u_{3}^{2}+\pi _{4}u_{4}^{2}+ \pi _{5}u_{5}^{2}+\pi _{6}u_{6}^{2}+ \pi _{7}u_{7}^{2} \\ &{}+\rho _{1}S+\rho _{2}I+\rho _{3}I_{R}+ \rho _{4}I_{A}-\rho _{5}R \\ &{}+\lambda _{1} \begin{pmatrix} \Lambda - ( \delta ( x ) ( 1-u_{1} ) ( I+w ( \beta I_{D}+\gamma I_{A}+\delta _{1}I_{R} ) ) +\gamma _{1}+\mu _{1} ) S \\ -u_{5}S-u_{2}S+u_{7}I_{R}-u_{6}S\end{pmatrix} \\ &{}+\lambda _{2} \bigl( \delta ( x ) ( 1-u_{1} ) \bigl( I+w ( \beta I_{D}+\gamma I_{A}+\delta _{1}I_{R} ) \bigr) S- ( \varepsilon +\xi +\lambda +\mu _{1} ) I \bigr) \\ &{}+\lambda _{3} \bigl( \xi I- ( \theta +\mu +\chi +\mu _{1} ) I_{A}-u_{3}I_{A} \bigr) \\ &{}+\lambda _{4} \bigl( \varepsilon I- ( \eta +\varphi +\mu _{1} ) I_{D} \bigr) \\ &{}+\lambda _{5} \bigl( \eta I_{D}+\theta I_{A}- ( v+\xi +\mu _{1} ) I_{R}-u_{7}I_{R}-u_{4}I_{R}+u_{6}S \bigr) \\ &{}+\lambda _{6} \bigl( \mu I_{A}+vI_{R}- ( \sigma +\tau +\mu _{1} ) I_{T} \bigr) \\ &{}+\lambda _{7} \bigl( \lambda I+\varphi I_{D}+\chi I_{A}+\xi I_{R}+ \sigma I_{T}- ( \Phi +\mu _{1} ) R+u_{4}I_{R}+u_{5}S+u_{3}I_{A} \bigr) \\ &{}+\lambda _{8} ( \gamma _{1}S+\Phi R-\mu _{1}V+u_{2}S ). \end{aligned}$$

Then there exists $\lambda \in \mathbb{R} ^{9}$ such that the first order necessary conditions for the existence of optimal control are given by the equations:

6.7 $$\begin{array}{rl} & \frac{d\lambda_{1}}{dt}=-\frac{\partial H}{\partial S}=-\left\{\begin{array}{c} \rho_{1}-\lambda_{1}\left(\begin{array}{c} \left(\delta (x)(1-u_{1})(I+w(\beta I_{D}+\gamma I_{A}+\delta_{1}I_{R}))+\gamma_{1}+\mu_{1}\right)\\ -u_{5}-u_{2}-u_{6} \end{array}\right)\\ +\lambda_{2}(\delta (x)(1-u_{1})(I+w(\beta I_{D}+\gamma I_{A}+\delta_{1}I_{R})))\\ +\lambda_{5}u_{6}+\lambda_{7}u_{5}+\lambda_{8}(u_{2}+\gamma_{1}) \end{array}\right\},\\ & \frac{d\lambda_{2}}{dt}=-\frac{\partial H}{\partial I}=-\left\{\begin{array}{c} \rho_{2}+\lambda_{1}(\delta (x)(1-u_{1})S)+\lambda_{2}(\delta (x)(1-u_{1})S-(\varepsilon +\xi +\lambda +\mu_{1}))\\ +\lambda_{3}\xi +\lambda_{4}\varepsilon +\lambda_{7}\lambda I \end{array}\right\},\\ & \frac{d\lambda_{3}}{dt}=-\frac{\partial H}{\partial I_{A}}=-\left\{\begin{array}{c} \rho_{4}+(\lambda_{1}+\lambda_{2})\delta (x)(1-u_{1})w\gamma S+\lambda_{3}(-(\theta +\mu +\chi +\mu_{1})-u_{3})\\ +\lambda_{5}\theta +\lambda_{6}\mu +\lambda_{7}(\chi +u_{3}) \end{array}\right\},\\ & \frac{d\lambda_{4}}{dt}=-\frac{\partial H}{\partial I_{D}}=-\left\{(\lambda_{1}+\lambda_{2})\delta (x)(1-u_{1})w\beta S-\lambda_{4}(\eta +\varphi +\mu_{1})+\lambda_{5}\eta +\lambda_{7}\varphi \right\},\\ & \frac{d\lambda_{5}}{dt}=-\frac{\partial H}{\partial I_{R}}=-\left\{\begin{array}{c} \rho_{3}+(\lambda_{1}+\lambda_{2})\delta (x)(1-u_{1})w\delta_{1}S+\lambda_{1}u_{7}\\ -\lambda_{5}((v+\xi +\mu_{1})+u_{7}+u_{4})+\lambda_{6}v+\lambda_{7}(\xi +u_{4}) \end{array}\right\},\\ & \frac{d\lambda_{6}}{dt}=-\frac{\partial H}{\partial I_{T}}=-\left\{-\lambda_{6}(\sigma +\tau +\mu_{1})+\lambda_{7}\sigma \right\},\\ & \frac{d\lambda_{7}}{dt}=-\frac{\partial H}{\partial R}=-\left\{-\rho_{5}-\lambda_{7}(\Phi +\mu_{1})+\lambda_{8}\Phi \right\},\\ & \frac{d\lambda_{8}}{dt}=-\frac{\partial H}{\partial V}=-\{-\lambda_{8}\mu_{1}\}. \end{array}$$

Hence the optimal controls are given as

6.8 $$\begin{aligned} &u_{1} = \frac{I ( t ) S ( t ) ( \lambda _{1}+\lambda _{2} ) }{2\pi _{1}} , \\ &u_{2} = \frac{S ( t ) ( \lambda _{1}-\lambda _{8} ) }{2\pi _{2}}, \\ &u_{3} = \frac{I_{A} ( t ) ( \lambda _{3}-\lambda _{7} ) }{2\pi _{3}}, \\ &u_{4} = \frac{I_{R} ( t ) ( \lambda _{5}-\lambda _{7} ) }{2\pi _{4}}, \\ &u_{5} = \frac{S ( t ) ( \lambda _{1}-\lambda _{7} ) }{2\pi _{5}}, \\ &u_{6} = \frac{S ( t ) ( \lambda _{1}-\lambda _{5} ) }{2\pi _{6}}, \\ &u_{7} = \frac{I_{R} ( t ) ( \lambda _{5}-\lambda _{1} ) }{2\pi _{7}}, \end{aligned}$$

and optimality conditions are given by

6.9 $$\begin{aligned} &u_{1}^{\ast } = \min \biggl\{ \widetilde{u}_{1}, \max \biggl\{ 0, \frac{I ( t ) S ( t ) ( \lambda _{1}+\lambda _{2} ) }{2\pi _{1}} \biggr\} \biggr\} , \\ &u_{2}^{\ast } = \min \biggl\{ \widetilde{u}_{2},\max \biggl\{ 0, \frac{S ( t ) ( \lambda _{1}-\lambda _{8} ) }{2\pi _{2}} \biggr\} \biggr\} , \\ &u_{3}^{\ast } = \min \biggl\{ \widetilde{u}_{3},\max \biggl\{ 0, \frac{I_{A} ( t ) ( \lambda _{3}-\lambda _{7} ) }{2\pi _{3}} \biggr\} \biggr\} , \\ &u_{4}^{\ast } = \min \biggl\{ \widetilde{u}_{4}, \max \biggl\{ 0, \frac{I_{R} ( t ) ( \lambda _{5}-\lambda _{7} ) }{2\pi _{4}} \biggr\} \biggr\} , \\ &u_{5}^{\ast } = \min \biggl\{ \widetilde{u}_{5}, \max \biggl\{ 0, \frac{S ( t ) ( \lambda _{1}-\lambda _{7} ) }{2\pi _{5}} \biggr\} \biggr\} , \\ &u_{6}^{\ast } = \min \biggl\{ \widetilde{u}_{6}, \max \biggl\{ 0, \frac{S ( t ) ( \lambda _{1}-\lambda _{5} ) }{2\pi _{6}} \biggr\} \biggr\} , \\ &u_{7}^{\ast } = \min \biggl\{ \widetilde{u}_{7}, \max \biggl\{ 0, \frac{I_{R} ( t ) ( \lambda _{5}-\lambda _{1} ) }{2\pi _{7}} \biggr\} \biggr\} . \end{aligned}$$

## Discussion, recommendations, and conclusions

The Covid-19 fatality on mankind prompted undertaking serious investigations covering various aspects within several fields of science, technology, and engineering in the last 4 months. While researchers have obtained some successful results, they are still struggling to get an effective vaccine that could prevent the spread of the deadly Covid-19 among human beings. From December 2019 to 30 April 2020, there were 3441767 confirmed infected cases, 1097858 recovered, and 243922 deaths worldwide, among which 6336 confirmed cases, 2549 recovered, and 123 deaths were recorded in South Africa, while 124 375 confirmed cases, 58259 recovered, and 3336 deaths in Turkey. South Africa registered its first confirmed case of Covid-19 before Turkey on 5 March 2020, while Turkey witnessed its first case on 11 March 2020. The unfolding of the spread of Covid-19 in both countries has defeated the general expectations that South Africa would record more infections and deaths compared to Turkey. As a result, endless scientific questions were asked within different fields of science, which impelled the compilation of this paper to present critical and comprehensive studies with cases studied in South Africa and Turkey in particular. Although both countries have put in place severe measures to protect their citizens, the predictions from the suggested mathematical models and statistical analysis show two different patterns for both countries. For instance, in Turkey, a high and exponential growth in newly infected from 11 March 2020 to 11 April 2020 was observed due to late implementation of the lockdown regulations; however, from 12 April 2020 to 2 May 2020 this country has observed an exponential decay in the daily numbers of new infected cases. Thus, Turkey curve seems to follow a lognormal distribution, which, of course, could mean that it is winning the Covid-19 war or that it took control of the situation. As a result, it is possible that Turkey in the next few months could end the spread of Covid-19, if it maintains the energy and adheres to the measures in place to combat this virus. However, if it relaxes, the prediction from reliability level method indicated that Turkey could see a very rapid exponential growth in numbers of daily deaths and new infections. Furthermore, it is observed that the exponential decay in the daily number of new infected and death cases corresponds to the period of lockdown implementation and the stringent rules put in place by the Turkish government, by which the contravening to the rule is punishable with a monetary fine. On the other hand, in South Africa, although the numbers are not as high as those of Turkey, three phases are observed from statistical results. The first phase goes from 5 March 2020 to 27 March 2020, where the country witnessed an exponential growth in numbers of new infected and deaths daily; and it corresponds to a pre-lockdown period. The second phase began on 28 March 2020 and lasted until 18 April 2020, in which the country observed a slow increase of new infected and dead daily; a period corresponding to lockdown period enforced with the presence of South Africa Defence Force. The last phase ranges from 19 April 2020 to 2 May 2020, in which the country observed an exponential growth in numbers of new infected and dead per day. This exponential growth is attributed to the relaxation and disobedience of lockdown regulations, probably due to economic breakdown, increasing poverty effects among the larger population, and also due to migration from level five to level four on 1 May 2020. Therefore, as the provision of a suitable vaccine to save and protect human beings, the wrath and fatality of the deadly Covid-19, which broke-out in Wuhan, China, in December 2019 is delayed, it is clearly evident that the exponential growth in numbers of new infected can only be stopped or halted by enforcing the implementation of social distancing and ensuring that people do frequently wash their hands upon touching any object or even animals, whether infected with Covid-19 or not. Additionally, the wearing of masks should be adopted in public places to avoid the spreading of the virus, in case the social distancing rules are not being kept. It is further paramount that the medical workers in charge of Covid-19 patients are well protected to minimize the contraction of the virus from the patients and passing it on to the general public. In addition to the prohibition of alcohol sales and usage in public places, public smoking should also be prohibited in the effort to combat the spread of Covid-19. Moreover, the statistical analysis results, specifically the reliability level prediction and the results obtained from suggested mathematical models, indicated that without social distancing restrictions or clear implementation of lockdown regulations, it will be impossible for countries to control the spread of Covid-19. This implies that the number of new infected and deaths per day would be difficult to contain, resulting in the fight against the virus getting out of hand. These outcomes from reliability level are therefore indicated in blue lines in Figs. 18, 19, 20, 29, and 30 those from reliability level. The suggested mathematical models with different differential operators, including classical and nonlocal operators in the last 12 figures, considered with lockdown and no-lockdown and presented for different fractional orders, also confirmed the results obtained from the reliability level. In consideration of all prediction results, it is concluded that South Africa has not yet won the war against Covid-19 and serious outbreaks are expected in the near future as the climate season changes to winter. Cold seasons are scientifically proven to be a thriving climate for the survival of coronavirus. Therefore to avoid this foreseen crisis, social distancing must be a responsibility of each person living within the Republic of South Africa, and the transition from level 5 to level 1 should be implemented very wisely.

## Data Availability

There are no data for this paper.
